# Supplementary material for: Organocatalytic enantio- and diastereoselective cycloetherification via dynamic kinetic resolution of chiral cyanohydrins
Source: Nat Commun. 2017 Nov 9;8:1397. doi: 10.1038/s41467-017-01099-x (PMC5680189; doi:10.1038/s41467-017-01099-x)
Supplement: Supplementary file 1 — Supplementary Information [file 41467_2017_1099_MOESM1_ESM.pdf]

Supplementary Methods

Instrumentation and Chemicals

^{1}H and ^{13}C Nuclear magnetic resonance spectra were taken on a Varian UNITY INOVA 500 (^{1}H, 500 MHz; ^{13}C, 125.7 MHz) spectrometer using tetramethylsilane as an internal standard for ^{1}H NMR (δ = 0 ppm) and $CDCl_3$ as an internal standard for ^{13}C NMR (δ = 77.0 ppm). ^{1}H NMR data are reported as follows: chemical shift, multiplicity (s = singlet, d = doublet, t = triplet, q = quartet, quint = quintet, sext = sextet, sept = septet, br = broad, m = multiplet), coupling constants (Hz), integration. ^{19}F NMR spectra were measured on a Varian Mercury 200 (^{19}F, 188 MHz) spectrometer with hexafluorobenzene as an internal standard (δ = 0 ppm). Mass spectra were recorded on a SHIMADZU GCMS-QP2010 Plus (EI) and a Thermo Scientific Exactive (ESI, APCI) spectrometers. High performance liquid chromatography (HPLC) was performed with a SHIMADZU Prominence. Gas chromatography (GC) was performed with a SHIMADZU GC-2014 Gas Chromatograph. Infrared (IR) spectra were determined on a SHIMADZU IR Affinity-1 spectrometer. Melting points were determined using a YANAKO MP-500D. Optical rotations were measured on a HORIBA SEPA-200. X-ray data were taken on a Rigaku XtalAB mini diffractometer equipped with a CCD detector. TLC analyses were performed by means of Merck Kieselgel 60 F_{254} (0.25 mm) Plates. Visualization was accomplished with UV light (254 nm) and/or such as an aqueous alkaline $KMnO_4$ solution followed by heating.

Flash column chromatography was carried out using Kanto Chemical silica gel (spherical, 40–50 μm). Unless otherwise noted, commercially available reagents were used without purification.

Experimental Procedure

General procedure for asymmetric synthesis of tetrahydropyrans 3

Substrate **1** (0.15 mmol), CH_2Cl_2 (0.30 mL), **4a** (6.8 mg, 0.015 mmol), and acetone cyanohydrin (**2**, 0.30 mmol) were sequentially added to a 5-mL vial. The mixture was stirred in an oil bath maintained at 25 °C for 24 h. The reaction mixture was subsequently diluted with hexane/EtOAc (v/v = 1/1), passed through a short silica gel pad to remove **4a**, and concentrated in vacuo. Purification of the reaction mixture by flash silica gel column chromatography using CH_2Cl_2/hexane (v/v = 20/1) and then hexane/EtOAc (v/v = 3/1–10/1) as an eluent afforded the corresponding tetrahydropyrans **3**.

Racemic compounds were prepared using triethylamine and 1,3-bis[3,5-bis(trifluoromethyl)-phenyl]thiourea as catalysts.

Procedure for reaction of ketone 5 with 2

1-Phenylpentan-1-one (**5**, 24.8 μL, 0.15 mmol), CH_2Cl_2 (0.30 mL), **4a** (6.8 mg, 0.015 mmol), and acetone cyanohydrin (**2**, 0.30 mmol) were sequentially added to a 5-mL vial. The mixture was stirred in an oil bath maintained at 25 °C for 24 h. The reaction mixture was subsequently diluted with hexane/EtOAc (v/v = 1/1), passed through a short silica gel pad to remove **4a**, and concentrated in vacuo. However, purification of the reaction mixture by flash silica gel column chromatography using hexane/EtOAc (v/v = 30/1) as an eluent failed to afford **6**.

Procedure for reaction of ketone 5 with trimethylsilylcyanide

1-Phenylpentan-1-one (**5**, 24.8 μL, 0.15 mmol), CH_2Cl_2 (0.30 mL), **4a** (6.8 mg, 0.015 mmol), and trimethylsilylcyanide (37.2 μL, 0.30 mmol) were sequentially added to a 5-mL vial. The mixture was stirred in an oil bath maintained at 25 °C for 24 h. The reaction mixture was subsequently diluted with hexane/EtOAc (v/v = 1/1), passed through a short silica gel pad to remove **4a**, and concentrated in vacuo. Purification of the reaction mixture by flash silica gel column chromatography using hexane/EtOAc (v/v = 30/1) as an eluent afforded **7**.

Procedure for preparation of bifunctional catalysts 4a

(1*S*,2*S*)-Cyclohexane-1,2-diamine (1.1 g, 10 mmol) and $NaBH(OAc)_3$ (7.2 g, 34 mmol) were dissolved in 1,2-dichloroethane (60 mL), and the solution was cooled to 0 °C. To the resulting solution was added dropwise glutaraldehyde (ca. 50% in H_2O, 2.0 mL, 11 mmol) at 0 °C. The mixture was allowed to warm to ambient temperature. After being stirred for 3 h, 10% aqueous NaOH (40 mL) was added at 0 °C. The aqueous phase was separated and washed with CH_2Cl_2 (25 mL × 3). The combined organic layers were washed with brine and dried over Na_2SO_4, and concentrated in vacuo. Purification by flash silica gel column chromatography using $CHCl_3$/MeOH/Et_3N (v/v = 20/20/1) as an eluent gave (1*S*,2*S*)-2-(piperidin-1-yl)cyclohexan-1-amine (0.81 g, 4.5 mmol). Next, to the solution of the obtained (1*S*,2*S*)-2-(piperidin-1-yl)cyclohexan-1-amine in THF (13 mL) was slowly added 3,5-bis(trifluoromethyl)phenyl isothiocyanate (1.2 g, 4.5 mmol) at 0 °C. The mixture was stirred overnight, and the solvents were removed in vacuo. Purification by flash silica gel column chromatography using $CHCl_3$/CH_3OH (v/v = 20/1) as an eluent gave bifunctional organocatalyst **4a**. The characterization results are as below.

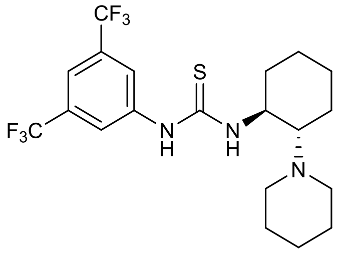

4a. White solid; 99% yield (for 2steps, 0.44 g). $[\alpha]_D^{23}$ +1.72 (*c* 1.63, CH_2Cl_2). ^1H NMR ($CDCl_3$) δ 7.84 (s, 2H), 7.70 (s, 1H), 3.77 (br s, 1H), 2.64 (m, 3H), 2.39 (m, 3H), 1.94–1.73 (m, 5H), 1.70–1.42 (m, 10H). ^{13}C NMR ($CDCl_3$) δ 181.4, 139.7, 132.7 (q, *J* = 33.9 Hz), 124.5, 122.9 (q, *J* = 272.8 Hz), 119.0, 68.8, 56.2, 49.5, 32.6, 26.3, 25.2, 24.4, 24.2, 23.4. Mp. 128.0–128.6 °C. IR (KBr): 3235, 3060, 2949, 2809, 1547, 1474, 1377, 1280, 1180, 1142, 1010, 969, 888, 706, 690 cm^{-1}. HRMS Calcd for $C_{20}H_{26}F_6N_3S$: $[M+H]^+$, 454.1746. Found: *m*/*z* 454.1737.

General procedure for the preparation of bifunctional catalysts 4d–4g

Bifunctional organocatalysts **4d**–**4g** were prepared by the literature procedure.[1] A cinchona alkaloid (5.0 mmol) and triphenylphosphine (1.6 g, 6.0 mmol) were dissolved in THF (25 mL), and the solution was cooled to 0 °C. Diethyl azodicarboxylate (1.0 g,

6.0 mmol) was subsequently added. To the resulting solution was added dropwise the solution of diphenyl phosphoryl azide (1.3 mL, 6.0 mmol) in THF (10 mL) at 0 °C. The mixture was allowed to warm to ambient temperature. After being stirred for 24 h, it was heated to 50 °C and stirred for 10 h. Triphenylphosphine (1.7 g, 6.5 mmol) was added again, and the mixture was stirred at 50 °C for additional 15 h. After the solution was cooled to ambient temperature, H_2O (0.50 mL) was added, and the solution was stirred for 24 h. The solvents were removed in vacuo, and the residue was dissolved in CH_2Cl_2/10% aqueous HCl (25 mL/25 mL). The aqueous phase was separated and washed with CH_2Cl_2 (25 mL × 4). It was subsequently made alkaline with aqueous NH_3, and the aqueous phase was extracted with CH_2Cl_2 (25 mL × 4). The combined organic layers were dried over Na_2SO_4, and concentrated in vacuo. Purification by flash silica gel column chromatography using EtOAc/CH_3OH (v/v = 9/1) then $CHCl_3$/CH_3OH (v/v = 8/2) as an eluent gave the corresponding 9-amino(9-deoxy)cinchona alkaloids. Next, to the solution of the obtained 9-amino(9-deoxy)cinchona alkaloid in THF (6.0 mL) was slowly added a solution of 3,5-bis(trifluoromethyl)phenyl isothiocyanate (1.0 equiv) in THF (4.0 mL) at ambient temperature. The mixture was stirred overnight, and the solvents were removed in vacuo. Purification by flash silica gel column chromatography using EtOAc/CH_3OH (v/v = 95/5–97.5/2.5) or EtOAc as an eluent gave the corresponding bifunctional organocatalysts **4**. The characterization results are as below.

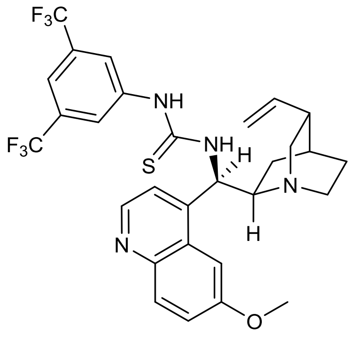

4d. White solid; 41% yield (for 2steps from quinidine, 1.2 g). $[\alpha]_D^{23}$ +122.6 (*c* 1.33, CH_2Cl_2). ^{1}H NMR ($CDCl_3$) δ 8.65 (br s, 1H), 8.02 (d, *J* = 9.0 Hz, 1H), 7.86 (s, 2H), 7.67 (s, 1H), 7.59 (br s, 1H), 7.40 (d, *J* = 9.0 Hz, 1H), 7.23 (br s, 1H), 5.86 (br s, 2H), 5.19 (br s, 1H), 5.15 (d, *J* = 9.5 Hz, 1H), 3.97 (s, 3H), 3.22 (br s, 1H), 3.10 (br s, 1H), 3.03 (m, 2H), 2.94 (m, 1H), 2.38 (m, 1H), 1.70 (s, 1H), 1.61 (m, 2H), 1.27 (br s, 1H), 1.02 (m, 1H). ^{13}C NMR ($CDCl_3$) δ 181.0, 158.1, 147.3, 144.7, 144.5, 140.1, 139.6, 132.5 (q, *J* = 33.6 Hz), 131.6, 128.0, 123.5, 122.9 (q, *J* = 273.0 Hz), 122.3, 118.7, 115.3, 101.7,

61.4, 55.6, 48.5, 47.1, 38.7, 27.1, 26.1, 25.0. Mp. 125.0–125.2 °C. IR (KBr): 3221, 2944, 2361, 1735, 1623, 1511, 1475, 1384, 1278, 1177, 1134, 1034, 959, 916, 884, 850, 826, 682 cm^{-1}. HRMS Calcd for $C_{29}H_{29}F_6N_4OS$: $[M+H]^+$, 595.1966. Found: *m*/*z* 595.1961.

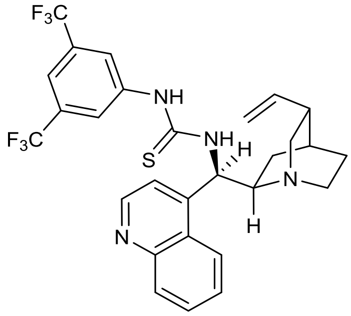

4e. White solid; 36% yield (for 2steps from cinchonine, 1.0 g). $[\alpha]_D^{23}$ +163.3 (*c* 1.23, CH_2Cl_2). ^1H NMR ($CDCl_3$) δ 8.83 (br s, 1H), 8.28 (br s, 1H), 8.15 (d, *J* = 8.5 Hz, 1H), 7.85 (br s, 2H), 7.56 (dd, *J* = 7.5, 7.5 Hz, 1H), 7.68 (s, 1H), 7.64 (dd, *J* = 7.5, 7.5 Hz, 1H), 7.29 (br s, 1H), 5.81 (br s, 2H), 5.14 (m, 2H), 3.21 (br s, 1H), 3.00 (m, 3H), 2.92 (br s, 1H), 2.36 (m, 1H), 1.66 (s, 1H), 1.59 (m, 2H), 1.22 (br s, 1H), 0.95 (m, 1H). ^{13}C NMR ($CDCl_3$) δ 181.3, 150.0, 148.6, 145.8, 140.2, 139.3, 132.5 (q, *J* = 33.6 Hz), 130.5, 129.5, 127.1, 126.7, 123.4, 122.9 (q, *J* = 273.1 Hz), 122.8, 119.0, 118.7, 115.5, 61.8, 55.7, 48.5, 47.0, 38.9, 27.3, 26.0, 24.9. Mp. 189.9–190.3 °C. IR (KBr): 3428, 3246, 2944, 2360, 1622, 1588, 1512, 1474, 1386, 1281, 1183, 1126, 960, 882, 848, 752, 682 cm^{-1}. HRMS Calcd for $C_{28}H_{27}F_6N_4S$: $[M+H]^+$, 565.1861. Found: *m*/*z* 565.1855.

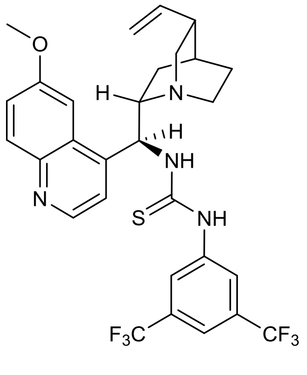

4f. White solid; 27% yield (for 2steps from quinine, 0.80 g). $[\alpha]_D^{23}$ –99.0 (*c* 1.24, CH_2Cl_2). ^1H NMR ($CDCl_3$) δ 8.60 (br s, 1H), 8.00 (d, *J* = 8.5 Hz, 1H), 7.82 (br s, 2H), 7.68 (s, 1H), 7.62 (br s, 1H), 7.39 (d, *J* = 8.5 Hz, 1H), 7.18 (br s, 1H), 5.84 (br s, 1H), 5.70 (m, 1H), 5.01 (m, 2H), 3.96 (s, 3H), 3.37 (br s, 1H), 3.30 (br s, 1H), 3.18 (m, 1H), 2.79 (br s, 2H), 2.35 (br s, 1H), 1.72 (s, 1H), 1.68 (m, 2H), 1.41 (m, 1H), 0.92 (br s, 1H).

^{13}C NMR ($CDCl_3$) δ 181.0, 158.2, 147.4, 144.8, 144.0, 140.6, 140.0, 132.6 (q, *J* = 33.6 Hz), 131.8, 127.9, 123.6, 122.9 (q, *J* = 273.0 Hz), 122.0, 118.8, 115.1, 102.1, 61.2, 55.7, 54.9, 41.3, 39.0, 27.5, 27.1, 25.7. Mp. 121.0–121.5 °C. IR (neat): 3220, 2946, 2360, 1623, 1510, 1475, 1384, 1279, 1180, 1134, 1032, 959, 917, 885, 850, 683 cm^{-1}. HRMS Calcd for $C_{29}H_{29}F_6N_4OS$: $[M+H]^+$, 595.1966. Found: *m*/*z* 595.1961.

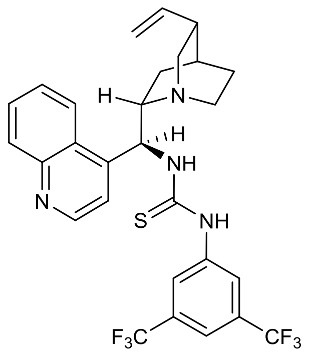

4g. White solid; 44% yield (for 2steps from cinchonidine, 1.2 g). $[\alpha]_D^{23}$ –101.0 (*c* 1.24, CH_2Cl_2). ^{1}H NMR ($CDCl_3$) δ 8.80 (br s, 1H), 8.35 (br s, 1H), 8.14 (d, *J* = 8.5 Hz, 1H), 7.80 (s, 2H), 7.74 (dd, *J* = 8.0, 7.5 Hz, 1H), 7.69 (s, 1H), 7.63 (dd, *J* = 8.0, 7.5 Hz, 1H), 7.27 (br s, 1H), 5.78 (br s, 1H), 5.67 (m, 1H), 4.98 (m, 2H), 3.26 (m, 1H), 3.20 (br s, 1H), 3.17 (dd, *J* = 13.5, 10.5 Hz, 1H), 2.78 (m, 2H), 2.33 (br s, 1H), 1.70 (m, 2H), 1.63 (m, 1H), 1.33 (m, 1H), 0.93 (br s, 1H). ^{13}C NMR ($CDCl_3$) δ 180.9, 149.9, 148.5, 145.9, 140.7, 139.9, 132.6 (q, *J* = 33.6 Hz), 130.4, 129.5, 127.0, 123.6, 122.9 (q, *J* = 273.0 Hz), 119.1, 118.9, 115.0, 61.5, 56.5, 54.9, 41.1, 39.2, 27.5, 27.1, 25.7. Mp. 122.8–123.1 °C. IR (neat): 3240, 3081, 2946, 2366, 1510, 1473, 1384, 1281, 1181, 1135, 990, 958, 884, 849, 755, 683 cm^{-1}. HRMS Calcd for $C_{28}H_{27}F_6N_4S$: $[M+H]^+$, 565.1861. Found: *m*/*z* 565.1855.

4b and **4c** were commercially available and used without any purification.

General procedures for preparation of substrates

*Procedure for preparation of **1a**–**1f**, and **1h***

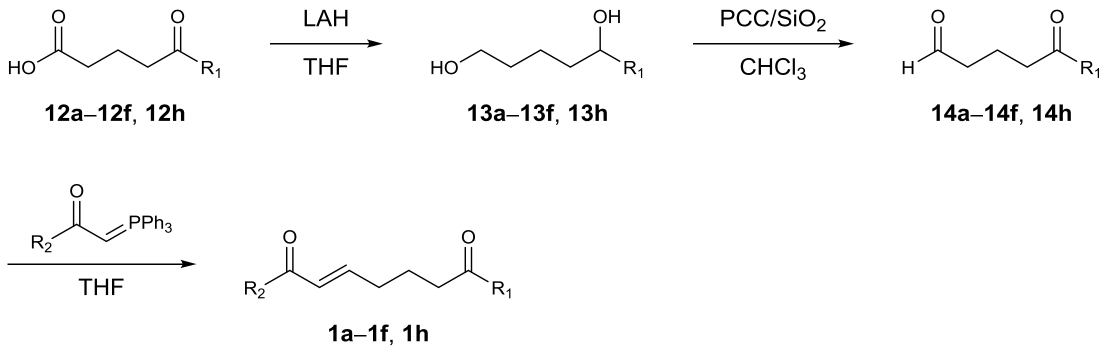

<u>General procedure for preparation of **13**</u>

Under argon atmosphere, a solution of **12** (26 mmol) in THF (10 mL) was added dropwise to a suspention of $LiAlH_4$ (2.96 g, 78 mmol) in THF (80 mL) at 0 °C. The mixture was allowed to warm to ambient temperature and stirred for 8 h. Next, H_2O (2.96 mL), 15% aqueous NaOH (2.96 mL), and H_2O (8.88 mL) were sequentially added dropwise at 0 °C, and the mixture was sttired for a while at ambient temperature. Subsequently, the reaction mixture was filtered through a Celite pad, and the Celite pad was washed with Et_2O. The filtrate was concentrated in vacuo to afford the corresponding diols **13**, which was used for the next step without further purification.

<u>General procedure for preparation of **14**</u>

To a round-bottom flask were added sequentially **13** (1.0 equiv), CH_2Cl_2 (0.26 M), and PCC (4.0 equiv)/SiO_2. After being stirred for 9 h at ambient temperature, the crude reaction mixture was filtered through a Celite pad, and the Celite pad was washed with CH_2Cl_2. The filtrate was concentrated in vacuo. Purification by flash silica gel column chromatography using hexane/EtOAc (v/v = 5/1–3/1) as an eluent gave **14** (**14a**–**14f**: 28%; **14h**: 22% for 2 steps).

<u>General procedure for preparation of **1a**–**1f**, and **1h**</u>

14 (1.0 equiv) and a stabilized ylide (3.0 equiv) were dissolved in THF (0.50 M), and the solution was refluxed in an oil bath maintained at 90 °C overnight. After the solution was cooled to ambient temperature, solvents were removed in vacuo. Purification by flash silica gel column chromatography using hexane/EtOAc (v/v = 3/1–8/1) as an eluent gave the corresponding **1**.

(*E*)-1,7-Diphenylhept-2-ene-1,7-dione (1a): CAS RN [1181701-34-7].

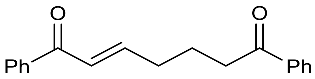

White solid; 47% yield (for the last step).

^{1}H NMR ($CDCl_3$) δ 7.96 (m, 2H), 7.92 (m, 2H), 7.58–7.54 (m, 2H), 7.48–7.45 (m, 4H), 7.08 (dt, *J* = 15.5, 7.0 Hz, 1H), 6.93 (dt, *J* = 15.5, 1.5 Hz, 1H), 3.05 (t, *J* = 7.0 Hz, 2H), 2.44 (m, 2H), 2.01 (m, 2H). ^{13}C NMR ($CDCl_3$) δ 199.6, 190.1, 148.7, 137.8, 136.8, 133.1, 132.7, 128.6, 128.5 (2C), 128.0, 126.4, 37.5, 32.1, 22.4.

(*E*)-1-(4-Methoxyphenyl)-7-phenylhept-2-ene-1,7-dione (1b).

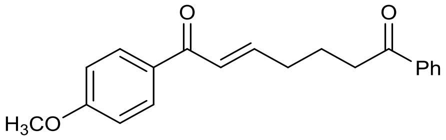

White solid; 40% yield (for the last step).

^{1}H NMR ($CDCl_3$) δ 7.97–7.93 (m, 4H), 7.56 (m, 1H), 7.46 (m, 2H), 7.06 (dt, *J* = 15.0, 7.0 Hz, 1H), 6.96–6.92 (m, 3H), 3.87 (s, 3H), 3.05 (t, *J* = 7.0 Hz, 2H), 2.43 (m, 2H), 2.01 (m, 2H). ^{13}C NMR ($CDCl_3$) δ 199.6, 188.9, 163.3, 147.6, 136.8, 133.1, 130.8, 130.6, 128.6, 128.0, 126.1, 113.7, 55.5, 37.5, 32.0, 22.5. Mp. 45.1–45.6 °C. TLC: R_f 0.19 (hexane/EtOAc = 5:1). IR (KBr): 3069, 3015, 2957, 2933, 2838, 2367, 1679, 1661, 1615, 1607, 1577, 1510, 1459, 1448, 1421, 1371, 1348, 1310, 1255, 1233, 1200, 1178, 1111, 1023, 973, 935, 878, 856, 829, 779, 764, 738, 692 cm^{-1}. HRMS Calcd for $C_{20}H_{21}O_3$: $[M+H]^+$, 309.1485. Found: *m*/*z* 309.1477.

(*E*)-7-Phenyl-1-(4-(trifluoromethyl)phenyl)hept-2-ene-1,7-dione (1c).

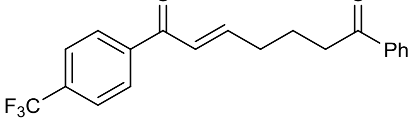

White solid; 39% yield (for the last step).

^{1}H NMR ($CDCl_3$) δ 8.00 (d, *J* = 8.0 Hz, 2H), 7.96 (m, 2H), 7.73 (d, *J* = 8.0 Hz, 2H), 7.57 (m, 1H), 7.47 (m, 2H), 7.12 (dt, *J* = 15.0, 6.5 Hz, 1H), 6.89 (dt, *J* = 15.0, 1.5 Hz, 1H), 3.06 (t, *J* = 7.0 Hz, 2H), 2.46 (m, 2H), 2.02 (m, 2H). ^{13}C NMR ($CDCl_3$) δ 199.4, 189.8, 150.3, 140.1, 136.8, 133.9 (q, *J* = 32.6 Hz), 133.2, 128.8, 128.6, 128.0, 126.1, 125.6 (q, *J* = 3.9 Hz), 123.6 (q, *J* = 272.6 Hz), 37.5, 32.2, 22.4. ^{19}F NMR ($CDCl_3$) δ 98.7. Mp. 73.2–73.7 °C. TLC: R_f 0.44 (hexane/EtOAc = 3:1). IR (KBr): 2940, 2351, 1680, 1669,

1619, 1614, 1597, 1588, 1512, 1452, 1422, 1410, 1324, 1312, 1303, 1235, 1170, 1157, 1120, 1112, 1069, 1016, 998, 965, 960, 868, 850, 842, 825, 794, 769, 757, 691 cm^{-1}. HRMS Calcd for $C_{20}H_{18}F_3O_2$: $[M+H]^+$, 347.1253. Found: *m*/*z* 347.1246.

(*E*)-7-Phenyl-1-(thiophen-2-yl)hept-2-ene-1,7-dione (1d).

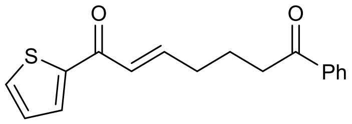

White solid; 56% yield (for the last step).
^{1}H NMR ($CDCl_3$) δ 7.97 (m, 2H), 7.75 (dd, *J* = 4.0, 1.5 Hz, 1H), 7.65 (dd, *J* = 5.0, 1.5 Hz, 1H), 7.56 (dd, *J* = 7.0, 1.5 Hz, 1H), 7.47 (m, 2H), 7.15 (dd, *J* = 5.0, 4.0 Hz, 1H), 7.14 (dt, *J* = 15.0, 7.0 Hz, 1H), 6.84 (dt, *J* = 15.0, 1.5 Hz, 1H), 3.05 (t, *J* = 7.0 Hz, 2H), 2.43 (m, 2H), 2.01 (m, 2H). ^{13}C NMR ($CDCl_3$) δ 199.6, 182.1, 147.9, 145.1, 136.8, 133.8, 133.1, 131.9, 128.6, 128.2, 128.0, 125.9, 37.5, 31.9, 22.5. Mp. 42.7–43.2 °C. TLC: R_f 0.33 (hexane/EtOAc = 3:1). IR (KBr): 3083, 2955, 2374, 1679, 1654, 1610, 1596, 1517, 1458, 1448, 1418, 1371, 1354, 1302, 1258, 1244, 1230, 1199, 1182, 1081, 1068, 1002, 972, 956, 854, 836, 799, 777, 736, 719, 709 cm^{-1}. HRMS Calcd for $C_{17}H_{17}O_2S$: $[M+H]^+$, 285.0944. Found: *m*/*z* 285.0937.

(*E*)-1-Phenyloct-5-ene-1,7-dione (1e).

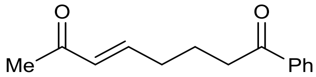

White solid; 41% yield (for the last step).
^{1}H NMR ($CDCl_3$) δ 7.95 (m, 2H), 7.57 (m, 1H), 7.46 (m, 2H), 6.81 (dt, *J* = 16.5, 7.0 Hz, 1H), 6.10 (dt, *J* = 16.5, 1.5 Hz, 1H), 3.01 (t, *J* = 7.0 Hz, 2H), 2.34 (m, 2H), 2.24 (s, 3H), 1.95 (m, 2H). ^{13}C NMR ($CDCl_3$) δ 199.4, 198.6, 147.3, 136.7, 133.1, 131.8, 128.6, 127.9, 37.5, 31.8, 26.9, 22.3. Mp. 44.0–44.5 °C. TLC: R_f 0.30 (hexane/EtOAc = 3:1). IR (KBr): 3066, 3042, 2998, 2935, 2893, 2363, 1679, 1669, 1666, 1640, 1629, 1596, 1582, 1456, 1418, 1362, 1306, 1281, 1262, 1203, 1091, 1081, 1064, 1002, 980, 758, 716, 693, 656 cm^{-1}. HRMS Calcd for $C_{14}H_{17}O_2$: $[M+H]^+$, 217.1223. Found: *m*/*z* 217.1219.

***S*-Phenyl (*E*)-7-oxo-7-phenylhept-2-enethioate (1f)**.

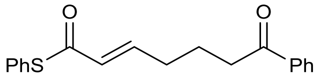

White solid; 62% yield (for the last step).
^{1}H NMR ($CDCl_3$) δ 7.98 (m, 2H), 7.58 (m, 1H), 7.48 (m, 2H), 7.50–7.46 (m, 2H), 7.41–

7.46 (m, 5H), 3.03 (t, J = 7.0 Hz, 2H), 2.36 (m, 2H), 1.97 (m, 2H). ^{13}C NMR ($CDCl_3$) δ 199.4, 188.0, 145.6, 136.8, 134.6, 133.1, 129.4, 129.1, 128.6, 128.4, 128.0, 127.5, 37.4, 31.6, 22.2. Mp. 43.6–44.1 °C. TLC: R_f 0.36 (hexane/EtOAc = 5:1). IR (KBr): 2980, 2947, 2362, 1696, 1680, 1635, 1441, 1414, 1352, 1286, 1262, 1201, 1041, 975, 923, 811, 756, 687 cm^{-1}. HRMS Calcd for $C_{19}H_{19}O_2S$: [M+H]$^+$, 311.1100. Found: m/z 311.1091.

(*E*)-7-(4-Fluorophenyl)-1-phenylhept-2-ene-1,7-dione (1h).

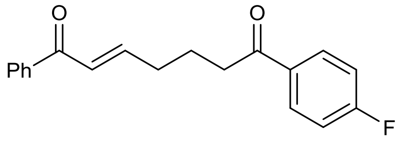

Yellow solid; 12% yield (for the last step).
^{1}H NMR ($CDCl_3$) δ 7.99 (m, 2H), 7.92 (m, 2H), 7.56 (m, 1H), 7.47 (m, 2H), 7.13 (m, 2H), 7.08 (dt, J = 15.0, 7.0 Hz, 1H), 6.93 (dt, J = 15.0, 1.5 Hz, 1H), 3.02 (t, J = 7.0 Hz, 2H), 2.44 (m, 2H), 2.00 (m, 2H). ^{13}C NMR ($CDCl_3$) δ 197.9, 190.6, 165.7 (d, J = 254.3 Hz), 148.6, 137.7, 133.2 (d, J = 3.4 Hz), 132.7, 130.6 (d, J = 9.1 Hz), 128.53, 128.51, 126.5, 115.7 (d, J = 22.0 Hz), 37.4, 32.0, 22.4. ^{19}F NMR ($CDCl_3$) δ 56.5. Mp. 45.5–46.0 °C. TLC: R_f 0.50 (hexane/EtOAc = 3:1). IR (KBr): 3068, 2956, 2905, 2873, 2367, 1684, 1663, 1617, 1597, 1580, 1506, 1458, 1448, 1432, 1412, 1361, 1316, 1299, 1254, 1241, 1227, 1220, 1197, 1158, 1100, 1072, 1045, 1030, 1004, 978, 969, 880, 852, 841, 815, 773, 751 cm^{-1}. HRMS Calcd for $C_{19}H_{18}FO_2$: [M+H]$^+$, 297.1285. Found: m/z 297.1279.

*Procedure for preparation of **1i–1k**, **1m**, and **1n***

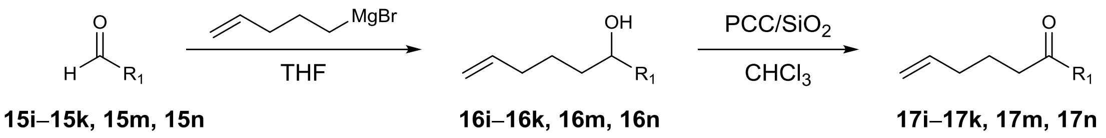

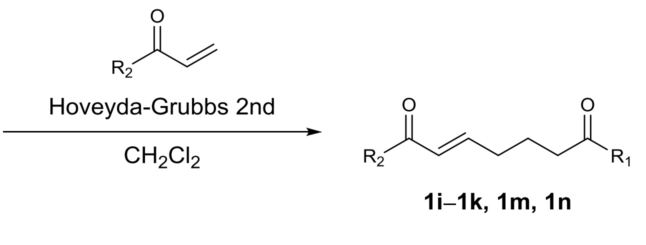

<u>General procedure for preparation of **16**</u>

For preparation of the Grignard reagent, under argon atmosphere, 4-bromo-1-butene (5.3 mL, 45 mmol) was added dropwise to a suspention of Mg (1.20 g, 49.5 mmol), THF

(45 mL), and 1,2-dibromoethane (few drops). To the Grignard reagent, THF (30 mL) was added, and subsequently **15** (30 mmol) was added dropwise at 0 °C. After being stirred for 3 h at ambient temperature, the reaction was quenched with saturated aqueous NH_4Cl, and the aqueous layers were extracted with EtOAc (× 3). The combined organic layers were washed with brine, dried over Na_2SO_4, and concentrated in vacuo to afford the corresponding **16**: **16i**–**16k**, and **16m** were used for the next step without further purification; **16n** was purified by flash silica gel column chromatography using hexane/EtOAc (v/v = 5/1) as an eluent (21%).

General procedure for preparation of **17**

To a round-bottom flask were added sequentially **16** (1 equiv), CH_2Cl_2 (0.26 M), and PCC (3.0 equiv)/SiO_2. After being stirred for 9 h at ambient temperature, the crude reaction mixture was filtered through a Celite pad, and the Celite was washed with CH_2Cl_2. The filtrate was concentrated in vacuo. Purification by flash silica gel column chromatography using hexane/EtOAc (v/v = 20/1–10/1) for **17i**–**17k** and penatne/Et_2O (v/v = 30/1) for **17m** as an eluent gave **17** (**17i**: 41%; **17j**: 53%; **17k**: 68%; **17m**: 84% for 2 steps); **17n** was used for the next step without further purification.

General procedure for preparation of **1i**–**1k**, **1m**, and **1n**

Under argon atmosphere, to a round-bottom flask were added sequentially a vinyl ketone (3.0 equiv), CH_2Cl_2 (0.30 M), **17** (1.0 equiv), Hoveyda–Grubbs 2nd generation catalyst (3.0 mol %). After the reaction mixture was stirred for 11 h at ambient temperature, DMSO (few drops) was added, and the resulting mixture was further stirred for a while. The reaction mixture was subsequently diluted with hexane/EtOAc (v/v = 1/1), passed through a short silica gel pad to remove catalyst, and concentrated in vacuo. Purification by flash silica gel column chromatography using hexane/EtOAc (v/v = 10/1–3/1) as an eluent gave **1i**–**1k**, **1m**, and **1n**.

(*E*)-7-(Naphthalen-1-yl)-1-phenylhept-2-ene-1,7-dione (1i).

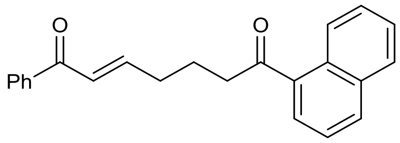

Yellow solid; 33% yield (for the last step).

^{1}H NMR ($CDCl_3$) δ 8.58 (dd, *J* = 8.5, 1.0 Hz, 1H), 7.99 (d, *J* = 8.5 Hz, 1H), 7.92–7.85 (m, 4H), 7.61–7.53 (m, 3H), 7.51–7.44 (m, 3H), 7.09 (dt, *J* = 15.5, 7.0 Hz, 1H), 6.92 (dt, *J* = 15.5, 1.5 Hz, 1H), 3.13 (t, *J* = 7.0 Hz, 2H), 2.47 (m, 2H), 2.07 (m, 2H). ^{13}C NMR

($CDCl_3$) δ 203.9, 190.6, 148.6, 137.8, 135.9, 133.9, 132.7, 132.6, 130.0, 128.5 (2C), 128.4, 127.9, 127.4, 126.48, 126.47, 125.6, 124.3, 41.1, 32.1, 22.9. Mp. 63.6–64.1 °C. TLC: R_f 0.26 (hexane/EtOAc = 5:1). IR (KBr): 3048, 2936, 2844, 2367, 1674, 1666, 1614, 1592, 1577, 1507, 1462, 1449, 1439, 1419, 1396, 1361, 1291, 1279, 1237, 1227, 1214, 1204, 1182, 1173, 1108, 1079, 1045, 1015, 981, 974, 953, 926, 882, 870, 858, 804 cm^{-1}. HRMS Calcd for $C_{23}H_{21}O_2$: $[M+H]^+$, 329.1536. Found: *m*/*z* 329.1529.

(*E*)-1-Phenyl-7-(thiophen-2-yl)hept-2-ene-1,7-dione (1j).

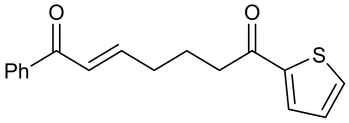

White solid; 59% yield (for the last step).

^{1}H NMR ($CDCl_3$) δ 7.92 (m, 2H), 7.71 (dd, *J* = 4.0, 1.0 Hz, 1H), 7.64 (dd, *J* = 5.0, 1.0 Hz, 1H), 7.56 (tt, *J* = 7.0, 3.5 Hz, 1H), 7.47 (m, 2H), 7.13 (dd, *J* = 5.0, 4.0 Hz, 1H), 7.07 (dt, *J* = 15.5, 7.0 Hz, 1H), 6.92 (dt, *J* = 15.5, 1.5 Hz, 1H), 2.98 (t, *J* = 7.5 Hz, 2H), 2.44 (m, 2H), 2.01 (tt, *J* = 7.0, 7.0 Hz, 2H). ^{13}C NMR ($CDCl_3$) δ 192.5, 190.7, 148.5, 144.2, 137.8, 133.6, 132.7, 131.8, 128.5 (2C), 128.1, 126.5, 38.2, 32.0, 22.8. Mp. 41.7–42.2 °C. TLC: R_f 0.17 (hexane/EtOAc = 5:1). IR (KBr): 3103, 2944, 2362, 1654, 1614, 1595, 1418, 1357, 1256, 1195, 971, 932, 766, 731 cm^{-1}. HRMS Calcd for $C_{17}H_{16}O_2SNa$: $[M+Na]^+$, 307.0763. Found: *m*/*z* 307.0756.

(*E*)-8,8-Dimethyl-1-phenylnon-2-ene-1,7-dione (1k).

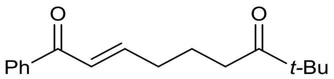

Pale yellow oil; 55% yield (for the last step).

^{1}H NMR ($CDCl_3$) δ 7.92 (m, 2H), 7.56 (m, 1H), 7.47 (m, 2H), 7.04 (dt, *J* = 15.5, 7.0 Hz, 1H), 6.90 (dt, *J* = 15.5, 1.5 Hz, 1H), 2.55 (t, *J* = 7.0 Hz, 2H), 2.32 (m, 2H), 1.81 (m, 2H), 1.14 (s, 9H). ^{13}C NMR ($CDCl_3$) δ 215.4, 190.7, 149.0, 137.8, 132.7, 128.5 (2C), 126.2, 44.1, 35.5, 32.0, 26.4, 22.2. TLC: R_f 0.18 (hexane/EtOAc = 10:1). IR (neat): 2968, 2871, 1704, 1670, 1621, 1598, 1579, 1478, 1449, 1395, 1367, 1350, 1289, 1228, 1180, 1100, 1075, 1017, 994, 976, 879, 849, 825, 768, 694, 665 cm^{-1}. HRMS Calcd for $C_{17}H_{23}O_2$: $[M+H]^+$, 259.1693. Found: *m*/*z* 259.1689.

(*E*)-1-Phenyldodec-2-ene-1,7-dione (1m).

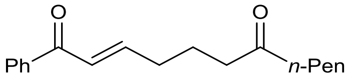

Pale yellow oil; 56% yield (for the last step).

^{1}H NMR ($CDCl_3$) δ 7.92 (m, 2H), 7.56 (m, 1H), 7.47 (m, 2H), 7.02 (dt, *J* = 15.0, 7.0 Hz, 1H), 6.89 (dt, *J* = 15.0, 1.5 Hz, 1H), 2.47 (t, *J* = 7.0 Hz, 2H), 2.39 (t, *J* = 7.5 Hz, 2H), 2.33 (m, 2H), 1.82 (m, 2H), 1.56 (m, 2H), 1.34–1.21 (m, 4H), 0.88 (t, *J* = 7.0 Hz, 3H). ^{13}C NMR ($CDCl_3$) δ 210.7, 190.7, 148.7, 137.8, 132.7, 128.51, 128.50, 126.3, 42.9, 41.6, 32.0, 31.4, 23.5, 22.4, 22.0, 13.9. TLC: R_f 0.14 (hexane/EtOAc = 10:1). IR (neat): 3060, 2949, 2362, 1709, 1665, 1614, 1597, 1578, 1462, 1448, 1418, 1379, 1355, 1313, 1289, 1247, 1236, 1219, 1180, 1156, 1126, 1091, 1039, 1024, 1006, 985, 968, 875, 850, 830, 778, 755, 733, 701 cm^{-1}. HRMS Calcd for $C_{18}H_{25}O_2$: $[M+H]^+$, 273.1849. Found: *m*/*z* 273.1843.

(*E*)-1-Phenyloct-2-ene-1,7-dione (1n): CAS RN [132559-69-4].

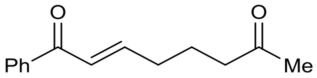

Pale brown solid; 35% yield (for the last step).

^{1}H NMR ($CDCl_3$) δ 7.93 (m, 2H), 7.56 (m, 1H), 7.47 (m, 2H), 7.02 (dt, *J* = 15.5, 6.5 Hz, 1H), 6.90 (dt, *J* = 15.5, 1.5 Hz, 1H), 2.50 (t, *J* = 7.0 Hz, 2H), 2.34 (m, 2H), 2.15 (s, 3H), 1.82 (m, 2H). ^{13}C NMR ($CDCl_3$) δ 208.2, 190.7, 148.6, 137.8, 132.7, 128.53, 128.51, 126.4, 42.6, 31.9, 30.0, 22.0.

*Procedure for preparation of **1g***

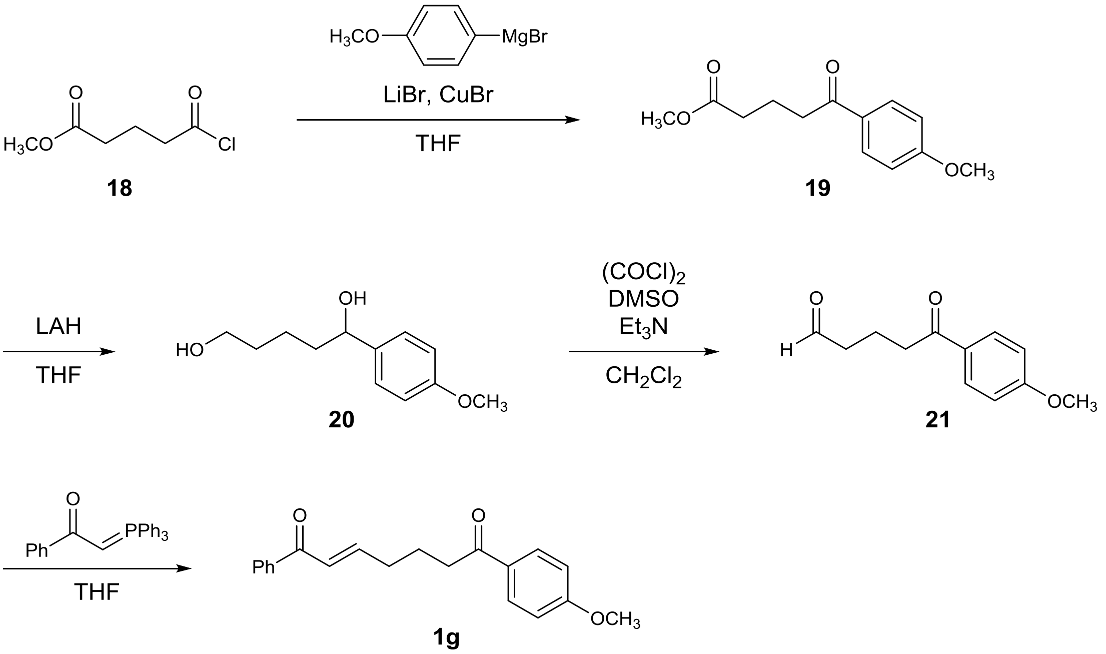

Procedure for preparation of **19**

For preparation of the Grignard reagent, under argon atmosphere, 1-bromo-4-methoxybenzene (1.5 mL, 12 mmol) was added dropwise to a suspention of Mg (0.35 g, 14.4 mmol), THF (12 mL), and 1,2-dibromoethane (few drops). Under argon atmosphere, to a suspention of LiBr (2.08 g, 24 mmol) and CuBr (1.72 g, 12 mmol) in THF (24 mL), the Gregnard reagent and **18** (1.4 mL, 10 mmol) were added dropwise at 0 °C. The reaction mixture was allowed to warm to ambient temperature and stirred for 8 h. Subsequently, the reaction was quenched with saturated aqueous NH_4Cl, and the aqueous layers were extracted with EtOAc (× 2). The combined organic layers were washed with brine (× 2), dried over Na_2SO_4, and concentrated in vacuo to afford **19**, which was used for the next step without further purification.

Procedure for preparation of **20**

Under argon atmosphere, **19** (10 mmol) in THF (15 mL) was added dropwise to a suspention of $LiAlH_4$ (2.9 g, 78 mmol) in THF (60 mL) at 0 °C. The mixture was allowed to warm to ambient temperature and stirred for 8 h. Next, H_2O (2.96 mL), 15% aqueous NaOH (2.96 mL), H_2O (8.88 mL) were sequentially added dropwise at 0 °C, and the mixture was sttired for a while at ambient temperature. Subsequently, the reaction mixture was filtered through a Celite pad, and the Celite pad was washed with Et_2O. The filtrate was concentrated in vacuo to afford the corresponding diols **20**, which was used

for the next step without further purification.

Procedure for preparation of **21**

Under argon atmosphere, to a solution of $(COCl)_2$ (2.6 mL, 30.5 mmol) in CH_2Cl_2 (160 mL), DMSO (2.2 mL, 41.0 mmol) was added dropwise at –78 °C, and the resulting mixture was stirred for 20 min at the same temperature. Next, a solution of **20** (10 mmol) in CH_2Cl_2 (20 mL) was added dropwise, and the mixture was stirred for 40 min at –78 °C. Subsequently, Et_3N (10.9 mL, 81.5 mmol) was added dropwise, and the mixture was stirred for 20 min at the same temperature. The reaction mixture was allowed to warm to ambient temperature and stirred for a while. Subsequently, the reaction was quenched with H_2O, and the aqueous layers were extracted with CH_2Cl_2 (× 2). The combined organic layers were washed with brine, dried over Na_2SO_4, and concentrated in vacuo. Purification by flash silica gel column chromatography using hexane/EtOAc (v/v = 3/1) as an eluent gave **21**.

Procedure for preparation of **1g**

21 (1.0 equiv) and 1-phenyl-2-(triphenyl-λ^5-phosphanylidene)ethan-1-one (3.0 equiv) were dissolved in THF (0.67 M), and the solution was refluxed in an oil bath maintained at 90 °C for 8.5 h. After the solution was cooled to ambient temperature, solvents were removed in vacuo. The residue was diluted with hexane/EtOAc (v/v = 3/1), and passed through Na_2SO_4 to remove phosphine oxide. Purification by flash silica gel column chromatography using hexane/EtOAc (v/v = 3/1) as an eluent gave **1g**.

(*E*)-7-(4-Methoxyphenyl)-1-phenylhept-2-ene-1,7-dione (1g).

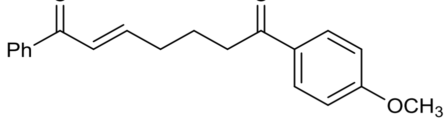

White solid; 45% yield (for the last step).

^{1}H NMR ($CDCl_3$) δ 7.96–7.90 (m, 4H), 7.55 (m, 1H), 7.46 (m, 2H), 7.12 (dt, J = 15.5, 7.0 Hz, 1H), 6.94–6.90 (m, 3H), 3.86 (s, 3H), 2.99 (t, J = 7.0 Hz, 2H), 2.43 (m, 2H), 1.99 (m, 2H). ^{13}C NMR ($CDCl_3$) δ 198.1, 190.7, 163.4, 148.8, 137.8, 132.7, 130.2, 129.9, 128.51, 128.50, 126.4, 113.7, 55.4, 37.1, 32.1, 22.7. Mp. 44.9–45.4 °C. TLC: R_f 0.33 (hexane/EtOAc = 3:1). IR (KBr): 3017, 2944, 2891, 2360, 1670, 1617, 1596, 1572, 1507, 1457, 1449, 1420, 1371, 1350, 1313, 1256, 1199, 1171, 1111, 1022, 1004, 977, 964, 942, 870, 844, 824, 810, 769, 742, 685 cm^{-1}. HRMS Calcd for $C_{20}H_{21}O_3$: $[M+H]^+$, 309.1485. Found: m/z 309.1478.

Procedure for preparation of ***1l***

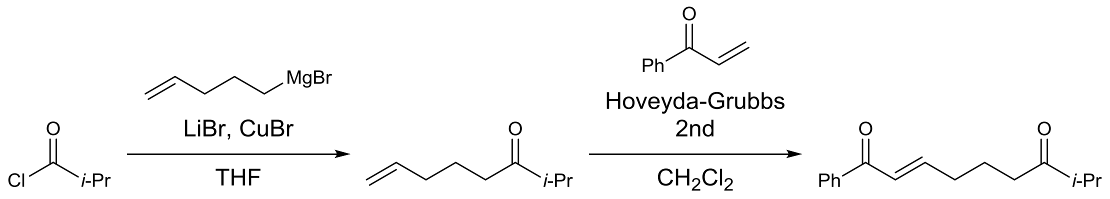

22 **23** **1l**

Procedure for preparation of **23**

For preparation of the Grignard reagent, under argon atmosphere, 4-bromo-1-butene (0.71 mL, 6.0 mmol) was added dropwise to a suspention of Mg (0.18 g, 7.2 mmol), THF (6.0 mL), and 1,2-dibromoethane (few drops). Under argon atmosphere, to a suspention of LiBr (1.04 g, 12 mmol) and CuBr (0.86 g, 6.0 mmol) in THF (12 mL), the Gregnard reagent and **22** (0.53 mL, 5.0 mmol) were added dropwise at 0 °C. The reaction mixture was allowed to warm to ambient temperature and stirred for 2 h. Subsequently, the reaction was quenched with saturated aqueous NH_4Cl, and the aqueous layers were extracted with EtOAc (× 2). The combined organic layers were washed with brine (× 2), dried over Na_2SO_4, and concentrated in vacuo to afford **23**, which was used for the next step without further purification.

Procedure for preparation of **1l**.

Under argon atmosphere, to a round-bottom flask were added sequentially phenyl vinyl ketone (1.82 g, 13.8 mmol), CH_2Cl_2 (14 mL), **23** (0.64 g, 4.6 mmol), Hoveyda–Grubbs 2nd generation catalyst (86.5 mg, 0.14 mmol). After the reaction mixture was stirred for 10 h at ambient temperature, DMSO (few drops) was added, and the resulting mixture was further stirred for a while. The reaction mixture was subsequently diluted with hexane/EtOAc (v/v = 1/1), passed through a short silica gel pad to remove catalyst, and concentrated in vacuo. Purification by flash silica gel column chromatography using hexane/EtOAc (v/v = 10/1) as an eluent gave **1l**.

(*E*)-8-Methyl-1-phenylnon-2-ene-1,7-dione (1l).

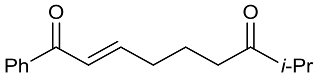

Pale yellow oil; 59% yield (for the last step).
^{1}H NMR ($CDCl_3$) δ 7.92 (m, 2H), 7.56 (tt, *J* = 7.5, 3.0 Hz, 1H), 7.47 (m, 2H), 7.03 (dt, *J* = 15.5, 7.0 Hz, 1H), 6.90 (dt, *J* = 15.5, 1.5 Hz, 1H), 2.59 (m, 1H), 2.52 (t, *J* = 7.0 Hz, 2H), 2.33 (m, 2H), 1.82 (tt, *J* = 7.5, 7.5 Hz, 2H), 1.09 (d, *J* = 7.0 Hz, 6H). ^{13}C NMR ($CDCl_3$) δ 214.1, 190.7, 148.8, 137.8, 132.7, 128.5 (2C), 126.3, 40.9, 39.2, 32.0, 22.0, 18.2. TLC: R_f 0.16 (hexane/EtOAc = 10/1). IR (neat): 2969, 1711, 1669, 1621, 1448, 1353, 1287, 1227, 1088, 1022, 977, 768, 695 cm^{-1}. HRMS Calcd for $C_{16}H_{20}O_2Na$: [M+Na]$^+$, 267.1356. Found: *m*/*z* 267.1349.

Procedure for preparation of ***28***

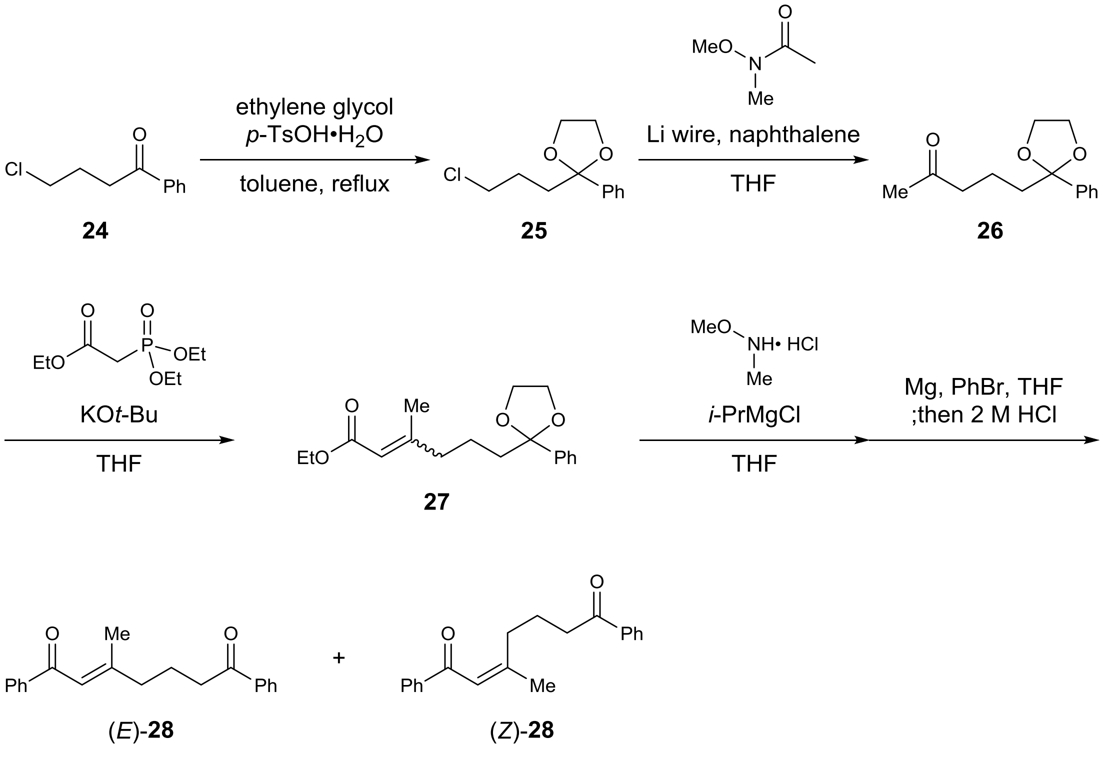

<u>Procedure for preparation of **25**</u>

To a round-bottom flask equipped with Dean–Stark apparatus were added sequentially 4-chlorobutyrophenone (4.3 g, 24 mmol), ethylene glycol (3.9 mL, 71 mmol), *p*-toluenesulfonic acid monohydrate (447 mg, 2.4 mmol), and toluene (200 mL), and the mixture was stirred for 11 h under reflux conditions. The reaction solution was cooled

to ambient temperature, washed with 5% aqueous $NaHCO_3$ (200 mL) and water (200 mL), dried over Na_2SO_4, and concentrated in vacuo. Purification by silica gel column chromatography using hexane/EtOAc (v/v = 10/1) as an eluent gave **25** as a white solid in 88% yield: CAS RN[3308-98-3]. ^{1}H NMR ($CDCl_3$) δ 7.47–7.45 (m, 2H), 7.37–7.34 (m, 2H), 7.31 (m, 1H), 4.02 (m, 2H), 3.78 (m, 2H), 3.54 (t, *J* = 6.5 Hz, 2H), 2.04 (m, 2H), 1.87 (m, 2H). ^{13}C NMR ($CDCl_3$) δ 142.2, 128.1, 127.9, 125.5, 109.8, 64.4, 45.1, 37.6, 26.9.

<u>Procedure for preparation of **26**</u>

To a dry, air free round-bottom flask were added sequentially lithium wire (ca. 0.5% Na, 347 mg, 50 mmol) and naphthalene (1.5 g, 12 mmol). Dry THF (17 mL) was added, and the mixture was stirred at ambient temperature for 2 h (notable color change to dark green) before being cooled to –78 °C. The solution of **25** (2.5 g, 1.1 mmol) in dry THF (3.0 mL) was added dropwise, and the mixture was stirred for additional 3 h at –78 °C. *N*-Methoxy-*N*-methylacetamide (1.0 g, 10 mmol) was added dropwise, and the mixture was allowed to slowly warm to ambient temperature over 4 h and stirred for additional 21 h. The reaction was quenched with saturated aqueous NH_4Cl, and the mixture was stirred for additional 1 h. The reaction solution was the extracted with Et_2O (× 3), washed with brine, dried over Na_2SO_4, and concentrated in vacuo. Purification by silica gel column chromatography using hexane/EtOAc (v/v = 5/1) as an eluent gave **26** as a colorless oil in 42% yield: CAS RN[381295-35-8]. ^{1}H NMR ($CDCl_3$): δ 7.44–7.43 (m, 2H), 7.35–7.32 (m, 2H), 7.28 (m, 1H), 4.02–3.99 (m, 2H), 3.77–3.74 (m, 2H), 2.41 (t, *J* = 7.5 Hz, 2H), 2.09 (s, 3H), 1.88 (t, *J* = 7.5 Hz, 2H), 1.64 (m, 2H). ^{13}C NMR ($CDCl_3$) δ 208.9, 142.3, 128.1, 127.8, 125.6, 110.1, 64.4, 43.5, 39.5, 29.8, 17.9.

<u>Procedure for preparation of **27**</u>

To a dry, air free round-bottom flask was added potassium *tert*-butoxide (577 mg, 5.1 mmol). Dry THF (15 mL) was added, and the suspension was cooled to 0 °C. Neat diethylphosphonoacetic acid ethyl ester (1.1 mL, 5.4 mmol) was added dropwise, and the mixture was stirred for 0.5 h. The resulting suspension was heated to 50 °C to form clear solution and stirred for additional 12 h. The reaction solution was concentrated in vacuo, and Et_2O (20 mL) was added. The organic layers were washed with saturated aqueous NH_4Cl, H_2O, and brine, respectively, dried over Na_2SO_4, and concentrated in vacuo. Purification by silica gel column chromatography using hexane/EtOAc (v/v = 5/1) as an eluent gave **27**.

Ethyl 3-methyl-6-(2-phenyl-1,3-dioxolan-2-yl)hex-2-enoate (27).

(*E*)- and (*Z*)-isomers could not be separated.

Yield: 71%, *E*:*Z* = 2.6:1, colorless oil. TLC: R_f 0.35 (hexane/EtOAc = 5/1). IR (neat): 2951, 2883, 1714, 1646, 1448, 1224, 1144, 1040, 937, 703 cm^{-1}. HRMS Calcd for $C_{18}H_{25}O_4$: [M+H]$^+$, 305.1747. Found: *m*/*z* 305.1740.

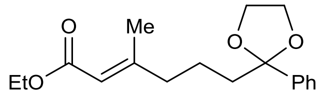

(*E*)-27: ^{1}H NMR ($CDCl_3$): δ 7.44–7.43 (m, 2H), 7.36–7.26 (m, 3H), 5.60 (s, 1H), 4.12 (q, *J* = 7.0 Hz, 2H), 4.01 (t, *J* = 6.5 Hz, 2H), 3.76 (t, *J* = 6.5 Hz, 2H), 2.10 (m, 2H), 2.09 (s, 3H), 1.87 (t, *J* = 8.0 Hz, 2H), 1.57–1.49 (m, 2H), 1.26 (t, *J* = 7.0 Hz, 3H). ^{13}C NMR ($CDCl_3$): δ 166.8, 159.7, 142.3, 128.1, 127.8, 125.6, 115.7, 110.1, 64.5, 59.4, 40.7, 39.8, 21.3, 18.5, 14.30.

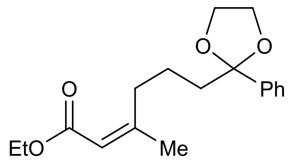

(*Z*)-27: ^{1}H NMR ($CDCl_3$): δ 7.44–7.43 (m, 2H), 7.36–7.26 (m, 3H), 5.62 (s, 1H), 4.12 (q, *J* = 7.0 Hz, 2H), 4.01 (t, *J* = 6.5 Hz, 2H), 3.76 (t, *J* = 6.5 Hz, 2H), 2.60 (t, *J* = 8.0 Hz, 2H), 1.92 (t, *J* = 8.0 Hz, 2H), 1.81 (s, 3H), 1.57–1.49 (m, 2H), 1.23 (t, *J* = 7.0 Hz, 3H). ^{13}C NMR ($CDCl_3$): δ 166.3, 160.0, 142.5, 128.1, 127.8, 125.7, 116.4, 110.3, 64.5, 59.4, 40.0, 32.8, 24.9, 22.0, 14.26.

Procedure for preparation of **28**

To a dry, air free round-bottom flask were added sequentially **27** (780 mg, 2.7 mmol), *N*,*O*-dimethyl hydroxylamine hydrochloride (524 mg, 5.4 mmol), and dry THF (5.5 mL), and the mixture was stirred for 0.5 h before being cooled to –15 °C. A solution of *i*-PrMgCl (1.3 M in THF, 8.6 mL, 11 mmol) was added dropwise over 15 min, and the mixture was allowed to warm to ambient temperature. After being stirred for 2 h, the reaction was quenched with saturated aqueous NH_4Cl, and the mixture was extracted with Et_2O. The combined organic layers were washed with brine, dried over Na_2SO_4, and concentrated in vacuo. Purification by silica gel column chromatography using hexane/EtOAc (v/v = 3/1) as an eluent gave the corresponding Weinreb amide as a colorless oil, which was used for the next step as a mixture of two isomers.

To a dry, air free round-bottom flask were added sequentially magnesium turnings (215 mg, 2.2 mmol) and dry THF (6.3 mL). Neat bromobenzene (467 μL, 4.4 mmol) and 1,2-dibromoethane (few drops) were added slowly to the stirring magnesium, and the

mixture was stirred at ambient temperature for 0.5 h. The resulting solution of phenylmagnesium bromide in THF was transferred slowly to a dry, air free round-bottom flask charged with a solution of prepared Weinreb amide (710 mg, 2.2 mmol) in THF (3.7 mL) at 0 °C, and the mixture was stirred for 2 h. 2 M Aqueous HCl (10 mL) was added to the solution at 0 °C, and the resulting mixture was stirred overnight. The solution was extracted with Et_2O, and the combined organic layers were washed with brine, dried over Na_2SO_4, and concentrated in vacuo. Purification by silica gel column chromatography using hexane/EtOAc (v/v = 10/1) as an eluent gave (*E*)-**28** and (*Z*)-**28**, respectively.

(*E*)-3-Methyl-1,7-diphenylhept-2-ene-1,7-dione ((*E*)-28).

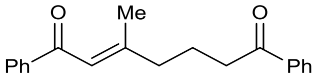

Colorless oil; 43% yield (for 2 steps from **27**).
^{1}H NMR ($CDCl_3$) δ 7.97 (d, *J* = 8.5 Hz, 2H), 7.90 (d, *J* = 8.5 Hz, 2H), 7.57 (dt, *J* = 7.5, 1.0 Hz, 1H), 7.52 (dt, *J* = 7.5, 1.0 Hz, 1H), 7.48–7.41 (m, 4H), 6.76 (s, 1H), 3.04 (t, *J* = 7.5 Hz, 2H), 2.37 (t, *J* = 7.5 Hz, 2H), 2.22 (s, 3H), 2.05 (m, 2H). ^{13}C NMR ($CDCl_3$) δ 199.6, 191.6, 159.1, 139.1, 136.8, 133.1, 132.3, 128.6, 128.4, 128.2, 128.0, 121.1, 40.6, 37.4, 21.7, 19.7. TLC: R_f 0.18 (hexane/EtOAc = 5:1). IR (neat): 3056, 2895, 1679, 1652, 1611, 1448, 1361, 1240, 1180, 1010, 859, 756, 692 cm^{-1}. HRMS Calcd for $C_{20}H_{20}O_2Na$: [M+Na]$^+$, 315.1356. Found: *m*/*z* 315.1348.

(*Z*)-3-Methyl-1,7-diphenylhept-2-ene-1,7-dione ((*Z*)-28).

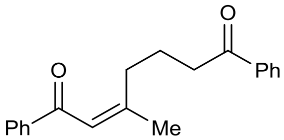

White solid; 20% yield (for 2 steps from **27**).
^{1}H NMR ($CDCl_3$) δ 7.95 (d, *J* = 8.0 Hz, 2H), 7.92 (d, *J* = 8.0 Hz, 2H), 7.55–7.52 (m, 2H), 7.46–7.42 (m, 4H), 6.78 (s, 1H), 3.09 (t, *J* = 8.0 Hz, 2H), 2.73 (t, *J* = 8.0 Hz, 2H), 2.06 (s, 3H), 1.99 (m, 2H). ^{13}C NMR ($CDCl_3$) δ 200.1, 191.2, 159.9, 139.1, 136.9, 132.9, 132.4, 128.5, 128.4, 128.2, 128.0, 121.8, 38.2, 33.4, 25.6, 22.5. Mp. 50.5–51.0 °C. TLC: R_f 0.28 (hexane/EtOAc = 5:1). IR (KBr): 3050, 2969, 2942, 1680, 1659, 1595, 1576, 1448, 1280, 1235, 1201, 1190, 1020, 985, 972, 669, 668 cm^{-1}. HRMS Calcd for $C_{20}H_{20}O_2Na$: [M+Na]$^+$, 315.1356. Found: *m*/*z* 315.1348.

Procedure for preparation of ***32***

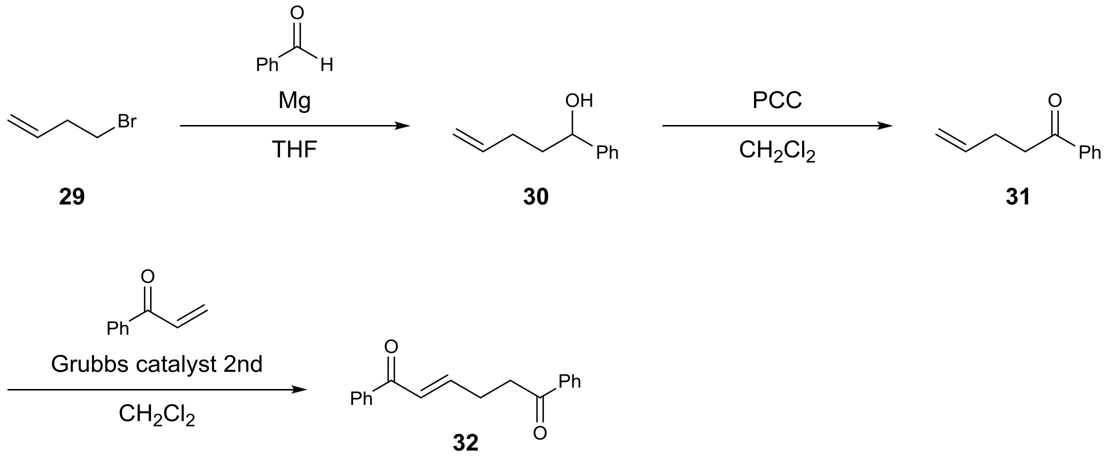

Procedure for preparation of **30**

To a dry, air free round-bottom flask were added sequentially magnesium turnings (401 mg, 16.5 mmol) and dry THF (15 mL). Neat 4-bromobut-1-ene (**29**, 1.5 mL, 15 mmol) and 1,2-dibromoethane (few drops) were added slowly to the stirring magnesium. After the mixture was stirred at ambient temperature for 0.5 h, dry THF (10 mL) was added, and the solution was cooled to 0 °C. Benzaldehyde (1.0 mL, 10 mmol) was added dropwise, and the mixture was allowed to warm to ambient temperature. After being stirred for 1 h, the reaction mixture was quenched with saturated aqueous NH_4Cl, and subsequently extracted with EtOAc. The combined organic layers were washed with brine, dried over Na_2SO_4, and concentrated in vacuo to yield 1-phenylpent-4-en-1-ol (**30**) as a colorless oil, which was used for the next step without further purification: CAS RN [54525-86-9]. ^{1}H NMR ($CDCl_3$) δ 7.38–7.34 (m, 4H), 7.29 (m, 1H), 5.85 (m, 1H), 5.04 (dd, *J* = 15.5, 2.0 Hz, 1H), 4.99 (dd, *J* = 10.0, 2.0 Hz, 1H), 4.71 (m, 1H), 2.12 (m, 2H), 1.92 (m, 2H), 1.88 (s, 1H). ^{13}C NMR ($CDCl_3$) δ 144.6, 138.2, 128.5, 127.6, 125.9, 115.0, 74.0, 38.0, 30.1.

Procedure for preparation of **31**

To a solution of **30** (1.6 g, 10 mmol) in dry CH_2Cl_2 (50 mL) was added pyridinium chlorochromate (11 g, 50 mmol) in one portion, and the mixture was stirred for 4 h at ambient temperature. The reaction solution was passed through Celite pad, and the filtrate was concentrated in vacuo to yield 1-phenylpent-4-en-1-one (**31**) as a colorless oil, which was used for the next step without further purification: CAS RN [3240-29-7]. ^{1}H NMR ($CDCl_3$) δ 7.97 (m, 2H), 7.56 (m, 1H), 7.46 (m, 2H), 5.91 (m, 1H), 5.09 (dd, *J* = 16.5, 1,0 Hz, 1H), 5.02 (dd, *J* = 10.0, 1.0 Hz, 1H), 3.08 (m, 2H), 2.50 (m, 2H). ^{13}C NMR

(CDCl$_3$) δ 199.2, 137.0, 136.6, 132.7, 128.3, 127.7, 115.0, 37.4, 27.8.

Procedure for preparation of **32**

To a solution of **31** (1.6 g, 10 mmol) and 1-phenylprop-2-en-1-one (3.3 g ,25 mmol) in dry CH_2Cl_2 (50 mL) was added Grubbs 2nd generation catalyst (170 mg, 0.20 mmol) at ambient temperature under argon atmosphere. The mixture was stirred overnight and then concentrated in vacuo. Purification of the reaction mixture by flash silica gel column chromatography using hexane/EtOAc (v/v = 5/1) as an eluent gave **32**.

(*E*)-1,6-Diphenylhex-2-ene-1,6-dione (32): CAS RN [24086-83-7]

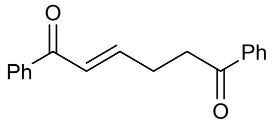

White solid; 8% yield (for 3 steps).

^{1}H NMR (CDCl$_3$) δ 7.99–7.97 (m, 2H), 7.94–7.91 (m, 2H), 7.61–7.55 (m, 2H), 7.54–7.45 (m, 4H), 7.11 (dt, J = 15.5, 7.0 Hz, 1H), 6.97 (dt, J = 15.5, 1.5 Hz, 1H), 3.23 (t, J = 7.0 Hz, 2H), 2.79 (ddt, J = 7.0, 1.5, 7.0 Hz, 2H). ^{13}C NMR (CDCl$_3$) δ 198.3, 190.7, 147.8, 137.7, 136.5, 133.3, 132.7, 128.7, 128.5 (2C), 128.0, 126.7, 36.8, 27.0.

Procedure for preparation of ***34***

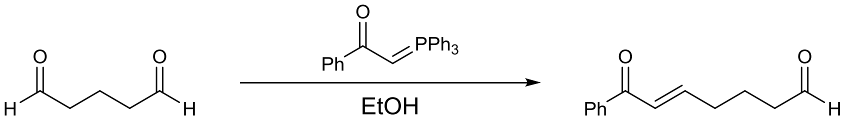

33 **34**

To a solution of glutaraldehyde (**33**, 6.6 g, 66 mmol) in EtOH (25 mL) was added 1-phenyl-2-(triphenyl-λ_5-phosphanylidene)ethan-1-one (1.3 g, 3.3 mmol), and the mixture was stirred at ambient temperature for 11 h. H_2O (60 mL) was added to the solution, and the mixture was extracted with EtOAc. The combined organic layers were washed with 0.2 M aqueous HCl and brine, dried over Na_2SO_4, and concentrated in vacuo. Purification by flash silica gel column chromatography using hexane/Et_2O (v/v = 2/1) as an eluent gave (*E*)-7-oxo-7-phenylhept-5-enal (**34**).

(*E*)-7-Oxo-7-phenylhept-5-enal (34): CAS RN [169892-12-0]

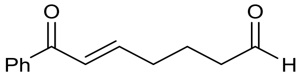

Colorless oil; 40% yield.

^{1}H NMR ($CDCl_3$) δ 9.80 (t, *J* = 1.0 Hz, 1H), 9.94–7.92 (m, 2H), 7.56 (m, 1H), 7,49–7.46 (m, 2H), 7.02 (dt, *J* = 15.5, 7.0 Hz, 1H), 6.91 (dt, *J* = 15.5, 1.0 Hz, 1H), 2.54 (dt, *J* = 1.0, 7.0 Hz, 2H), 2.37 (ddt, *J* = 7.0, 1.0, 7.0 Hz, 2H), 1.88 (tt, *J* = 7.0, 7.0 Hz, 2H). ^{13}C NMR ($CDCl_3$) δ 201.7, 190.6, 148.0, 137.7, 132.8, 128.55, 128.50, 126.5, 43.0, 31.8, 20.4.

Procedure for preparation of ***37***

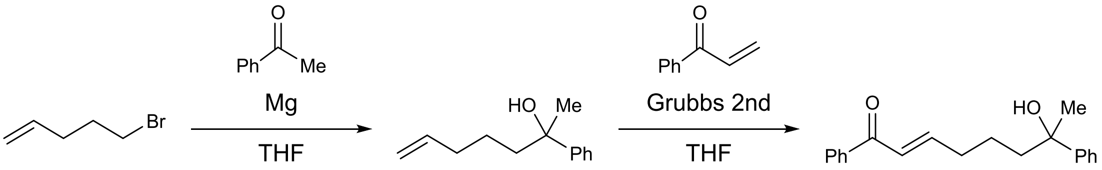

To a dry, air free round-bottom flask were added sequentially magnesium turnings (221 mg, 9.1 mmol) and dry THF (12 mL). Neat 5-bromopent-1-ene (**35**, 0.95 mL, 8.0 mmol) and 1,2-dibromoethane (few drops) were added slowly to the stirring magnesium. After the mixture was stirred at ambient temperature for 0.5 h, dry THF (4.0 mL) was added. Acetophenone (0.47 mL, 4.0 mmol) was added dropwise, and the mixture was stirred at ambient temperature for 6 h. The reaction mixture was quenched with saturated aqueous NH_4Cl and subsequently extracted with EtOAc. The combined organic layers were washed with brine, dried over Na_2SO_4, and concentrated in vacuo to yield 2-phenylhept-6-en-2-ol (**36**) as a colorless oil.

Next, to a solution of **36** (580 mg, 3.0 mmol) and 1-phenylprop-2-en-1-one (1.6 g, 12 mmol) in dry CH_2Cl_2 (6.0 mL) was added Grubbs 2nd generation catalyst (52 mg, 0.061 mmol). After being stirred for 18 h, the reaction mixture was concentrated in vacuo. Purification of the reaction mixture by flash silica gel column chromatography using hexane/EtOAc (v/v = 3/1) as an eluent gave (*E*)-7-hydroxy-1,7-diphenyloct-2-en-1-one (**37**).

(*E*)-7-Hydroxy-1,7-diphenyloct-2-en-1-one (37): CAS RN [2087909-59-7]

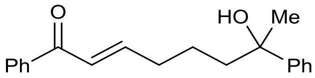

White solid; 18% yield (for 2 steps).

^{1}H NMR (C_6D_6) δ 7.88 (m, 2H), 7.35 (m, 2H), 7.20 (m, 2H), 7.14–7.00 (m, 5H), 6.64 (dt, *J* = 15.5, 1.5 Hz, 1H), 1.84 (m, 2H), 1.64–1.49 (m, 2H), 1.36 (m, 1H), 1.28 (s, 3H), 1.25–1.13 (m, 2H). ^{13}C NMR (C_6D_6) δ 189.5, 149.0, 148.4, 138.6, 132.4, 128.8, 128.6, 128.4, 126.7, 126.0, 125.2, 74.0, 44.0, 33.0, 30.7, 22.9.

Supplementary Discussion

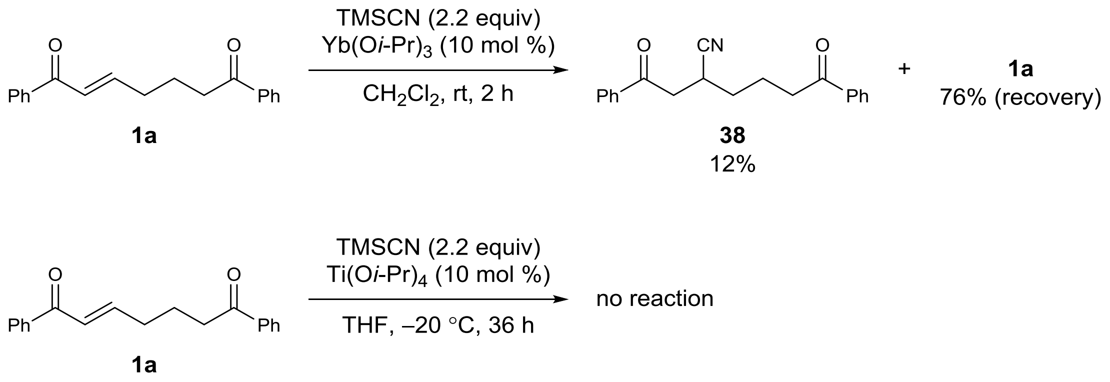

Supplementary Figure 1. Reactions with Yb(O*i*-Pr)$_3$ or Ti(O*i*-Pr)$_4$ (Shibasaki's protocol[3]).

The cyanosilylation protocols corresponding to the method reported by Shibasaki group[3] were applied to our substrate. Under the conditions with Yb(O*i*-Pr)$_3$ as a catalyst, only the conjugate addition of the cyanide to the enone proceeded in 12% yield. Additionally, under the conditions with Ti(O*i*-Pr)$_4$ as a catalyst, no reaction took place. Thus, we consider our protocol is essential for the described reactions to proceed.

Procedure

To the solution of Yb(O*i*-Pr)$_3$ or Ti(O*i*-Pr)$_4$ (0.015 mmol) in solvent (0.30 mL) was added trimethylsilylcyanide (3.0 μL, 0.030 mmol) in an ice bath, and the mixture was stirred at ambient temperature for 30 min. To the solution were added **1a** (41.8 mg, 0.15 mmol) followed by additional trimethylsilylcyanide (30 μL, 0.30 mmol) at ambient temperature or –20 °C. After the mixture was stirred for hours, H_2O (0.20 mL) was added. The reaction mixture was subsequently diluted with hexane/EtOAc (v/v = 1/1), passed through a short silica gel pad to remove the catalyst, and concentrated in vacuo. In the case of the reaction using Yb(O*i*-Pr)$_3$, purification of the crude product by flash silica gel column chromatography using hexane/EtOAc (v/v = 3/1) was carried out.

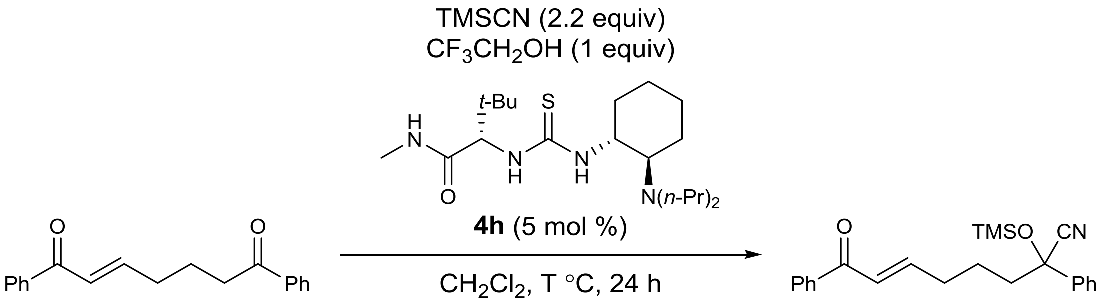

1a **39**

entry	T (°C)	yield (%)[b]	ee (%)
1	–78	27	98
2	25	16	60

Supplementary Figure 2. Reactions with catalyst **4h** (Jacobsen's protocol[4]). Reactions were run using **1a** (0.15 mmol), TMSCN (0.33 mmol), CF_3CH_2OH (0.15 mmol), **4h** (0.015 mmol), and CH_2Cl_2 (0.30 mL). Yields represent material isolated after silica gel column chromatography.

The cyanosilylation protocols corresponding to the method reported by Jacobsen group[4] were applied to our substrate. Under the conditions with the Jacobsen's chiral thiourea catalyst, only the 1,2-addition of the cyanide affording **39** proceeded with moderate to good enantioselectivities. Thus, we consider our protocol is essential for the described reactions to proceed.

Procedure

To a 5-mL round bottom flask were sequentially added **4h** (2.89 mg, 0.0075 mmol), **1a** (41.8 mg, 0.15 mmol), trimethylsilylcyanide (41 μL, 0.33 mmol) and CH_2Cl_2 (0.30 mL). The reaction mixture was stirred at –78 °C or 25 °C for 15 min. 2,2,2-Trifluoroethan-1-ol (11 μL, 0.15 mmol) was then added, and the mixture was stirred at the same temperature for 24 h. After being warmed to ambient temperature, the reaction mixture was subsequently diluted with hexane/EtOAc (v/v = 1/1), passed through a short silica gel pad to remove **4h**, and concentrated in vacuo. Purification of the crude product by flash silica gel column chromatography using hexane/EtOAc (v/v = 5/1) as an eluent afforded **39**.

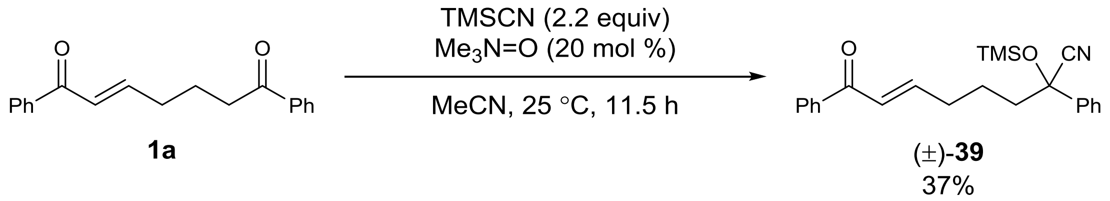

Supplementary Figure 3. Synthesis of racemic **39** for HPLC analysis.

Procedure

To a solution of **1a** (83.6 mg, 0.30 mmol) in CH_3CN (0.30 mL) were added trimethylamine *N*-oxide (4.51 mg, 0.060 mmol) and trimethylsilylcyanide (56 μL, 0.45 mmol) at 25 °C. The solution was stirred for 11.5 h. The reaction mixture was subsequently diluted with hexane/EtOAc (v/v = 1/1), passed through a short silica gel pad to remove trimethylamine *N*-oxide, and concentrated in vacuo. Purification of the crude product by flash silica gel column chromatography using hexane/EtOAc (v/v = 5/1) as an eluent afforded (±)-**39**.

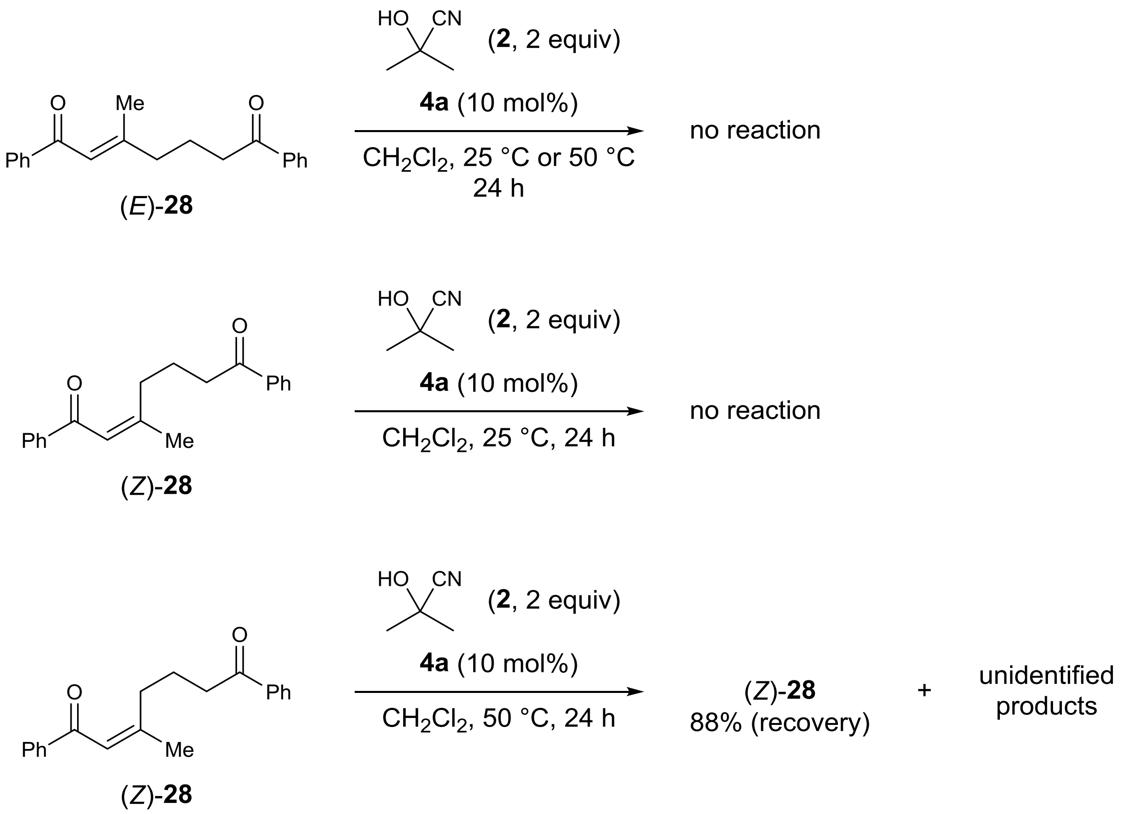

Supplementary Figure 4. Reactions of (*E*)-3-methyl-1,7-diphenylhept-2-ene-1,7-dione ((*E*)-**28**) and (*Z*)-3-methyl-1,7-diphenylhept-2-ene-1,7-dione ((*Z*)-**28**).

The reactions from substrates (*E*)- and (*Z*)-**28** bearing trisubstituted olefins were carried out, however, the reactions failed to yield cyclic products at 25 °C or 50 °C. Further optimization for the use of such substrates is currently underway.

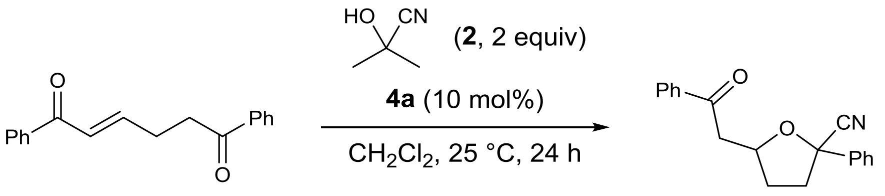

32

40
99%
1.4:1 dr
76% *ee* (44% *ee*)

Supplementary Figure 5. Reaction of (*E*)-1,6-diphenylhex-2-ene-1,6-dione (**32**).

The synthesis of a tetrahydrofuran (THF) derivative **40** was investigated, however, under the present conditions, the stereoselectivities were not as good as those obtained from the reactions affording the tetrahydropyran (THP) derivatives **3**. Although we believe the anomeric effect also assists with obtaining the excellent diastereoselectivities, the small steric effect of the cyano group, which weakens 1,3-diaxial interactions in the six-membered oxacycle, seems to be essential. Individual optimization of bifunctional catalysts is also necessary for obtaining higher enantioselectivity in THF synthesis. Thus, we consider the current protocol is useful for the construction of chiral THPs. Further optimization for the synthesis of THFs is currently underway.

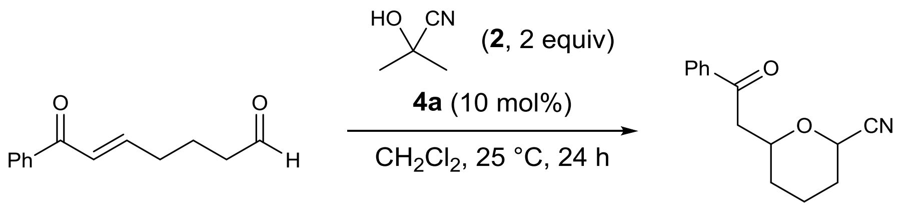

Supplementary Figure 6. Reaction of (*E*)-7-oxo-7-phenylhept-5-enal (**34**).

The reaction from an aldehyde was carried out, however, the diastereomeric ratio (dr) was not as good as that of the reactions from ketones under the present conditions. Although we believe the anomeric effect also assists with obtaining the excellent diastereoselectivities, the small steric effect of the cyano group compared with that of another substituent on the same carbon seems to be essential to favour its axial position. Thus, we consider the current protocol is useful for the construction of a tetrasubstituted chiral center, and supplements the conventional diastereoselective synthesis of less substituted derivatives.[2] Further optimization for the use of aldehyde substrates is currently underway.

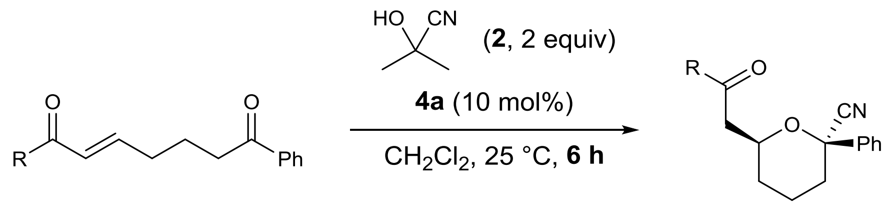

1 **3**

entry	R	yield (%)	dr	ee (%)
1	Ph (**1a**)	86	>20:1	97
2	4-$CH_3OC_6H_4$ (**1b**)	22	>20:1	93

Supplementary Figure 7. Investigations of electronic effects of enone moieties. Reactions were run using **1** (0.15 mmol), **2** (0.30 mmol), **4a** (0.015 mmol), and CH_2Cl_2 (0.30 mL). Yields represent material isolated after silica gel column chromatography.

All reactions shown in Table 2 of the main manuscript were carried out for 24 h. The reactions of **1a** and **1b** were also investigated for a shorter time, 6 h, and the results indicated that there are electronic effects of the enone moieties on the reaction rate.

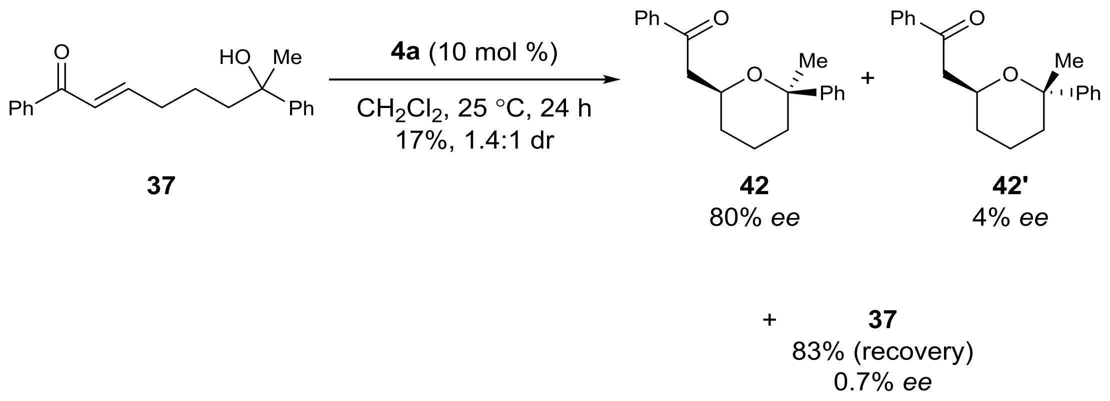

Supplementary Figure 8. Kinetic resolution of **37**.

In order to examine the anomeric effect by the cyano group, the kinetic resolution of a tertiary alcohol **37** bearing a methyl group, which does not show anomeric effects, was investigated; the reaction resulted in significantly low diastereoselectivity with low to moderate enantioselectivities. We consider these results support the importance of the anomeric effect by the cyano group in the control of stereoselectivity on the tetrasubstituted chiral center.

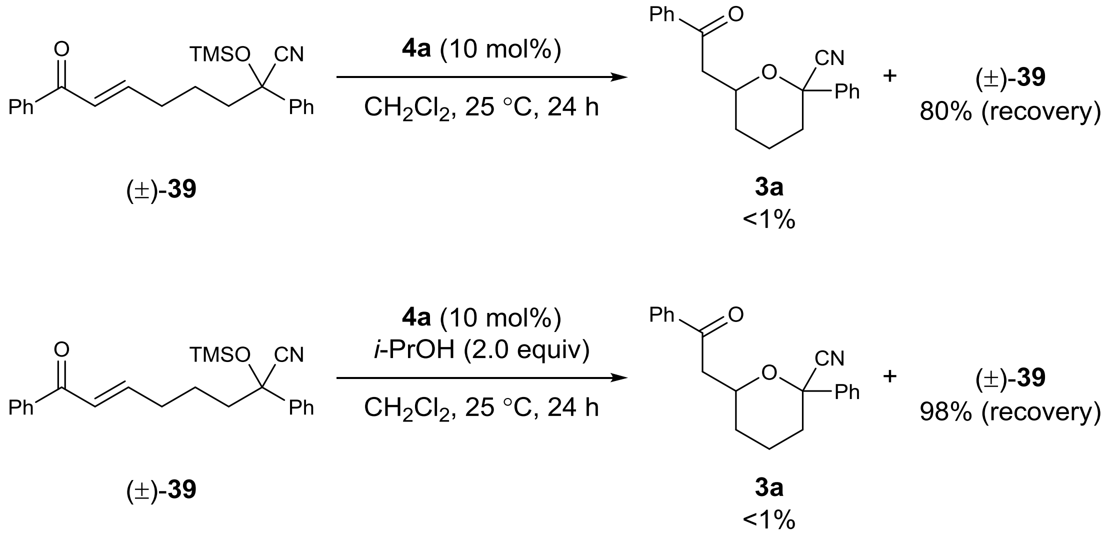

Supplementary Figure 9. Reactions from racemic cyanohydrin silyl ether (±)-**39**.

To gain insight into the reaction mechanism, the reaction from the possible racemic intermediate was investigated. Because it is difficult to isolate the cyanohydrin because of the reversibility of its formation, the reaction using the corresponding raccemic silyl ether was carried out, however, the reaction did not afford the cyclic product. The reaction did not proceed even in the presence of *i*-PrOH. Thus, the reaction mechanism was discussed by referring to the results indicated in Figure 4 of the main manuscript.

Procedure

To a 5-mL vial were sequentially added (±)-**39** (43.3 mg, 0.115 mmol), CH_2Cl_2 (0.23 mL), and **4a** (5.2 mg, 0.0115 mmol) (in the latter case, additionally 2-propanol (2.0 equiv) was added). The mixture was stirred in an oil bath maintained at 25 °C for 24 h. The reaction mixture was subsequently diluted with hexane/EtOAc (v/v = 1/1), passed through a short silica gel pad to remove **4a**, and concentrated in vacuo.

Characterization Data of Products

(2*R*,6*S*)-6-(2-Oxo-2-phenylethyl)-2-phenyltetrahydro-2*H*-pyran-2-carbonitrile (3a).

The diastereomers were further separated by flash silica gel column chromatography using hexane/EtOAc (v/v = 5/1) as an eluent.

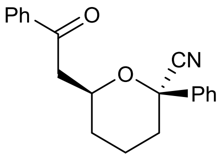

Yield: 99% (33.1 mg), dr = >20:1, 97% *ee*, white solid. $[\alpha]_D^{18}$ +20.2 (*c* 0.43, CH_2Cl_2). ^{1}H NMR ($CDCl_3$) δ 7.99 (m, 2H), 7.58 (tt, *J* = 7.5, 1.5 Hz, 1H), 7.50–7.46 (m, 4H), 7.39–7.32 (m, 3H), 4.66 (m, 1H), 3.42 (dd, *J* = 16.0, 6.0 Hz, 1H), 3.19 (dd, *J* = 16.0, 7.0 Hz, 1H), 2.19 (m, 1H), 2.11 (m, 1H), 2.03–1.96 (m, 2H), 1.75 (ddd, *J* = 13.5, 13.5, 4.0 Hz,1H), 1.44 (m, 1H). ^{13}C NMR ($CDCl_3$) δ 197.2, 139.6, 137.0, 133.2, 128.8, 128.61, 128.60, 128.2, 124.8, 118.6, 76.9, 72.7, 44.7, 37.6, 30.0, 20.9. Mp. 120.7–121.2 °C. TLC: R_f 0.40 (hexane/EtOAc = 3:1). IR (KBr): 3058, 2954, 1678, 1666, 1616, 1597, 1578, 1560, 1458, 1449, 1413, 1358, 1291, 1258, 1223, 1197, 1180, 1007, 982, 966, 932, 872, 841, 772, 754, 736, 697, 689 cm^{-1}. HRMS Calcd for $C_{20}H_{19}NO_2Na$: $[M+Na]^+$, 328.1308. Found: *m*/*z* 328.1299. HPLC (Daicel Chiralpak IE, hexane/*i*-PrOH = 92.5/7.5, flow rate = 1.5 mL/min, λ = 254 nm, 40 °C): t_{minor} = 19.5 min, t_{major} = 15.1 min.

6-(2-(4-Methoxyphenyl)-2-oxoethyl)-2-phenyltetrahydro-2*H*-pyran-2-carbonitrile (3b).

The diastereomers were further separated by flash silica gel column chromatography using hexane/EtOAc (v/v = 3/1) as an eluent.

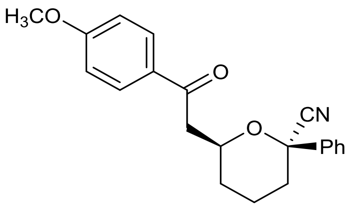

Yield: 99% (53.9 mg), dr = >20:1, 97% *ee*, colorless oil. $[\alpha]_D^{18}$ +11.0 (*c* 0.42, CH_2Cl_2). ^{1}H NMR ($CDCl_3$) δ 7.97 (m, 2H), 7.49 (m, 2H), 7.38–7.34 (m, 3H), 6.94 (m, 2H), 4.63 (m, 1H), 3.87 (s, 3H), 3.36 (dd, *J* = 16.0, 5.0 Hz, 1H), 3.13 (dd, *J* = 16.0, 7.0 Hz, 1H), 2.18 (m, 1H), 2.09 (m, 1H), 2.02–1.95 (m, 2H), 1.74 (ddd, *J* = 13.5, 13.5, 4.0 Hz, 1H), 1.42 (m, 1H). ^{13}C NMR ($CDCl_3$) δ 195.6, 163.6, 139.6, 130.5, 130.1, 128.8, 128.6, 128.2, 124.8, 118.6, 113.7, 76.9, 55.5, 44.4, 37.6, 30.0, 20.9. TLC: R_f 0.33 (hexane/EtOAc = 3:1). IR (neat): 2940, 1676, 1601, 1577, 1511, 1450, 1420, 1309,

1262, 1170, 1099, 1045, 965, 837, 759, 698 cm^{-1}. HRMS Calcd for $C_{21}H_{21}NO_3Na$: $[M+Na]^+$, 358.1414. Found: *m*/*z* 358.1408. HPLC (Daicel Chiralpak IE, hexane/*i*-PrOH = 90.0/10.0, flow rate = 2.0 mL/min, λ = 254 nm, 40 °C): t_{minor} = 23.6 min, t_{major} = 18.6 min.

6-(2-Oxo-2-(4-(trifluoromethyl)phenyl)ethyl)-2-phenyltetrahydro-2*H*-pyran-2-carbonitrile (3c).

The diastereomers were further separated by flash silica gel column chromatography using hexane/EtOAc (v/v = 5/1) as an eluent.

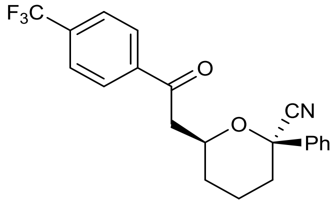

Yield: 89% (49.8 mg), dr = >20:1, 93% *ee*, white solid. $[\alpha]_D^{18}$ +15.1 (*c* 1.05, CH_2Cl_2). ^{1}H NMR ($CDCl_3$) δ 8.08 (dd, *J* = 9.5, 9.0 Hz, 2H), 7.73 (m, 2H), 7.44 (m, 2H), 7.37–7.33 (m, 3H), 4.66 (m, 1H), 3.42 (dd, *J* = 16.0, 6.0 Hz, 1H), 3.20 (dd, *J* = 16.0, 6.0 Hz, 1H), 2.19 (m, 1H), 2.11 (m, 1H), 2.02–1.97 (m, 2H), 1.75 (ddd, *J* = 13.5, 13.5, 4.0 Hz, 1H), 1.46 (m, 1H). ^{13}C NMR ($CDCl_3$) δ 196.3, 139.6, 134.4 (q, *J* = 32.7 Hz), 128.9, 128.6, 125.66 (q, *J* = 3.7 Hz), 125.64, 125.62, 124.7, 123.6 (q, *J* = 272.5 Hz), 118.5, 76.8, 72.6, 44.9, 37.5, 29.9, 20.9. ^{19}F NMR ($CDCl_3$) δ 98.6. Mp. 120.3–120.7 °C. TLC: R_f 0.35 (hexane/EtOAc = 5:1). IR (KBr): 2951, 2924, 1704, 1511, 1492, 1440, 1407, 1334, 1313, 1238, 1217, 1164, 1142, 1097, 1032, 984, 964, 862, 830, 763, 699 cm^{-1}. HRMS Calcd for $C_{21}H_{18}F_3NO_2Na$: $[M+Na]^+$, 396.1182. Found: *m*/*z* 396.1178. HPLC (Daicel Chiralpak IB, hexane/*i*-PrOH = 98.5/1.5, flow rate = 1.5 mL/min, λ = 254 nm, 40 °C): t_{minor} = 14.9 min, t_{major} = 11.2 min.

6-(2-Oxo-2-(thiophen-2-yl)ethyl)-2-phenyltetrahydro-2*H*-pyran-2-carbonitrile (3d).

The diastereomers were further separated by flash silica gel column chromatography using hexane/EtOAc (v/v = 5/1) as an eluent.

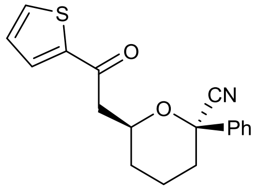

Yield: 99% (50.9 mg), dr = >20:1, 97% *ee*, white solid. $[\alpha]_D^{18}$ +23.0 (*c* 1.22, CH_2Cl_2).

^{1}H NMR ($CDCl_3$) δ 7.75 (dd, J = 4.0, 1.0 Hz, 1H), 7.66 (dd, J = 5.0, 1.0 Hz, 1H), 7.48 (m, 2H), 7.38–7.33 (m, 3H), 7.13 (dd, J = 5.0, 4.0 Hz, 1H), 4.62 (m, 1H), 3.32 (dd, J = 15.5, 5.5 Hz, 1H), 3.12 (dd, J = 15.5, 7.0 Hz, 1H), 2.17 (m, 1H), 2.08 (m, 1H), 2.00–1.98 (m, 2H), 1.74 (ddd, J = 13.5, 13.5, 4.0 Hz, 1H), 1.47 (m, 1H). ^{13}C NMR ($CDCl_3$) δ 189.9, 144.4, 139.5, 134.1, 132.5, 128.8, 128.6, 128.1, 124.8, 118.5, 76.9, 72.6, 45.4, 37.5, 29.9, 20.8. Mp. 94.3–95.2 °C. TLC: R_f 0.37 (hexane/EtOAc = 3:1). IR (KBr): 3088, 2957, 2846, 1661, 1517, 1491, 1448, 1411, 1383, 1368, 1356, 1293, 1247, 1216, 1177, 1091, 1032, 972, 943, 918, 869, 850, 752, 732, 695 cm^{-1}. HRMS Calcd for $C_{18}H_{17}NO_2SNa$: $[M+Na]^+$, 334.0872. Found: m/z 334.0867. HPLC (Daicel Chiralpak IE, hexane/i-PrOH = 92.5/7.5, flow rate = 1.5 mL/min, λ = 254 nm, 40 °C): t_{minor} = 28.5 min, t_{major} = 22.4 min.

6-(2-Oxopropyl)-2-phenyltetrahydro-2*H*-pyran-2-carbonitrile (3e).
The diastereomers were further separated by flash silica gel column chromatography using hexane/EtOAc (v/v = 3/1) as an eluent.

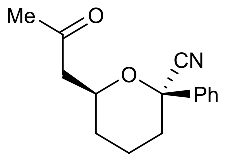

Yield: 58% (21.0 mg), dr = 14:1, 91% *ee*, pale yellow oil. $[\alpha]_D^{18}$ +42.4 (c 0.38, CH_2Cl_2). ^{1}H NMR ($CDCl_3$) δ 7.52 (m, 2H), 7.42–7.34 (m, 3H), 4.48 (m, 1H), 2.79 (dd, J = 16.0, 7.0 Hz, 1H), 2.65 (dd, J = 16.0, 5.0 Hz, 1H), 2.21 (s, 3H), 2.17 (m, 1H), 2.06 (m, 1H), 1.97 (m, 1H), 1.87 (m, 1H), 1.73 (ddd, J = 14.0, 14.0, 4.0 Hz, 1H), 1.37 (m, 1H). ^{13}C NMR ($CDCl_3$) δ 206.0, 139.4, 128.9, 128.7, 124.7, 118.5, 76.9, 72.4, 49.8, 37.5, 30.4, 29.9, 20.7. TLC: R_f 0.29 (hexane/EtOAc = 3:1). IR (neat): 2948, 2869, 1718, 1493, 1450, 1363, 1283, 1242, 1163, 1084, 1047, 1004, 955, 879, 760, 699 cm^{-1}. HRMS Calcd for $C_{15}H_{17}NO_2Na$: $[M+Na]^+$, 266.1152. Found: m/z 266.1149. HPLC (Daicel Chiralpak IE, hexane/i-PrOH = 92.5/7.5, flow rate = 1.5 mL/min, λ = 254 nm, 40 °C): t_{minor} = 13.1 min, t_{major} = 11.1 min.

***S*-Phenyl 2-(6-cyano-6-phenyltetrahydro-2*H*-pyran-2-yl)ethanethioate (3f)**.

The diastereomers were further separated by flash silica gel column chromatography using hexane/EtOAc (v/v = 5/1) as an eluent.

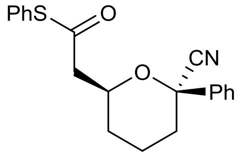

Yield: 70% (35.5 mg), dr = >20:1, 96% *ee*, colorless oil. $[\alpha]_D^{28}$ +75.3 (*c* 0.72, CH_2Cl_2). ^{1}H NMR ($CDCl_3$) δ 7.58 (m, 2H), 7.45–7.35 (m, 8H), 4.52 (m, 1H), 3.04 (dd, *J* = 15.0, 7.0 Hz, 1H), 2.92 (dd, *J* = 15.0, 6.0 Hz, 1H), 2.20 (m, 1H), 2.11–1.97 (m, 2H), 1.90 (m, 1H), 1.75 (ddd, *J* = 13.5, 13.5, 4.0 Hz, 1H), 1.48 (m, 1H). ^{13}C NMR ($CDCl_3$) δ 194.1, 139.4, 134.5, 129.5, 129.2, 128.9, 128.7, 127.4, 124.8 (2C), 118.5, 72.6, 49.4, 37.4, 29.6, 20.8. TLC: R_f 0.30 (hexane/EtOAc = 5:1). IR (neat): 3061, 2947, 2924, 1697, 1479, 1450, 1280, 1239, 1087, 1047, 988, 961, 748, 696 cm^{-1}. HRMS Calcd for $C_{20}H_{19}NO_2SNa$: $[M+Na]^+$, 360.1029. Found: *m/z* 360.1018. HPLC (Daicel Chiralpak IE, hexane/*i*-PrOH = 90.0/10.0, flow rate = 2.0 mL/min, λ = 254 nm, 40 °C): t_{major} = 5.5 min, t_{minor} = 6.2 min.

2-(4-Methoxyphenyl)-6-(2-oxo-2-phenylethyl)tetrahydro-2*H*-pyran-2-carbonitrile (3g).

The diastereomers were further separated by flash silica gel column chromatography using hexane/EtOAc (v/v = 5/1) as an eluent.

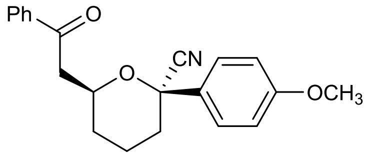

Yield: 55% (29.5 mg), dr = >20:1, 98% *ee*, white solid. $[\alpha]_D^{18}$ +30.6 (*c* 0.63, CH_2Cl_2). ^{1}H NMR ($CDCl_3$) δ 7.97 (m, 2H), 7.58 (m, 1H), 7.47 (m, 2H), 7.41 (m, 2H), 6.88 (m, 2H), 4.63 (m, 1H), 3.80 (s, 3H), 3.40 (dd, *J* = 16.5, 5.5 Hz, 1H), 3.16 (dd, *J* = 16.0, 6.5 Hz, 1H), 2.17 (m, 1H), 2.08 (m, 1H), 2.01–1.95 (m, 2H), 1.76 (ddd, *J* = 13.5, 13.5, 4.0 Hz, 1H), 1.41 (m, 1H). ^{13}C NMR ($CDCl_3$) δ 197.2, 159.8, 137.0, 133.3, 131.7, 128.6, 128.2, 126.2, 118.8, 113.9, 76.5, 72.7, 55.3, 44.8, 37.2, 30.0, 20.9. Mp. 79.2–80.0 °C. TLC: R_f 0.32 (hexane/EtOAc = 3:1). IR (KBr): 3355, 2888, 2837, 1691, 1678, 1609, 1516, 1511, 1452, 1363, 1306, 1259, 1214, 1176, 1129, 1099, 1035, 997, 962, 906, 820, 763, 694 cm^{-1}. HRMS Calcd for $C_{21}H_{21}NO_3Na$: $[M+Na]^+$, 358.1414. Found: *m/z* 358.1407. HPLC (Daicel Chiralpak IE, hexane/*i*-PrOH = 92.5/7.5, flow rate = 1.5 mL/min, λ = 254

nm, 40 °C): t_{minor} = 13.1 min, t_{major} = 11.1 min.

2-(4-Fluorophenyl)-6-(2-oxo-2-phenylethyl)tetrahydro-2*H*-pyran-2-carbonitrile (3h).

The diastereomers were further separated by flash silica gel column chromatography using hexane/EtOAc (v/v = 5/1) as an eluent.

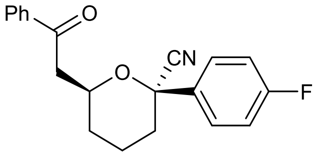

Yield: 89% (46.2 mg), dr = >20:1, 94% *ee*, colorless oil. $[\alpha]_D^{18}$ +23.0 (*c* 1.22, CH_2Cl_2). ^{1}H NMR ($CDCl_3$) δ 7.98 (m, 2H), 7.58 (m, 1H), 7.49–7.43 (m, 4H), 7.04 (m, 2H), 4.65 (m, 1H), 3.40 (dd, *J* = 16.0, 6.0 Hz, 1H), 3.18 (dd, *J* = 16.0, 6.5 Hz, 1H), 2.17 (m, 1H), 2.08 (m, 1H), 2.02–1.96 (m, 2H), 1.71 (ddd, *J* = 13.5, 13.5, 4.0 Hz, 1H), 1.44 (m, 1H). ^{13}C NMR ($CDCl_3$) δ 197.1, 162.7 (d, *J* = 247.6 Hz), 136.9, 135.5 (d, *J* = 2.8 Hz), 133.3, 128.6, 128.2, 126.8 (d, *J* = 8.7 Hz), 118.4, 115.5 (d, *J* = 22.1 Hz), 76.4, 72.8, 44.6, 37.5, 29.9, 20.8. ^{19}F NMR ($CDCl_3$) δ 48.9. TLC: R_f 0.43 (hexane/EtOAc = 3:1). IR (neat): 3068, 2947, 2927, 1683, 1598, 1510, 1449, 1289, 1235, 1161, 1093, 1045, 1002, 965, 832, 754, 691 cm^{-1}. HRMS Calcd for $C_{20}H_{18}FNO_2Na$: $[M+Na]^+$, 346.1214. Found: *m*/*z* 346.1209. HPLC (Daicel Chiralpak IE, hexane/*i*-PrOH = 92.5/7.5, flow rate = 1.5 mL/min, λ = 254 nm, 40 °C): t_{minor} = 13.1 min, t_{major} = 11.1 min.

2-(Naphthalen-1-yl)-6-(2-oxo-2-phenylethyl)tetrahydro-2*H*-pyran-2-carbonitrile (3i).

The diastereomers were further separated by flash silica gel column chromatography using hexane/EtOAc (v/v = 3/1) as an eluent.

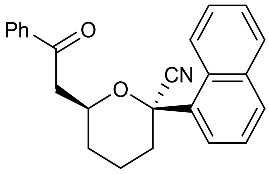

Yield: 75% (42.5 mg), dr = 20:1, 91% *ee*, colorless oil. $[\alpha]_D^{18}$ +54.7 (*c* 0.92, CH_2Cl_2). ^{1}H NMR ($CDCl_3$) δ 8.48 (m, 1H), 7.96 (m, 2H), 7.85 (m, 2H), 7.69 (dd, *J* = 7.5, 1.0 Hz, 1H), 7.55–7.46 (m, 3H), 7.44–7.40 (m, 3H), 4.92 (m, 1H), 3.43 (dd, *J* = 16.5, 6.5 Hz, 1H), 3.16 (dd, *J* = 16.5, 6.0 Hz, 1H), 2.53 (m, 1H), 2.32–2.20 (m, 2H), 2.11 (m, 1H), 2.06 (m, 1H), 1.55 (m, 1H). ^{13}C NMR ($CDCl_3$) δ 197.0, 136.9, 134.4, 133.6, 133.2, 130.5, 129.9, 128.9, 128.5, 128.1, 126.3, 125.8, 125.3, 124.6, 123.4, 118.5, 75.8, 72.5, 44.6, 34.6, 30.1,

20.7. TLC: R_f 0.36 (hexane/EtOAc = 3:1). IR (neat): 3356, 3055, 2942, 2870, 1683, 1598, 1450, 1387, 1363, 1290, 1221, 1181, 1055, 1036, 976, 945, 802, 778, 736, 690 cm^{-1}. HRMS Calcd for $C_{24}H_{21}NO_2Na$: $[M+Na]^+$, 378.1465. Found: *m/z* 378.1459. HPLC (Daicel Chiralpak IE, hexane/*i*-PrOH = 92.5/7.5, flow rate = 1.5 mL/min, λ = 254 nm, 40 °C): t_{minor} = 13.1 min, t_{major} = 11.1 min.

6-(2-Oxo-2-phenylethyl)-2-(thiophen-2-yl)tetrahydro-2*H*-pyran-2-carbonitrile (3j). The diastereomers were further separated by flash silica gel column chromatography using hexane/EtOAc (v/v = 5/1) as an eluent.

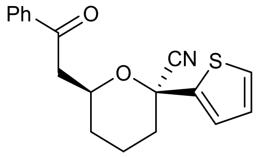

Yield: 46% (21.3 mg), dr = >20:1, 95% *ee*, colorless oil. $[\alpha]_D^{18}$ +20.9 (*c* 0.49, CH_2Cl_2). ^1H NMR ($CDCl_3$) δ 7.97(m, 2H), 7.57 (tt, *J* = 7.5, 1.5 Hz, 1H), 7.47 (m, 2H), 7.32 (dd, *J* = 5.0, 1.5 Hz, 1H), 7.20 (dd, *J* = 3.5, 1.5 Hz, 1H), 6.98 (dd, *J* = 5.0, 3.5 Hz, 1H), 4.63 (m, 1H), 3.21 (dd, *J* = 16.0, 6.0 Hz, 1H), 3.13 (dd, *J* = 16.0, 7.0 Hz, 1H), 2.31 (m, 1H), 2.09 (m, 1H), 2.03–1.93 (m, 3H), 1.42 (m, 1H). ^{13}C NMR ($CDCl_3$) δ 197.0, 142.9, 137.0, 133.2, 128.6, 128.2, 126.6, 126.2, 124.9, 117.9, 74.2, 73.2, 44.7, 37.4, 30.0, 20.7. IR (neat): 3087, 2938, 1680, 1596, 1449, 1290, 1252, 1096, 1035, 996, 837, 756, 701, 510 cm^{-1}. HRMS Calcd for $C_{18}H_{17}NO_2S$: $[M+Na]^+$, 334.0872. Found: *m/z* 334.0864. HPLC (Daicel Chiralpak IE, hexane/*i*-PrOH = 92.5/7.5, flow rate = 1.5 mL/min, λ = 254 nm, 40 °C): t_{minor} = 29.1 min, t_{major} = 17.4 min.

2-(*tert*-Butyl)-6-(2-oxo-2-phenylethyl)tetrahydro-2*H*-pyran-2-carbonitrile (3k). The diastereomers were further separated by flash silica gel column chromatography using hexane/EtOAc (v/v = 10/1) as an eluent.

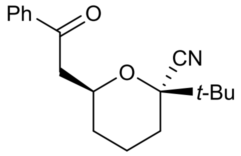

Yield: 59% (730 mg), dr = >20:1, 94% *ee*, white solid. $[\alpha]_D^{18}$ –1.4 (*c* 0.69, CH_2Cl_2). ^1H NMR ($CDCl_3$) δ 7.96 (m, 2H), 7.56 (m, 1H), 7.46 (m, 2H), 4.36 (m, 1H), 3.24 (dd, *J* = 15.5, 6.5 Hz, 1H), 3.02 (dd, *J* = 15.5, 6.0 Hz, 1H), 1.92–1.87 (m, 2H), 1.85 (m, 1H), 1.77 (m, 1H), 1.57 (m, 1H), 1.27 (m, 1H), 0.96 (s, 9H). ^{13}C NMR ($CDCl_3$) δ 197.8, 137.2, 133.1, 128.5, 128.3, 118.7, 82.2, 72.6, 44.7, 37.8, 30.2, 28.1, 24.7, 20.5. Mp.

82.3–83.0 °C. TLC: R_f 0.23 (hexane/EtOAc = 10:1). IR (KBr): 2984, 2938, 2920, 2877, 1666, 1596, 1452, 1369, 1333, 1286, 1212, 1172, 1111, 1090, 1047, 984, 969, 944, 892, 860, 839, 808, 750, 700 cm^{-1}. HRMS Calcd for $C_{18}H_{23}NO_2Na$: $[M+Na]^+$, 308.1621. Found: *m*/*z* 308.1616. HPLC (Daicel Chiralpak IE, hexane/*i*-PrOH = 92.5/7.5, flow rate = 1.5 mL/min, λ = 254 nm, 40 °C): t_{minor} = 10.6 min, t_{major} = 9.4 min.

2-Isopropyl-6-(2-oxo-2-phenylethyl)tetrahydro-2*H*-pyran-2-carbonitrile (3l).
The diastereomers were further separated by flash silica gel column chromatography using hexane/EtOAc (v/v = 5/1) as an eluent.

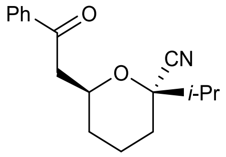

Yield: 99% (43.0 mg), dr = >20:1, 95% *ee*, colorless oil. $[\alpha]_D^{18}$ –0.0965 (*c* 1.14, CH_2Cl_2). ^{1}H NMR ($CDCl_3$) δ 7.95 (m, 2H), 7.56 (tt, *J* = 7.0, 3.0 Hz, 1H), 7.45 (m, 2H), 4.37 (m, 1H), 3.26 (dd, *J* = 16.0, 6.5 Hz, 1H), 3.02 (dd, *J* = 16.0, 6.5 Hz, 1H), 1.93–1.80 (m, 5 H), 1.44 (m, 1H), 1.28 (m, 1H), 0.99 (d, *J* = 1.5 Hz, 3H), 0.98 (d, *J* = 1.5 Hz, 3H). ^{13}C NMR ($CDCl_3$) δ 197.5, 137.1, 133.0, 128.5, 128.2, 118.3, 79.8, 72.4, 44.8, 37.4, 31.7, 30.3, 20.4, 16.9. IR (neat): 2924, 1706, 1598, 1448, 1291, 1205, 1061, 993, 755, 694, 497 cm^{-1}. HRMS Calcd for $C_{17}H_{21}NO_2Na$: $[M+Na]^+$, 294.1465. Found: *m*/*z* 294.1456. HPLC (Daicel Chiralpak IE, hexane/*i*-PrOH = 92.5/7.5, flow rate = 1.5 mL/min, λ = 254 nm, 40 °C): t_{minor} = 14.5 min, t_{major} = 12.7 min.

6-(2-Oxo-2-phenylethyl)-2-pentyltetrahydro-2*H*-pyran-2-carbonitrile (3m).
The diastereomers were further separated by flash silica gel column chromatography using hexane/EtOAc (v/v = 10/1) as an eluent.

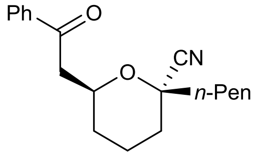

Yield: 99% (48.3 mg), dr = >20:1, 82% *ee*, white solid. $[\alpha]_D^{18}$ +1.6 (*c* 1.16, CH_2Cl_2). ^{1}H NMR ($CDCl_3$) δ 7.94 (m, 2H), 7.56 (m, 1H), 7.45 (m, 2H), 4.37 (m, 1H), 3.27 (dd, *J* = 16.0, 6.0 Hz, 1H), 3.01 (dd, *J* = 16.0, 6.5 Hz, 1H), 1.89–1.85 (m, 4H), 1.70–1.66 (m, 2H), 1.50–1.41 (m, 3H), 1.32–1.23 (m, 5H), 0.86 (t, *J* = 7.0 Hz, 3H). ^{13}C NMR ($CDCl_3$) δ 197.7, 137.3, 133.4, 128.8, 128.5, 119.5, 76.0, 72.6, 45.1, 40.9, 34.7, 31.8, 30.7, 23.5, 22.7, 20.7, 14.2. Mp. 44.5–45.3 °C. TLC: R_f 0.14 (hexane/EtOAc = 10:1). IR

(KBr): 2954, 2931, 2870, 1685, 1598, 1449, 1388, 1364, 1289, 1203, 1068, 1044, 991, 909, 753, 691 cm^{-1}. HRMS Calcd for $C_{19}H_{25}NO_2Na$: $[M+Na]^+$, 322.1778. Found: *m*/*z* 322.1772. HPLC (Daicel Chiralpak IE, hexane/*i*-PrOH = 92.5/7.5, flow rate = 1.5 mL/min, λ = 254 nm, 40 °C): t_{minor} = 22.7 min, t_{major} = 13.1 min.

2-Methyl-6-(2-oxo-2-phenylethyl)tetrahydro-2*H*-pyran-2-carbonitrile (3n).
The diastereomers were further separated by flash silica gel column chromatography using hexane/EtOAc (v/v = 5/1) as an eluent.

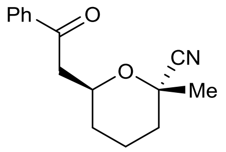

Yield: 99% (39.9 mg), dr = 19:1, 93% *ee*, colorless oil. $[\alpha]_D^{18}$ –0.47 (*c* 0.85, CH_2Cl_2). ^1H NMR ($CDCl_3$) δ 7.95 (m, 2H), 7.56 (m, 1H), 7.46 (m, 2H), 4.40 (m, 1H), 3.39 (dd, *J* = 16.5, 6.0 Hz, 1H), 3.03 (dd, *J* = 16.5, 7.0 Hz, 1H), 1.93–1.83 (m, 4H), 1.55 (s, 3H), 1.50 (m, 1H), 1.27 (m, 1H). ^{13}C NMR ($CDCl_3$) δ 197.1, 136.9, 133.1, 128.6, 128.1, 119.8, 72.2, 71.9, 44.8, 35.8, 30.0, 27.7, 20.5. TLC: R_f 0.36 (hexane/EtOAc = 3:1). IR (neat): 2941, 2927, 1684, 1597, 1450, 1364, 1290, 1209, 1123, 1075, 1047, 976, 907, 872, 754, 691 cm^{-1}. HRMS Calcd for $C_{15}H_{17}NO_2Na$: $[M+Na]^+$, 266.1152. Found: *m*/*z* 266.1148. HPLC (Daicel Chiralpak IE, hexane/*i*-PrOH = 92.5/7.5, flow rate = 1.5 mL/min, λ = 254 nm, 40 °C): t_{minor} = 13.1 min, t_{major} = 11.1 min.

2-Phenyl-2-((trimethylsilyl)oxy)hexanenitrile (7): CAS RN [801219-24-9].

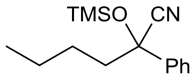

Yield: 36% (14.0 mg), 17% *ee*, yellow oil. $[\alpha]_D^{28}$ –5.70 (*c* 0.19, CH_2Cl_2). ^1H NMR ($CDCl_3$) δ 7.52–7.50 (m, 2H), 7.41–7.32 (m, 3H), 2.04–1.99 (m, 1H), 1.93–1.87 (m, 1H), 1.50–1.45 (m, 1H), 1.33–1.26 (m, 3H), 0.87 (t, *J* = 7.0 Hz, 3H), 0.13 (s, 9H). ^{13}C NMR ($CDCl_3$) δ 141.1, 128.5 (2C), 125.0, 121.0, 75.6, 45.8, 26.4, 22.3, 13.8, 0.9.

Procedure for synthesis of 9

Under argon atmosphere, **3a** (0.031 g, 0.10 mmol) in Et_2O (1.0 mL) was added dropwise to a suspension of $LiAlH_4$ (0.019 g, 0.50 mmol) in Et_2O (1.0 mL) at 0 °C. The mixture was allowed to warm to ambient temperature and stirred for 3 h. Next, H_2O (19 μL), 15% aqueous NaOH (19 μL), and H_2O (57 μL) were sequentially added dropwise at

0 °C, and the mixture was sttired for a while at ambient temperature. Subsequently, the reaction mixture was filtered through a Celite pad, and the Celite pad was washed with Et_2O. The filtrate was concentrated in vacuo to afford **8**, which was used for the next step without further purification.

To a round-bottom flask were added sequentially **8** (0.031 g, 0.10 mmol), CH_2Cl_2 (2.0 mL), and MnO_2 (0.087 g, 1.0 mmol). After being stirred for 36.5 h at ambient temperature, the crude reaction mixture was filtered through a Celite pad, and the Celite pad was washed with CH_2Cl_2. The filtrate was concentrated in vacuo. Purification by flash silica gel column chromatography using $CHCl_3$/MeOH (v/v = 10/1) as an eluent gave **9**.

2-(6-(Aminomethyl)-6-phenyltetrahydro-2*H*-pyran-2-yl)-1-phenylethan-1-one (9).

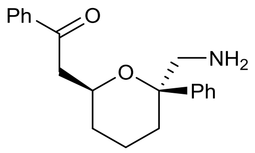

Yield: 40% (12.4 mg), yellow oil. $[\alpha]_D^{28}$ +60.8 (*c* 0.41, CH_2Cl_2).
^{1}H NMR ($CDCl_3$) δ 8.03 (m, 2H), 7.60 (tt, *J* = 7.0, 1.5 Hz, 1H), 7.50 (m, 2H), 7.28–7.23 (m, 4H), 7.18 (m, 1H), 4.39 (m, 1H), 3.65 (d, *J* = 14.0 Hz, 1H), 3.47 (dd, *J* = 16.5, 3.0 Hz, 1H), 2.95 (dd, *J* = 16.5, 3.0 Hz, 1H), 2.61 (d, *J* = 14.0 Hz, 1H), 2.01 (m, 1H), 1.93–1.74 (m, 5H), 1.62 (ddd, *J* = 13.5, 13.0, 4.5 Hz, 1H), 1.41 (ddd, *J* = 25.0, 13.0, 4.5 Hz, 1H). ^{13}C NMR ($CDCl_3$) δ 198.7, 145.9, 137.4, 133.2, 128.6, 128.2, 128.0, 126.5, 124.8, 77.8, 66.7, 45.1, 44.8, 33.3, 30.8, 20.1. TLC: R_f 0.22 ($CHCl_3$/MeOH = 10:1). IR (neat): 3351, 3057, 2910, 1683, 1957, 1449, 1395, 1288, 1219, 1054, 900, 768, 700, 582. HRMS Calcd for $C_{20}H_{24}NO_2$: $[M+H]^+$, 310.1802. Found: *m*/*z* 310.1794.

The enantiomeric excess of **9** was determined by HPLC analysis after acylation.

Procedure for acylation of 9

To a round-bottom flask were added sequentially **9** (12.4 mg, 0.040 mmol), CH_2Cl_2 (2.0 mL), acetic anhydride (9.5 μL, 0.10 mmol), and pyridine (16 μL, 0.20 mmol). After being stirred for 12 h at ambient temperature, the crude reaction mixture was concentrated in vacuo. Purification by flash silica gel column chromatography using $CHCl_3$/MeOH (v/v = 10/1) as an eluent gave *N*-((6-(2-oxo-2-phenylethyl)-2-phenyltetrahydro-2*H*-pyran-2-yl)methyl)acetamide (**9´**).

***N*-((6-(2-Oxo-2-phenylethyl)-2-phenyltetrahydro-2*H*-pyran-2-yl)methyl)acetamide (9')**.

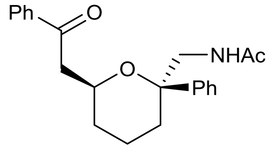

Yield: 90% (12.6 mg), 97% *ee*, white solid. $[\alpha]_D^{28}$ +56.5 (*c* 0.31, CH_2Cl_2).
^{1}H NMR ($CDCl_3$) δ 8.03 (m, 2H), 7.62 (m, 1H), 7.51 (m, 2H), 7.33 (m, 2H), 7.24 (m, 2H), 7.18 (m, 1H), 7.01 (br s, 1H), 4.36 (m, 1H), 3.88 (m, 2H), 3.46 (dd, *J* = 17.5, 9.5 Hz, 1H), 3.03 (dd, *J* = 17.5, 2.0 Hz, 1H), 2.00 (m, 1H), 1.98 (s, 3H), 1.96–1.83 (m, 2H), 1.76 (m, 1H), 1.66 (ddd, *J* = 13.0, 13.0, 5.0 Hz, 1H), 1.47 (ddd, *J* = 25.0, 13.0, 4.5 Hz, 1H). ^{13}C NMR ($CDCl_3$) δ 198.6, 170.8, 145.5, 136.9, 133.5, 128.7, 128.1, 127.9, 126.8, 124.6, 76.3, 66.3, 44.8, 41.5, 33.8, 30.2, 23.0, 20.2. TLC: R_f 0.30 ($CHCl_3$/MeOH = 20:1). IR (KBr): 3357, 2941, 1672, 1545, 1448, 1372, 1285, 1221, 1174, 1065, 1000, 757, 700, 594 cm^{-1}. HRMS Calcd for $C_{22}H_{25}NO_3Na$: $[M+Na]^+$, 374.1727. Found: *m*/*z* 374.1715. HPLC (Daicel Chiralpak ID, hexane/*i*-PrOH = 85/15, flow rate = 2.0 mL/min, λ = 254 nm, 40 °C): t_{major} = 10.2 min, t_{minor} = 12.2 min.

Procedure for synthesis of 11

Under argon atmosphere, **3a** (0.031 g, 0.10 mmol) in CH_2Cl_2 (1.0 mL) was added dropwise to a suspension of DIBAL (0.40 mL, 1.0 M solution in toluene, 0.40 mmol) at –40 °C. The mixture was stirred for 3 h at –40 °C. Next, H_2O (3.0 mL) were added dropwise, and the mixture was sttired for a while at ambient temperature. The aqueous layers were extracted with EtOAc (25 mL × 3). The combined organic layers were washed with saturated aqueous NH_4Cl (25 mL), saturated aqueous $NaHCO_3$ (25 mL), and brine (25 mL), dried over Na_2SO_4, and concentrated in vacuo. Purification by flash silica gel column chromatography using hexane/EtOAc (v/v = 4/1) as an eluent gave **10**.

To a round-bottom flask were added sequentially **10** (0.018 g, 0.056 mmol), CH_2Cl_2 (2.0 mL), and PCC (0.037 g, 0.17 mmol)/MS 4A. After being stirred for 20 h at ambient temperature, the crude reaction mixture was filtered through a Celite pad, and the Celite pad was washed with CH_2Cl_2. The filtrate was concentrated in vacuo. Purification by flash silica gel column chromatography using hexane/EtOAc (v/v = 5/1) as an eluent gave **11**.

6-(2-Hydroxy-2-phenylethyl)-2-phenyltetrahydro-2*H*-pyran-2-carbaldehyde (10).

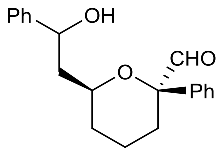

Yield: 56% (17.5 mg), dr = 1.5:1, colorless oil. $[\alpha]_D^{28}$ +190.0 (*c* 0.62, CH_2Cl_2). IR (neat): 3440, 3060, 2953, 1735, 1612, 1502, 1455, 1381, 1049, 925, 768, 734, 701 cm^{-1}. HRMS Calcd for $C_{20}H_{22}O_3Na$: $[M+Na]^+$, 333.1461. Found: *m*/*z* 333.1449.

Major diastereomer

^1H NMR ($CDCl_3$) δ 9.47 (s, 1H), 7.45–7.35 (m, 8H), 7.34–7.27 (m, 2H), 5.12 (dd, *J* = 8.5, 5.0 Hz, 1H), 3.81 (m, 1H), 3.38 (br s, 1H), 2.61 (m, 1H), 2.24 (ddd, *J* = 14.5, 9.5, 8.5 Hz, 1H), 1.95 (ddd, *J* = 14.5, 4.5, 3.0 Hz, 1H), 1.80 (m, 1H), 1.63–1.57 (m, 3H), 1.40 (m, 1H). ^{13}C NMR ($CDCl_3$) δ 201.4, 143.7, 138.0, 128.8, 128.6, 128.3, 127.8, 126.0, 125.2, 84.5, 75.5, 73.8, 45.5, 30.9, 30.6, 20.5. TLC: R_f 0.29 (hexane/EtOAc = 4:1).

Minor diastereomer

^1H NMR ($CDCl_3$) δ 9.47 (s, 1H), 7.45–7.35 (m, 8H), 7.34–7.27 (m, 2H), 5.24 (dd, *J* = 8.5, 3.0 Hz, 1H), 3.96 (m, 1H), 3.07 (br s, 1H), 2.66 (m, 1H), 2.11 (ddd, *J* = 14.0, 9.0, 3.0 Hz, 1H), 2.01 (ddd, *J* = 14.0, 9.0, 3.0 Hz, 1H), 1.84 (m, 1H), 1.63–1.57 (m, 3H), 1.40 (m, 1H). ^{13}C NMR ($CDCl_3$) δ 201.9, 144.3, 139.4, 128.8, 128.5, 128.3, 127.4, 125.6, 125.4, 84.4, 72.6, 70.9, 45.0, 30.5, 30.4, 20.8. TLC: R_f 0.36 (hexane/EtOAc = 4:1).

6-(2-Oxo-2-phenylethyl)-2-phenyltetrahydro-2*H*-pyran-2-carbaldehyde (11).

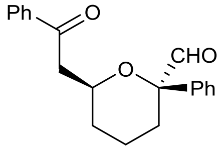

Yield: 78% (13.5 mg), 97% *ee*, colorless oil. $[\alpha]_D^{28}$ +213.5 (*c* 0.51, CH_2Cl_2).
^1H NMR ($CDCl_3$) δ 9.49 (s, *J* = 2.5 Hz, 1H), 8.04 (m, 2H), 7.61 (m, 1H), 7.51 (m, 2H), 7.32–7.23 (m, 5H), 4.33 (m, 1H), 3.55 (dd, *J* = 15.5, 7.5 Hz, 1H), 3.04 (dd, *J* = 15.5, 4.5 Hz, 1H), 2.67 (m, 1H), 1.85 (m, 1H), 1.78 (m, 1H), 1.68 (m, 1H), 1.52 (m, 1H), 1.43 (m, 1H). ^{13}C NMR ($CDCl_3$) δ 202.3, 198.1, 138.3, 137.3, 133.2, 128.6, 128.5, 128.3, 128.0, 125.4, 84.3, 72.4, 45.2, 30.5, 30.0, 20.6. TLC: R_f 0.45 (hexane/EtOAc = 4:1). IR (neat): 3351, 2937, 1729, 1691, 1587, 1455, 1374, 1280, 1134, 1054, 755, 683 cm^{-1}. HRMS Calcd for $C_{20}H_{20}O_3Na$: $[M+Na]^+$, 331.1305. Found: *m*/*z* 331.1294. HPLC (Daicel Chiralpak IE, hexane/*i*-PrOH = 95.0/5.0, flow rate = 1.5 mL/min, λ = 254 nm, 40 °C): t_{major} = 10.1 min, t_{minor} = 14.1 min.

6-Oxo-2-(2-oxo-2-phenylethyl)-6-phenylhexanenitrile (38).

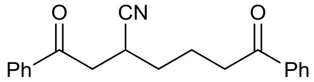

Yield: 12% (35.5 mg), white solid. ^{1}H NMR ($CDCl_3$) δ 7.97–7.95 (m, 4H), 7.61 (tt, *J* = 7.5, 1.5 Hz, 1H), 7.58 (tt, *J* = 7.5, 1.5 Hz, 1H), 7.51–7.46 (m, 4H), 3.45 (dd, *J* = 17.5, 6.5 Hz, 1H), 3.35 (m, 1H), 3.29 (dd, *J* = 17.0, 6.5 Hz, 1H), 3.14–3.03 (m, 2H), 2.06 (m, 1H), 1.98 (m, 1H), 1.81 (m, 2H). ^{13}C NMR ($CDCl_3$) δ 199.1, 195.2, 136.6, 135.8, 133.9, 133.2, 128.8, 128.7, 128.0, 128.0, 121.7, 40.6, 37.4, 31.3, 26.2, 21.5. TLC: R_f 0.31 (hexane/EtOAc = 3:1). IR (KBr): 2956, 2239, 1689, 1682, 1597, 1448, 1364, 1253, 1206, 971, 759, 731, 691, 570 cm^{-1}. HRMS Calcd for $C_{20}H_{19}NO_2Na$: [M+Na]$^+$, 328.1308. Found: *m*/*z* 328.1301.

(*E*)-8-Oxo-2,8-diphenyl-2-((trimethylsilyl)oxy)oct-6-enenitrile (39).

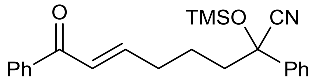

Yield: 27% (15.5 mg), 98% *ee*, yellow oil. $[\alpha]_D^{28}$ –13.5 (*c* 0.22, CH_2Cl_2).
^{1}H NMR ($CDCl_3$) δ 7.91 (m, 2H), 7.56 (tt, *J* = 7.5, 1.5 Hz, 1H), 7.51–7.45 (m, 4H), 7.42–7.34 (m, 3H), 6.99 (dt, *J* = 15.5, 7.0, 1H), 6.86 (dt, *J* = 15.5, 1.5 Hz, 1H), 2.33 (ddd, *J* = 14.0, 7.0, 1.5 Hz, 2H), 2.07 (ddd, *J* = 14.0, 12.0, 5.0 Hz, 1H), 1.95 (ddd, *J* = 14.0, 12.0, 5.0 Hz, 1H), 1.77 (m, 1H), 1.62 (m, 1H), 0.13 (s, 9H). ^{13}C NMR ($CDCl_3$) δ 199.1, 148.4, 140.7, 137.7, 132.7, 128.8, 128.6, 128.5 (2C), 126.3, 125.0, 120.7, 75.4, 45.5, 32.0, 22.9, 0.91. TLC: R_f 0.51 (hexane/EtOAc = 5:1). IR (neat): 3434, 2926, 1672, 1624, 1447, 1254, 1108, 1025, 844, 755, 698, 485 cm^{-1}. HRMS Calcd for $C_{23}H_{27}NO_2SiNa$: [M+Na]$^+$, 400.1703. Found: *m*/*z* 400.1696. HPLC (Daicel Chiralpak IB, hexane/*i*-PrOH = 99.5/0.5, flow rate = 10.0 mL/min, λ = 254 nm, 40 °C): t_{major} = 11.5 min, t_{minor} = 11.1 min.

5-(2-Oxo-2-phenylethyl)-2-phenyltetrahydrofuran-2-carbonitrile (40).
The diastereomers could not be separated.

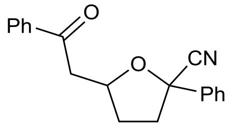

Yield: 99% (44.3 mg), dr = 1.4:1, colorless oil. TLC: R_f 0.28 (CH_2Cl_2/hexane = 20:1). IR (neat): 3060, 2954, 1680, 1598, 1581, 1449, 1383, 1230, 1212, 1053, 1002, 755, 686. $[\alpha]_D^{28}$ –26.8 (*c* 0.14, CH_2Cl_2). HRMS Calcd for $C_{19}H_{17}NO_2Na$: [M+Na]$^+$, 314.1152. Found: *m*/*z* 314.1143.

Major diastereomer: 76% *ee*. ^{1}H NMR (CDCl$_3$) δ 8.02–7.98 (m, 2H), 7.60 (m, 1H), 7.55–7.48 (m, 4H), 7.43–7.35 (m, 3H), 4.97 (m, 1H), 3.62 (ddd, *J* = 17.0, 5.0, 2.0 Hz, 1H), 3.25 (dd, *J* = 17.0, 8.0 Hz, 1H), 2.80 (m, 1H), 2.63 (m, 1H), 2.29 (m, 1H), 1.91 (m, 1H). ^{13}C NMR (CDCl$_3$) δ 197.1, 138.2, 136.6, 135.5, 129.0, 128.77, 128.72, 128.15, 124.9, 120.4, 80.5, 77.4, 43.8, 41.2, 30.7. HPLC (Daicel Chiralpak IE, hexane/*i*-PrOH = 90.0/10.0, flow rate = 2.0 mL/min, λ = 254 nm, 40 °C): t_{major} = 11.2 min, t_{minor} = 12.5 min.

Minor diastereomer: 44% *ee*. ^{1}H NMR (CDCl$_3$) δ 8.02–7.98 (m, 2H), 7.60 (m, 1H), 7.55–7.48 (m, 4H), 7.43–7.35 (m, 3H), 4.97 (m, 1H), 3.76 (ddd, *J* = 17.0, 6.0, 2.0 Hz, 1H), 3.32 (ddd, *J* = 17.0, 7.0, 2.0 Hz, 1H), 2.80 (m, 1H), 2.63 (m, 1H), 2.29 (m, 1H), 2.15 (m, 1H). ^{13}C NMR (CDCl$_3$) δ 197.6, 138.2, 136.6, 133.5, 129.0, 128.82, 128.71, 128.18, 124.8, 121.3, 80.3, 78.8, 44.8, 42.7, 32.7. HPLC (Daicel Chiralpak IE, hexane/*i*-PrOH = 90.0/10.0, flow rate = 2.0 mL/min, λ = 254 nm, 40 °C): t_{major} = 7.0 min, t_{minor} = 9.9 min.

6-(2-Oxo-2-phenylethyl)tetrahydro-2*H*-pyran-2-carbonitrile (41).
The diastereomers could not be separated.

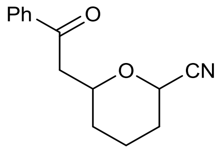

Yield: 99% (37.1 mg), dr = 1.6:1, colorless oil. TLC: R_f 0.38 (hexane/EtOAc = 3:1). IR (neat): 3059, 2920, 1977, 1700, 1597, 1581, 1442, 1387, 1334, 1195, 1158, 1073, 1038, 996, 927, 749. $[\alpha]_D^{28}$ –69.3 (*c* 0.10, CH_2Cl_2). HRMS Calcd for $C_{14}H_{15}NO_2Na$: $[M+Na]^+$, 252.0995. Found: *m*/*z* 252.0992.

Major diastereomer: 70% *ee*. ^{1}H NMR (CDCl$_3$) δ 7.96–7.94 (m, 2H), 7.76 (m, 1H), 7.49–7.46 (m, 2H), 4.84 (m, 1H), 4.43 (m, 1H), 3.28 (dd, *J* = 16.5, 6.0 Hz, 1H), 3.03 (dd, *J* = 16.5, 6.0 Hz, 1H), 1.98–1.79 (m, 5H), 1.39 (m, 1H). ^{13}C (CDCl$_3$) δ 197.0, 136.8, 133.3, 128.6, 128.15, 117.6, 71.4, 64.9, 44.6, 30.6, 28.3, 19.4. HPLC (Daicel Chiralpak IB, hexane/*i*-PrOH = 90.0/10.0, flow rate = 1.0 mL/min, λ = 254 nm, 40 °C): t_{major} = 10.0 min, t_{minor} = 13.0 min.

Minor diastereomer: 95% *ee*. ^{1}H NMR (CDCl$_3$) δ 7.96–7.94 (m, 2H), 7.76 (m, 1H), 7.49–7.46 (m, 2H), 4.31 (m, 1H), 4.07 (m, 1H), 3.39 (dd, *J* = 17.0, 6.0 Hz, 1H), 2.97 (dd, *J* = 17.0, 6.0 Hz, 1H), 1.98–1.79 (m, 4H), 1.64 (m, 1H), 1.39 (m, 1H). ^{13}C (CDCl$_3$) δ 197.2, 136.8, 133.4, 128.6, 128.14, 118.1, 75.2, 66.0, 44.5, 30.3, 29.8, 22.4. HPLC (Daicel Chiralpak IB, hexane/*i*-PrOH = 90.0/10.0, flow rate = 1.0 mL/min, λ = 254 nm, 40 °C): t_{major} = 10.7 min, t_{minor} = 12.5 min.

2-(6-Methyl-6-phenyltetrahydro-2*H*-pyran-2-yl)-1-phenylethan-1-one.

The diastereomers were further separated by preparative TLC using hexane/EtOAc (v/v = 15/1) as an eluent.

Yield: 17% (5.0 mg), dr = 1.4:1.

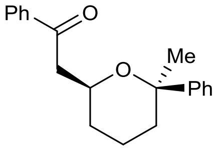

Major diastereomer (42): CAS RN [2087909-75-7], 80% *ee*, colorless oil. ^{1}H NMR ($CDCl_3$) δ 8.00–7.99 (m, 2H), 7.57 (m, 1H), 7.48–7.45 (m, 2H), 7.32–7.28 (m, 4H), 7.20 (m, 1H), 3.95 (m, 1H), 3.31 (dd, J = 15.5, 7.0 Hz, 1H), 2.96 (dd, J = 15.5, 5.5 Hz, 1H), 2.42 (m, 1H), 1.70–1.60 (m, 3H), 1.54 (m, 1H), 1.34 (m, 1H), 1.32 (s, 3H). ^{13}C NMR ($CDCl_3$): δ 198.7, 144.4, 137.4, 132.9, 128.5, 128.4, 128.3, 126.4, 126.1, 76.7, 68.5, 46.0, 34.6, 33.6, 31.6, 20.1. HPLC (Daicel Chiralpak ID, hexane/*i*-PrOH = 90.0/10.0, flow rate = 0.3 mL/min, λ = 254 nm, 40 °C): t_{major} = 18.6 min, t_{minor} = 19.0 min.

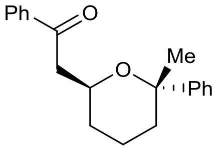

Minor diastereomer (42'): 4.3% *ee*, colorless oil. ^{1}H NMR ($CDCl_3$) δ 8.03–8.01 (m, 2H), 7.58 (m, 1H), 7.49–7.46 (m, 2H), 7.34–7.32 (m, 2H), 7.27–7.23 (m, 2H), 7.18 (m, 1H), 4.44 (m, 1H), 3.40 (dd, J = 15.5, 7.0 Hz, 1H), 2.97 (dd, J = 15.5, 5.5 Hz, 1H), 1.95 (m, 1H), 1.89 (m, 1H), 1.85–1.78 (m, 2H), 1.62 (m, 1H), 1.47 (s, 3H), 1.31 (m, 1H). ^{13}C NMR ($CDCl_3$): δ 199.3, 149.8, 137.6, 133.0, 128.5, 128.4, 127.9, 126.3, 124.1, 75.2, 68.1, 45.8, 35.7, 31.6, 23.1, 20.0. TLC: R_f 0.49 (benzene). IR (neat): 3059, 2971, 2933, 2863, 2360, 2331, 1687, 1597, 1560, 1448, 1384, 1369, 1288, 1215, 1180, 1065, 1041, 1002, 974, 943, 907, 863, 826, 764, 701, 668 cm^{-1}. HRMS Calcd for $C_{20}H_{23}O_2$: [M+H]$^+$, 295.1693. Found: m/z 295.1688. HPLC (Daicel Chiralpak ID, hexane/*i*-PrOH = 95.0/5.0, flow rate = 0.5 mL/min, λ = 254 nm, 40 °C): t_{major} = 11.9 min, t_{minor} = 12.9 min.

NMR Spectra (^{1}H, ^{13}C) of Products

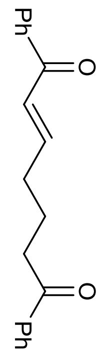

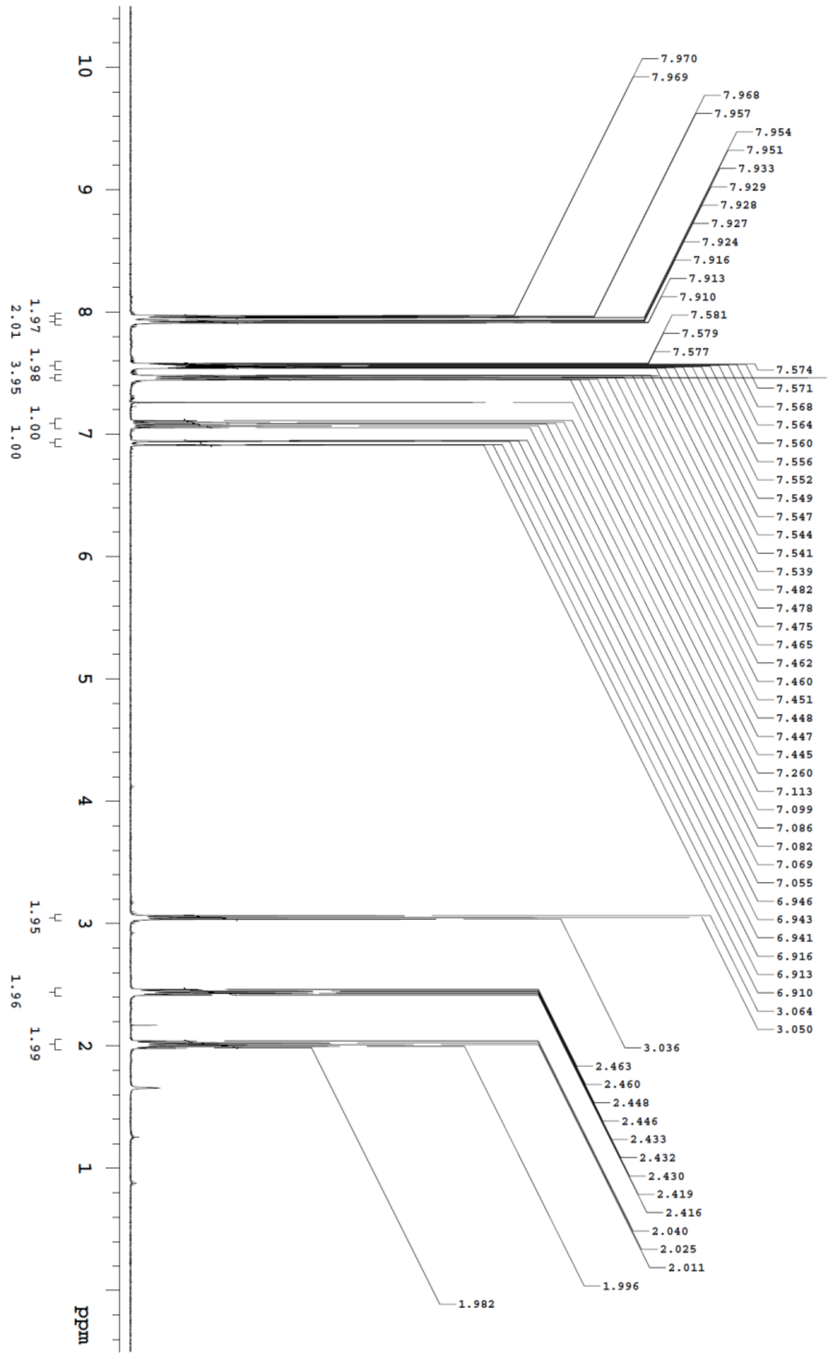

Supplementary Figure 10. ^{1}H NMR spectrum of (*E*)-1,7-diphenylhept-2-ene-1,7-dione (**1a**).

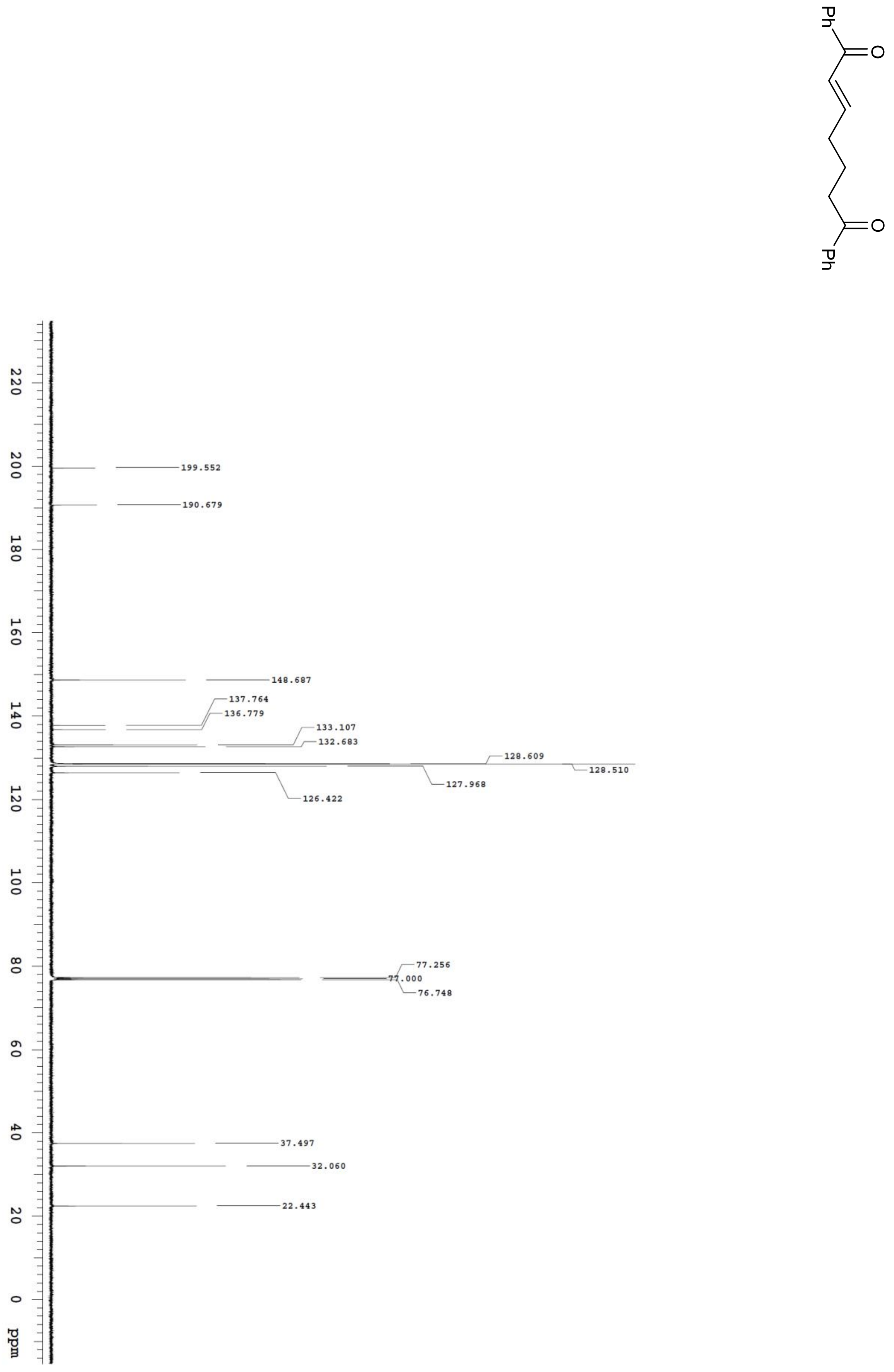

Supplementary Figure 11. ^{13}C NMR spectrum of (*E*)-1,7-diphenylhept-2-ene-1,7-dione (**1a**).

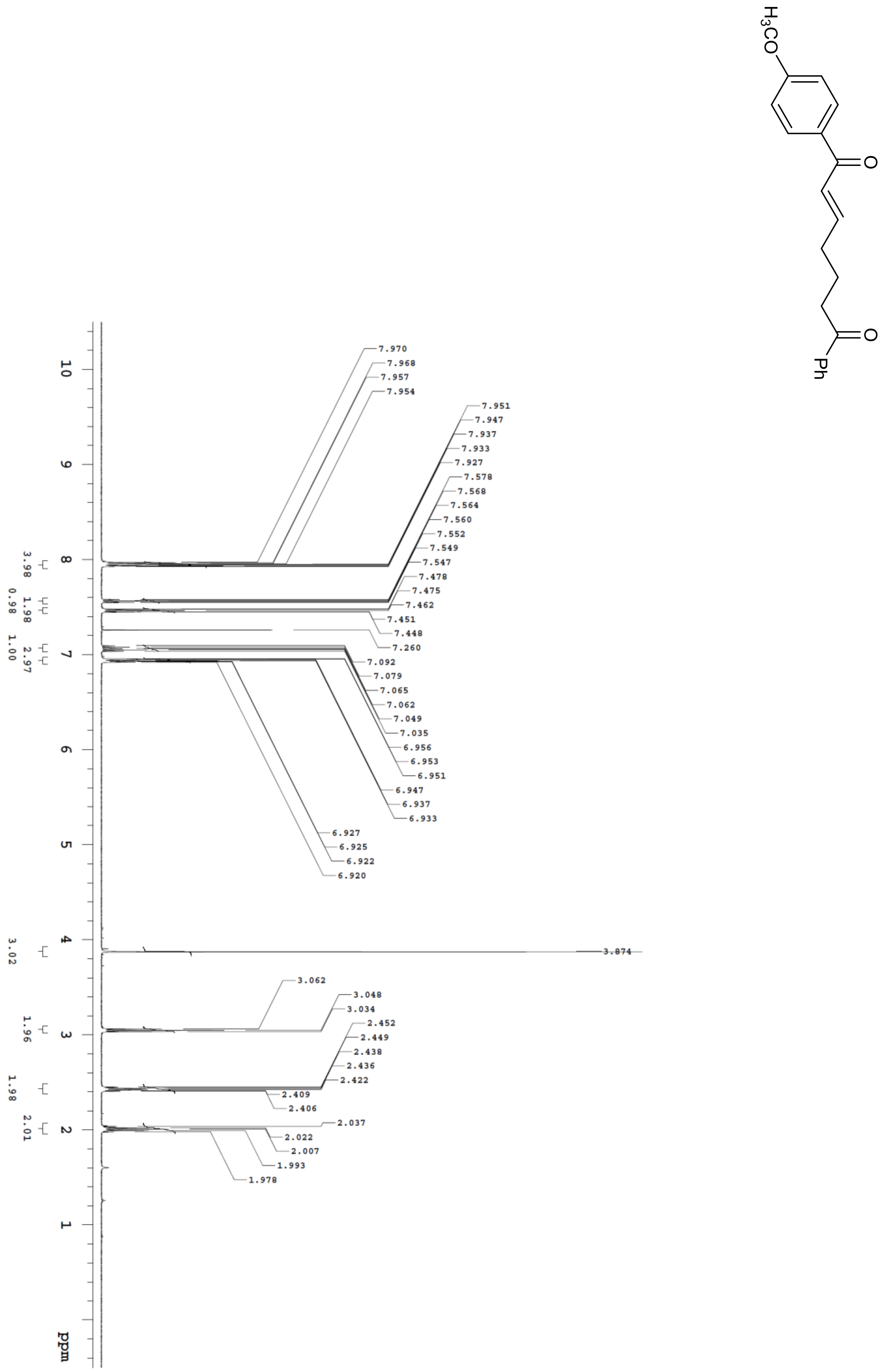

Supplementary Figure 12. ^{1}H NMR spectrum of (*E*)-1-(4-methoxyphenyl)-7-phenylhept-2-ene-1,7-dione (**1b**).

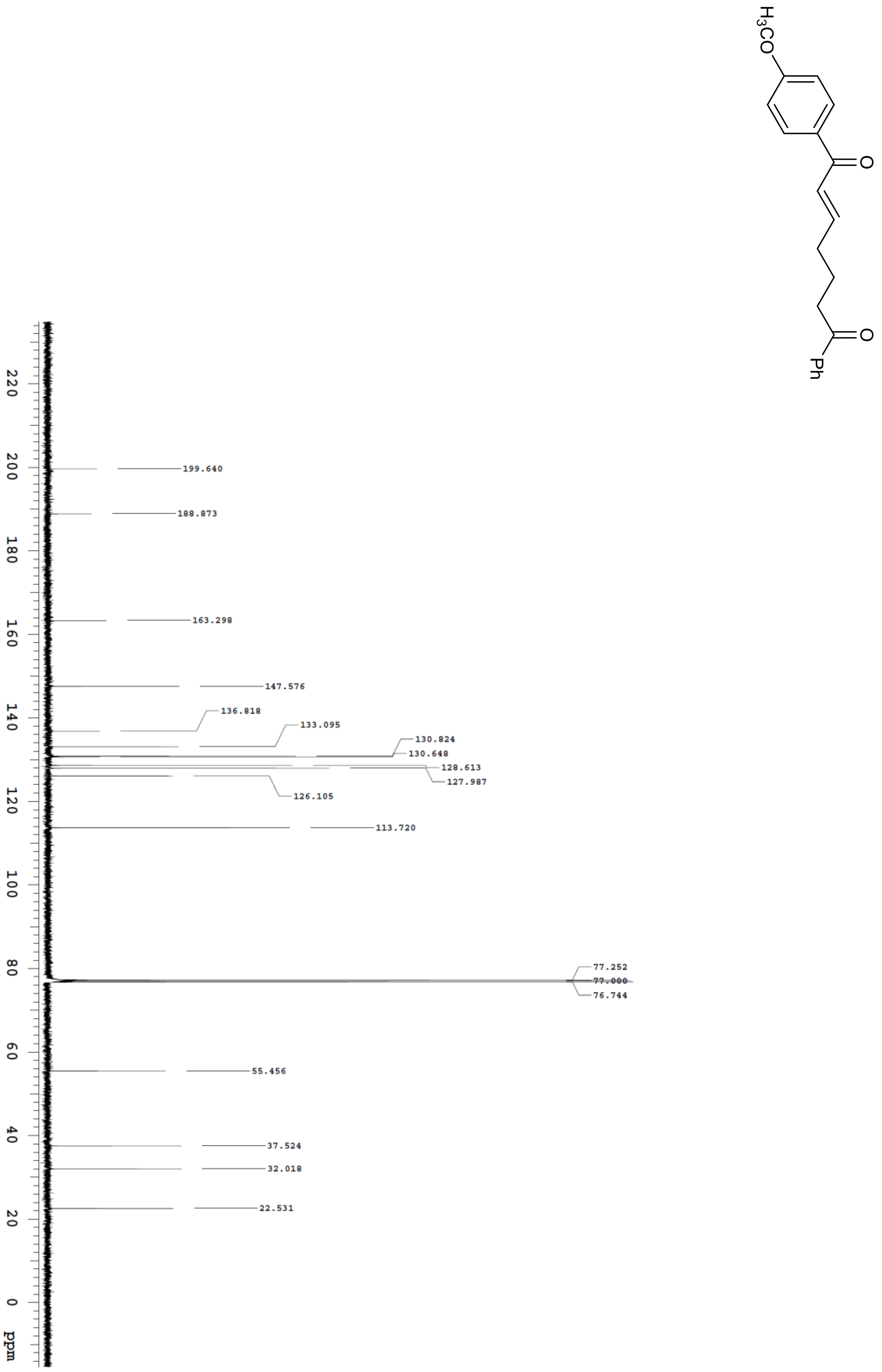

Supplementary Figure 13. ^{13}C NMR spectrum of (*E*)-1-(4-methoxyphenyl)-7-phenylhept-2-ene-1,7-dione (**1b**).

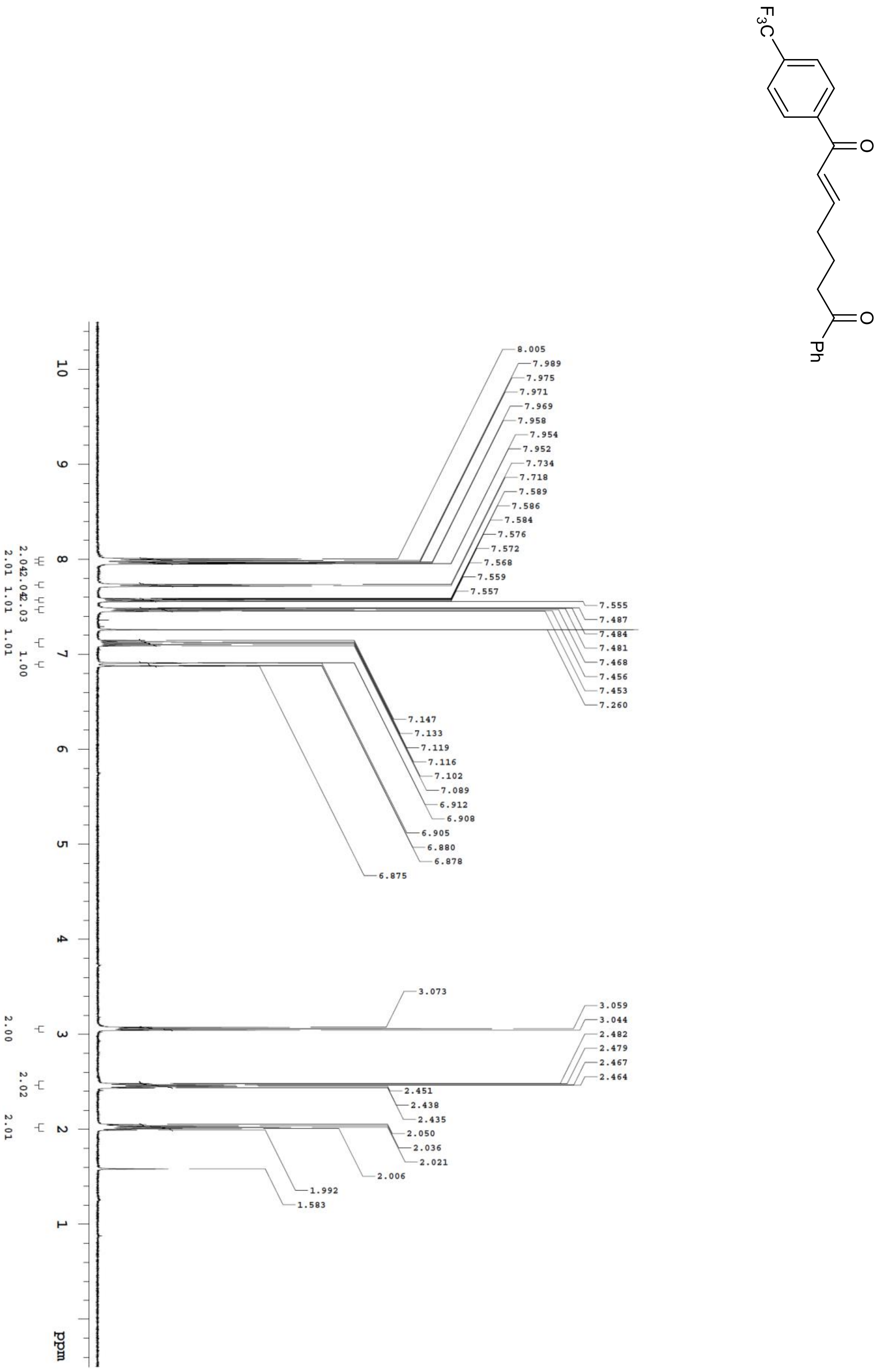

Supplementary Figure 14. ^{1}H NMR spectrum of (*E*)-7-phenyl-1-(4-(trifluoromethyl)phenyl)hept-2-ene-1,7-dione (**1c**).

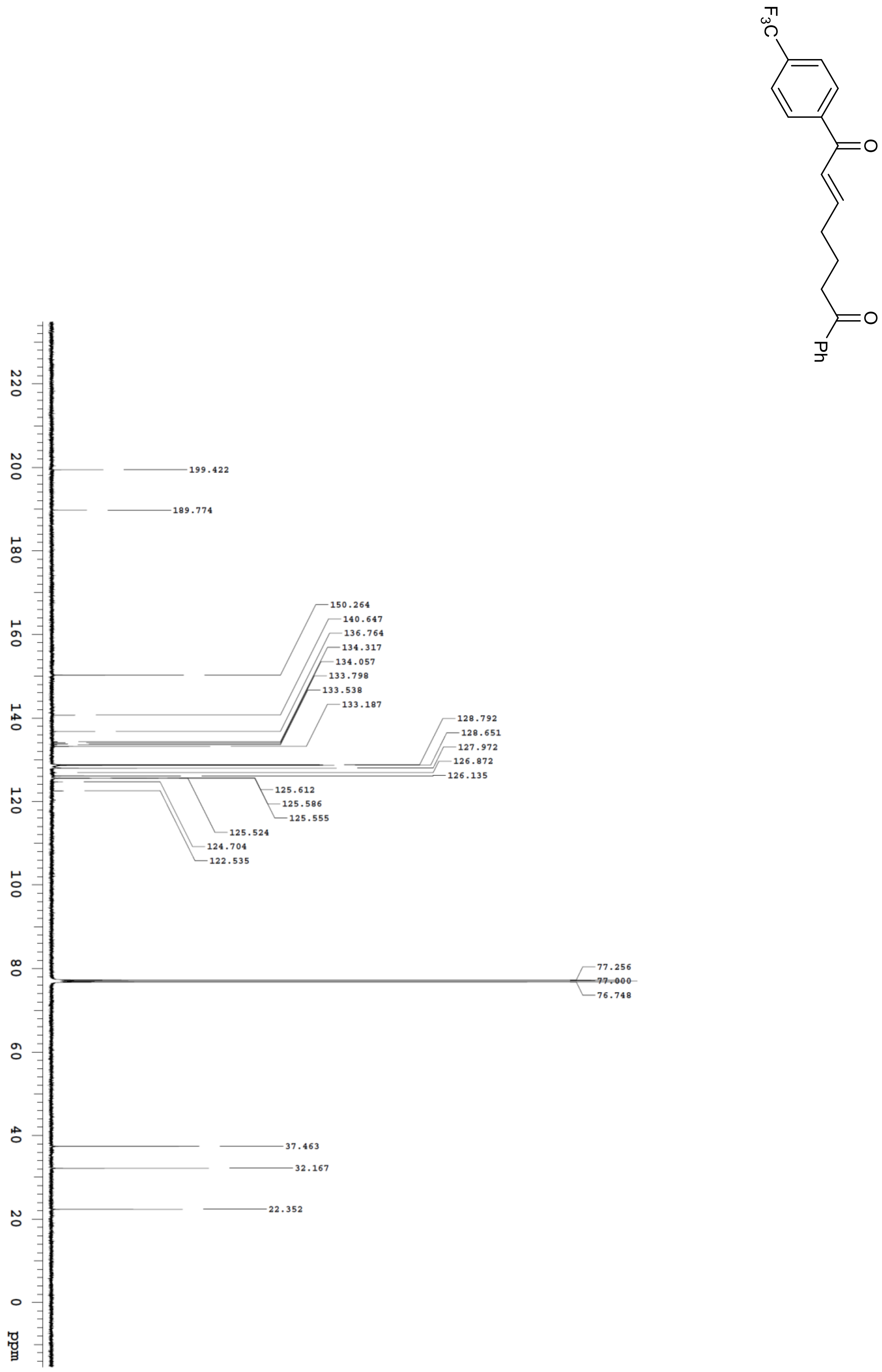

Supplementary Figure 15. ^{13}C NMR spectrum of (*E*)-7-phenyl-1-(4-(trifluoromethyl)phenyl)hept-2-ene-1,7-dione (**1c**).

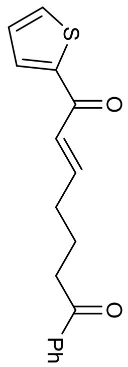

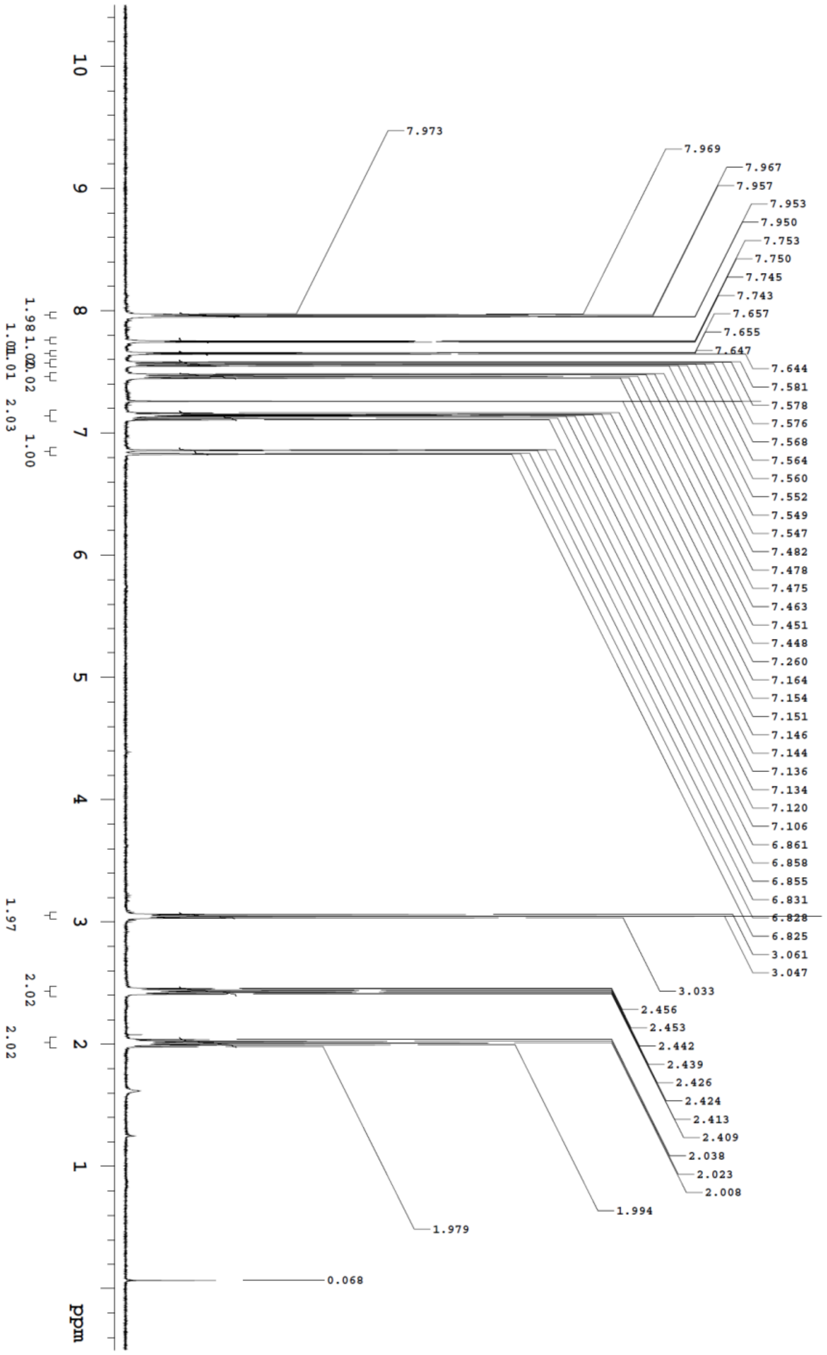

Supplementary Figure 16. ^{1}H NMR spectrum of (*E*)-7-phenyl-1-(thiophen-2-yl)hept-2-ene-1,7-dione (**1d**).

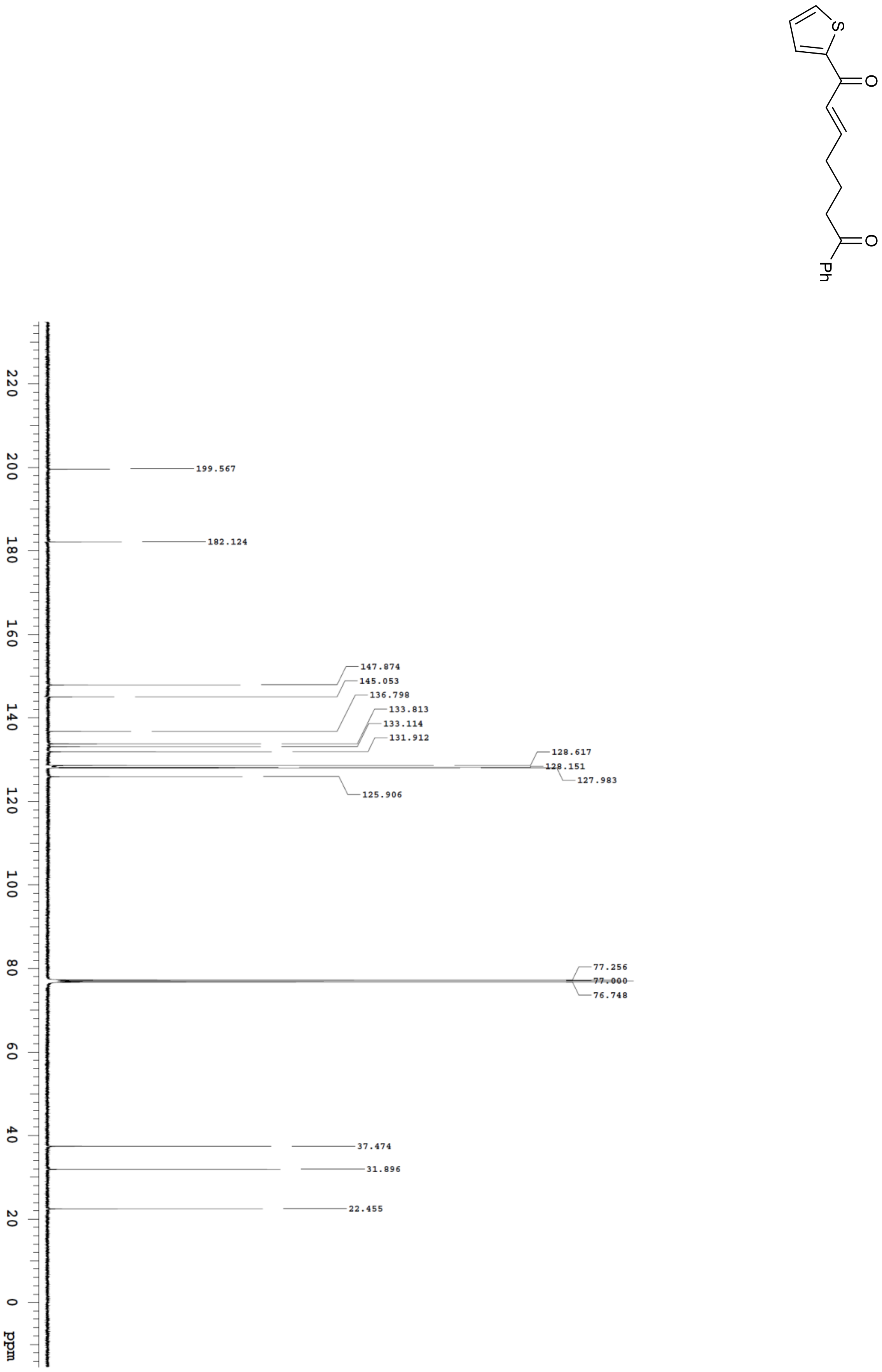

Supplementary Figure 17. ^{13}C NMR spectrum of (*E*)-7-phenyl-1-(thiophen-2-yl)hept-2-ene-1,7-dione (**1d**).

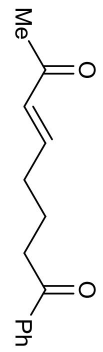

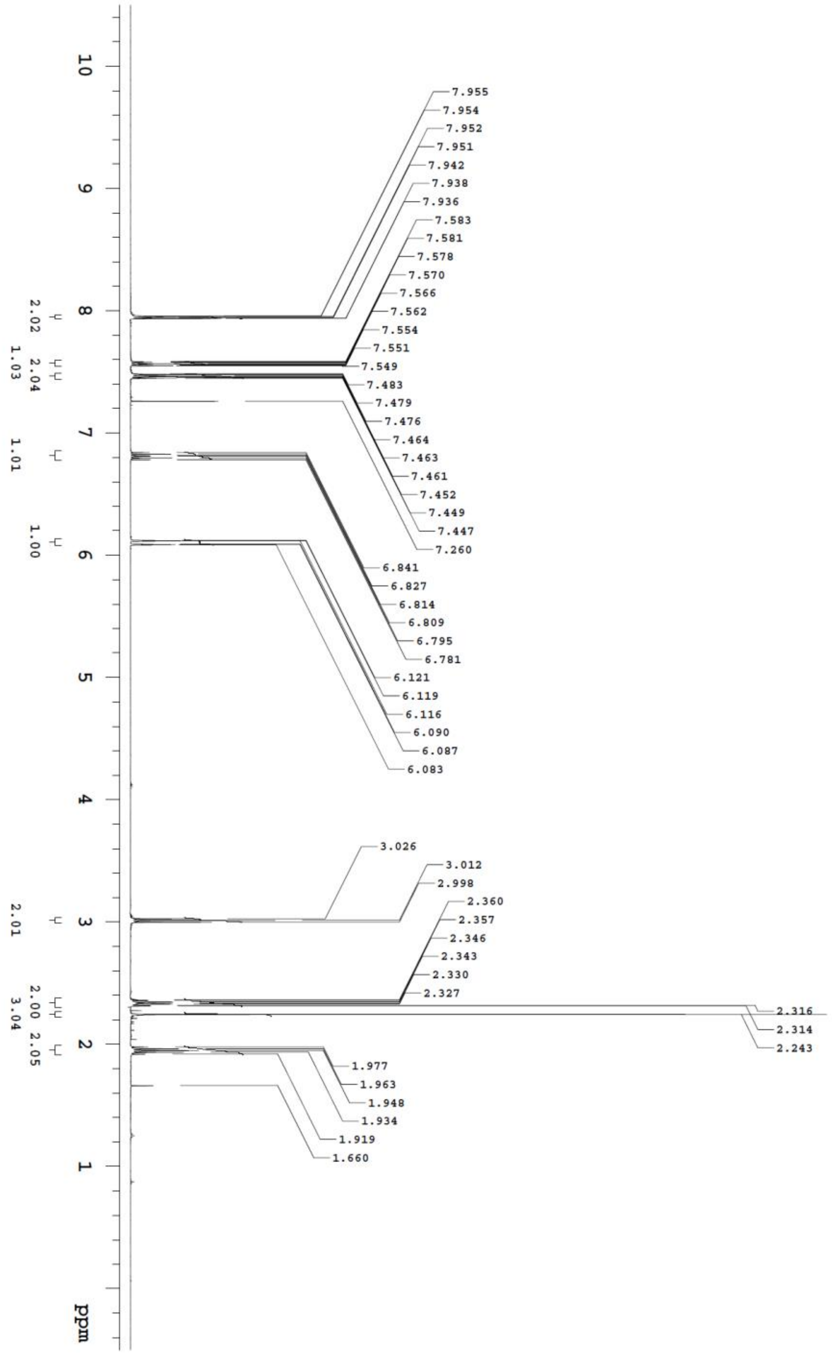

Supplementary Figure 18. ^{1}H NMR spectrum of (*E*)-1-phenyloct-5-ene-1,7-dione (**1e**).

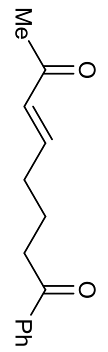

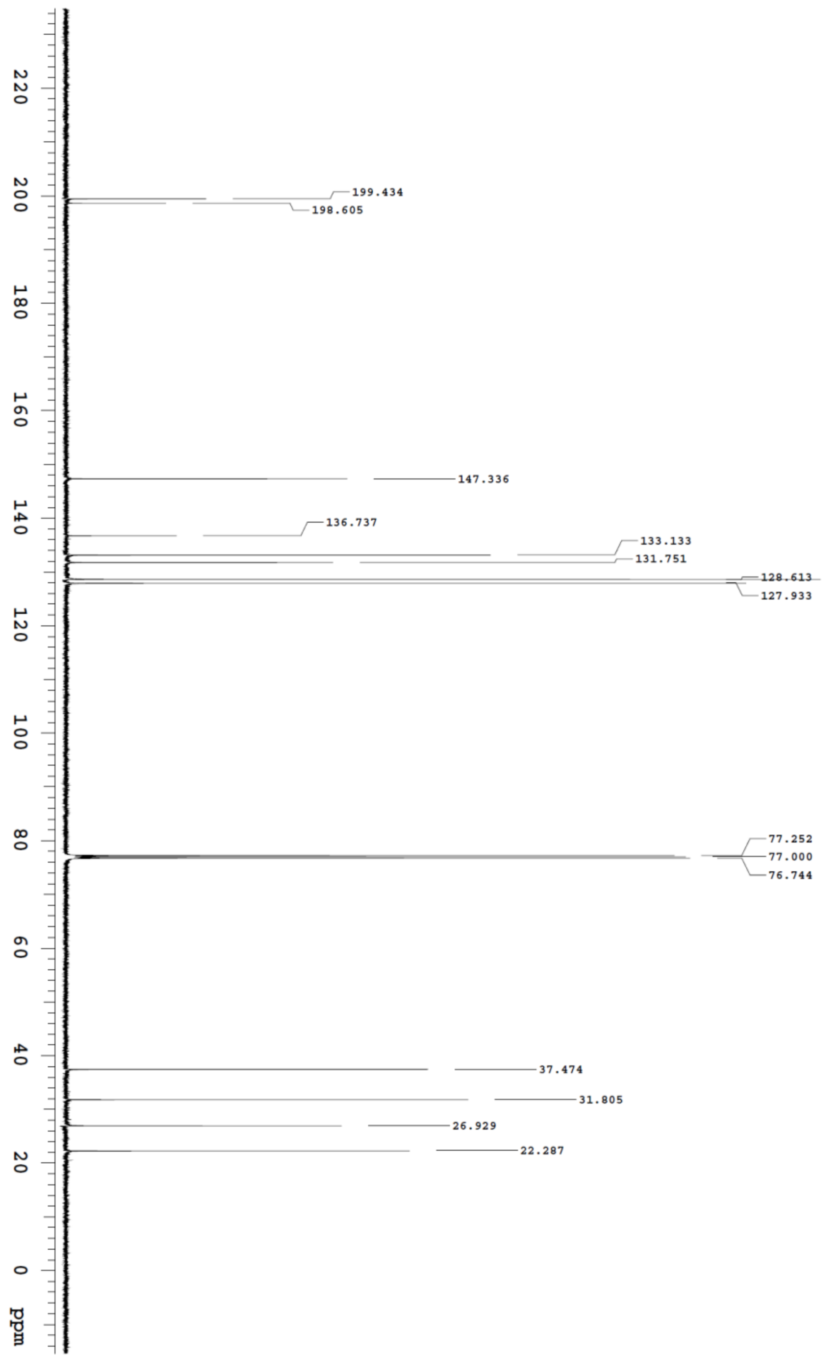

Supplementary Figure 19. ^{13}C NMR spectrum of (*E*)-1-phenyloct-5-ene-1,7-dione (**1e**).

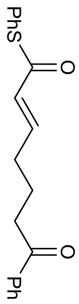

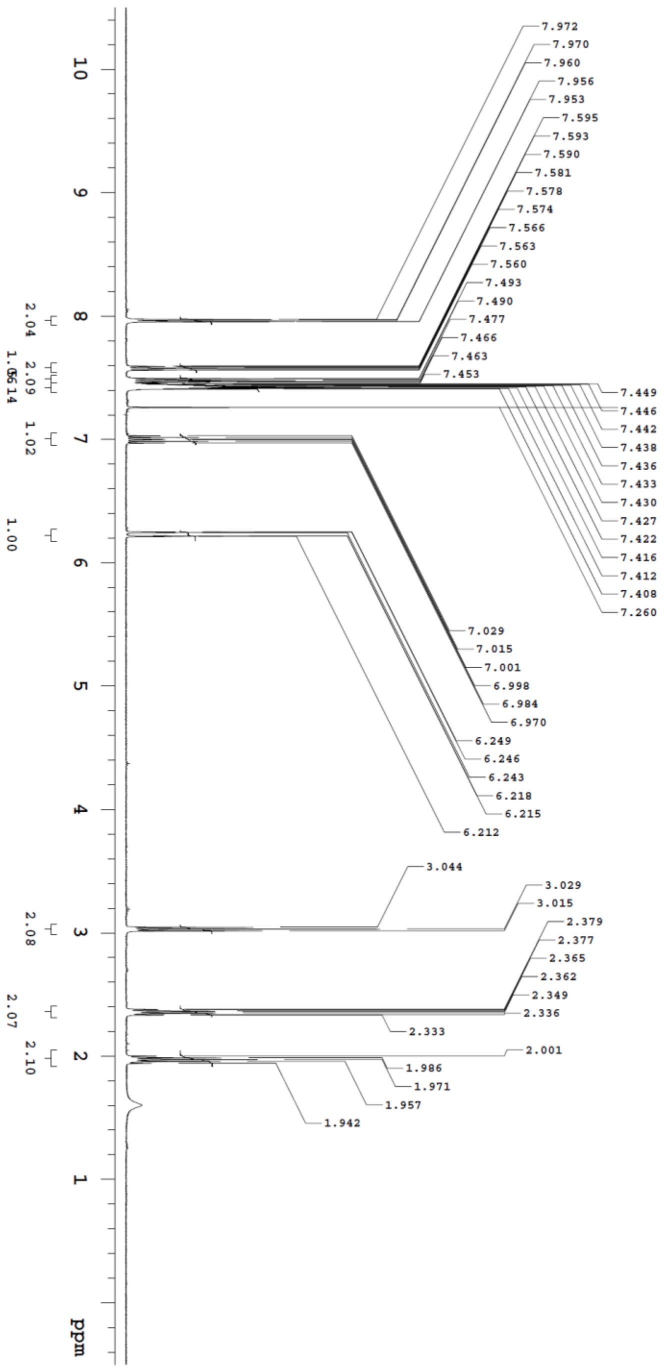

Supplementary Figure 20. ^{1}H NMR spectrum of *S*-phenyl (*E*)-7-oxo-7-phenylhept-2-enethioate (**1f**).

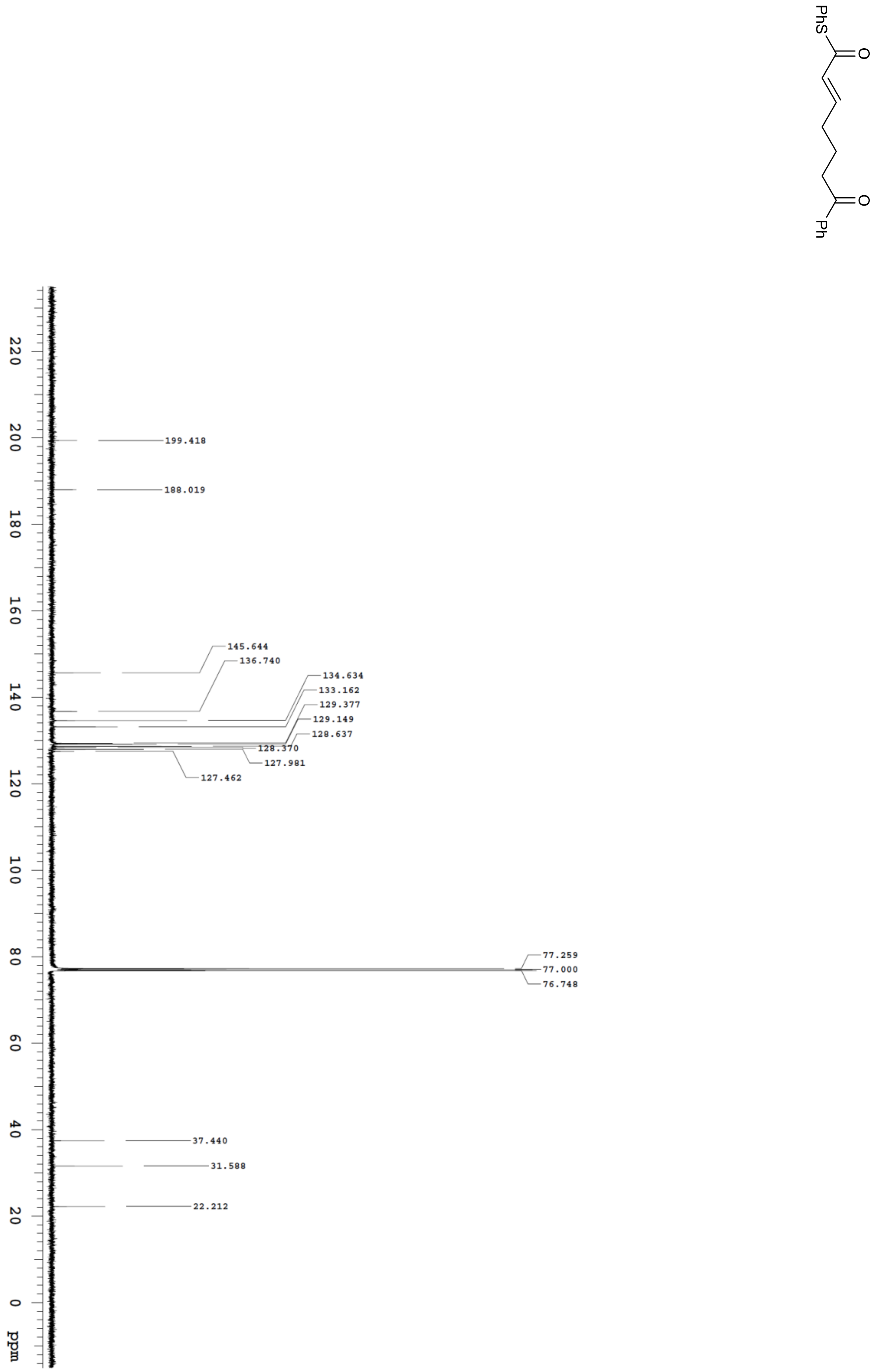

Supplementary Figure 21. ^{13}C NMR spectrum of *S*-phenyl (*E*)-7-oxo-7-phenylhept-2-enethioate (**1f**).

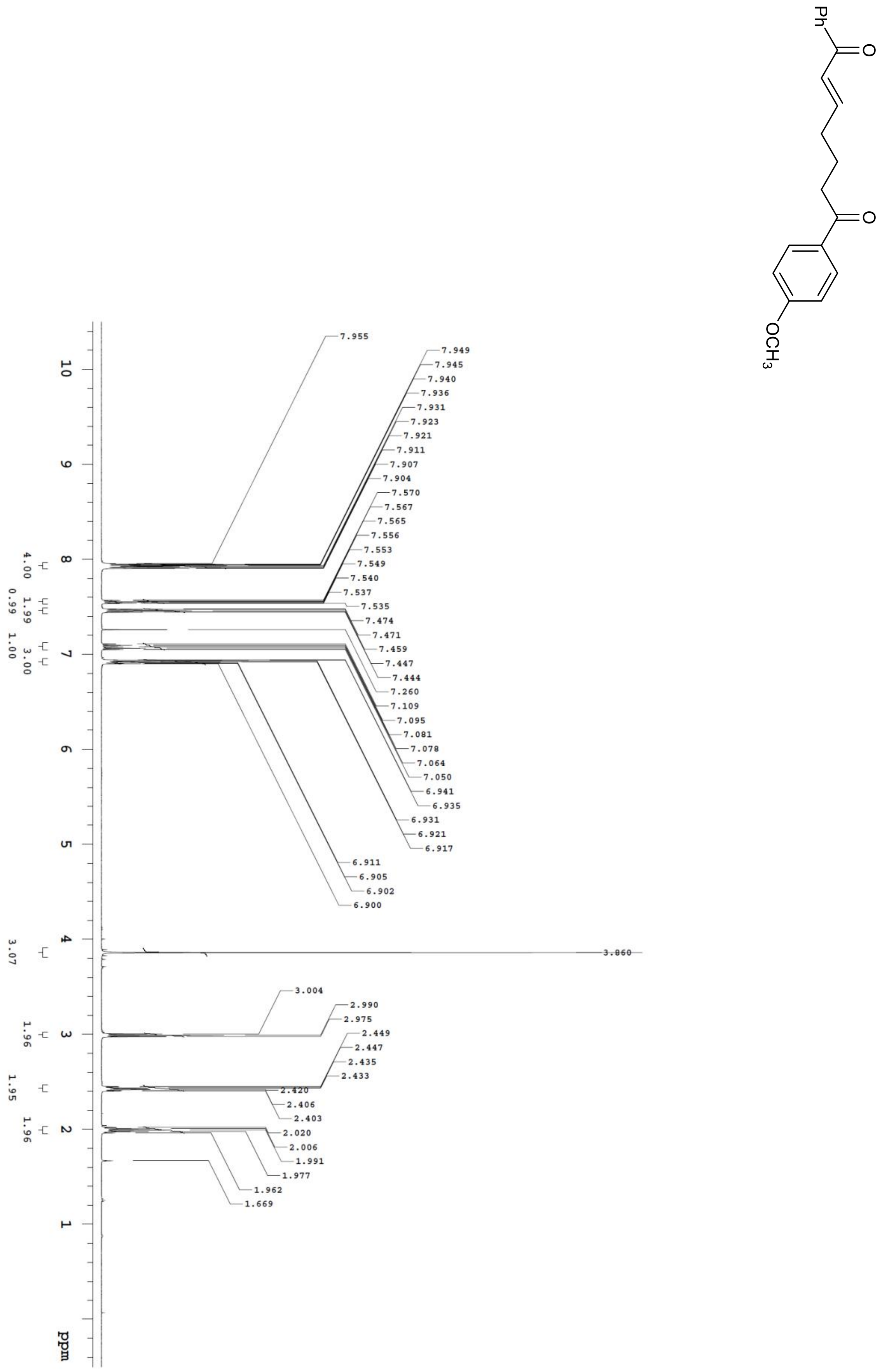

Supplementary Figure 22. ^{1}H NMR spectrum of (*E*)-7-(4-methoxyphenyl)-1-phenylhept-2-ene-1,7-dione (**1g**).

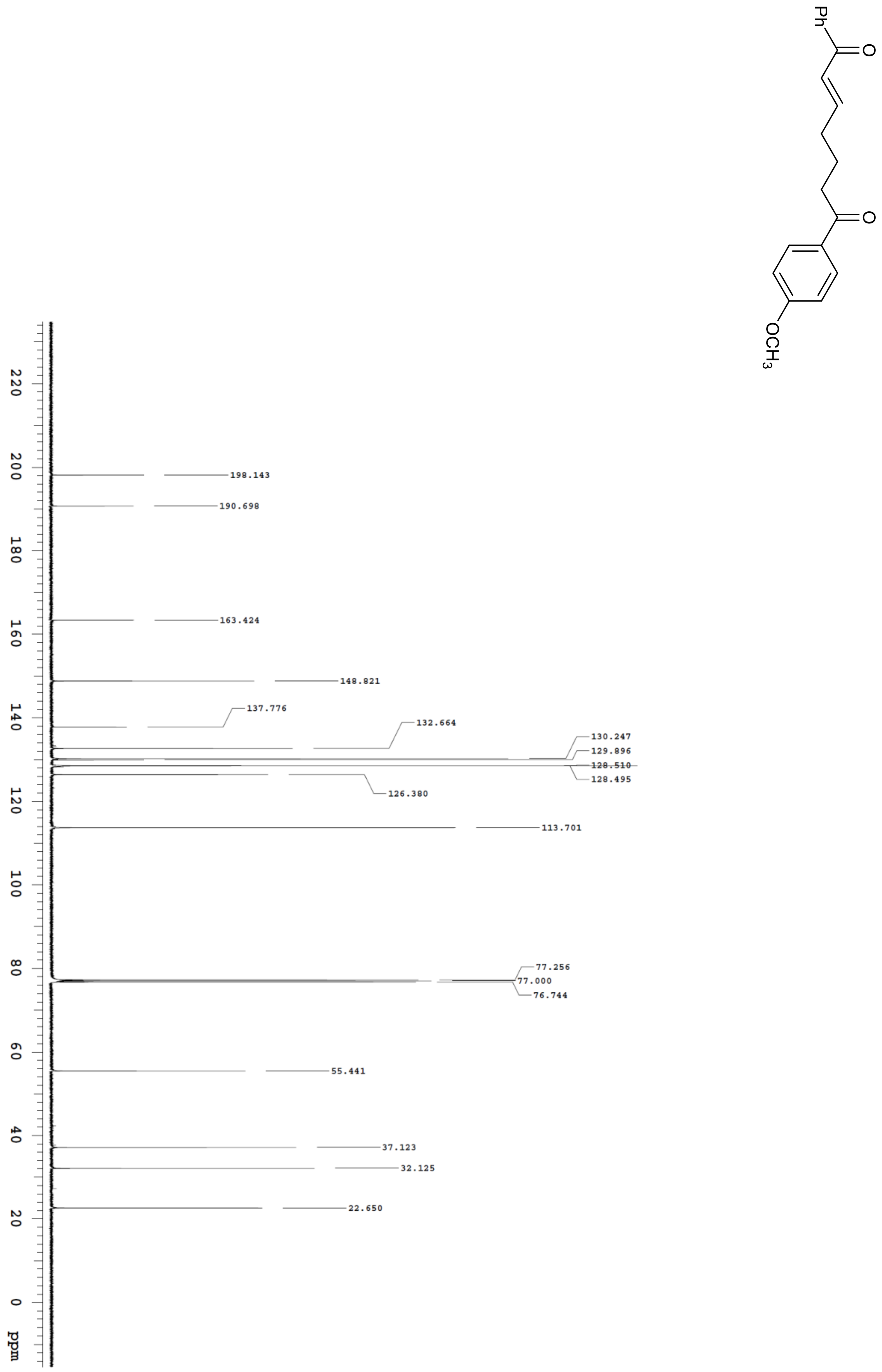

Supplementary Figure 23. ^{13}C NMR spectrum of (*E*)-7-(4-methoxyphenyl)-1-phenylhept-2-ene-1,7-dione (**1g**).

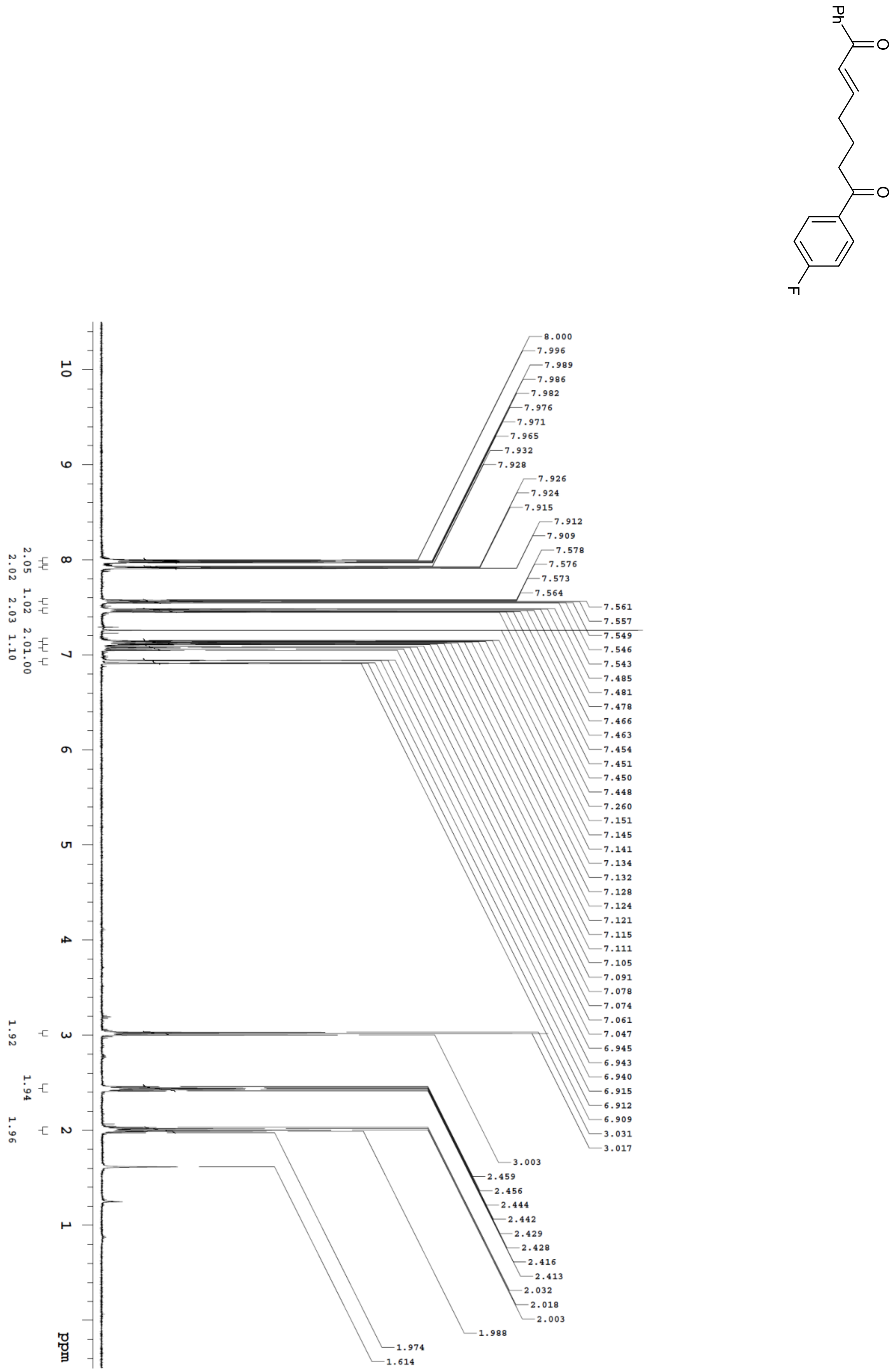

Supplementary Figure 24. ^{1}H NMR spectrum of (*E*)-7-(4-fluorophenyl)-1-phenylhept-2-ene-1,7-dione (**1h**).

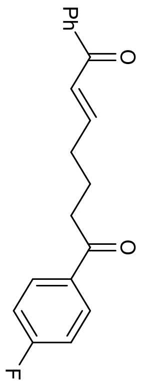

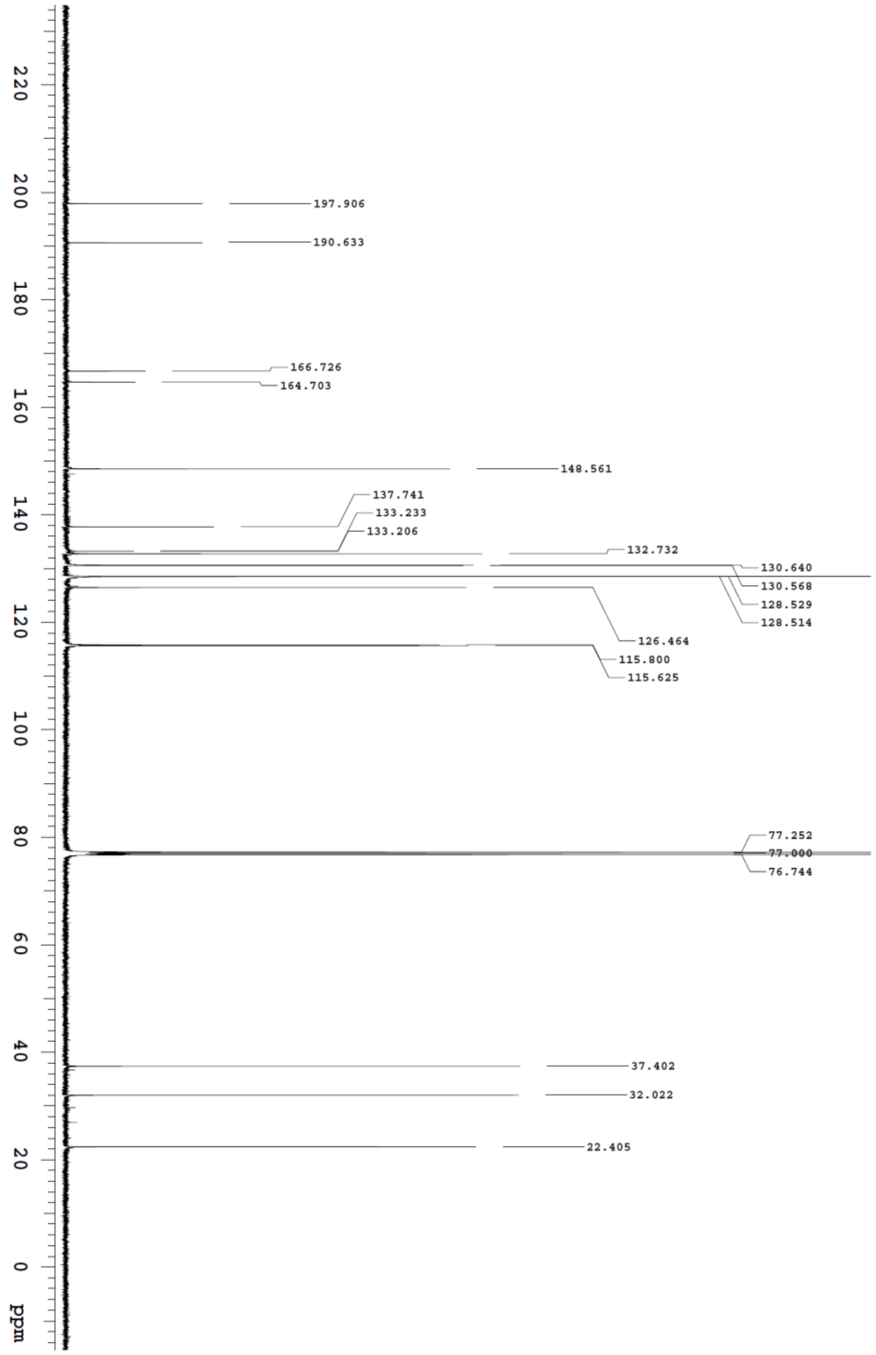

Supplementary Figure 25. ^{13}C NMR spectrum of (*E*)-7-(4-fluorophenyl)-1-phenylhept-2-ene-1,7-dione (**1h**).

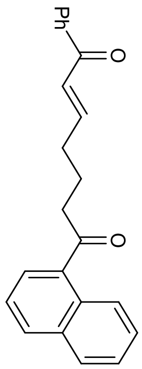

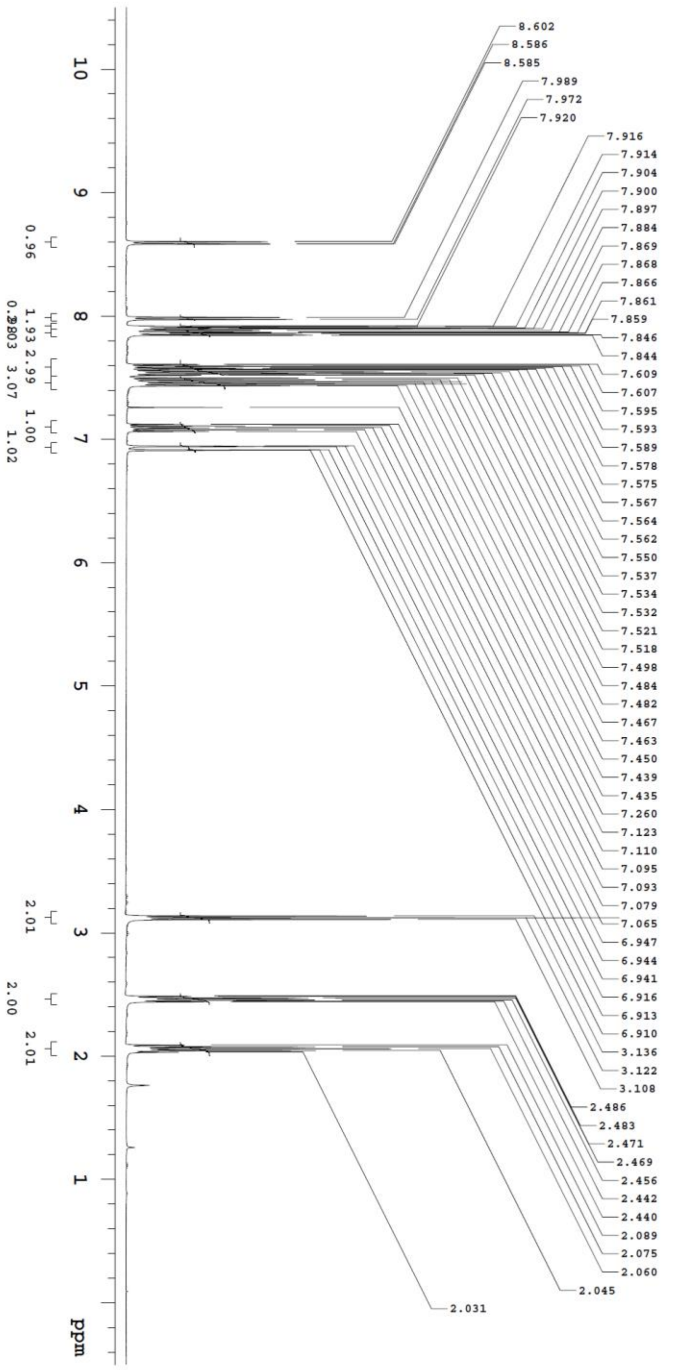

Supplementary Figure 26. ^{1}H NMR spectrum of (*E*)-7-(naphthalen-1-yl)-1-phenylhept-2-ene-1,7-dione (**1i**).

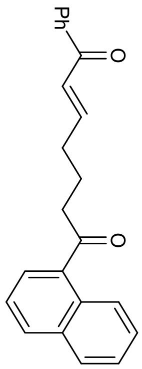

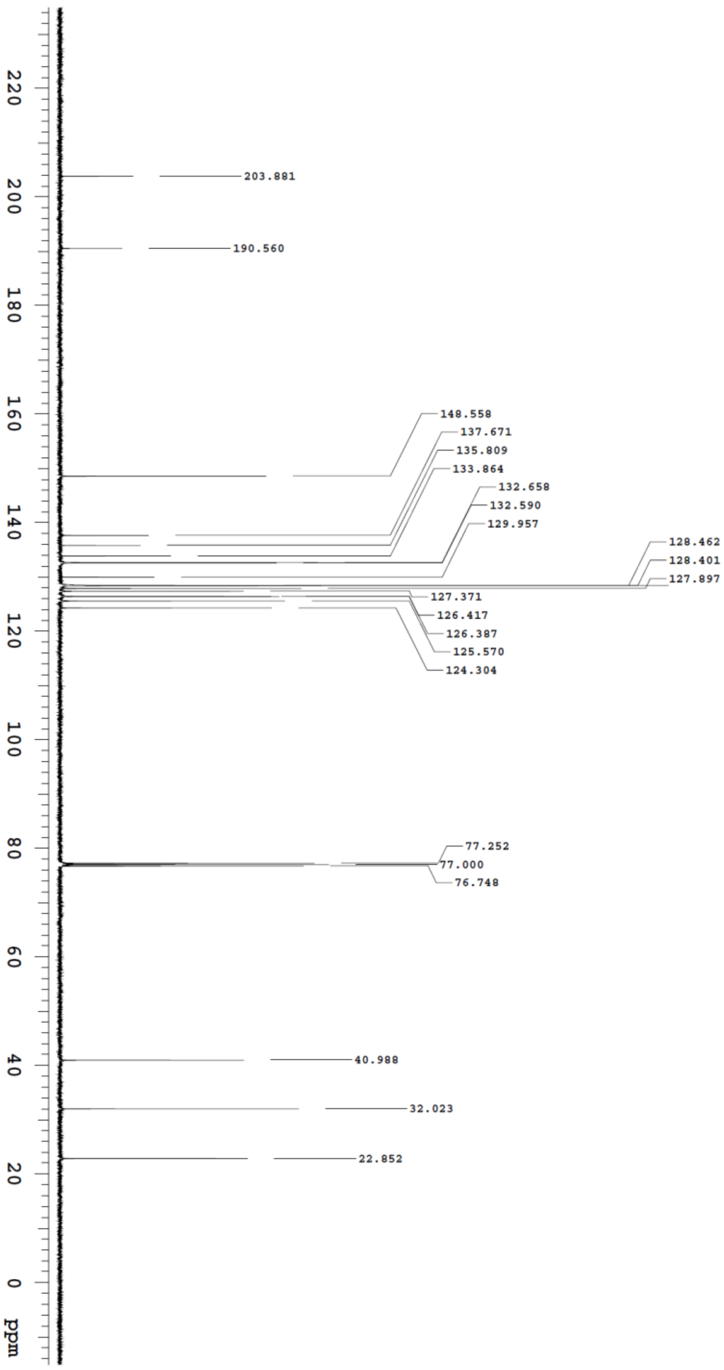

Supplementary Figure 27. ^{13}C NMR spectrum of (*E*)-7-(naphthalen-1-yl)-1-phenylhept-2-ene-1,7-dione (**1i**).

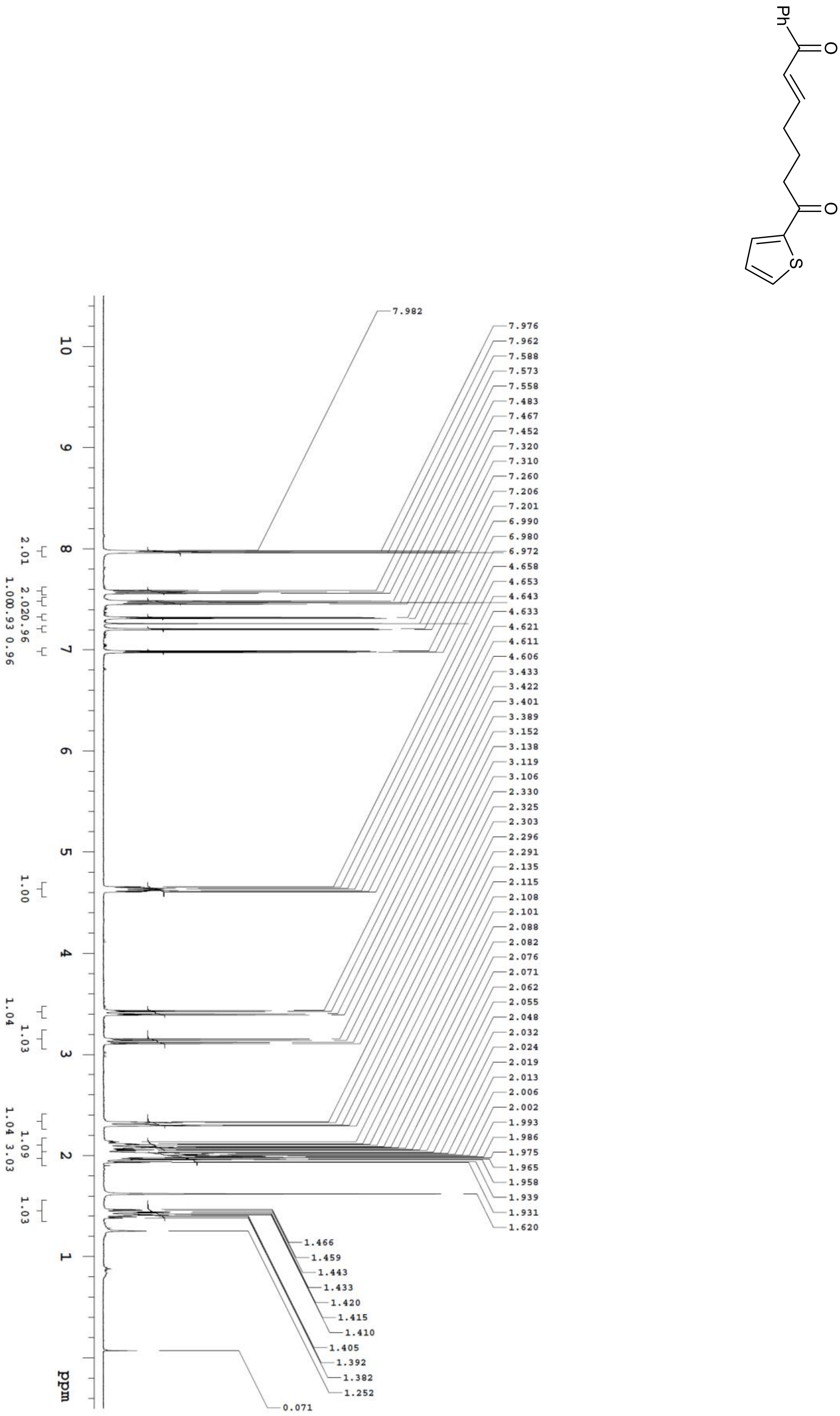

Supplementary Figure 28. ^{1}H NMR spectrum of (*E*)-1-phenyl-7-(thiophen-2-yl)hept-2-ene-1,7-dione (**1j**).

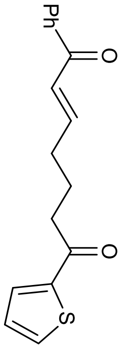

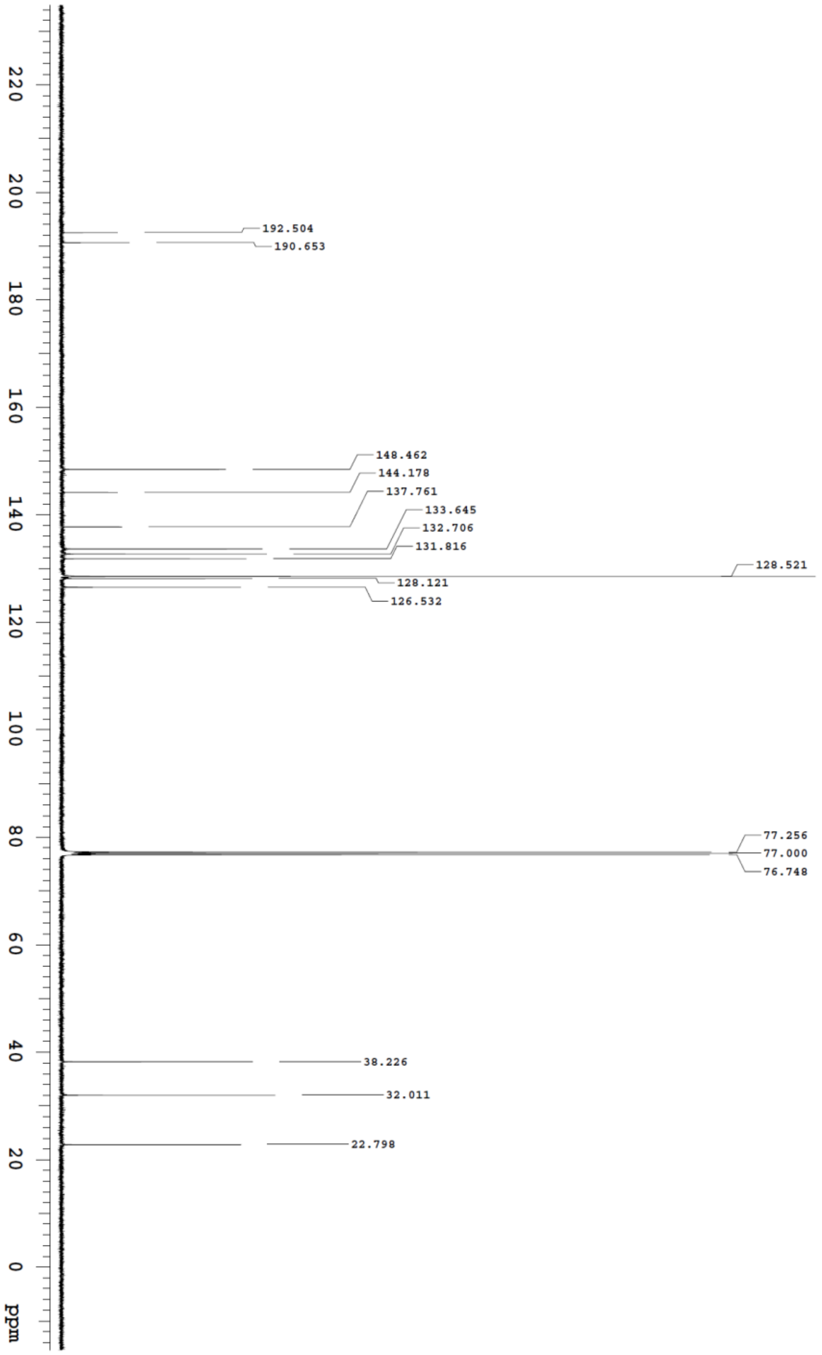

Supplementary Figure 29. ^{13}C NMR spectrum of (*E*)-1-phenyl-7-(thiophen-2-yl)hept-2-ene-1,7-dione (**1j**).

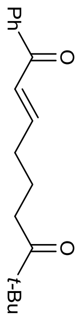

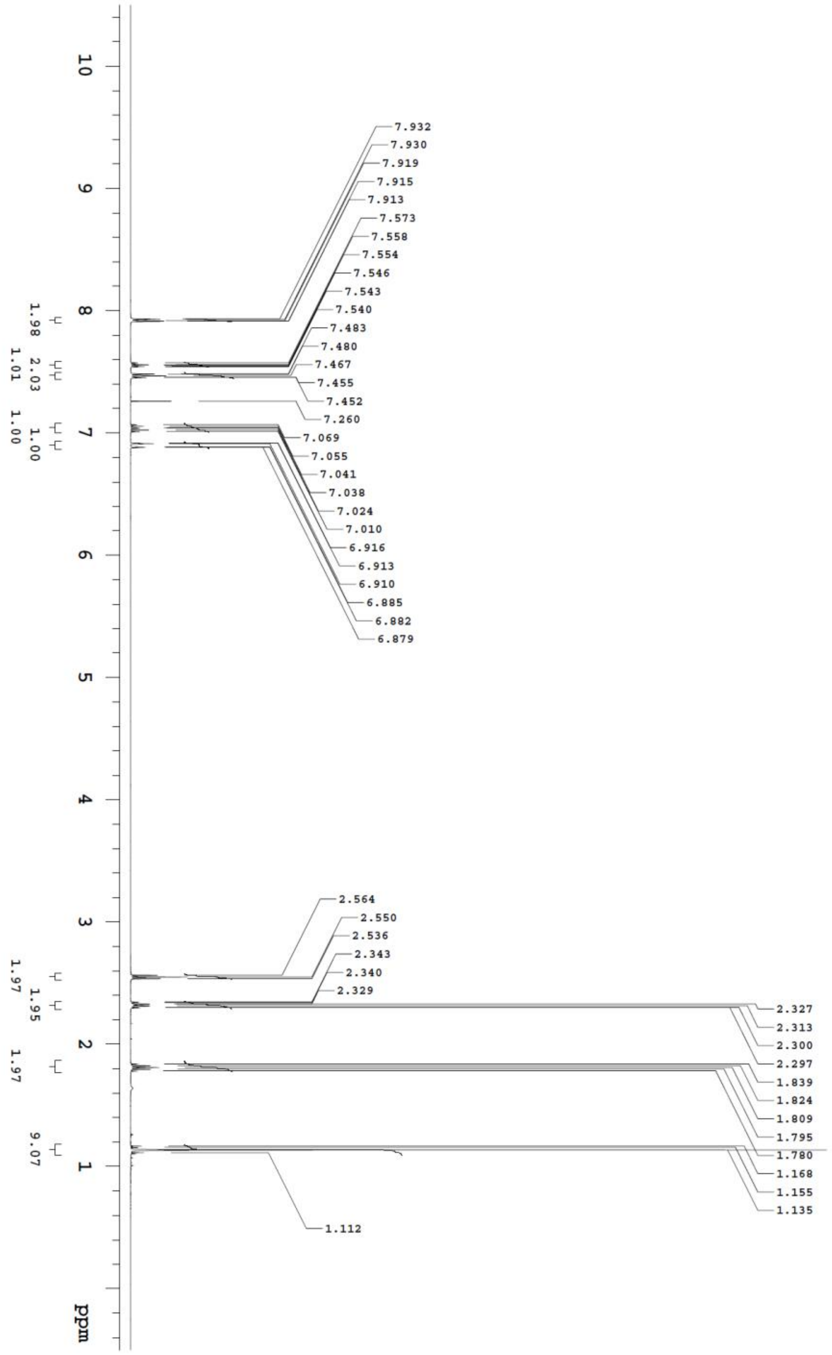

Supplementary Figure 30. ^{1}H NMR spectrum of (*E*)-8,8-dimethyl-1-phenylnon-2-ene-1,7-dione (**1k**).

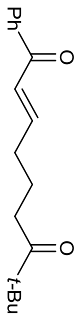

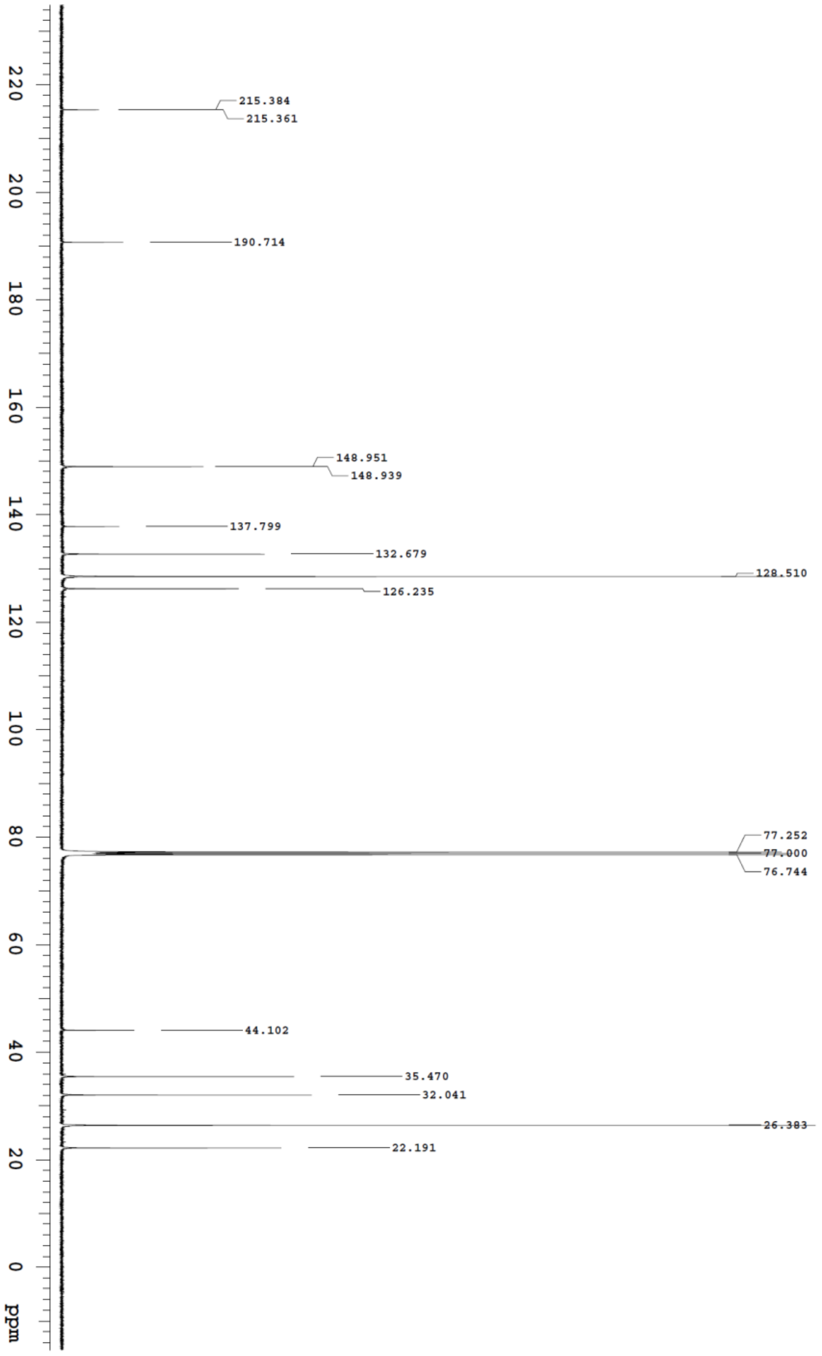

Supplementary Figure 31. ^{13}C NMR spectrum of (*E*)-8,8-dimethyl-1-phenylnon-2-ene-1,7-dione (**1k**).

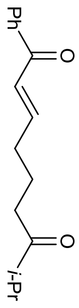

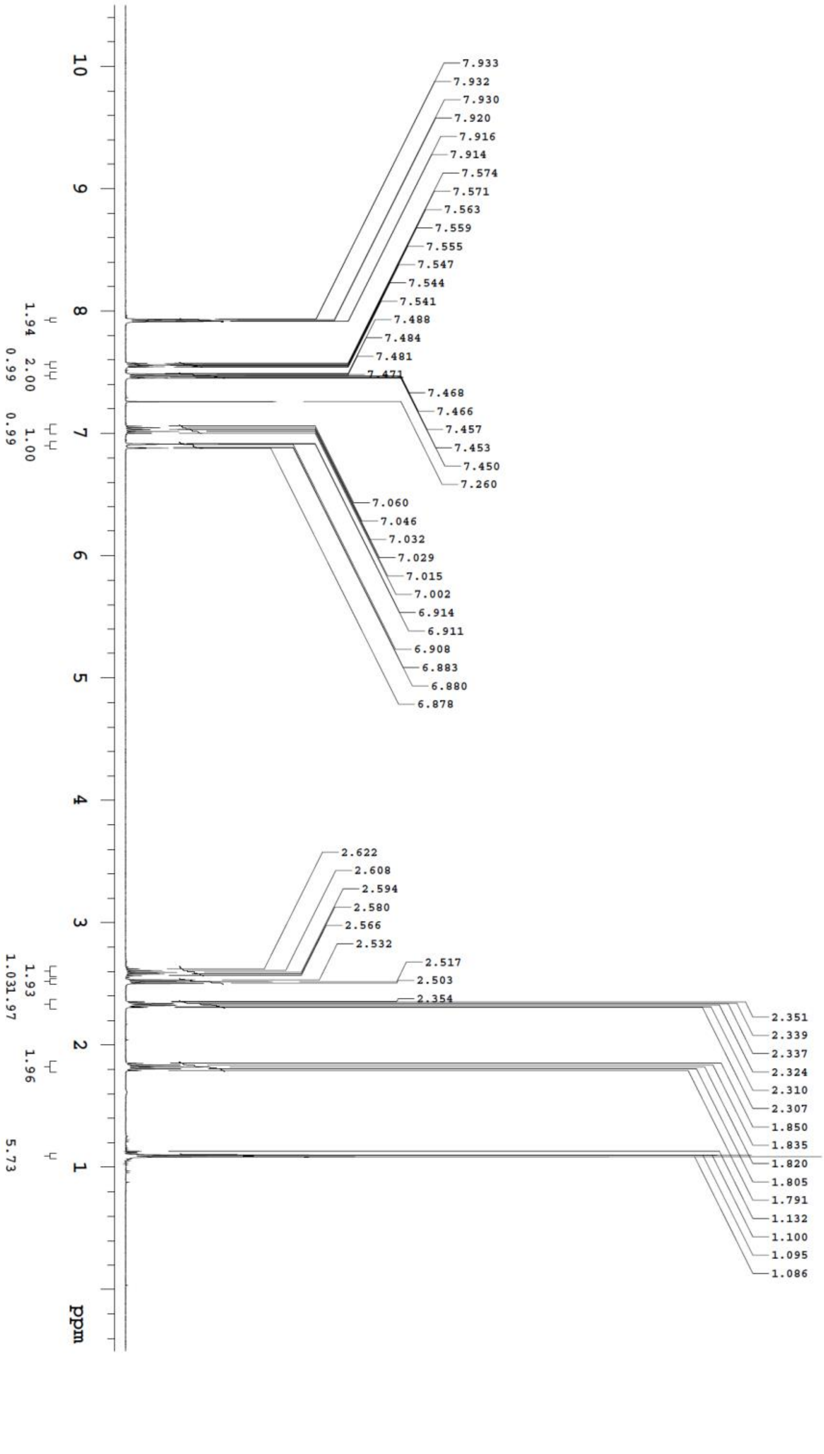

Supplementary Figure 32. ^{1}H NMR spectrum of (*E*)-8-methyl-1-phenylnon-2-ene-1,7-dione (**1l**).

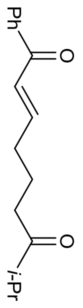

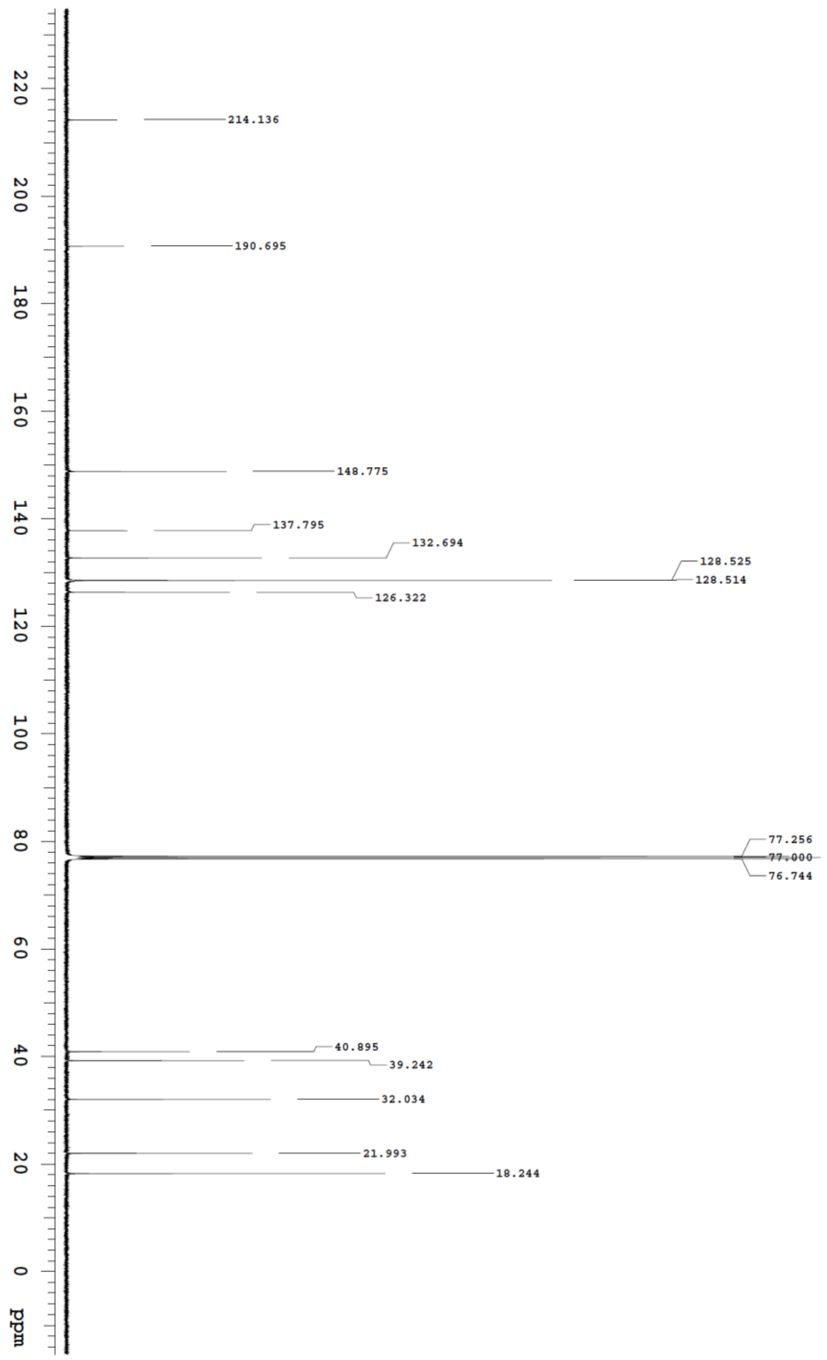

Supplementary Figure 33. ^{13}C NMR spectrum of (*E*)-8-methyl-1-phenylnon-2-ene-1,7-dione (**1l**).

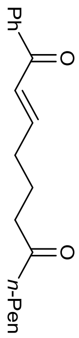

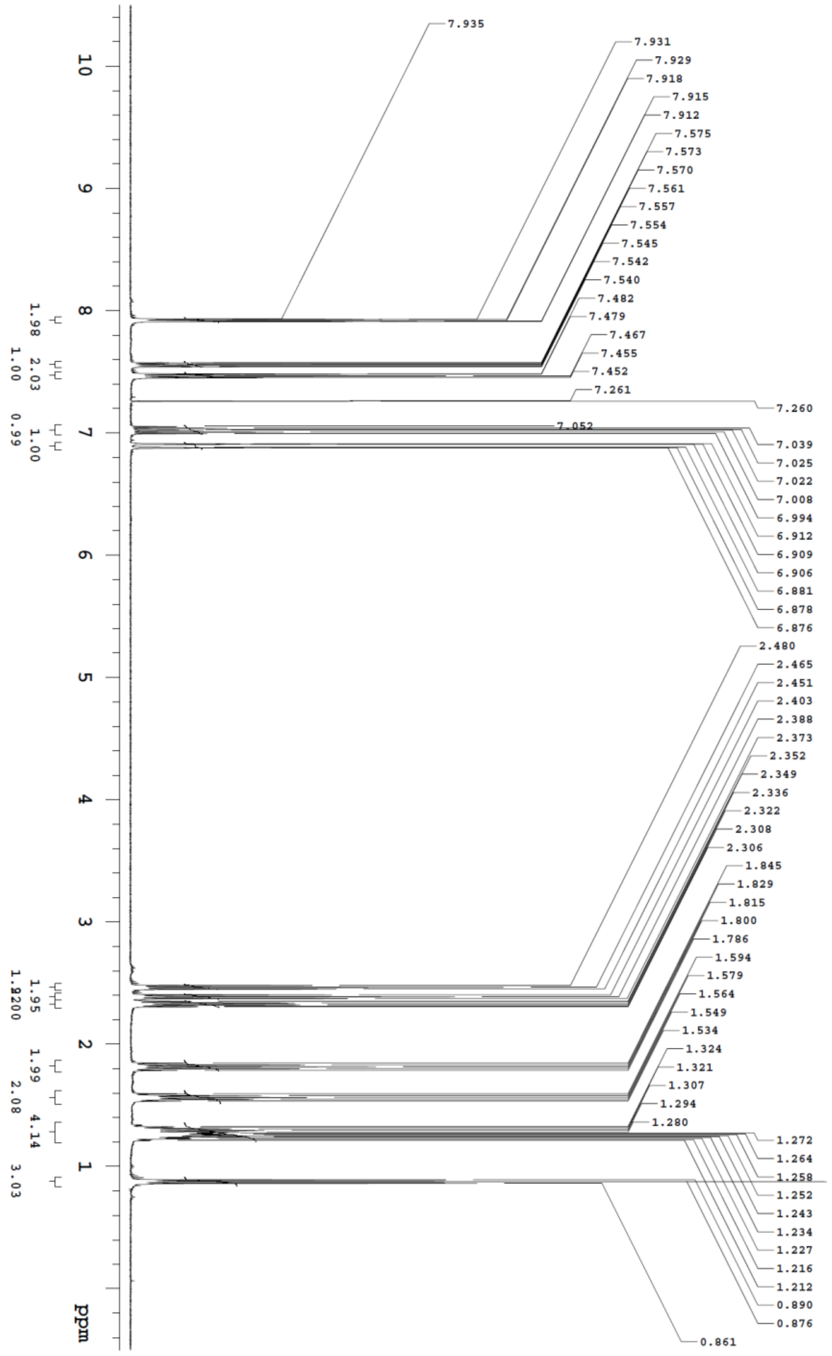

Supplementary Figure 34. ^{1}H NMR spectrum of (*E*)-1-phenyldodec-2-ene-1,7-dione (**1m**).

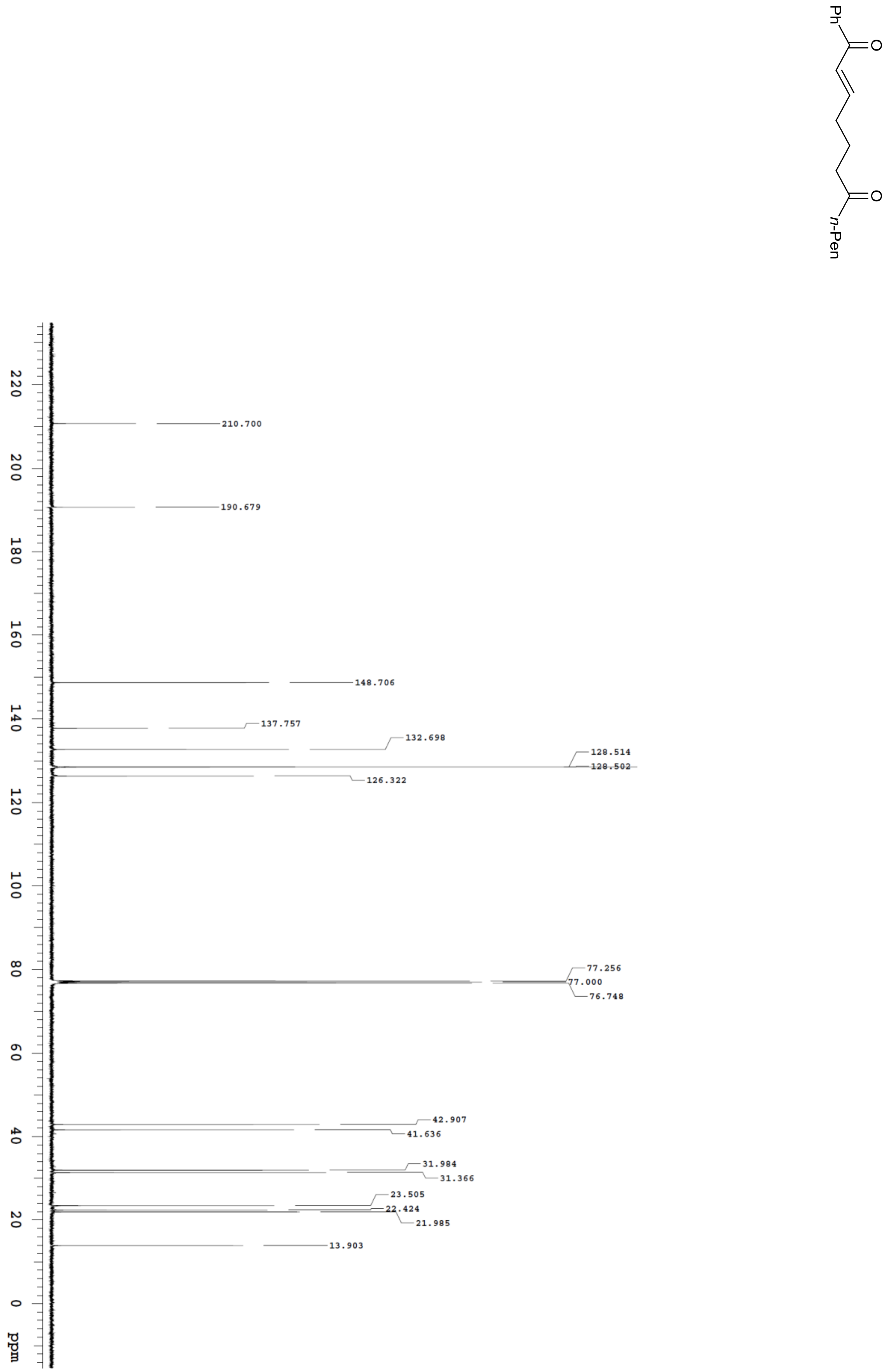

Supplementary Figure 35. ^{13}C NMR spectrum of (*E*)-1-phenyldodec-2-ene-1,7-dione (**1m**).

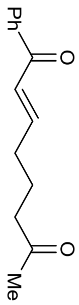

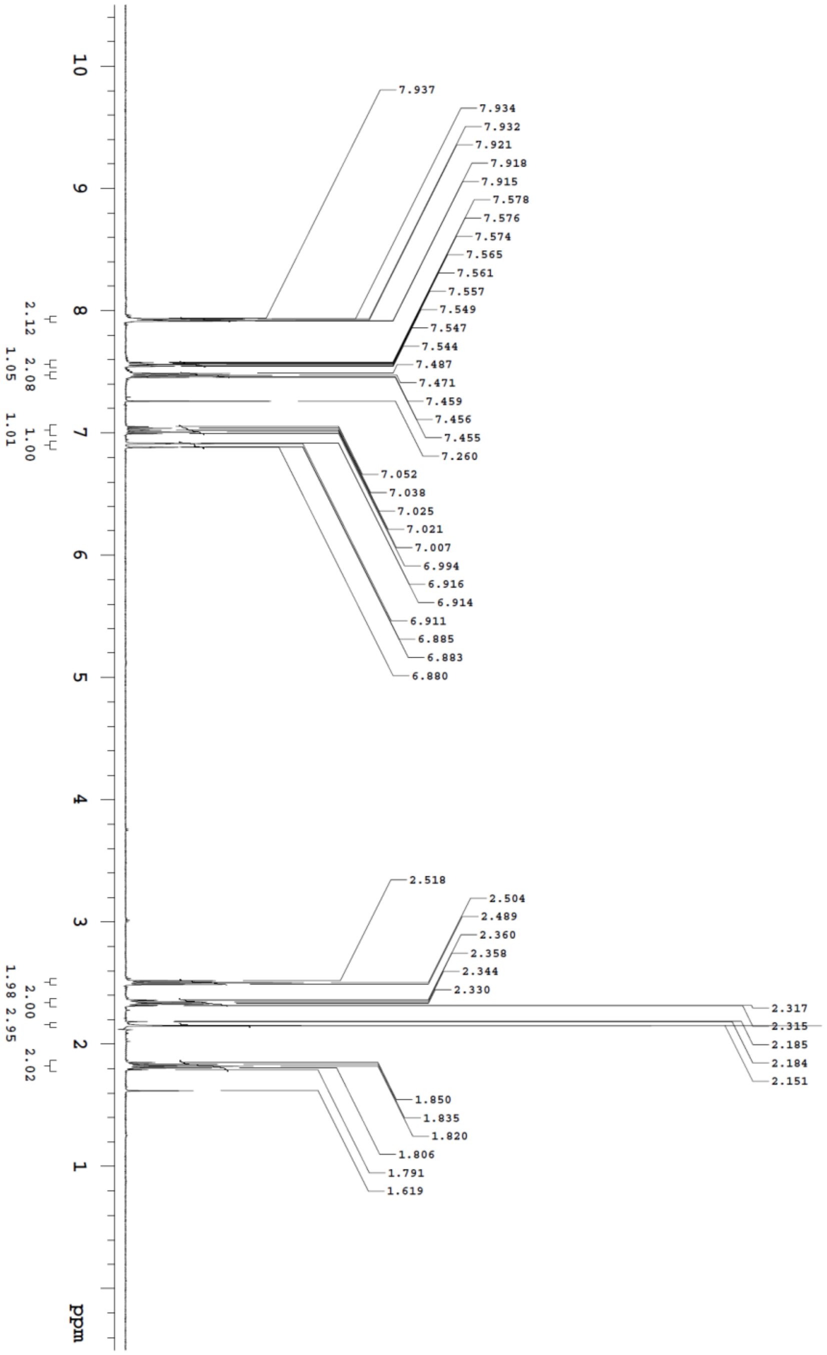

Supplementary Figure 36. ^{1}H NMR spectrum of (*E*)-1-phenyloct-2-ene-1,7-dione (**1n**).

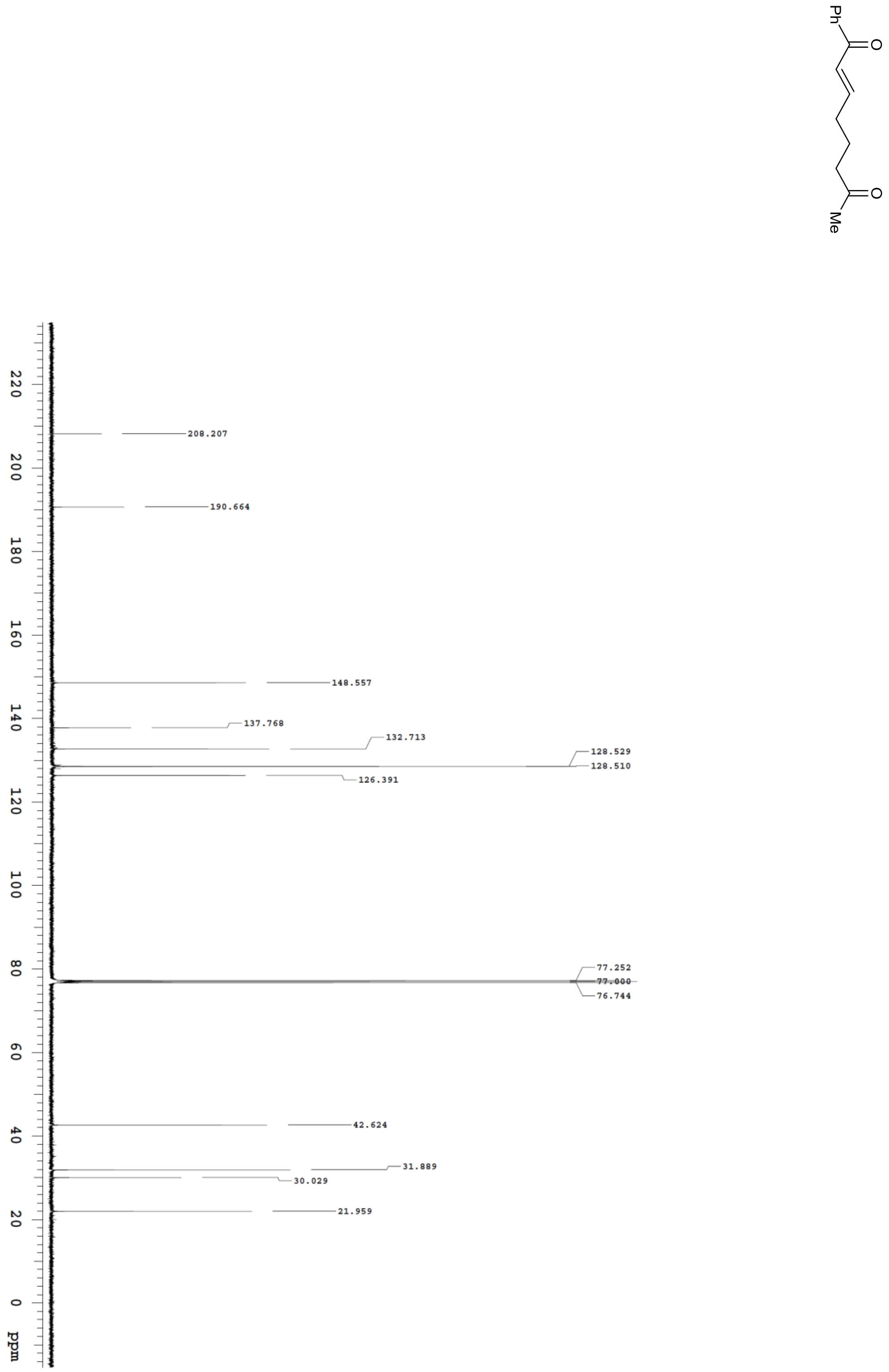

Supplementary Figure 37. ^{13}C NMR spectrum of (*E*)-1-phenyloct-2-ene-1,7-dione (**1n**).

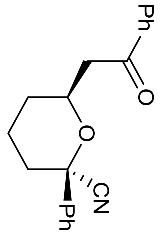

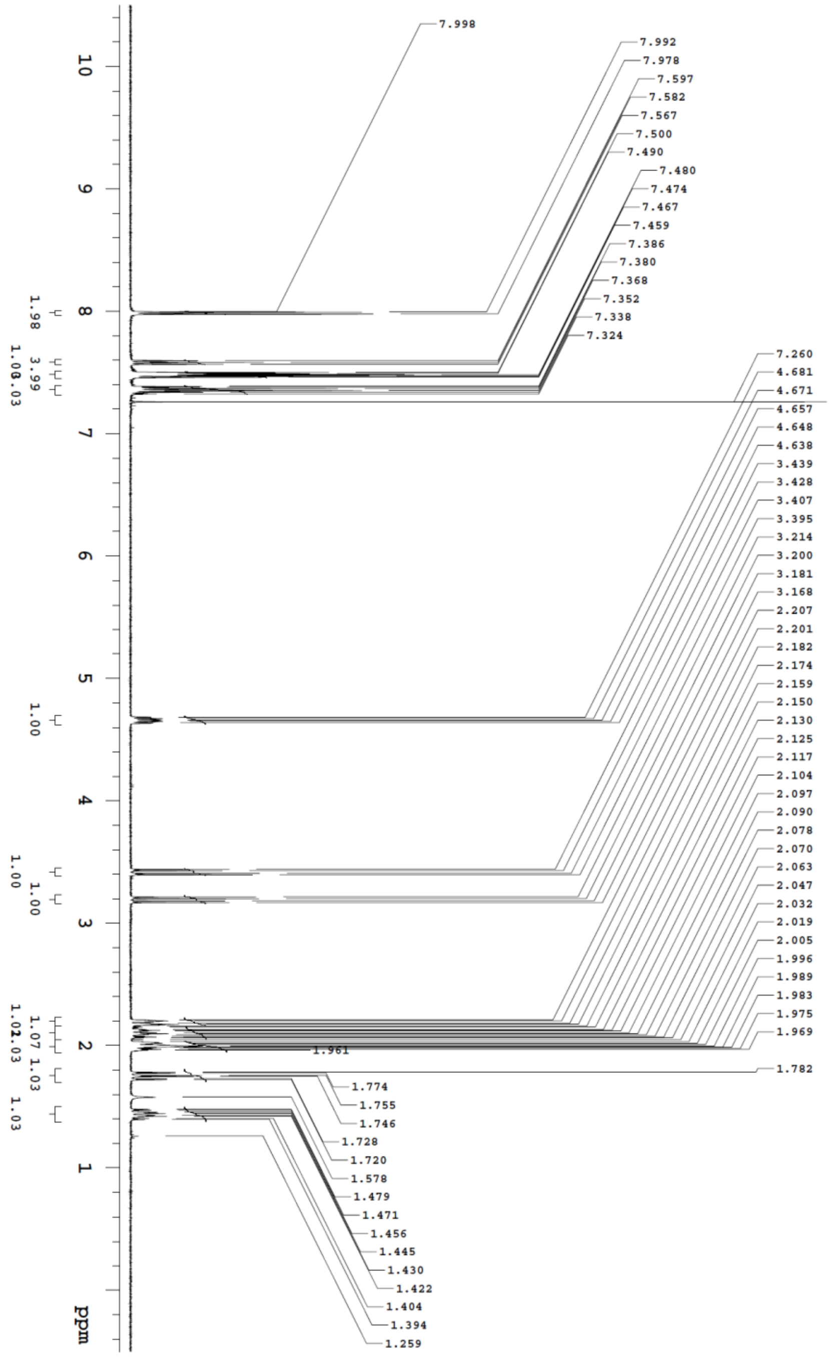

Supplementary Figure 38. ^{1}H NMR spectrum of (2*R*,6*S*)-6-(2-oxo-2-phenylethyl)-2-phenyltetrahydro-2*H*-pyran-2-carbonitrile (**3a**).

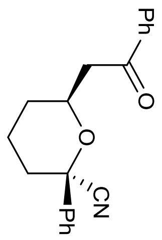

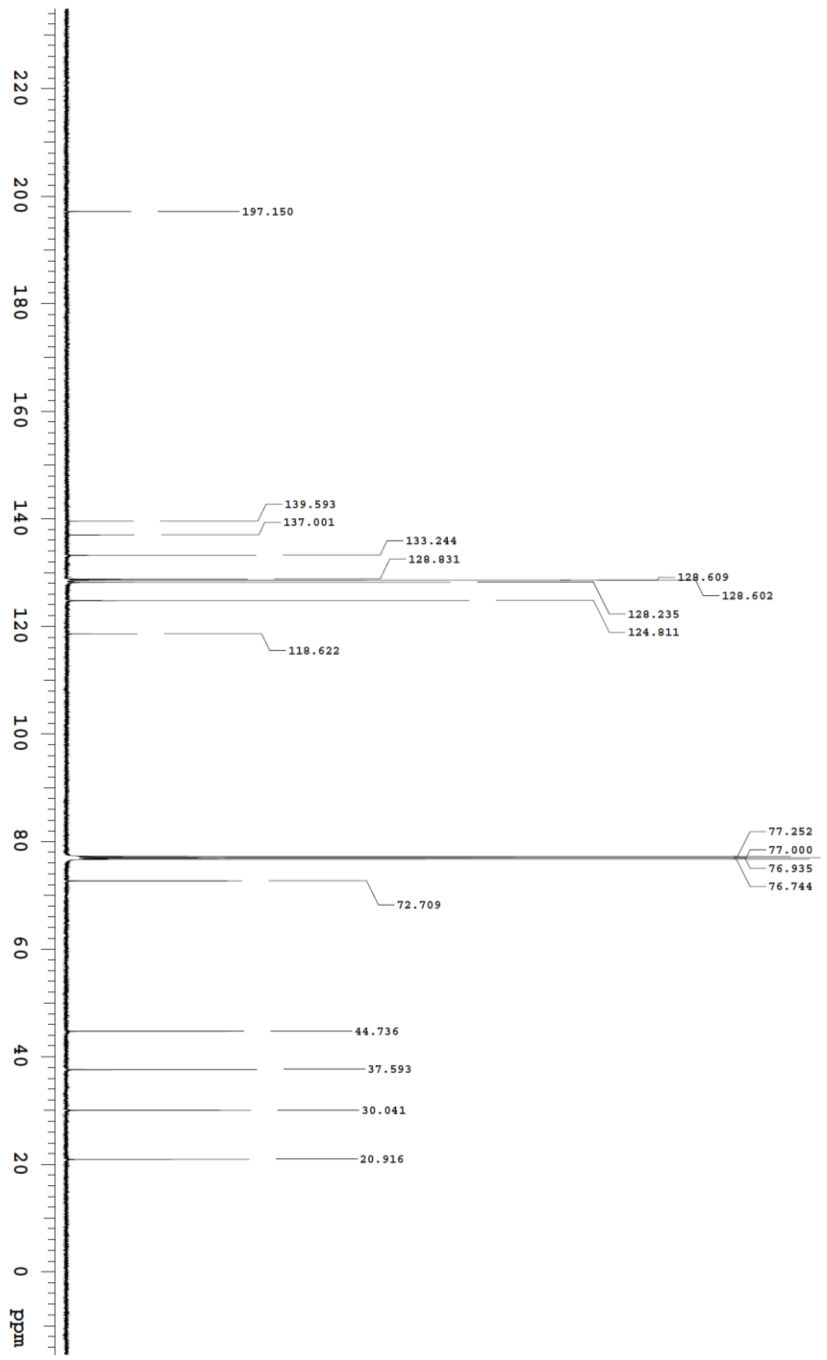

Supplementary Figure 39. ^{13}C NMR spectrum of (2*R*,6*S*)-6-(2-oxo-2-phenylethyl)-2-phenyltetrahydro-2*H*-pyran-2-carbonitrile (**3a**).

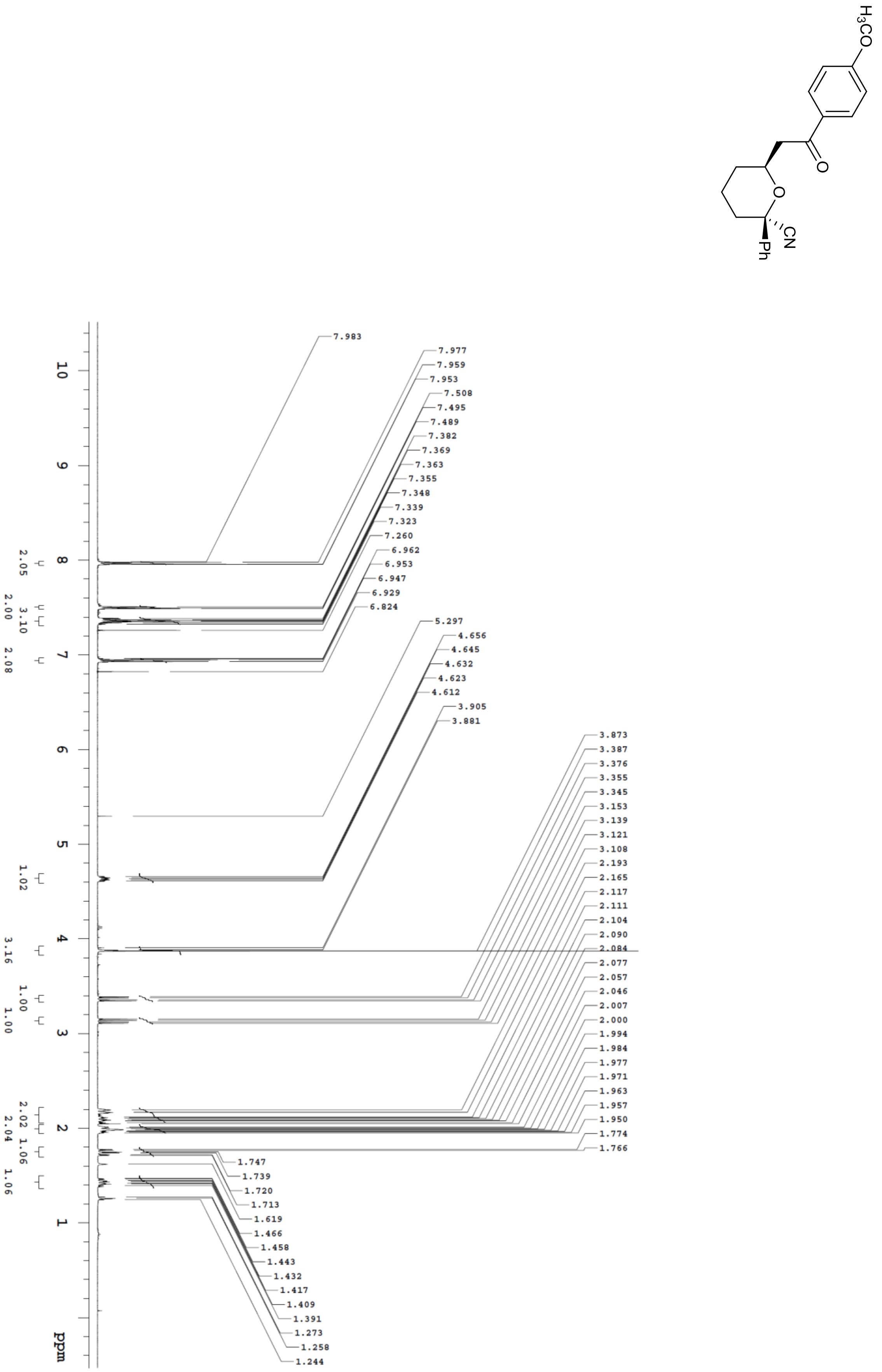

Supplementary Figure 40. ^{1}H NMR spectrum of 6-(2-(4-methoxyphenyl)-2-oxoethyl)-2-phenyltetrahydro-2*H*-pyran-2-carbonitrile (**3b**).

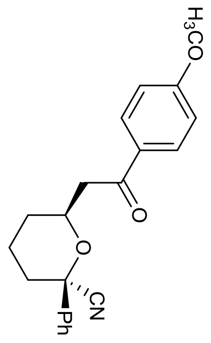

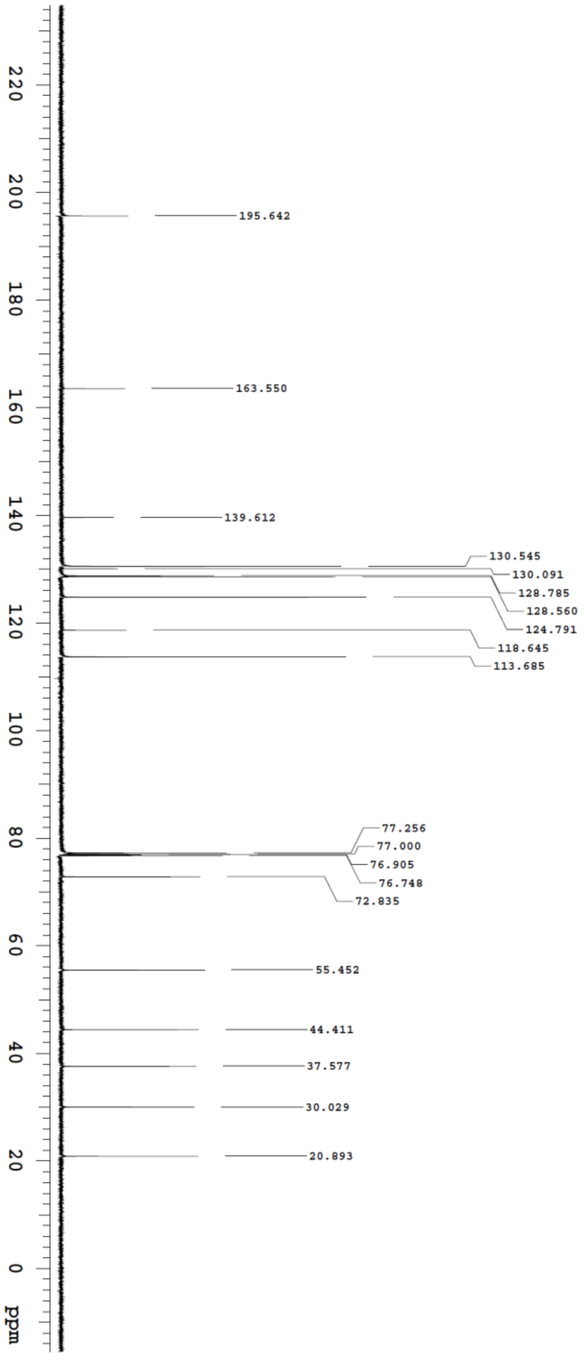

Supplementary Figure 41. ^{13}C NMR spectrum of 6-(2-(4-methoxyphenyl)-2-oxoethyl)-2-phenyltetrahydro-2*H*-pyran-2-carbonitrile (**3b**).

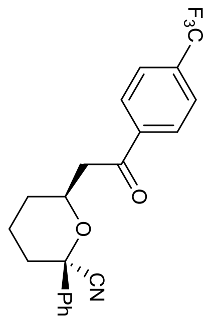

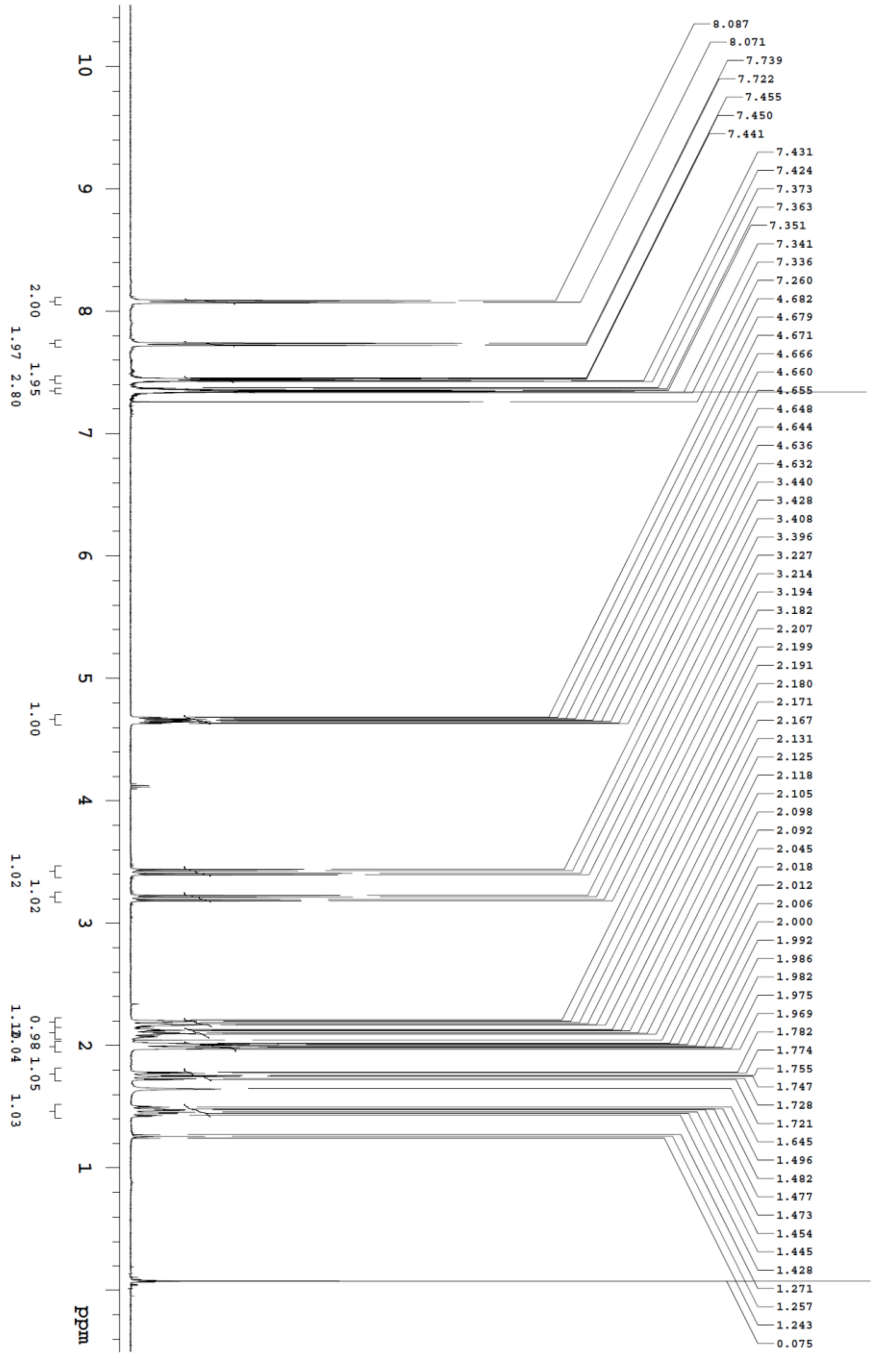

Supplementary Figure 42. ^{1}H NMR spectrum of 6-(2-oxo-2-(4-(trifluoromethyl)phenyl)ethyl)-2-phenyltetrahydro-2*H*-pyran-2-carbonitrile (**3c**).

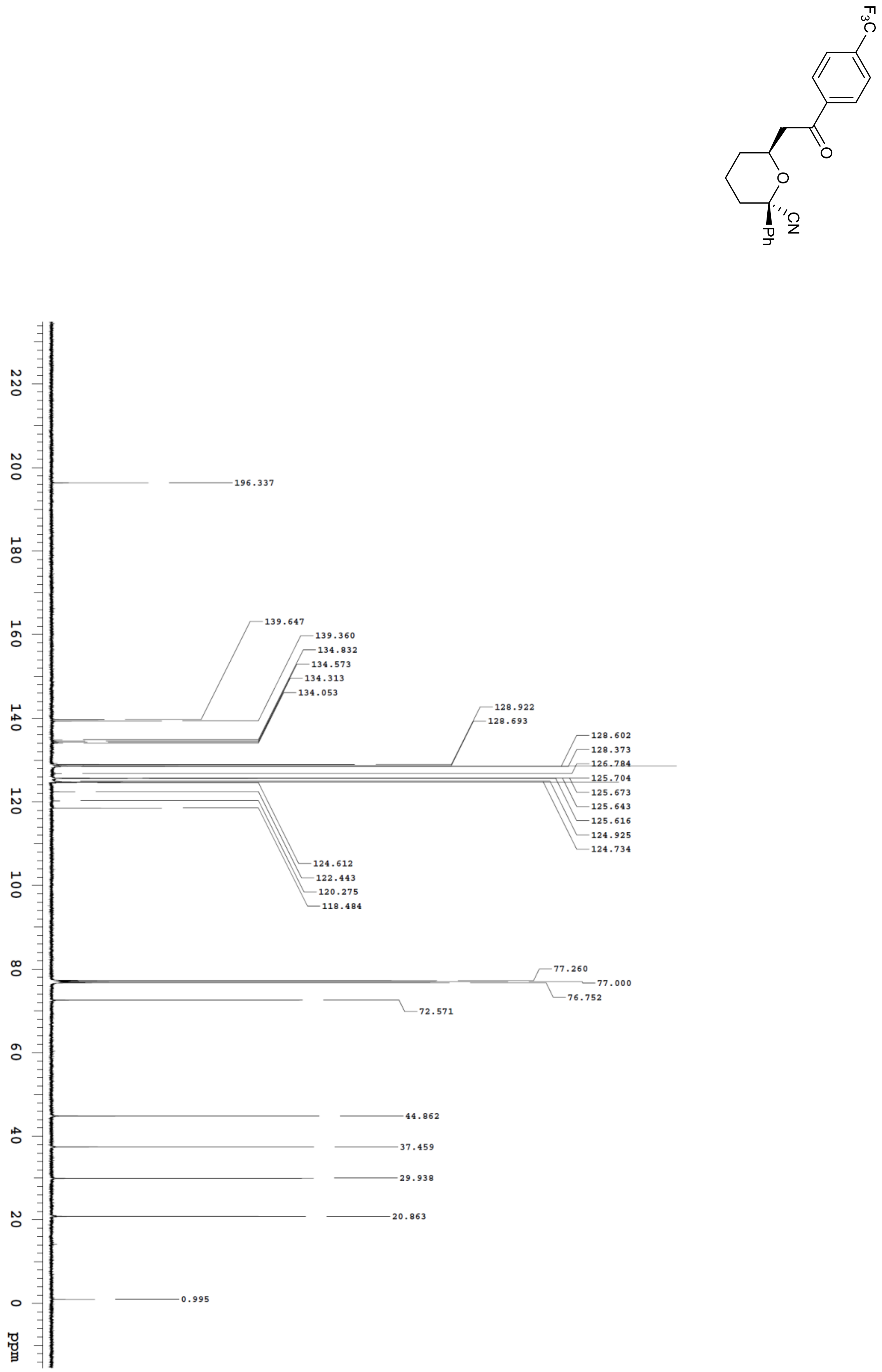

Supplementary Figure 43. ^{13}C NMR spectrum of 6-(2-oxo-2-(4-(trifluoromethyl)phenyl)ethyl)-2-phenyltetrahydro-2*H*-pyran-2-carbonitrile (**3c**).

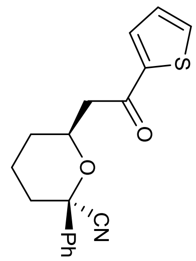

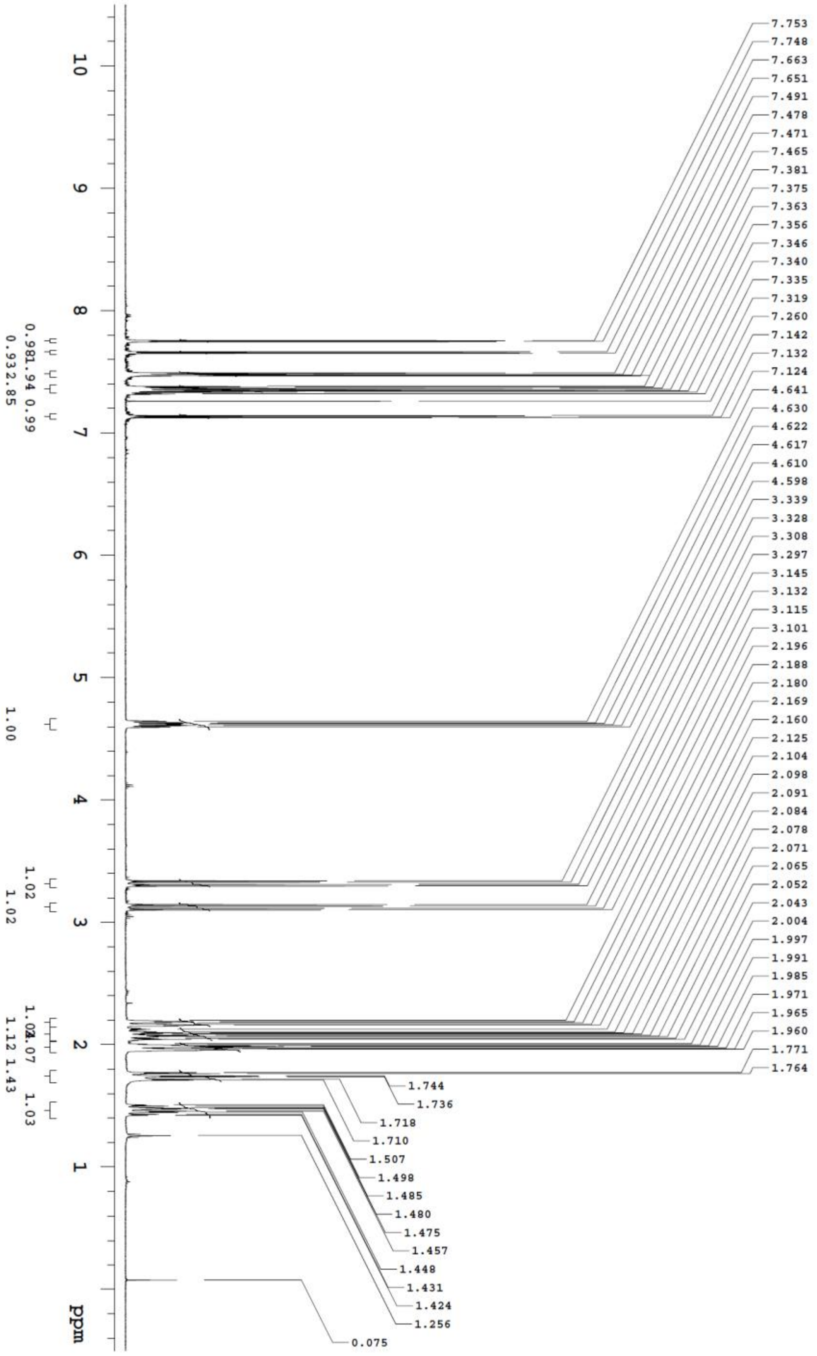

Supplementary Figure 44. ^{1}H NMR spectrum of 6-(2-oxo-2-(thiophen-2-yl)ethyl)-2-phenyltetrahydro-2*H*-pyran-2-carbonitrile (**3d**).

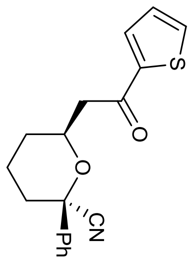

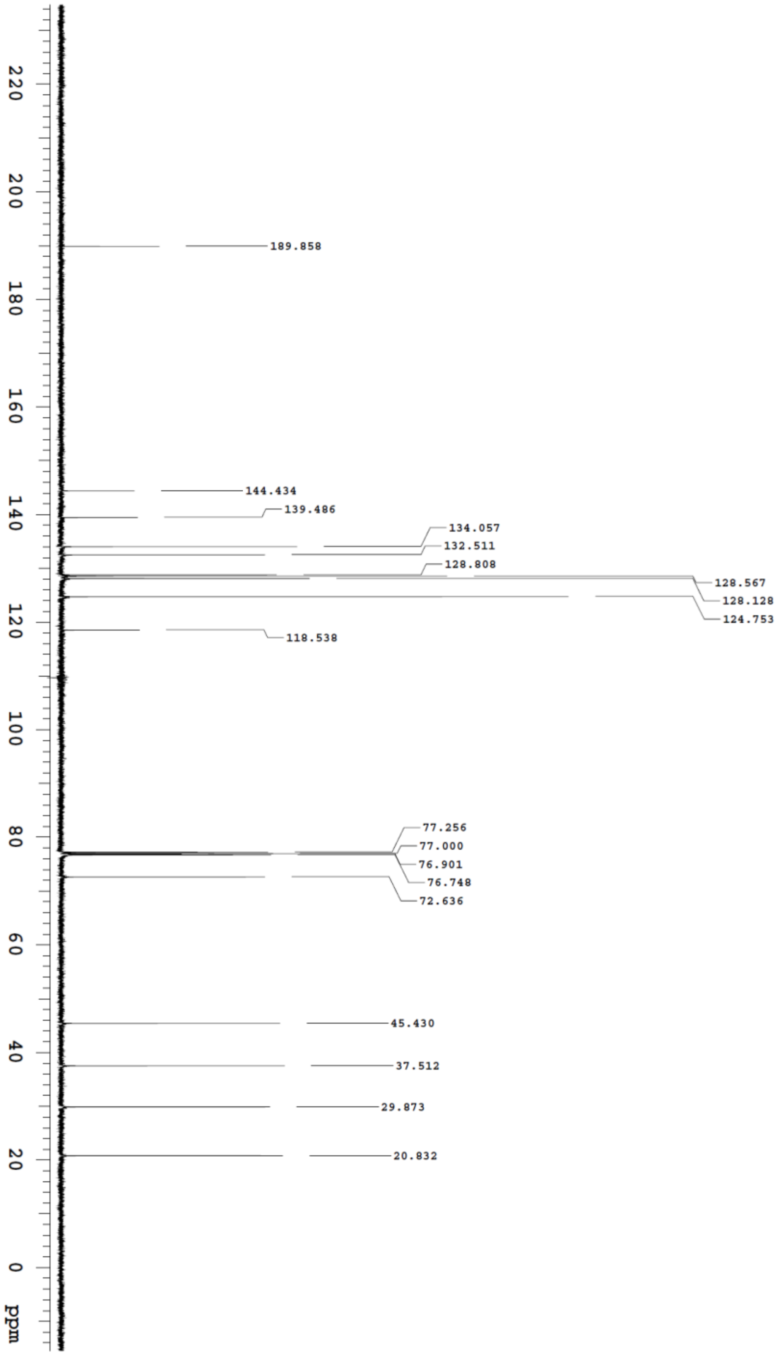

Supplementary Figure 45. ^{13}C NMR spectrum of 6-(2-oxo-2-(thiophen-2-yl)ethyl)-2-phenyltetrahydro-2*H*-pyran-2-carbonitrile (**3d**).

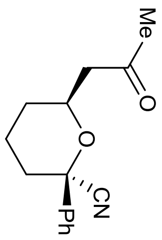

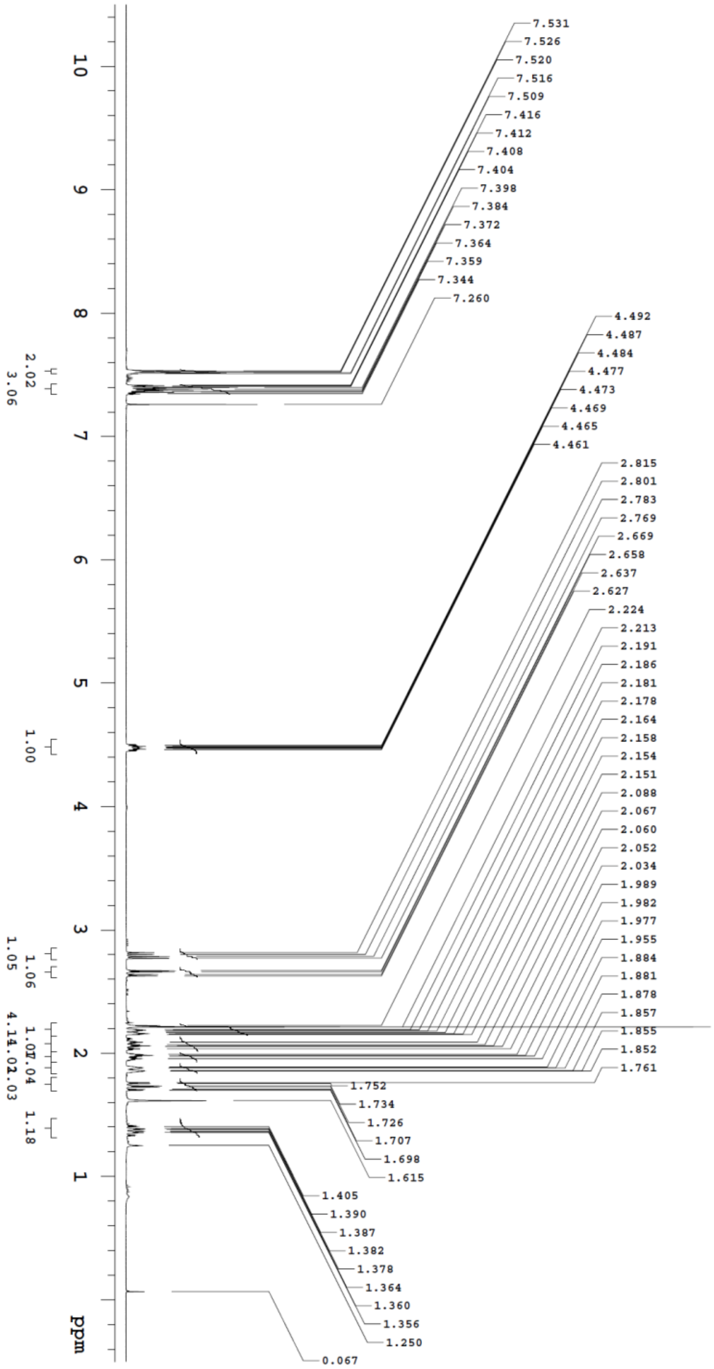

Supplementary Figure 46. ^{1}H NMR spectrum of 6-(2-oxopropyl)-2-phenyltetrahydro-2*H*-pyran-2-carbonitrile (**3e**).

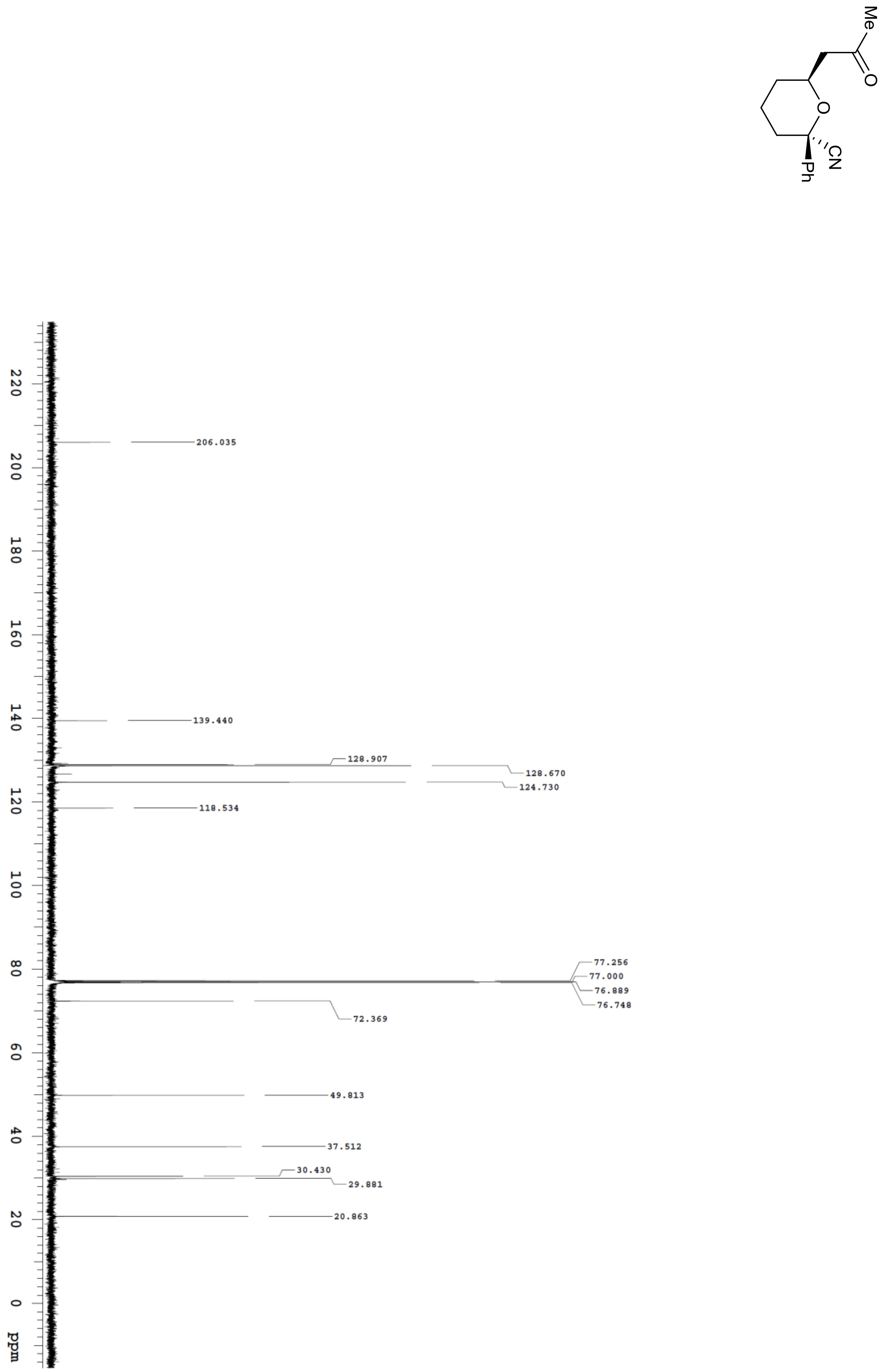

Supplementary Figure 47. ^{13}C NMR spectrum of 6-(2-oxopropyl)-2-phenyltetrahydro-2*H*-pyran-2-carbonitrile (**3e**).

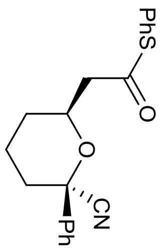

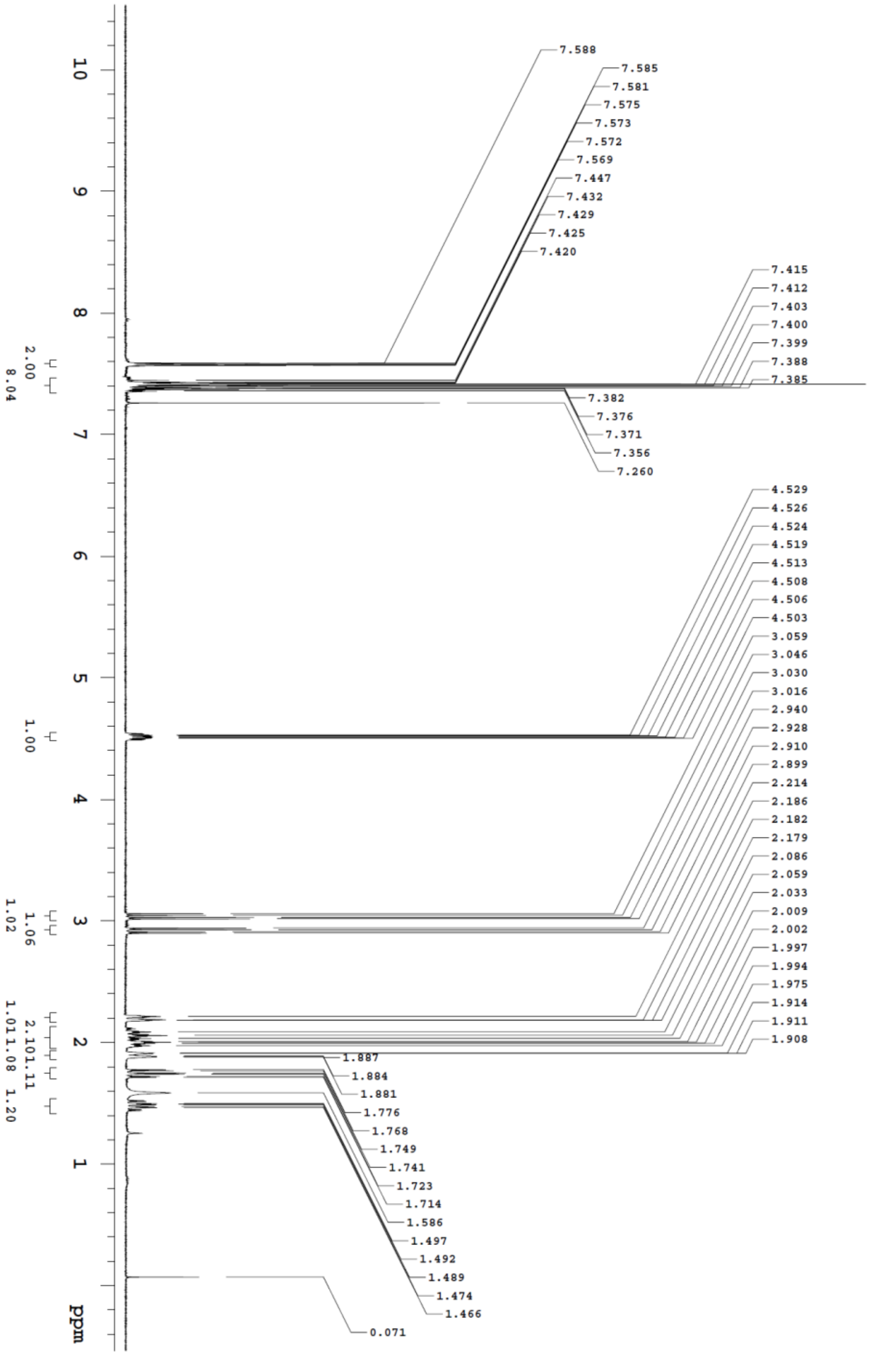

Supplementary Figure 48. ^{1}H NMR spectrum of *S*-phenyl 2-(6-cyano-6-phenyltetrahydro-2*H*-pyran-2-yl)ethanethioate (**3f**).

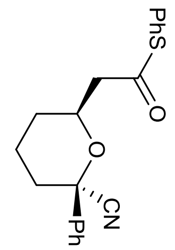

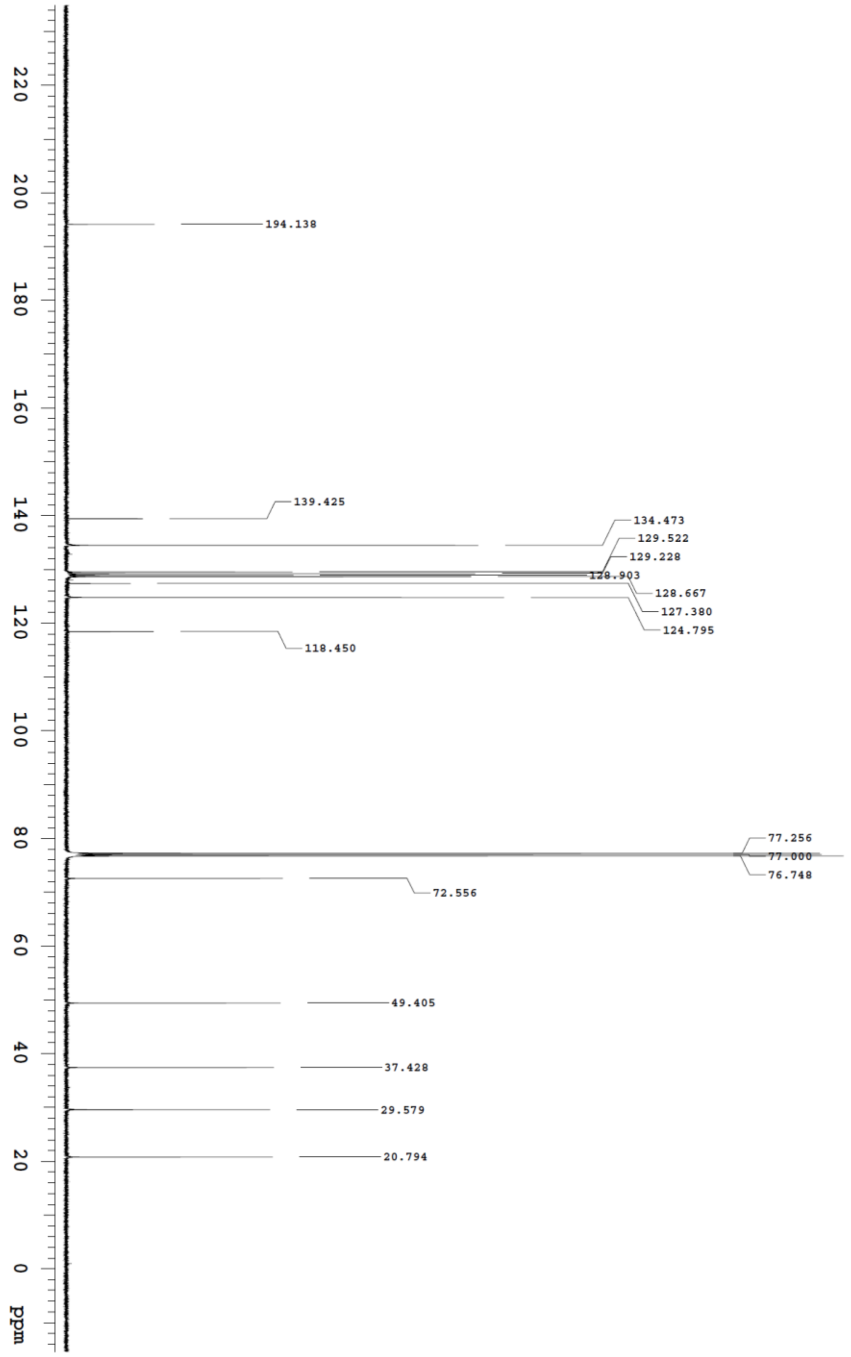

Supplementary Figure 49. ^{13}C NMR spectrum of *S*-phenyl 2-(6-cyano-6-phenyltetrahydro-2*H*-pyran-2-yl)ethanethioate (**3f**).

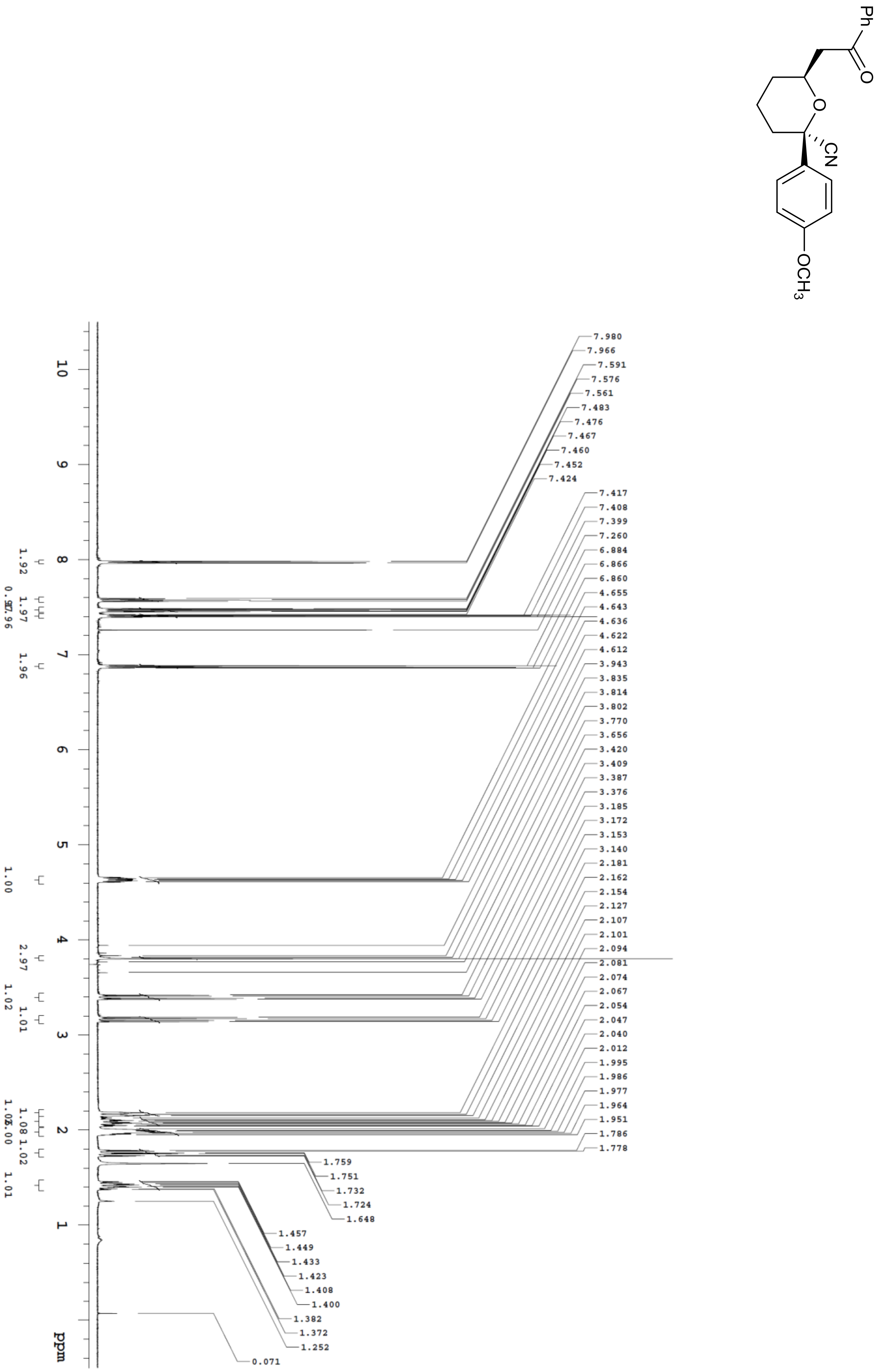

Supplementary Figure 50. ^{1}H NMR spectrum of 2-(4-methoxyphenyl)-6-(2-oxo-2-phenylethyl)tetrahydro-2*H*-pyran-2-carbonitrile (**3g**).

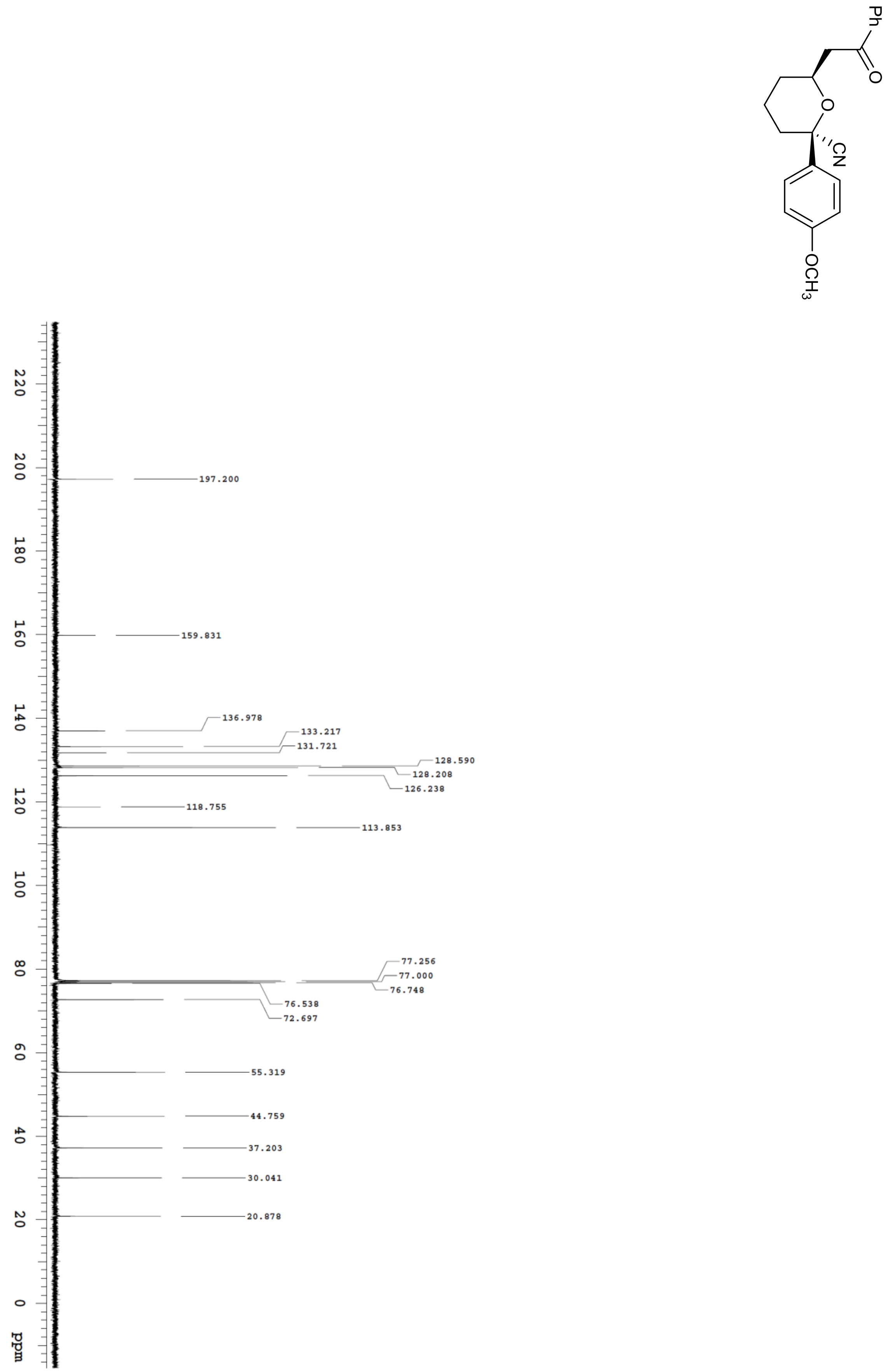

Supplementary Figure 51. ^{13}C NMR spectrum of 2-(4-methoxyphenyl)-6-(2-oxo-2-phenylethyl)tetrahydro-2*H*-pyran-2-carbonitrile (**3g**).

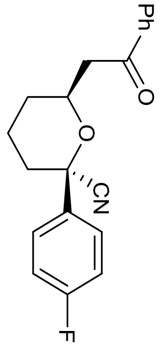

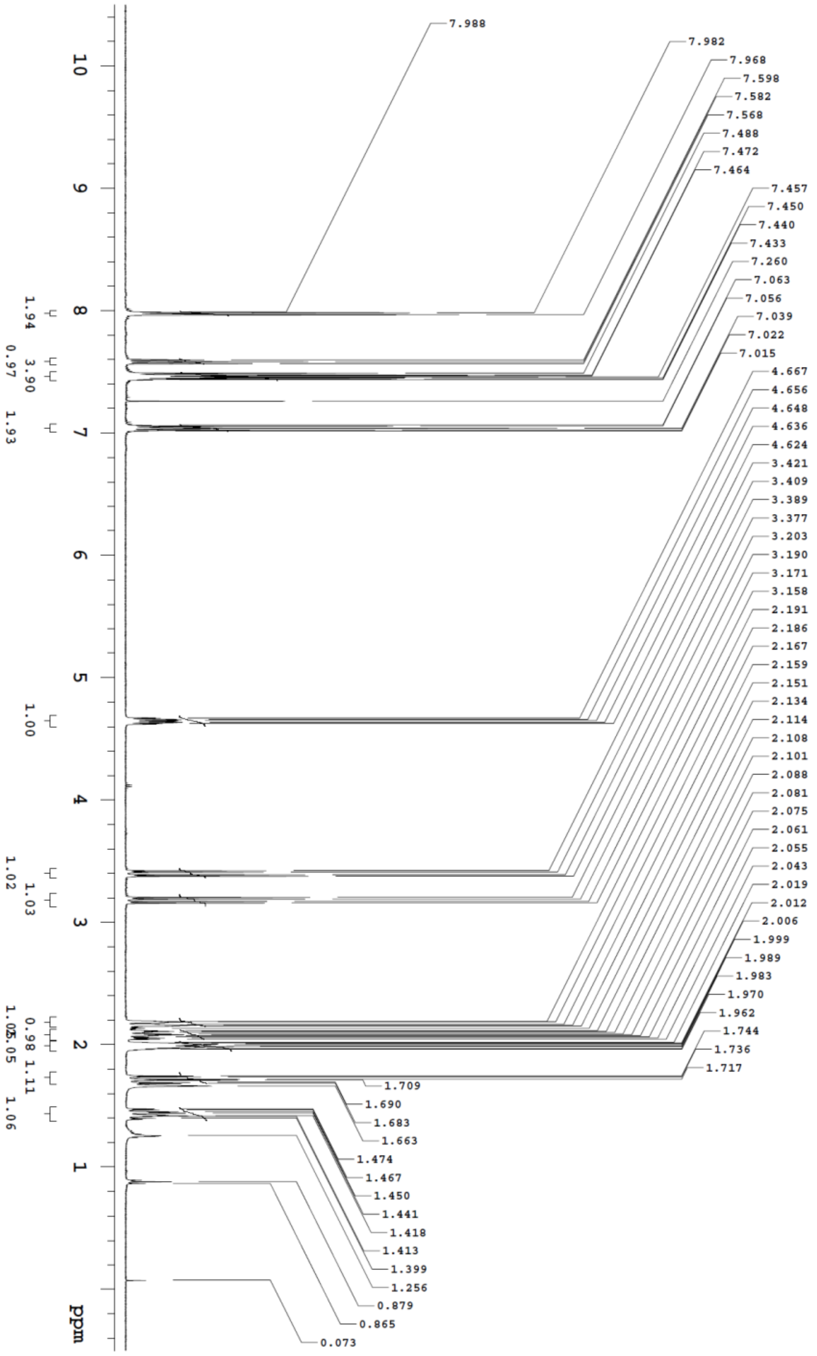

Supplementary Figure 52. ^{1}H NMR spectrum of 2-(4-fluorophenyl)-6-(2-oxo-2-phenylethyl)tetrahydro-2*H*-pyran-2-carbonitrile (**3h**).

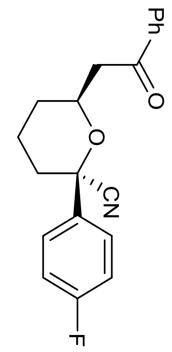

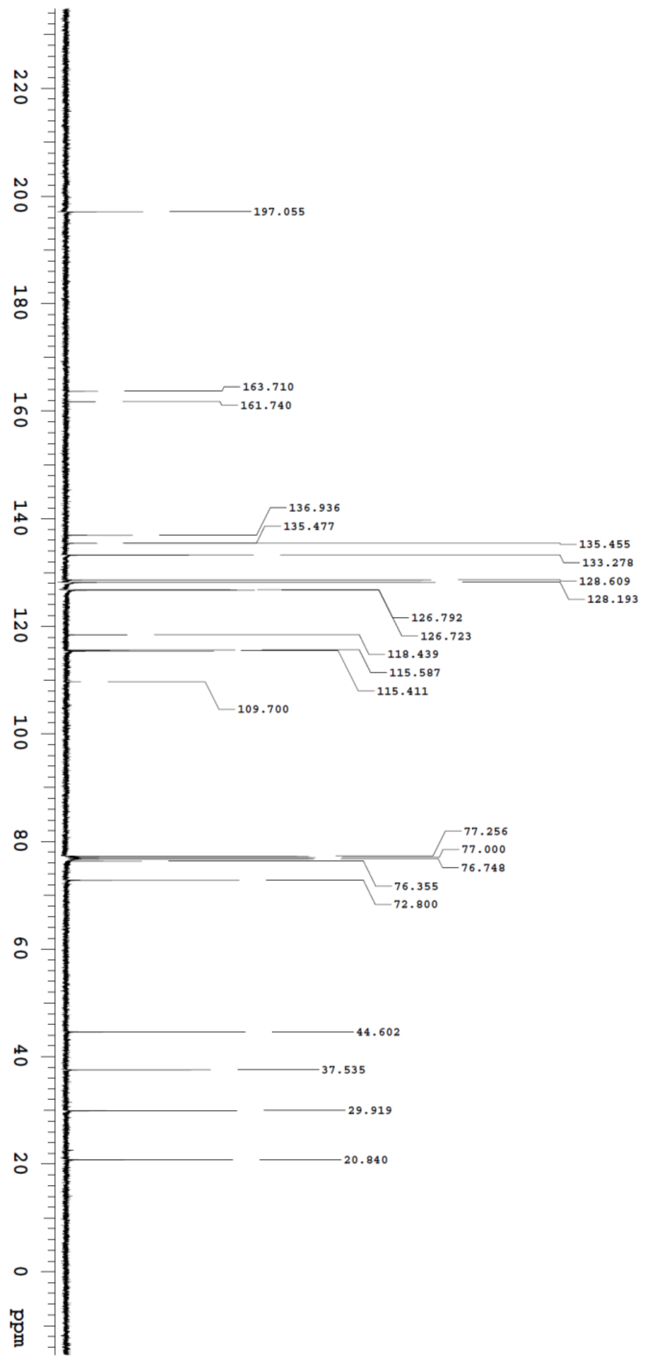

Supplementary Figure 53. ^{13}C NMR spectrum of 2-(4-fluorophenyl)-6-(2-oxo-2-phenylethyl)tetrahydro-2*H*-pyran-2-carbonitrile (**3h**).

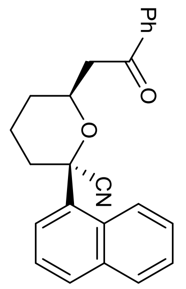

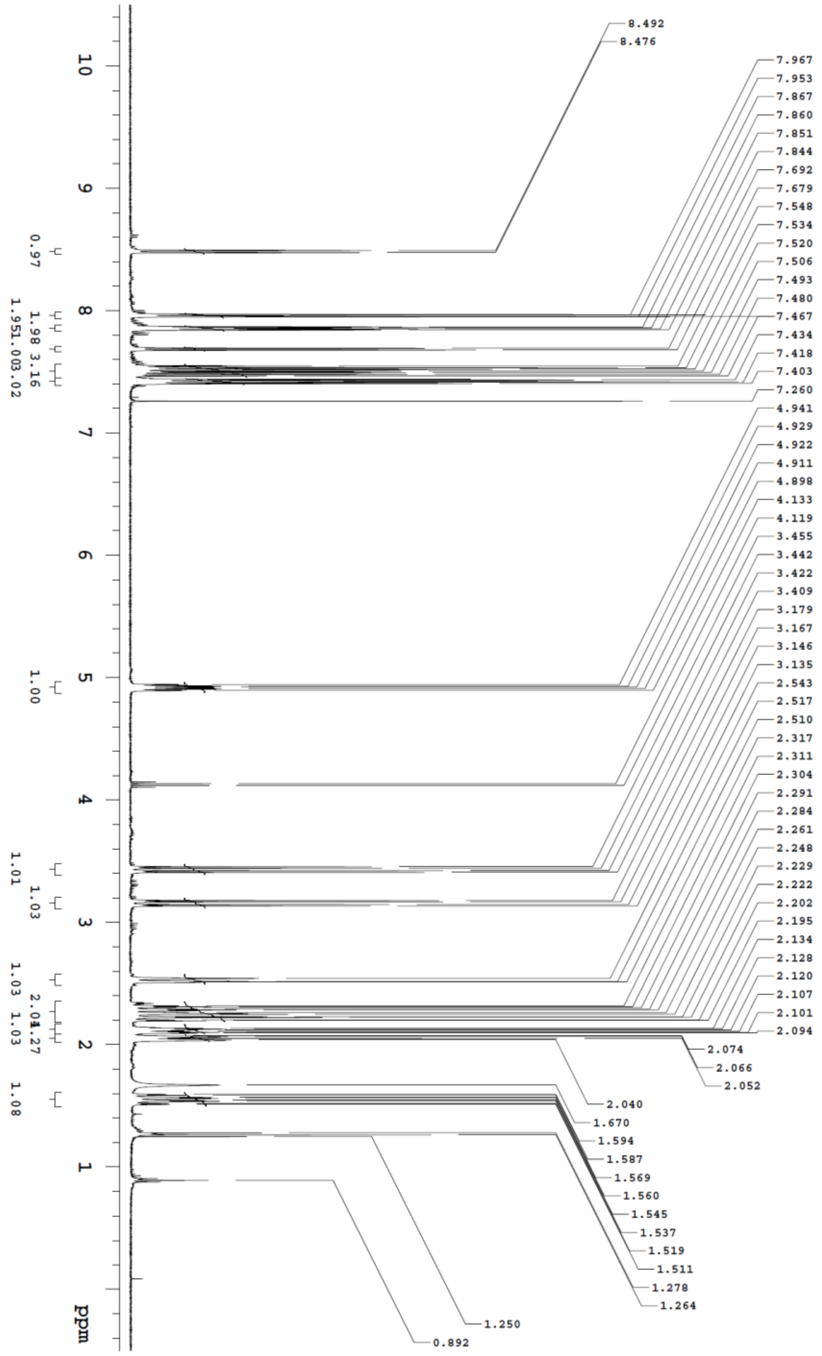

Supplementary Figure 54. ^{1}H NMR spectrum of 2-(naphthalen-1-yl)-6-(2-oxo-2-phenylethyl)tetrahydro-2*H*-pyran-2-carbonitrile (**3i**).

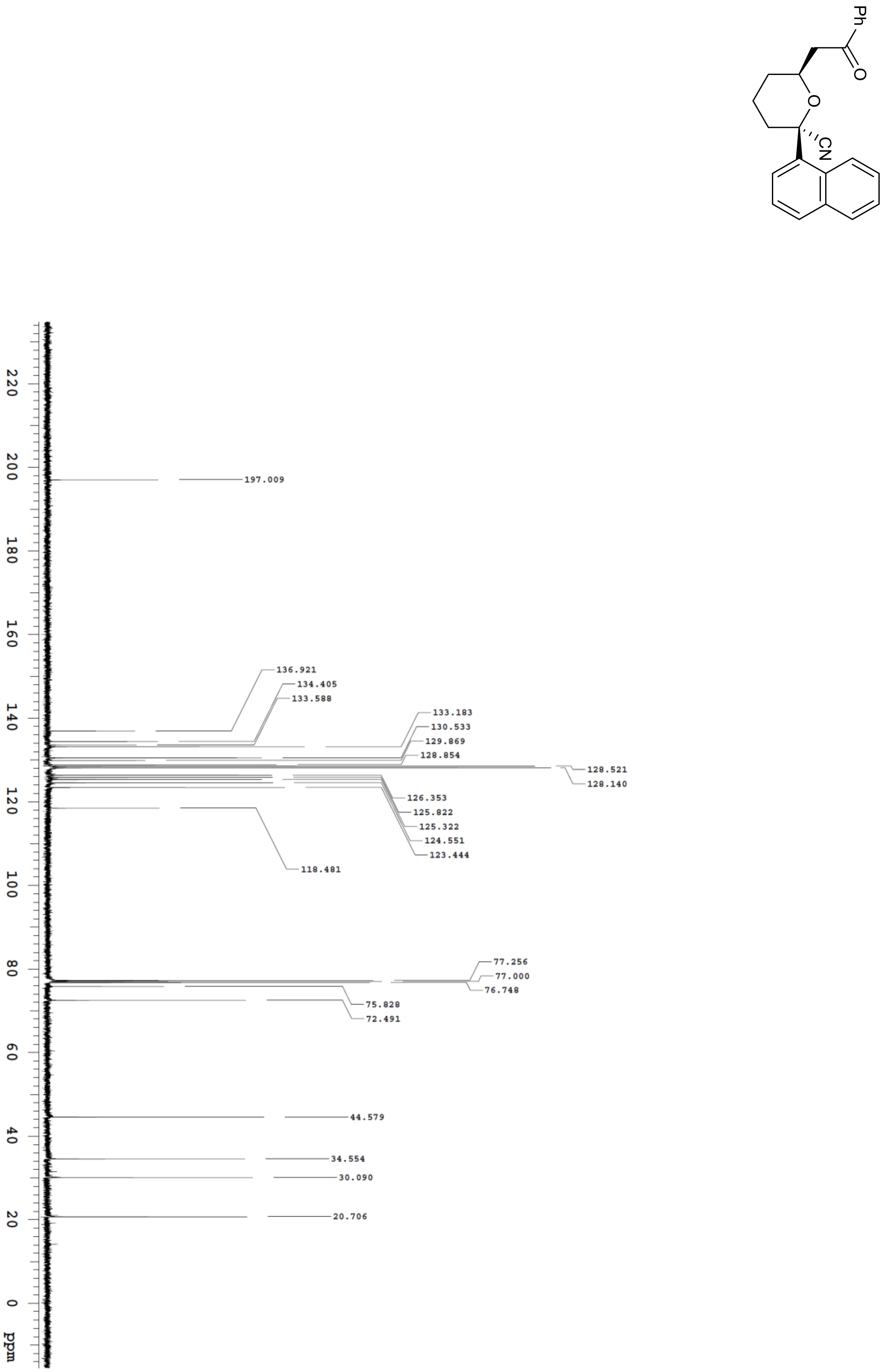

Supplementary Figure 55. ^{13}C NMR spectrum of 2-(naphthalen-1-yl)-6-(2-oxo-2-phenylethyl)tetrahydro-2*H*-pyran-2-carbonitrile (**3i**).

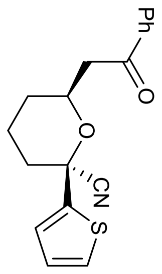

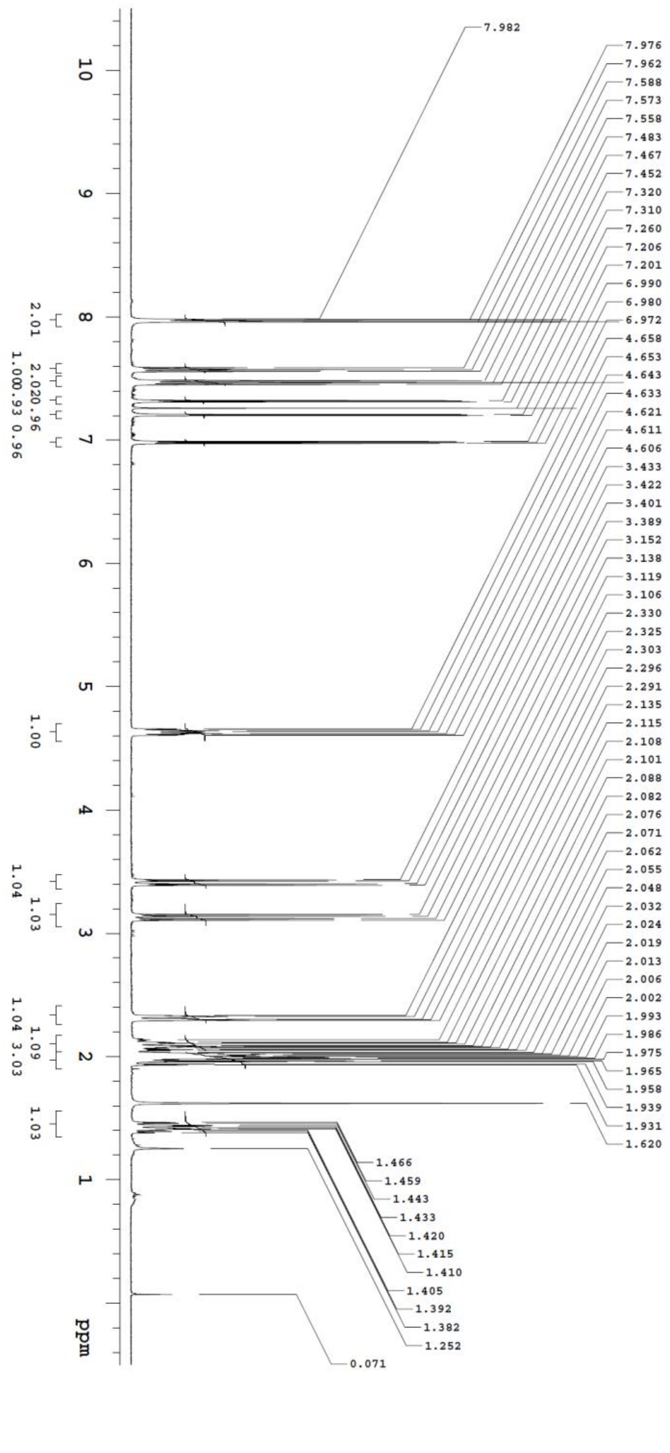

Supplementary Figure 56. ^{1}H NMR spectrum of 6-(2-oxo-2-phenylethyl)-2-(thiophen-2-yl)tetrahydro-2*H*-pyran-2-carbonitrile (**3j**).

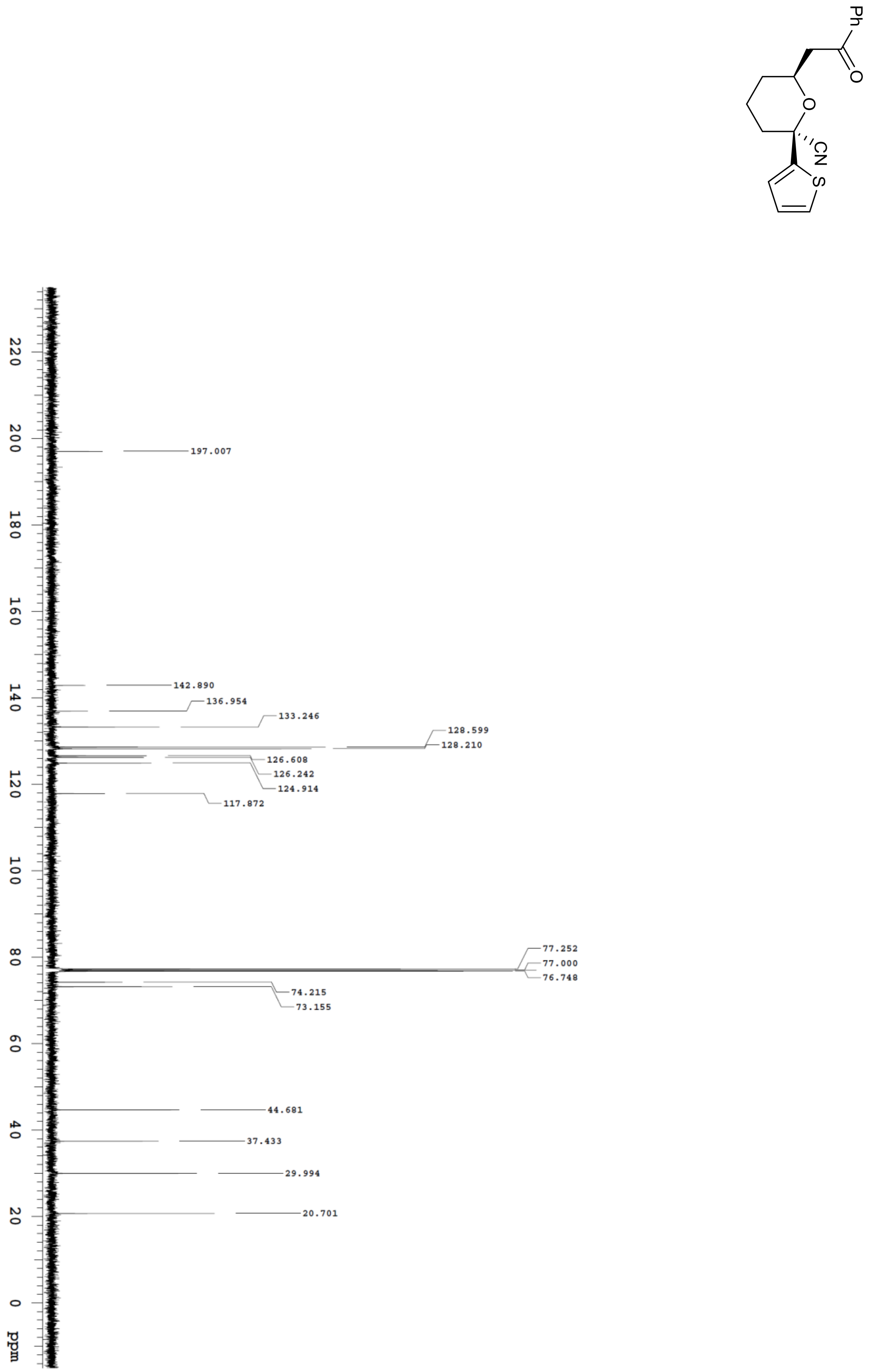

Supplementary Figure 57. ^{13}C NMR spectrum of 6-(2-oxo-2-phenylethyl)-2-(thiophen-2-yl)tetrahydro-2*H*-pyran-2-carbonitrile (**3j**).

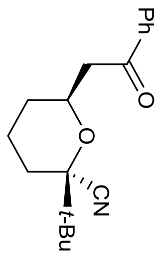

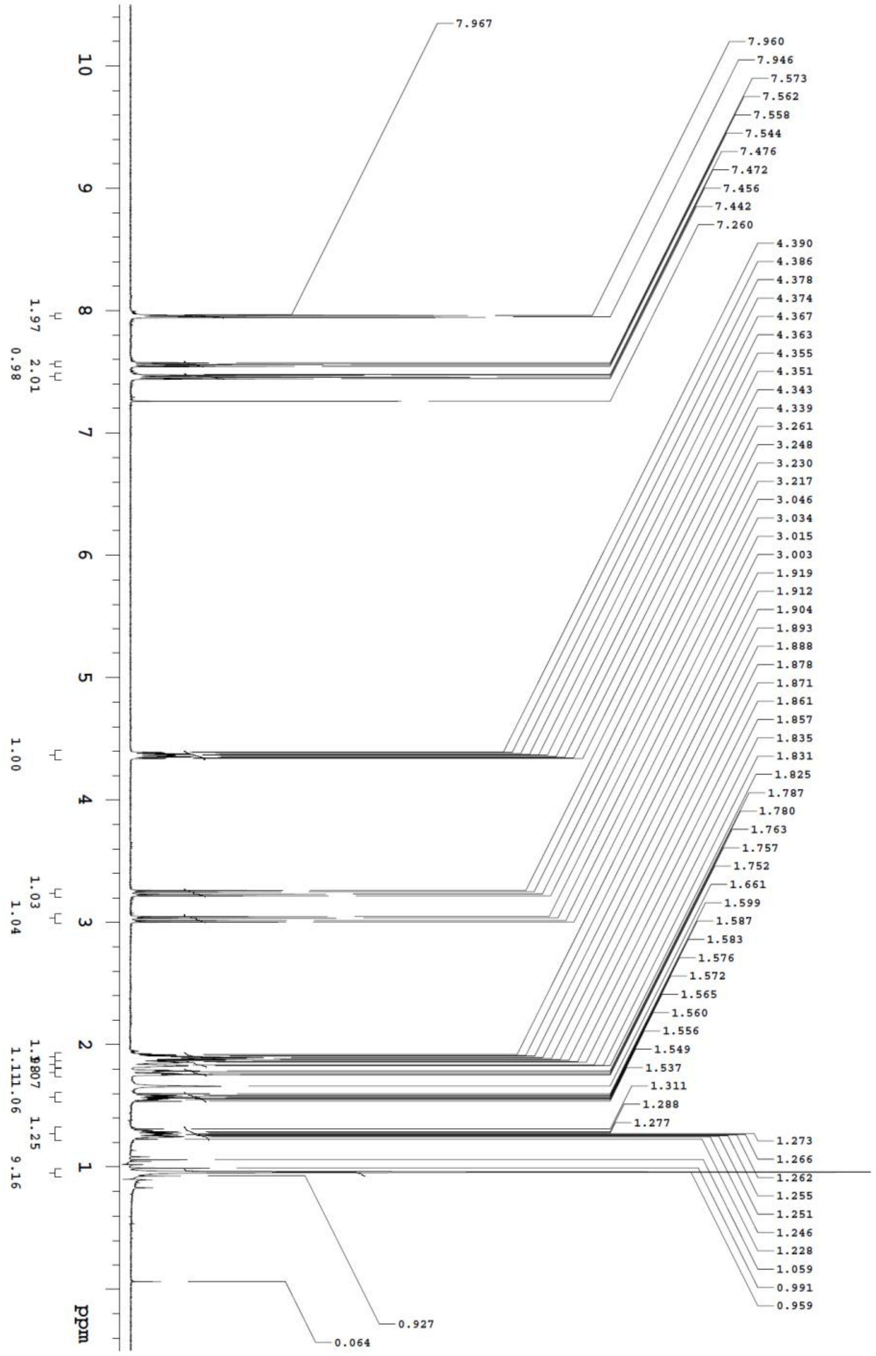

Supplementary Figure 58. ^{1}H NMR spectrum of 2-(*tert*-butyl)-6-(2-oxo-2-phenylethyl)tetrahydro-2*H*-pyran-2-carbonitrile (**3k**).

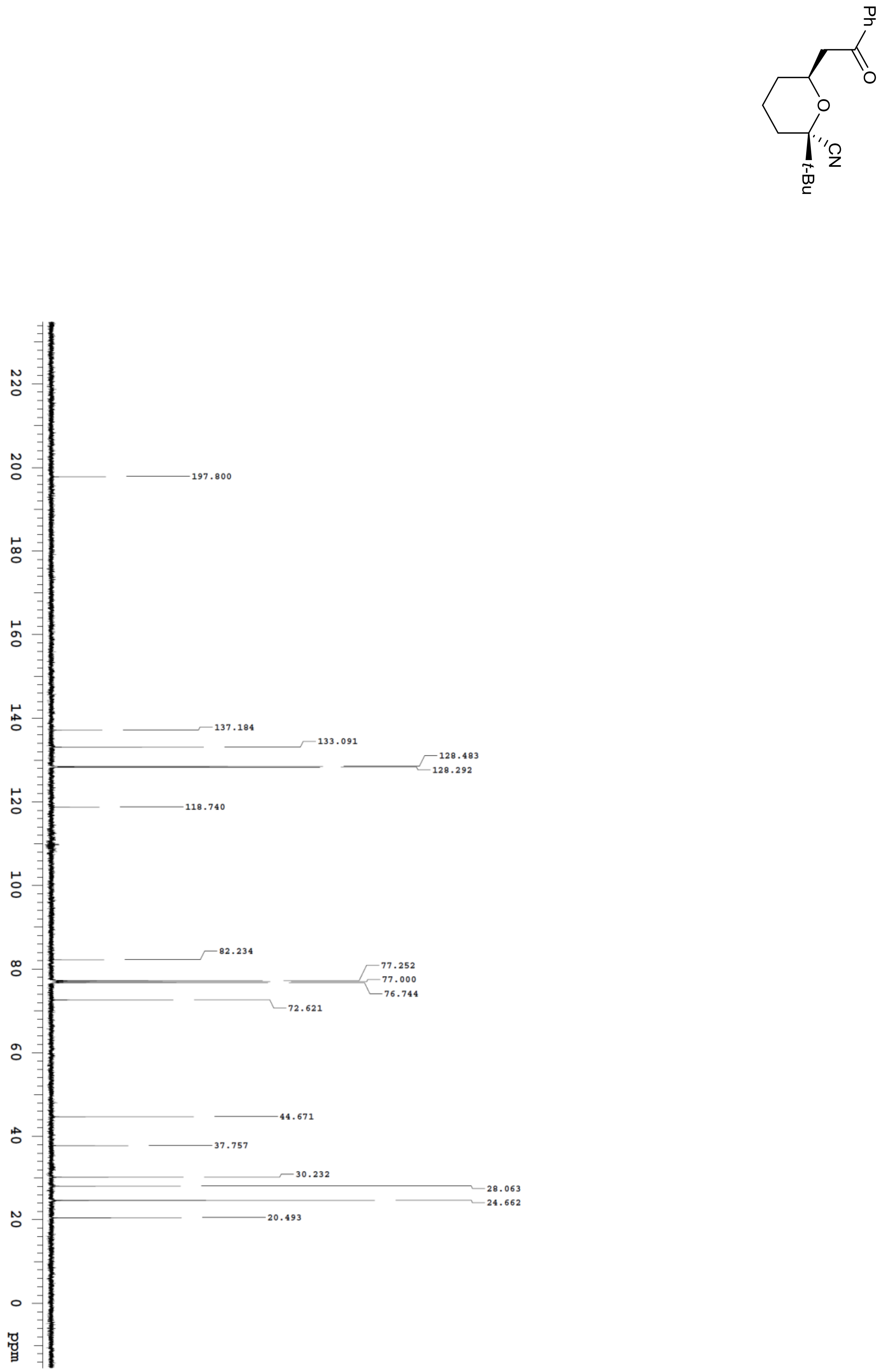

Supplementary Figure 59. ^{13}C NMR spectrum of 2-(*tert*-butyl)-6-(2-oxo-2-phenylethyl)tetrahydro-2*H*-pyran-2-carbonitrile (**3k**).

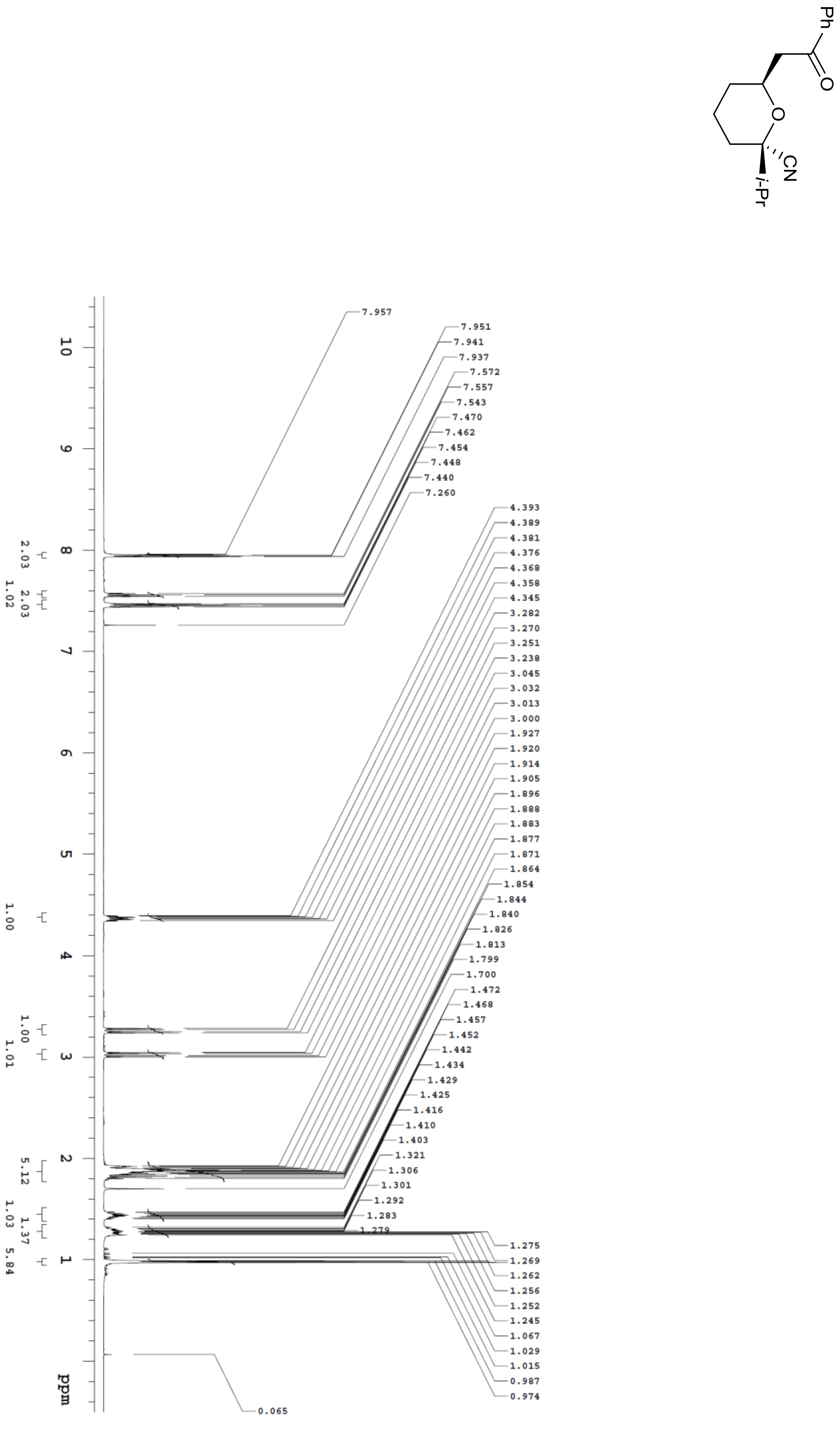

Supplementary Figure 60. ^{1}H NMR spectrum of 2-isopropyl-6-(2-oxo-2-phenylethyl)tetrahydro-2*H*-pyran-2-carbonitrile (**3l**).

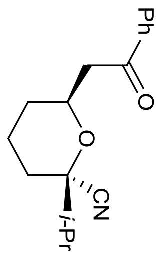

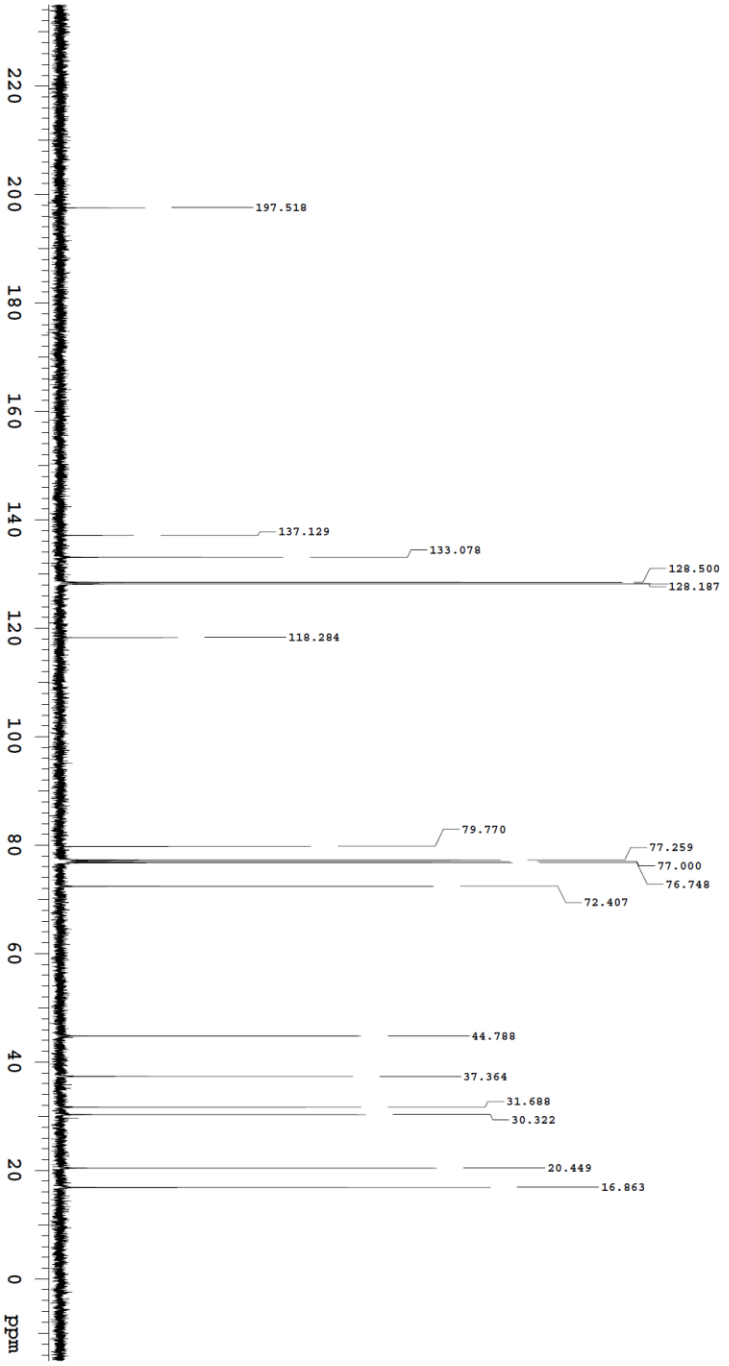

Supplementary Figure 61. ^{13}C NMR spectrum of 2-isopropyl-6-(2-oxo-2-phenylethyl)tetrahydro-2*H*-pyran-2-carbonitrile (**3l**).

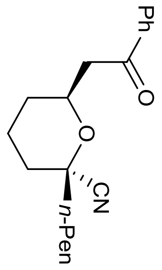

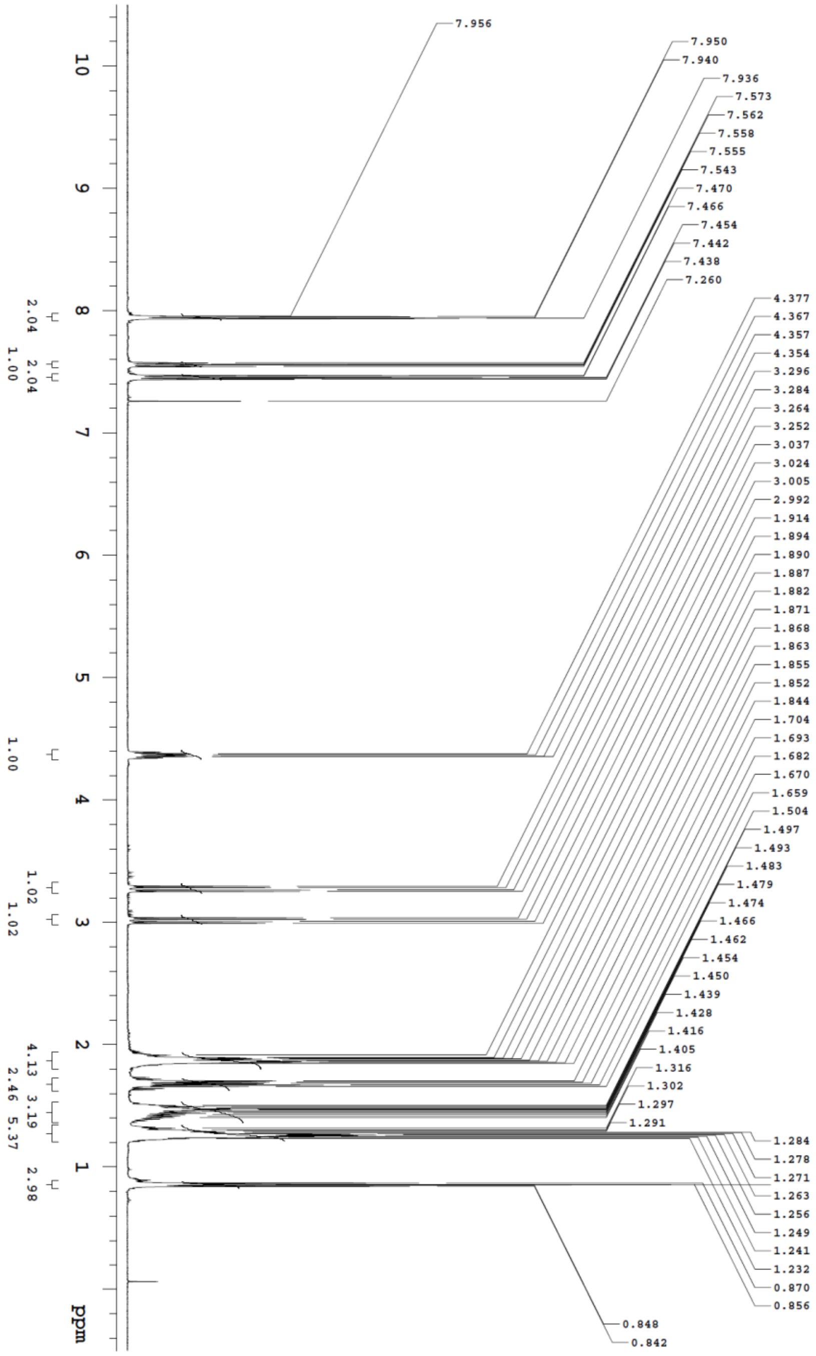

Supplementary Figure 62. ^{1}H NMR spectrum of 6-(2-oxo-2-phenylethyl)-2-pentyltetrahydro-2*H*-pyran-2-carbonitrile (**3m**).

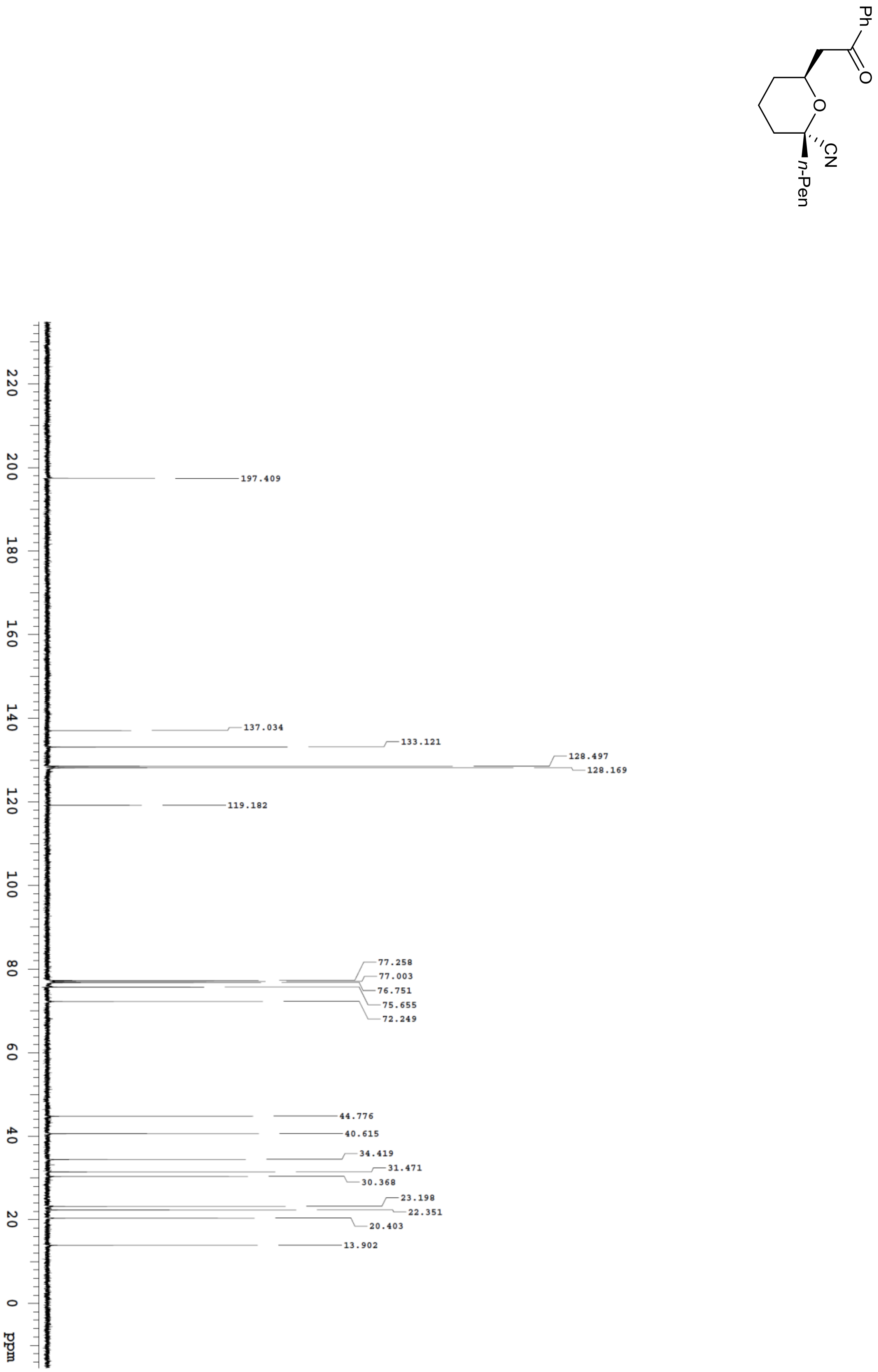

Supplementary Figure 63. ^{13}C NMR spectrum of 6-(2-oxo-2-phenylethyl)-2-pentyltetrahydro-2*H*-pyran-2-carbonitrile (**3m**).

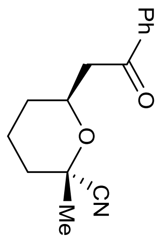

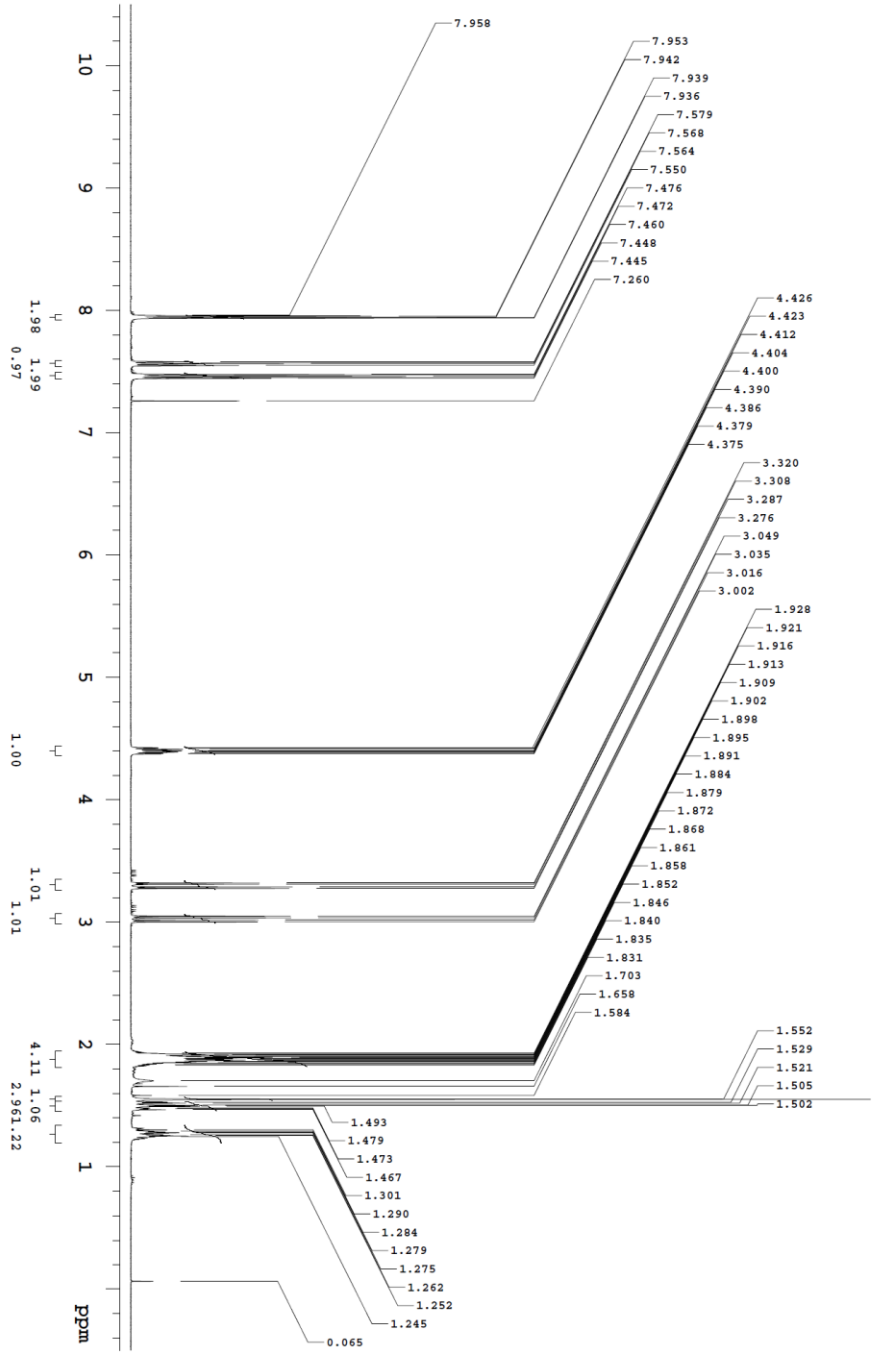

Supplementary Figure 64. ^{1}H NMR spectrum of 2-methyl-6-(2-oxo-2-phenylethyl)tetrahydro-2*H*-pyran-2-carbonitrile (**3n**).

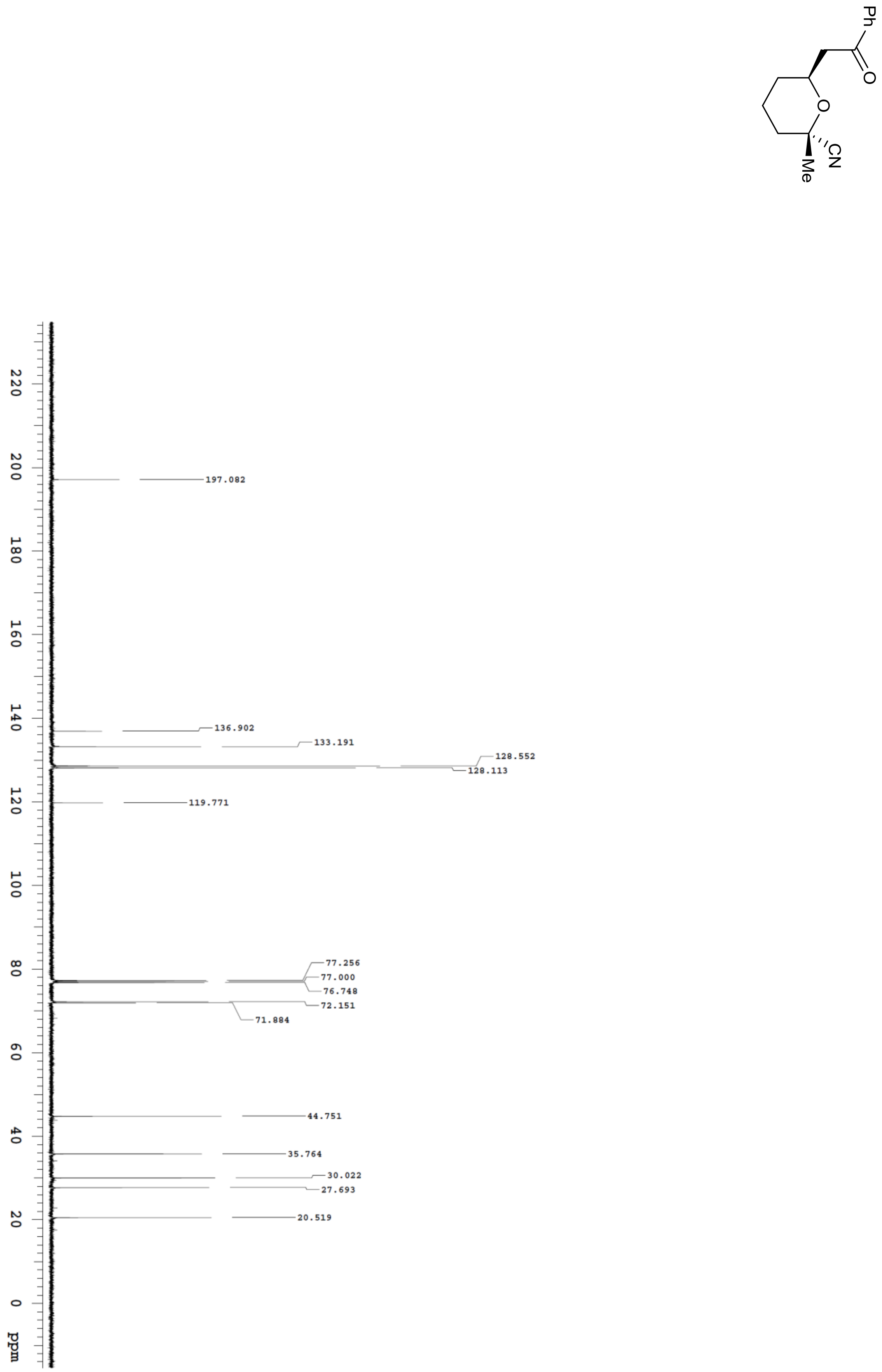

Supplementary Figure 65. ^{13}C NMR spectrum of 2-methyl-6-(2-oxo-2-phenylethyl)tetrahydro-2*H*-pyran-2-carbonitrile (**3n**).

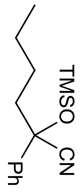

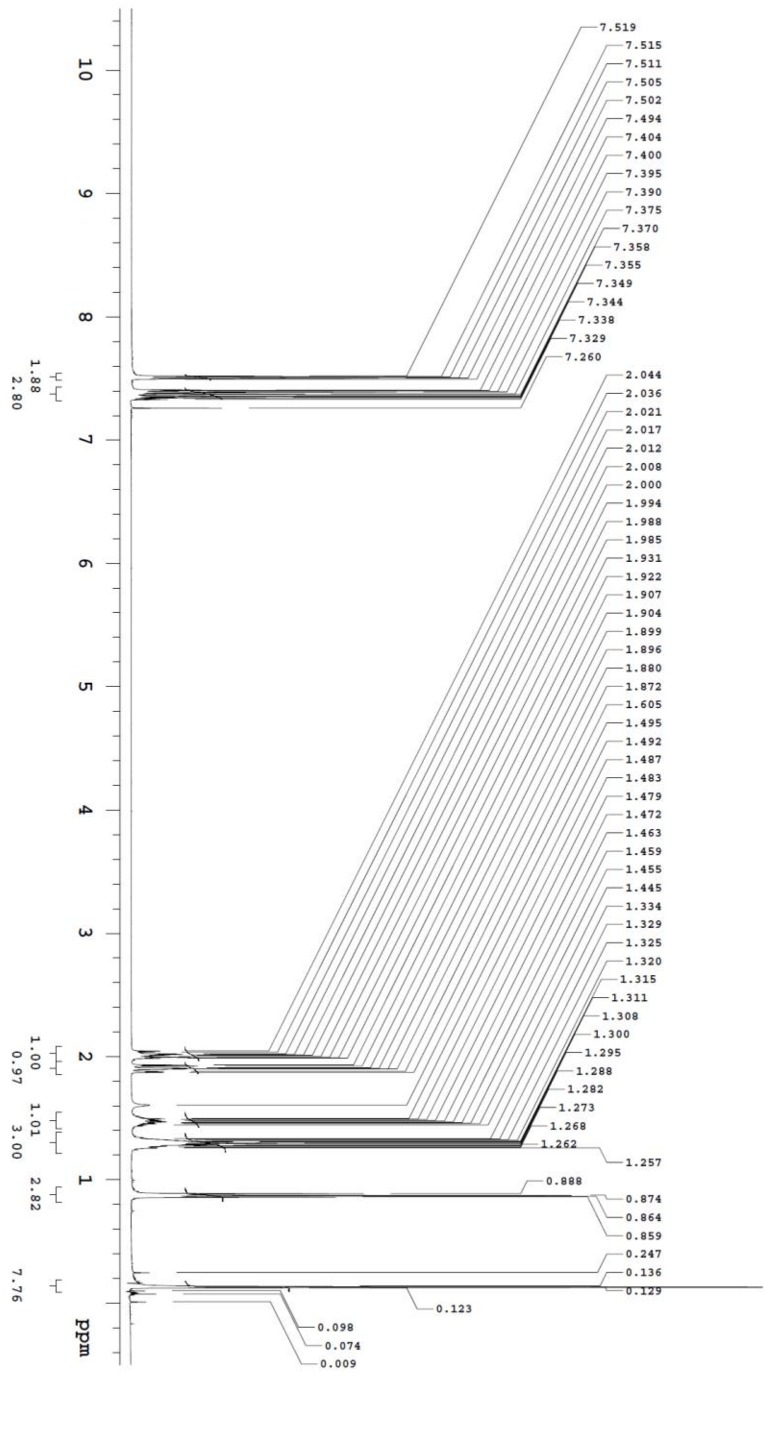

Supplementary Figure 66. ^{1}H NMR spectrum of 2-phenyl-2-((trimethylsilyl)oxy)hexanenitrile (**7**).

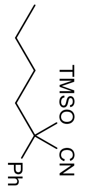

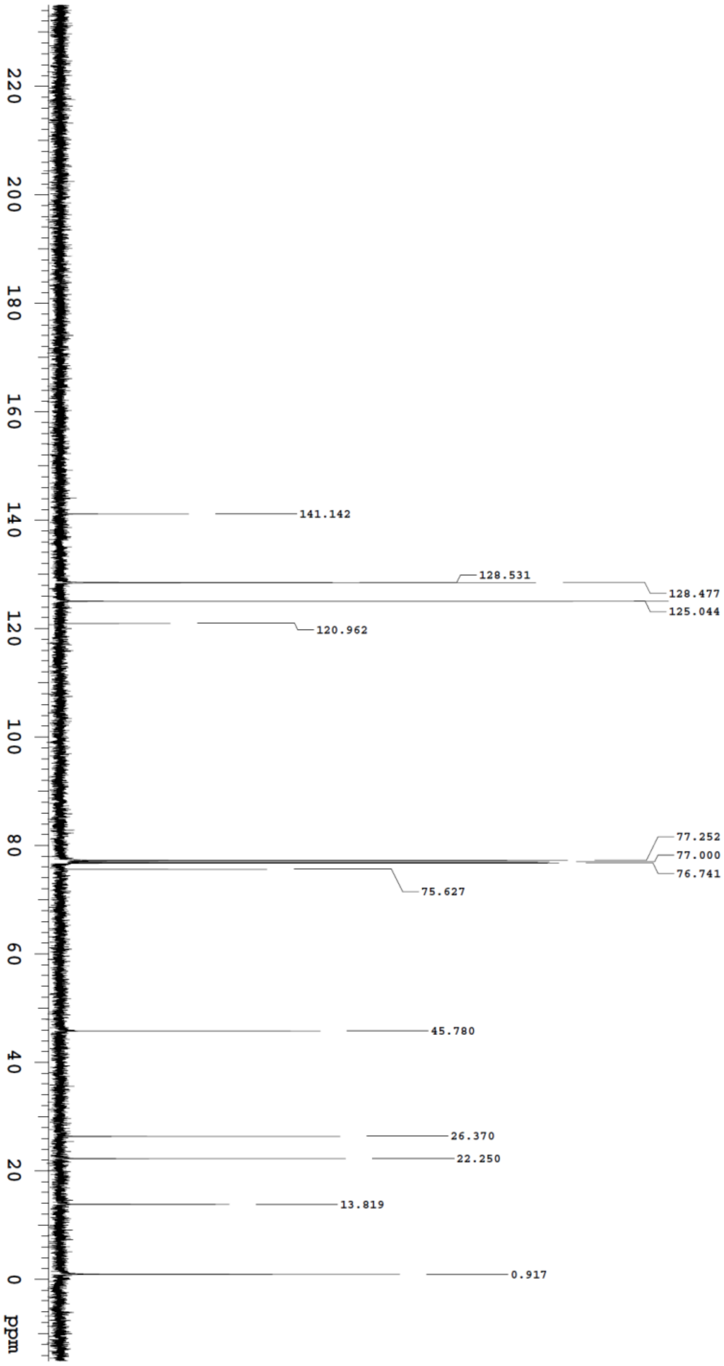

Supplementary Figure 67. ^{13}C NMR spectrum of 2-phenyl-2-((trimethylsilyl)oxy)hexanenitrile (**7**).

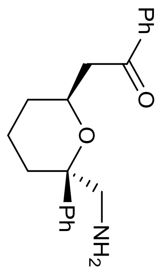

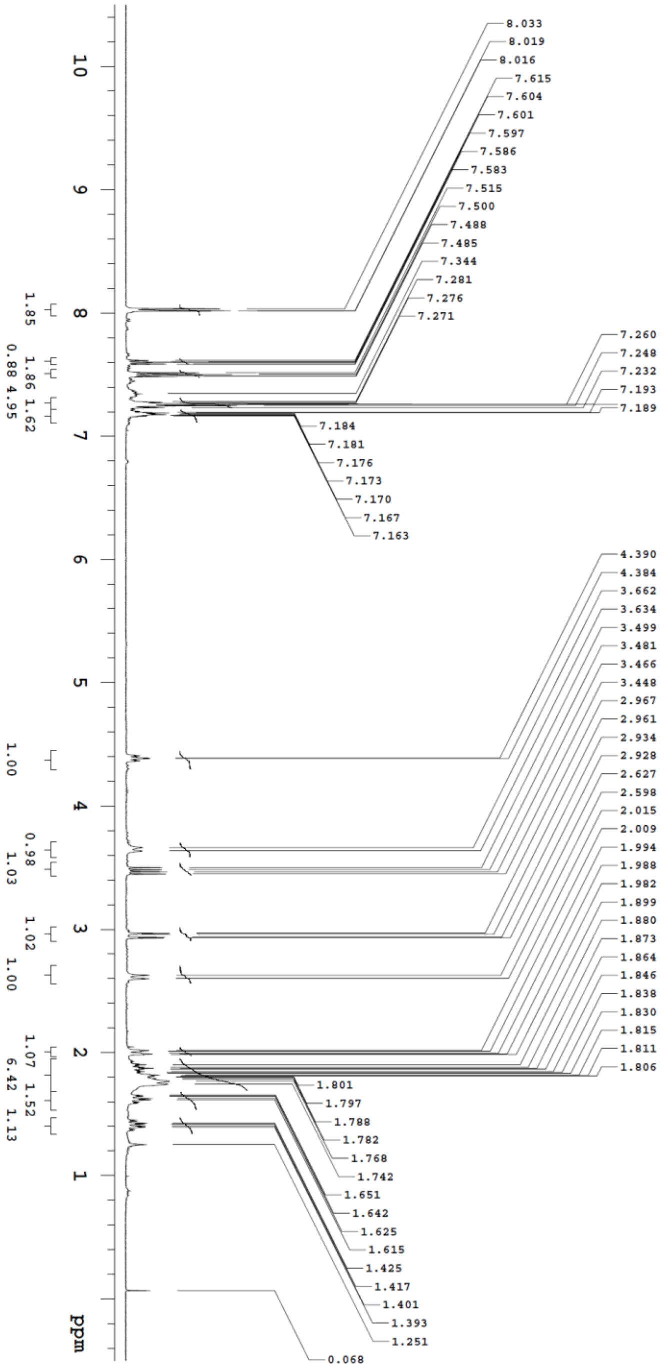

Supplementary Figure 68. ^{1}H NMR spectrum of 2-(6-(aminomethyl)-6-phenyltetrahydro-2*H*-pyran-2-yl)-1-phenylethan-1-one (**9**).

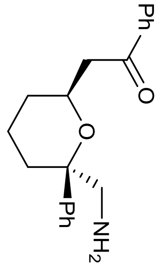

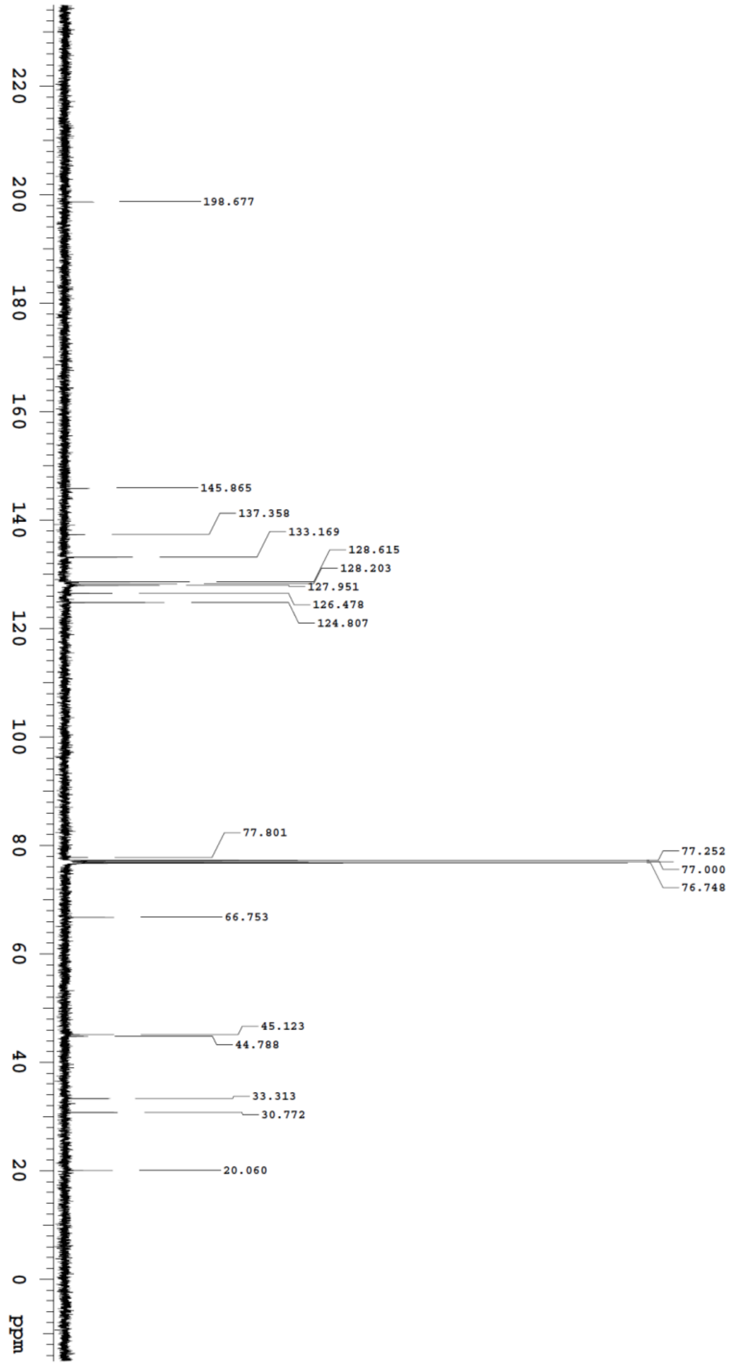

Supplementary Figure 69. ^{13}C NMR spectrum of 2-(6-(aminomethyl)-6-phenyltetrahydro-2*H*-pyran-2-yl)-1-phenylethan-1-one (**9**).

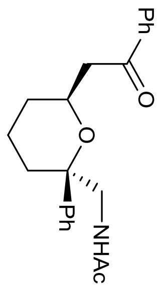

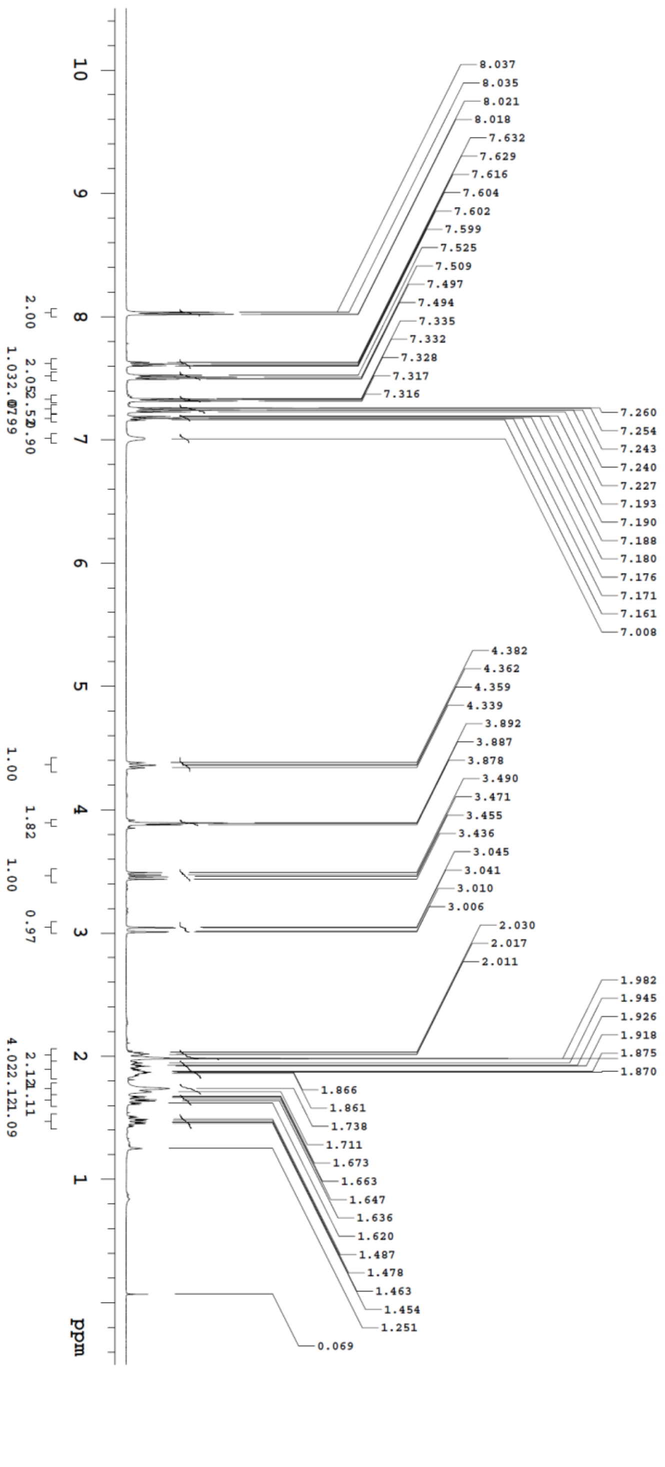

Supplementary Figure 70. ^{1}H NMR spectrum of *N*-((6-(2-oxo-2-phenylethyl)-2-phenyltetrahydro-2*H*-pyran-2-yl)methyl)acetamide (**9´**).

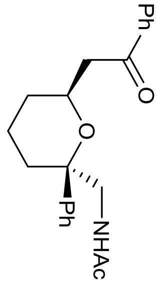

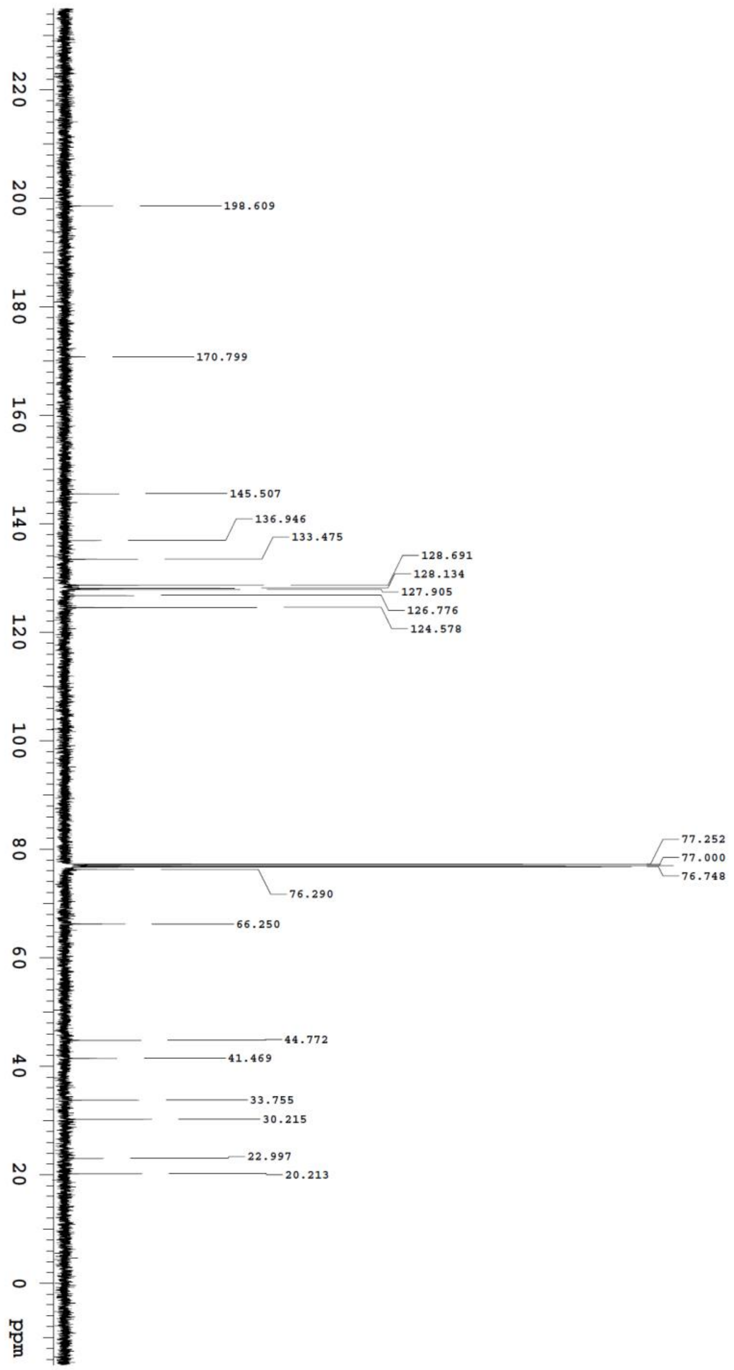

Supplementary Figure 71. ^{13}C NMR spectrum of *N*-((6-(2-oxo-2-phenylethyl)-2-phenyltetrahydro-2*H*-pyran-2-yl)methyl)acetamide (**9´**).

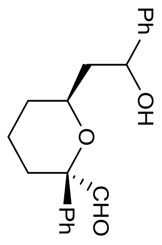

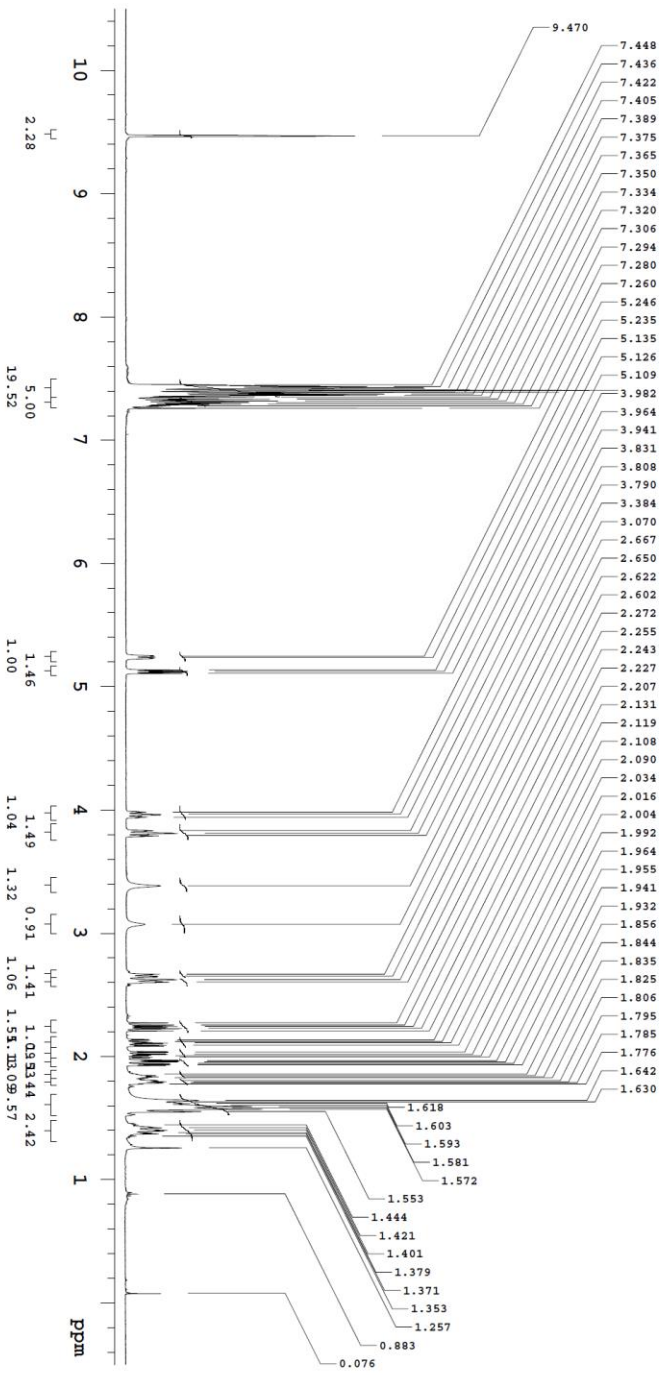

Supplementary Figure 72. ^{1}H NMR spectrum of 6-(2-hydroxy-2-phenylethyl)-2-phenyltetrahydro-2*H*-pyran-2-carbaldehyde (**10**).

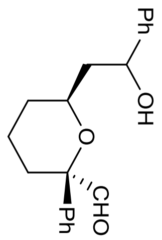

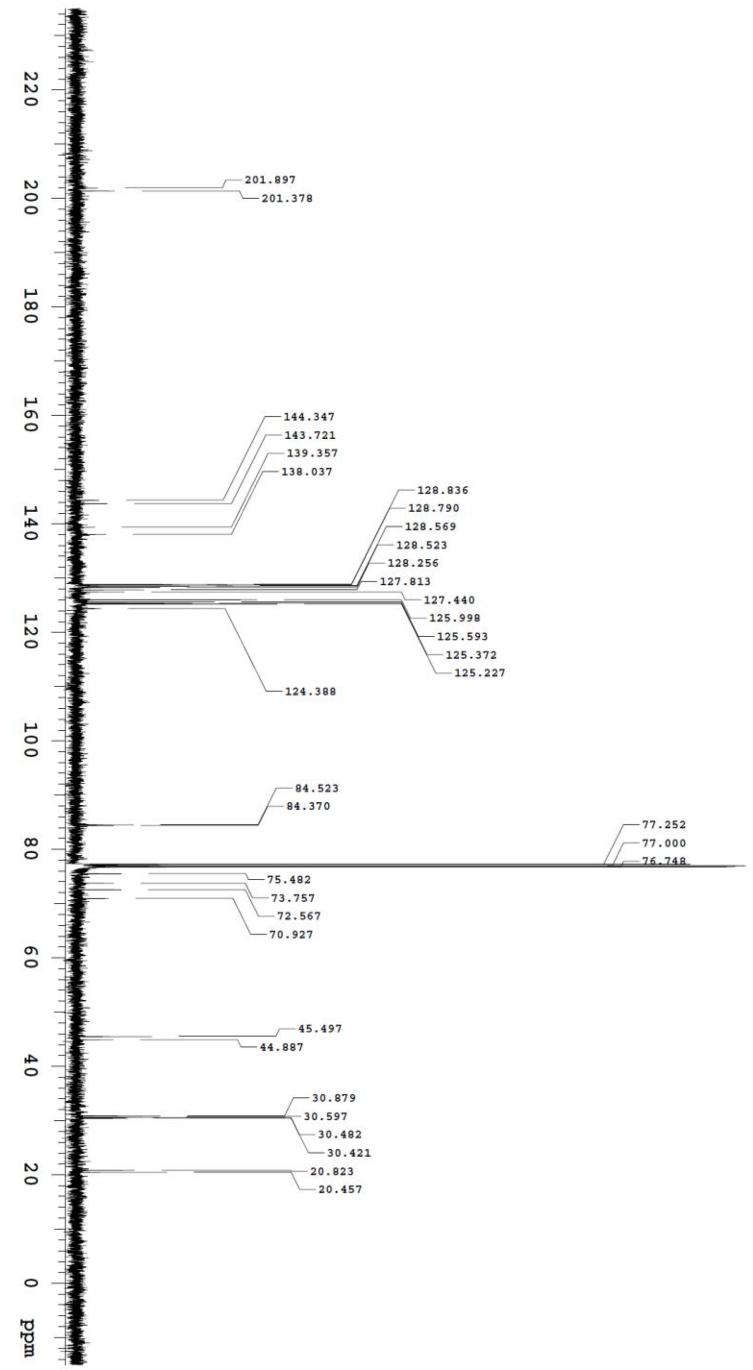

Supplementary Figure 73. ^{13}C NMR spectrum of 6-(2-hydroxy-2-phenylethyl)-2-phenyltetrahydro-2*H*-pyran-2-carbaldehyde (**10**).

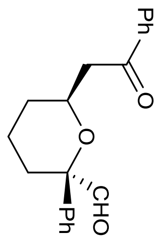

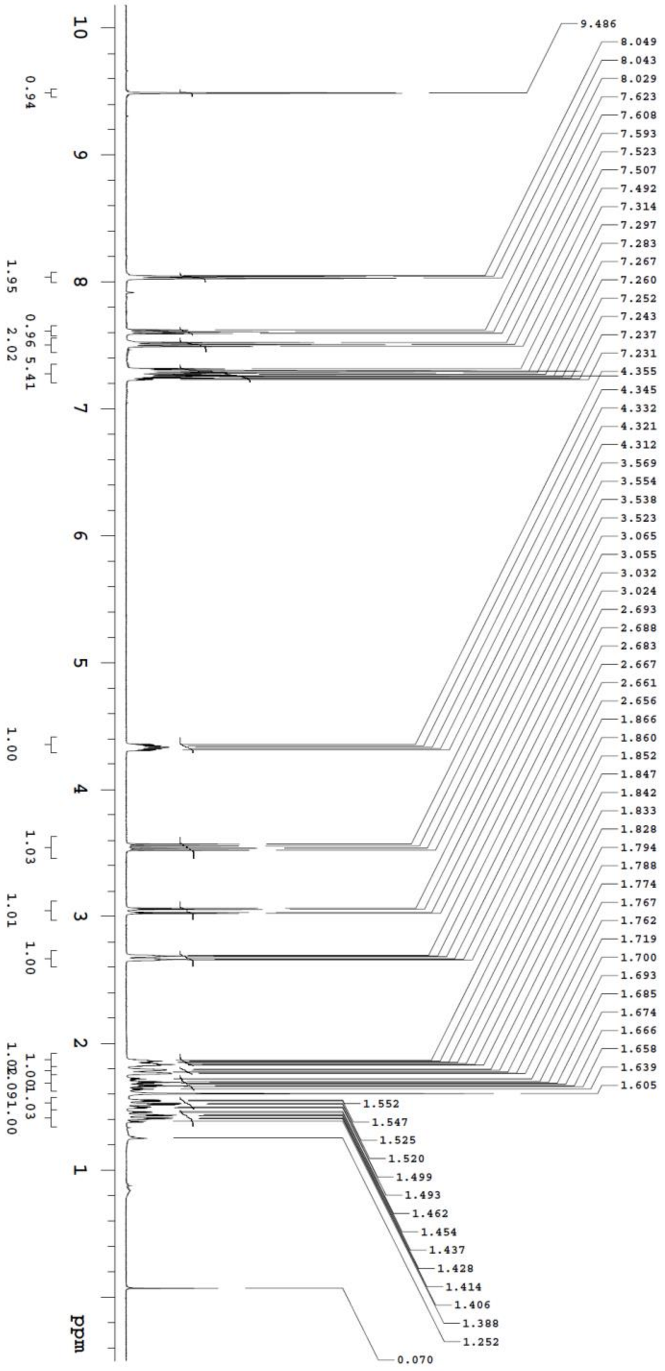

Supplementary Figure 74. ¹H NMR spectrum of 6-(2-oxo-2-phenylethyl)-2-phenyltetrahydro-2*H*-pyran-2-carbaldehyde (**11**).

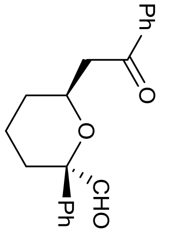

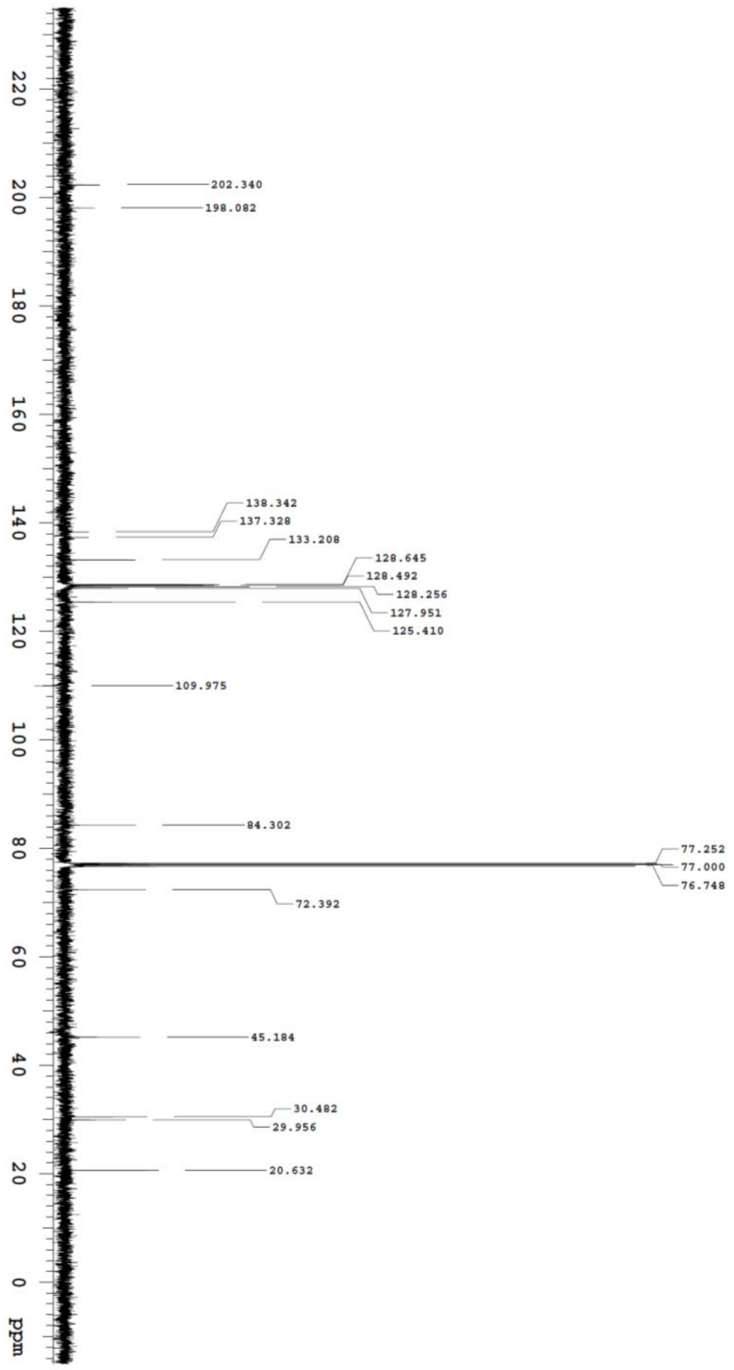

Supplementary Figure 75. ^{13}C NMR spectrum of 6-(2-oxo-2-phenylethyl)-2-phenyltetrahydro-2*H*-pyran-2-carbaldehyde (**11**).

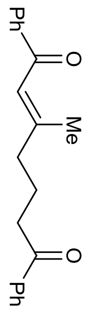

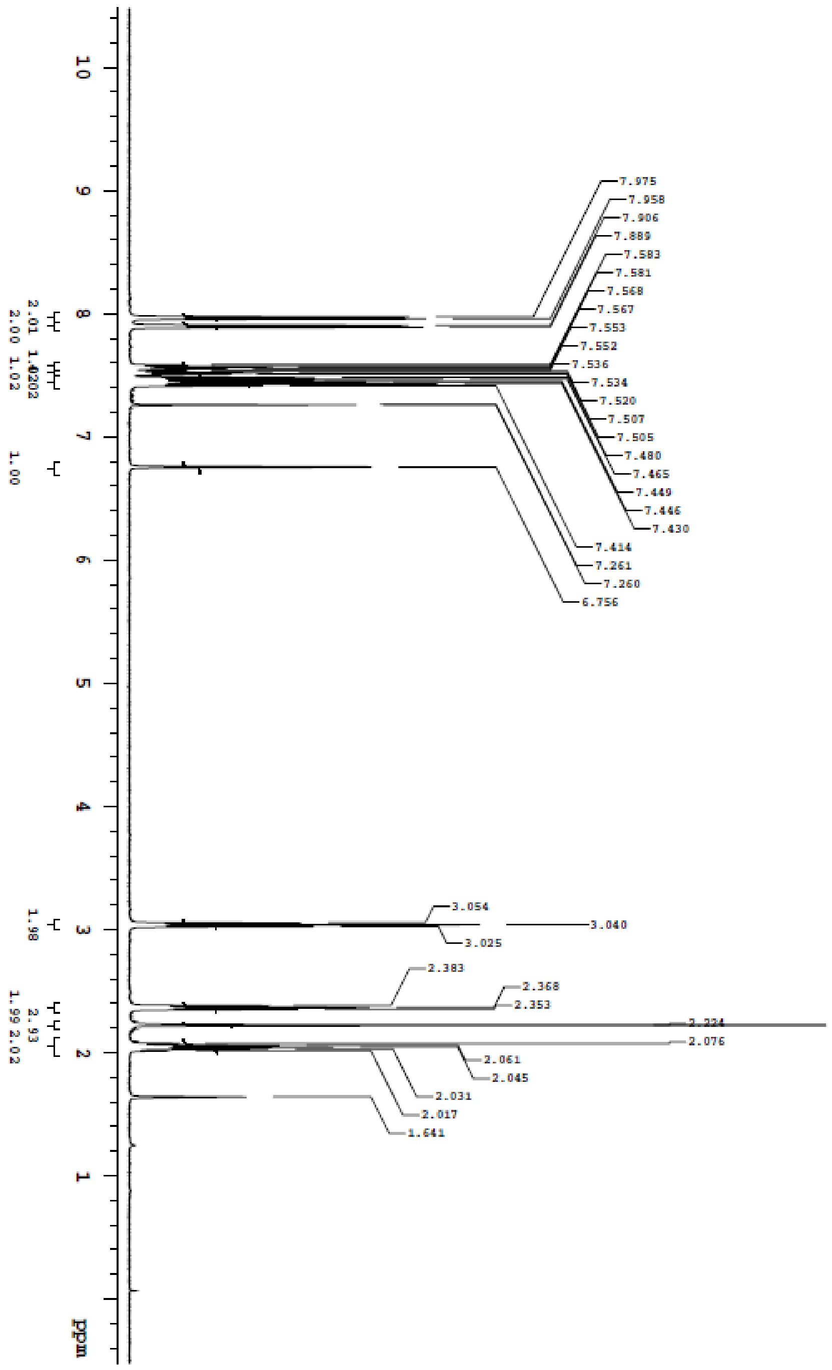

Supplementary Figure 76. ^{1}H NMR spectrum of (*E*)-3-methyl-1,7-diphenylhept-2-ene-1,7-dione ((*E*)-**28**).

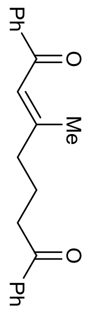

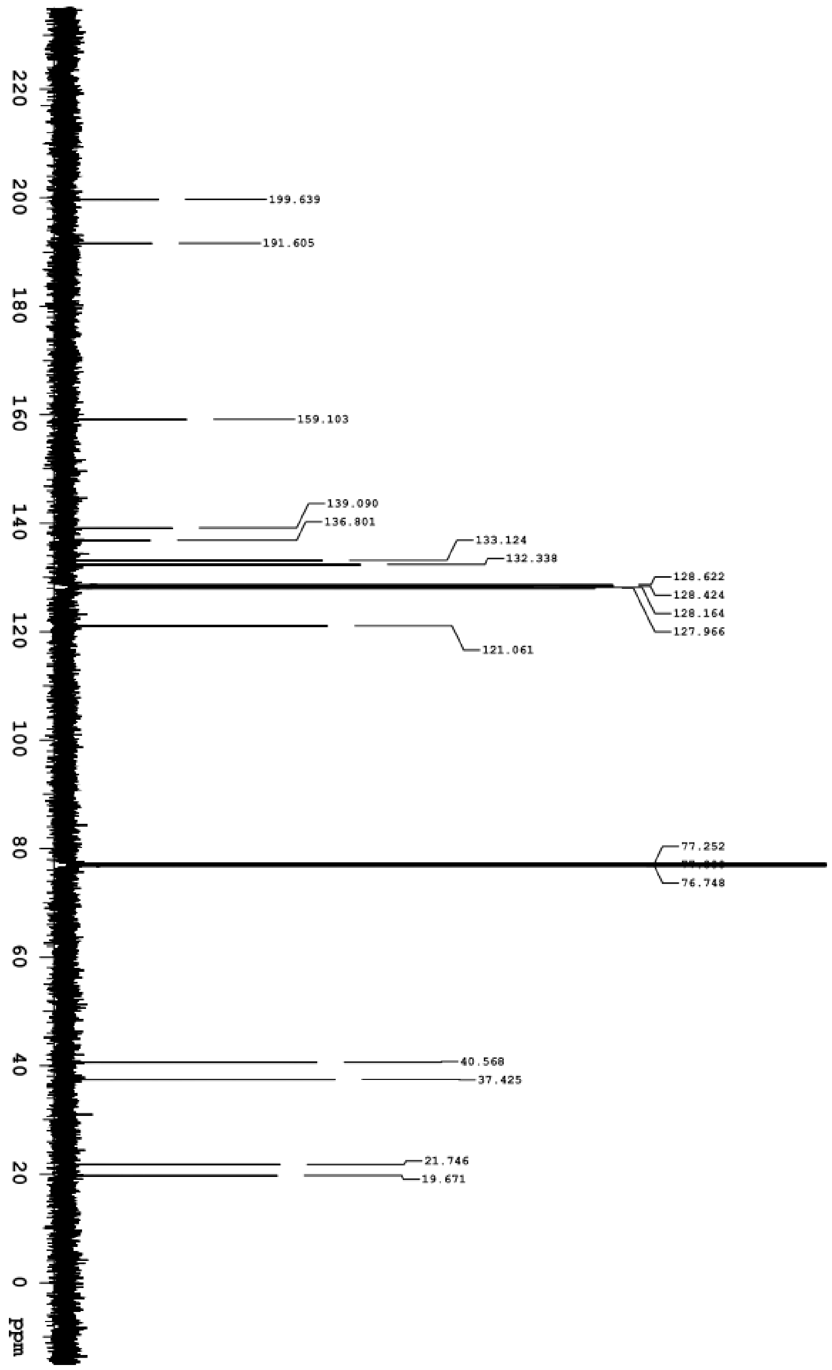

Supplementary Figure 77. ^{13}C NMR spectrum of (*E*)-3-methyl-1,7-diphenylhept-2-ene-1,7-dione ((*E*)-**28**).

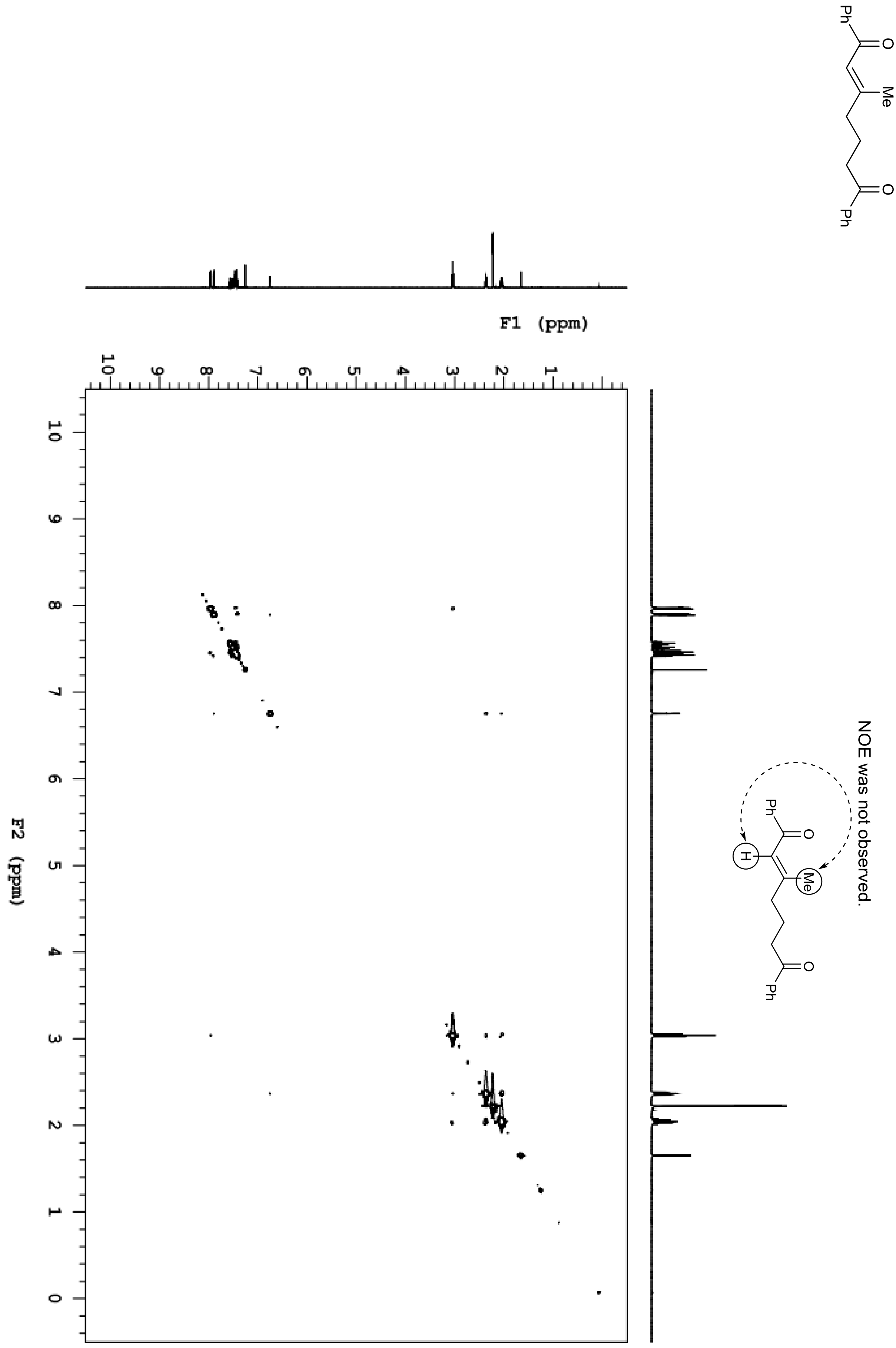

Supplementary Figure 78. NOESY spectrum of (*E*)-3-methyl-1,7-diphenylhept-2-ene-1,7-dione ((*E*)-**28**).

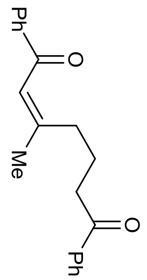

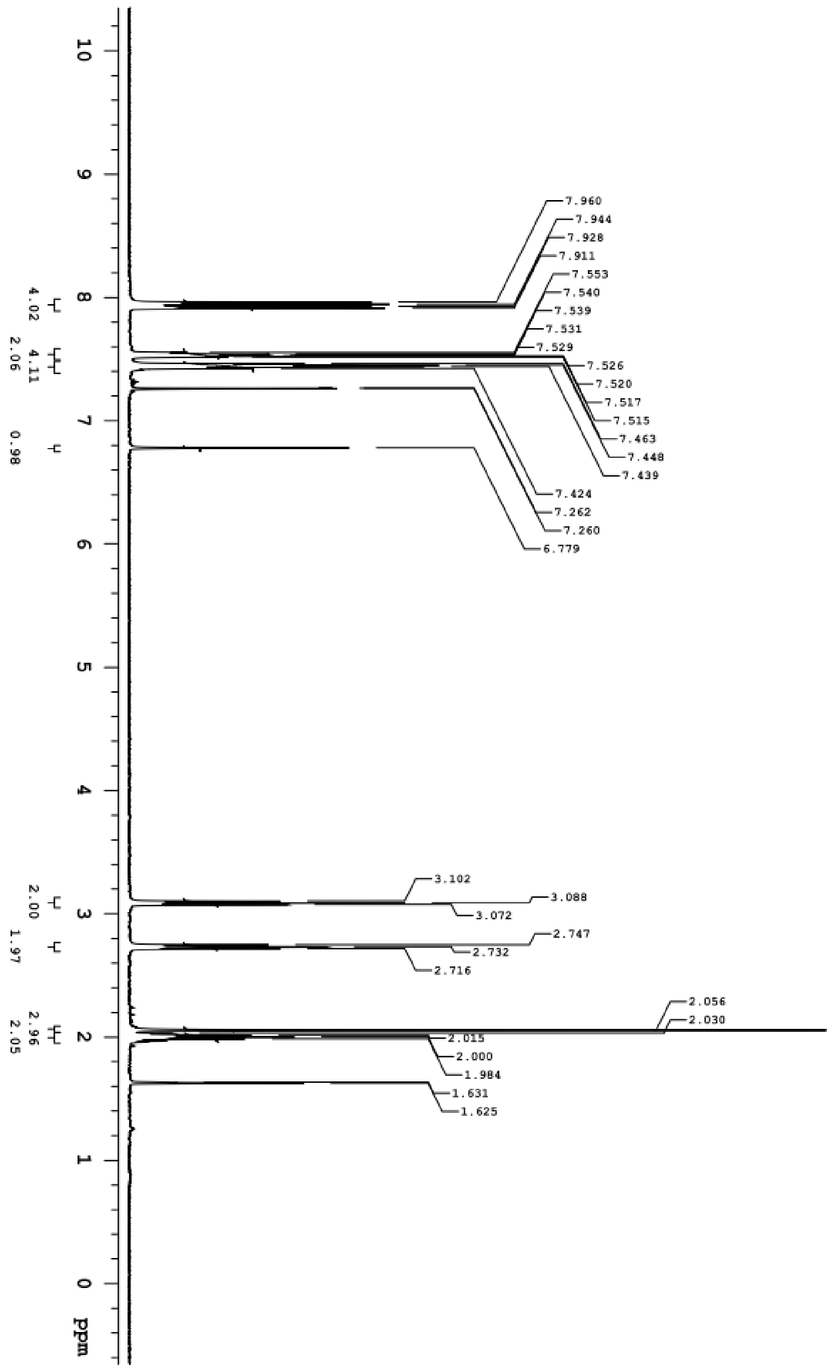

Supplementary Figure 79. ^{1}H NMR spectrum of (*Z*)-3-methyl-1,7-diphenylhept-2-ene-1,7-dione ((*Z*)-**28**).

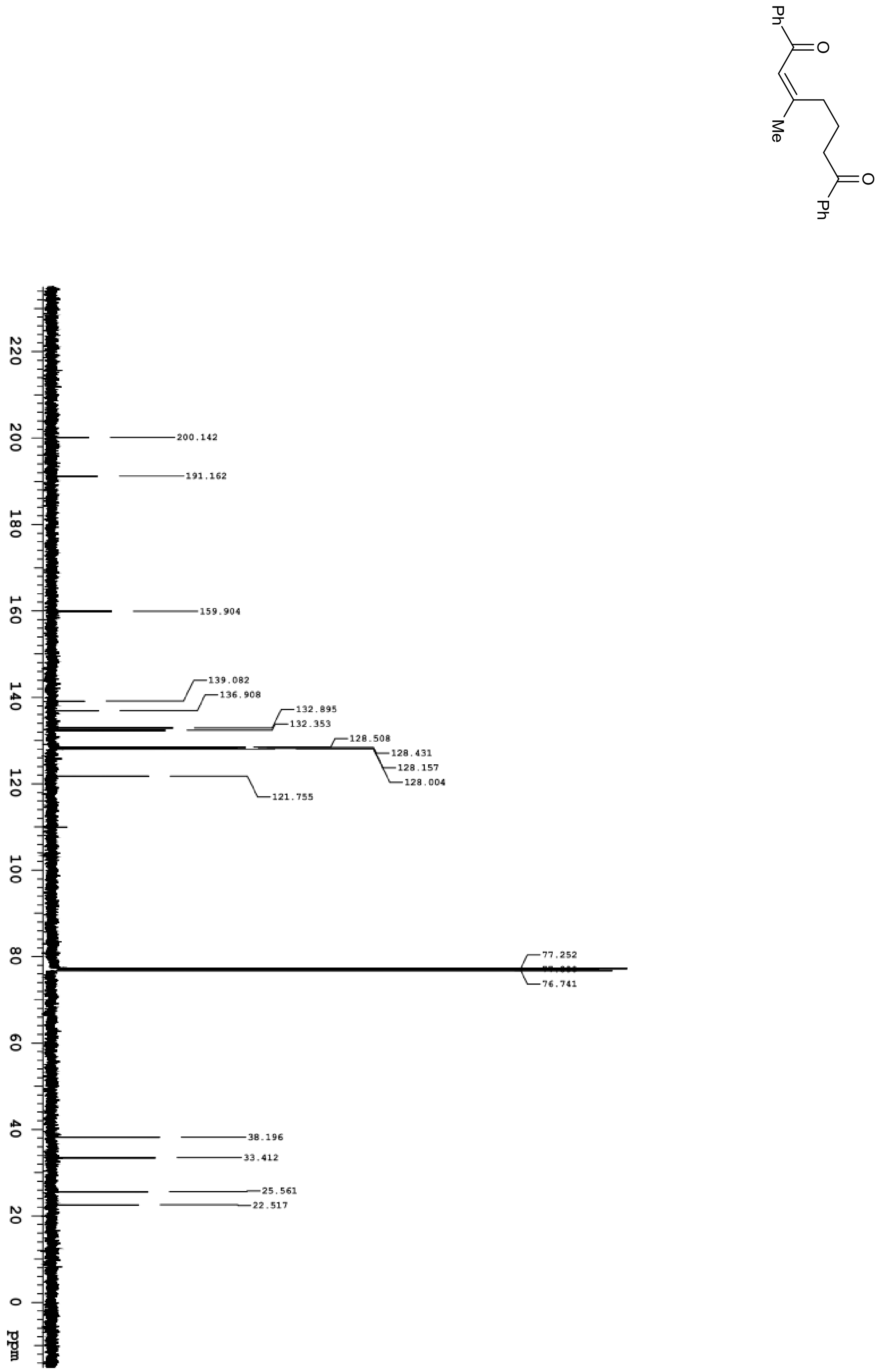

Supplementary Figure 80. ^{13}C NMR spectrum of (*Z*)-3-methyl-1,7-diphenylhept-2-ene-1,7-dione ((*Z*)-**28**).

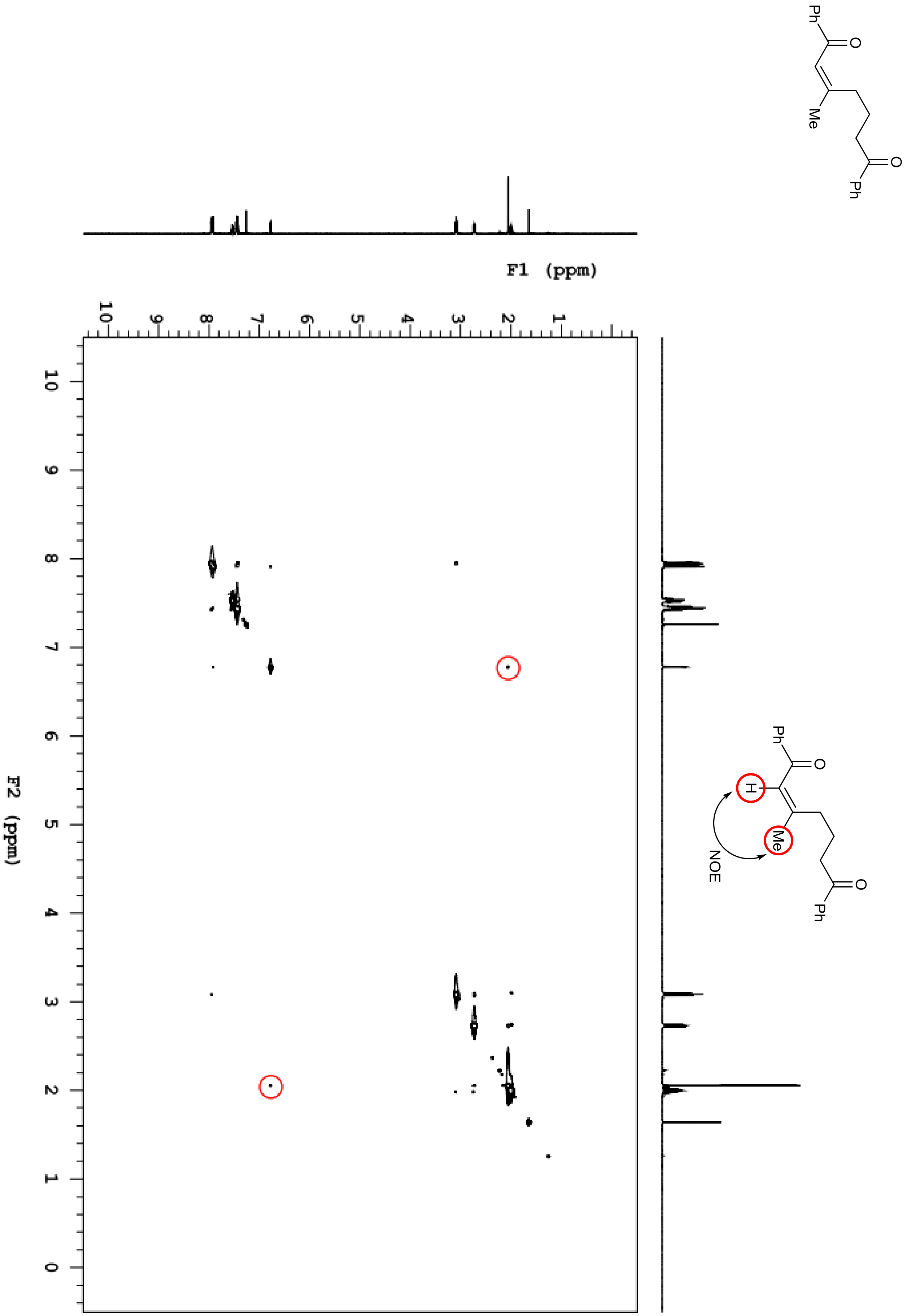

Supplementary Figure 81. NOESY spectrum of (*Z*)-3-methyl-1,7-diphenylhept-2-ene-1,7-dione ((*Z*)-**28**).

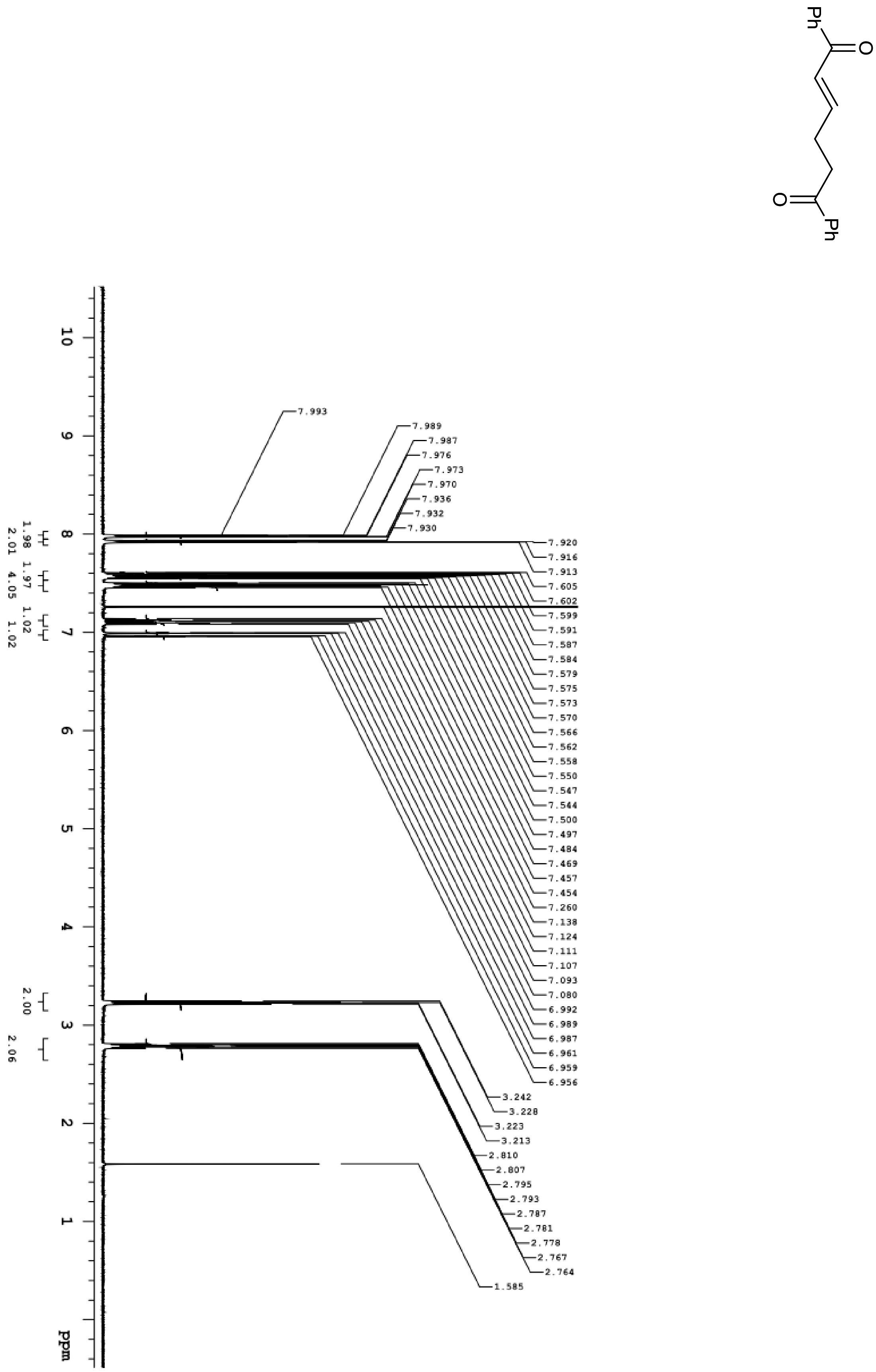

Supplementary Figure 82. ^{1}H NMR spectrum of (*E*)-1,6-diphenylhex-2-ene-1,6-dione (**32**).

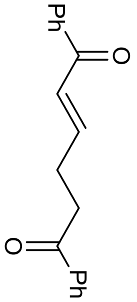

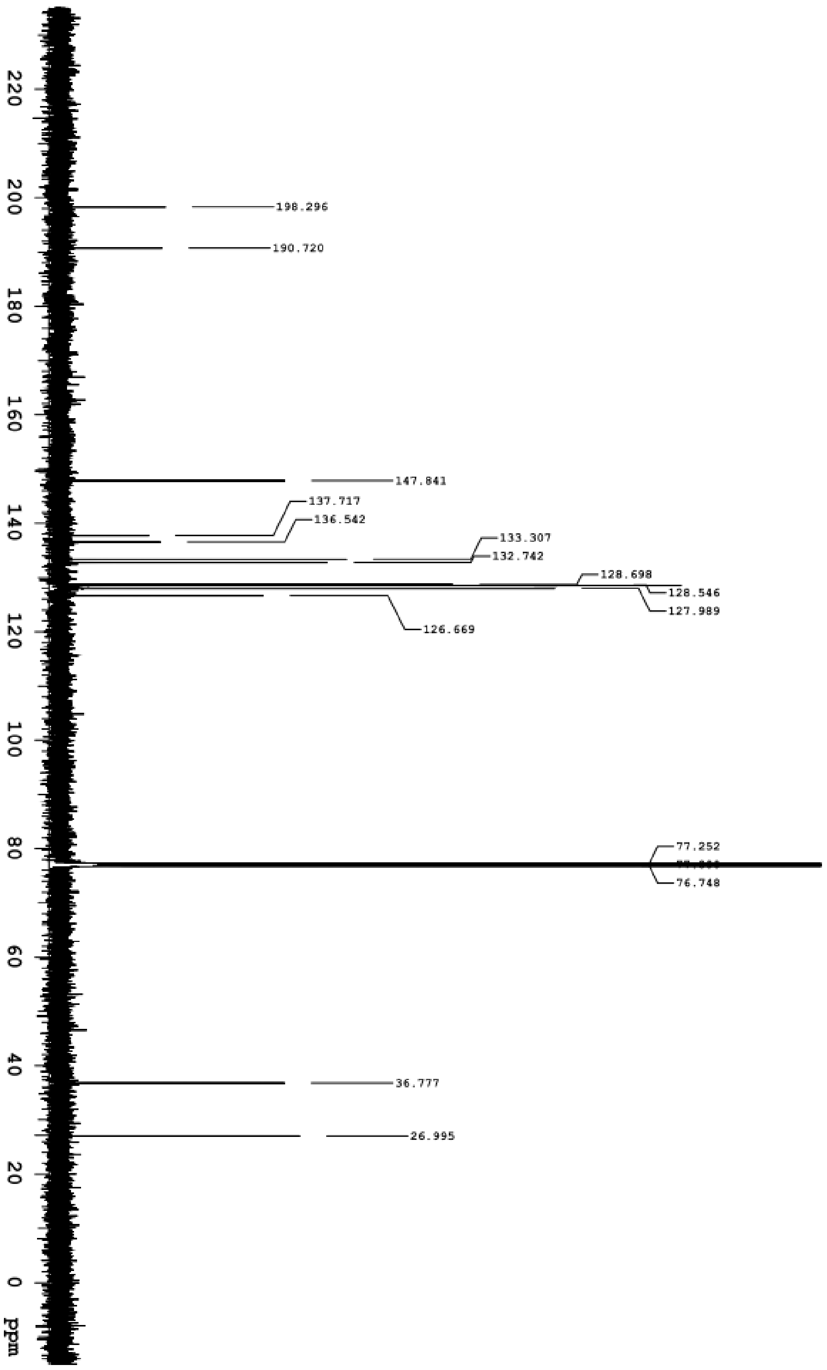

Supplementary Figure 83. ^{13}C NMR spectrum of (*E*)-1,6-diphenylhex-2-ene-1,6-dione (**32**).

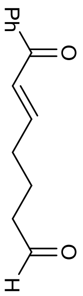

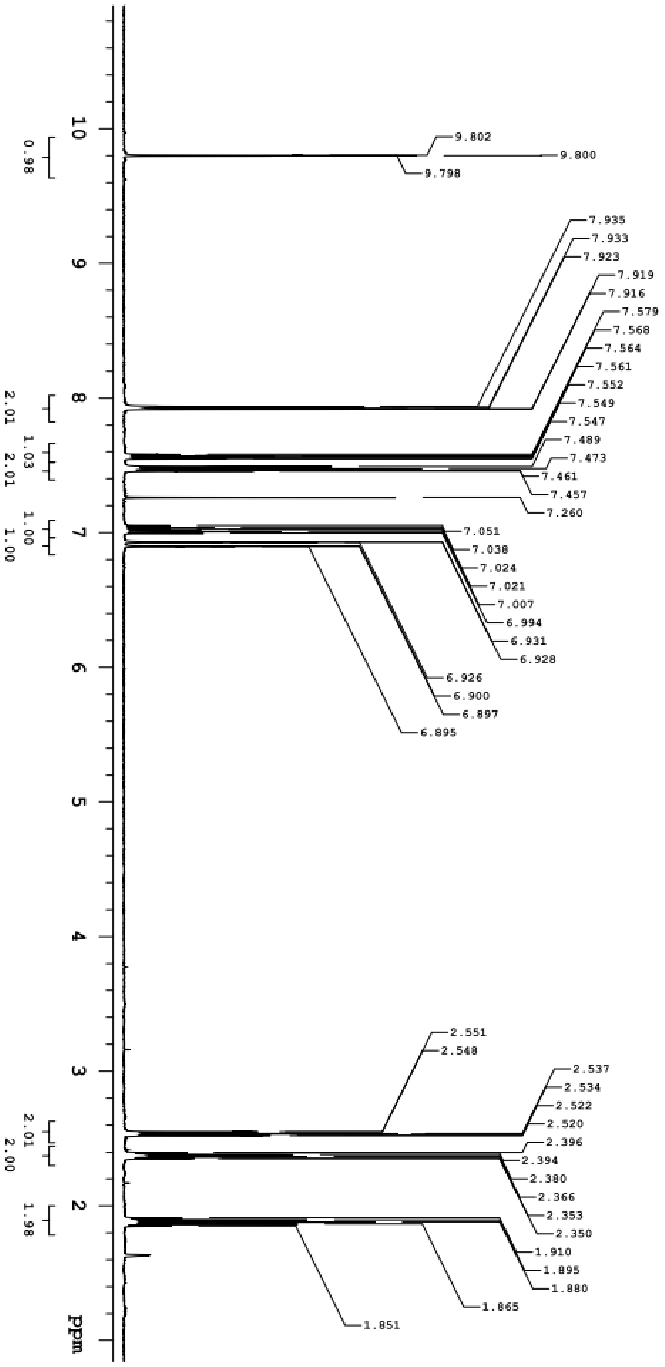

Supplementary Figure 84. ^{1}H NMR spectrum of (*E*)-7-oxo-7-phenylhept-5-enal (**34**).

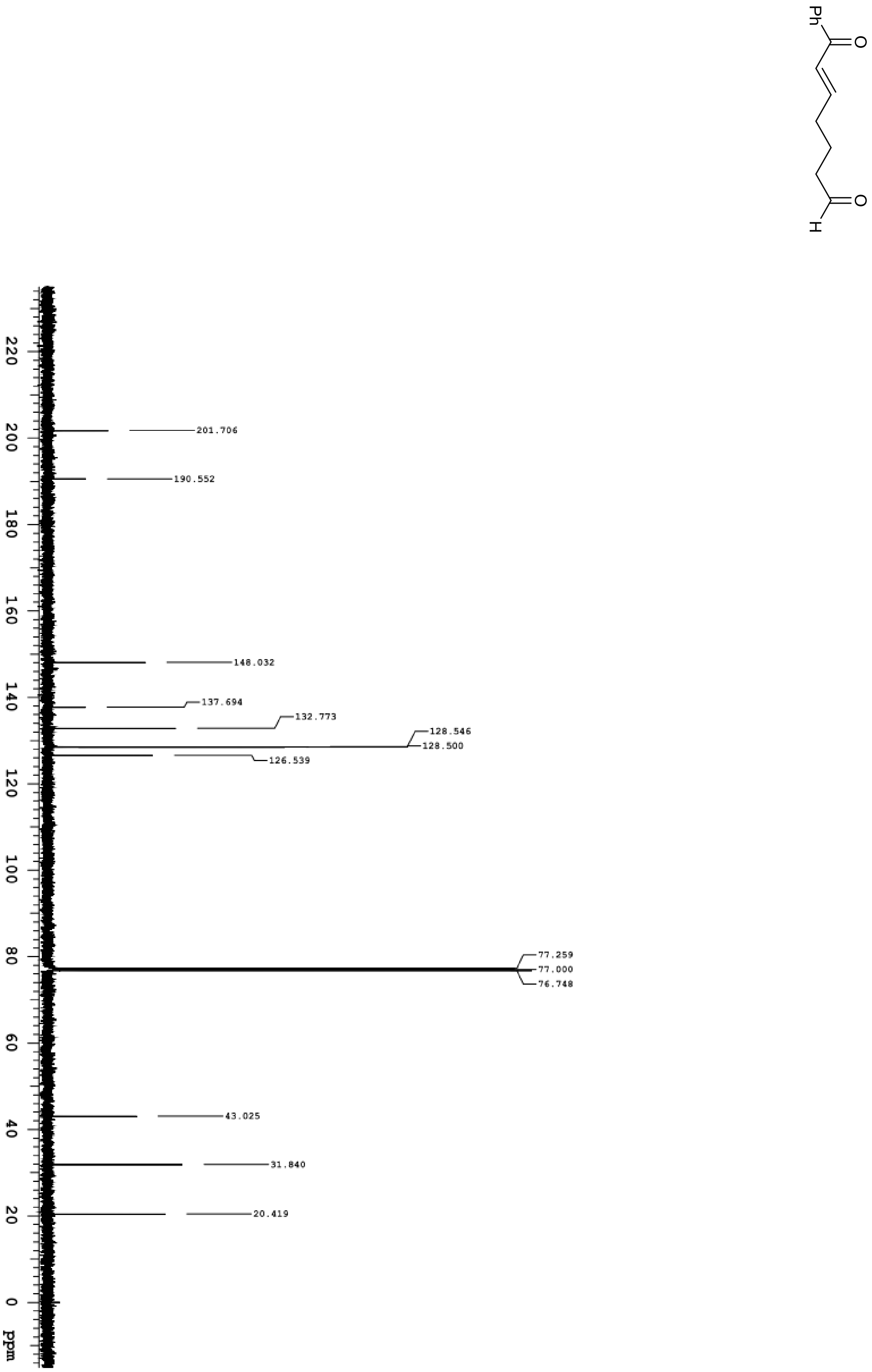

Supplementary Figure 85. ^{13}C NMR spectrum of (*E*)-7-oxo-7-phenylhept-5-enal (**34**).

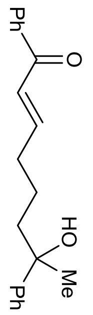

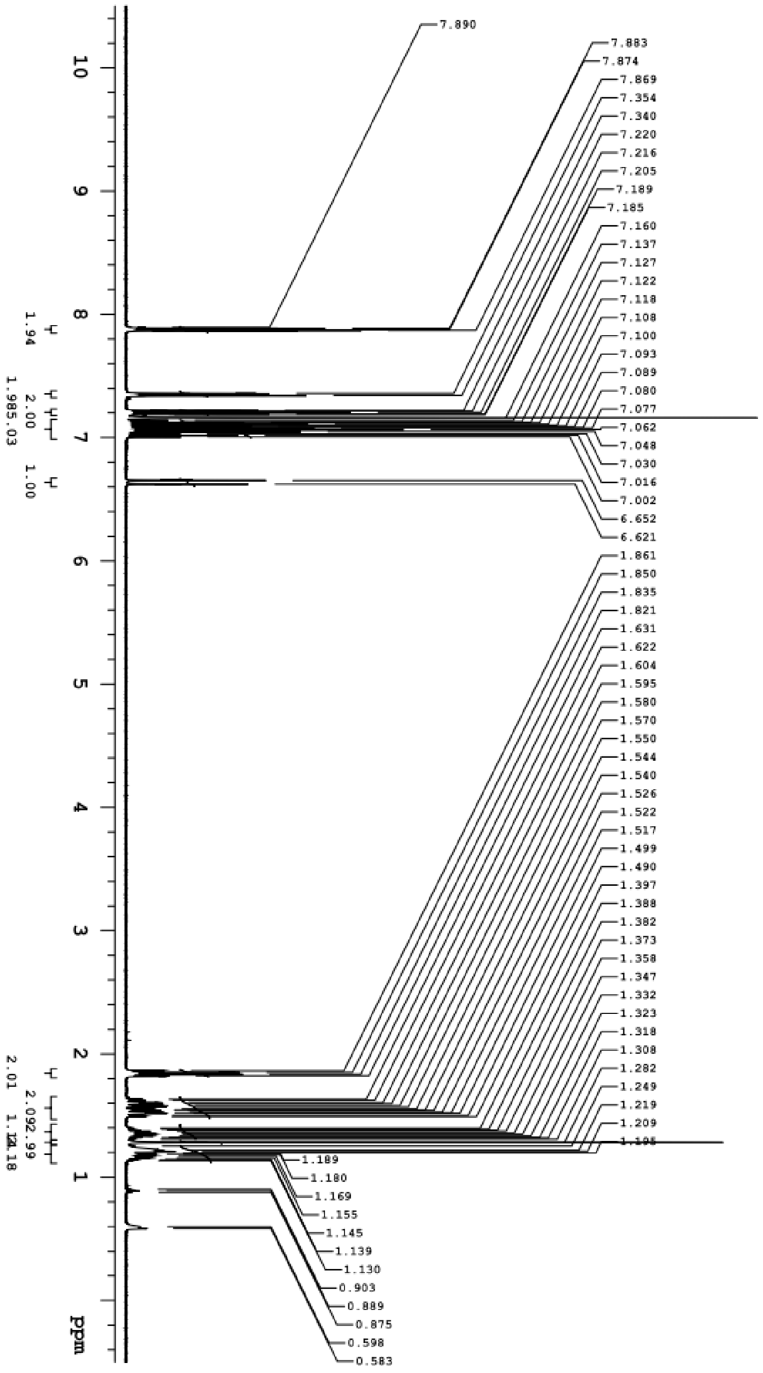

Supplementary Figure 86. ^{1}H NMR spectrum of (*E*)-7-hydroxy-1,7-diphenyloct-2-en-1-one (**37**).

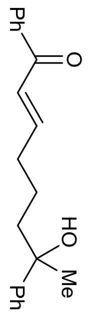

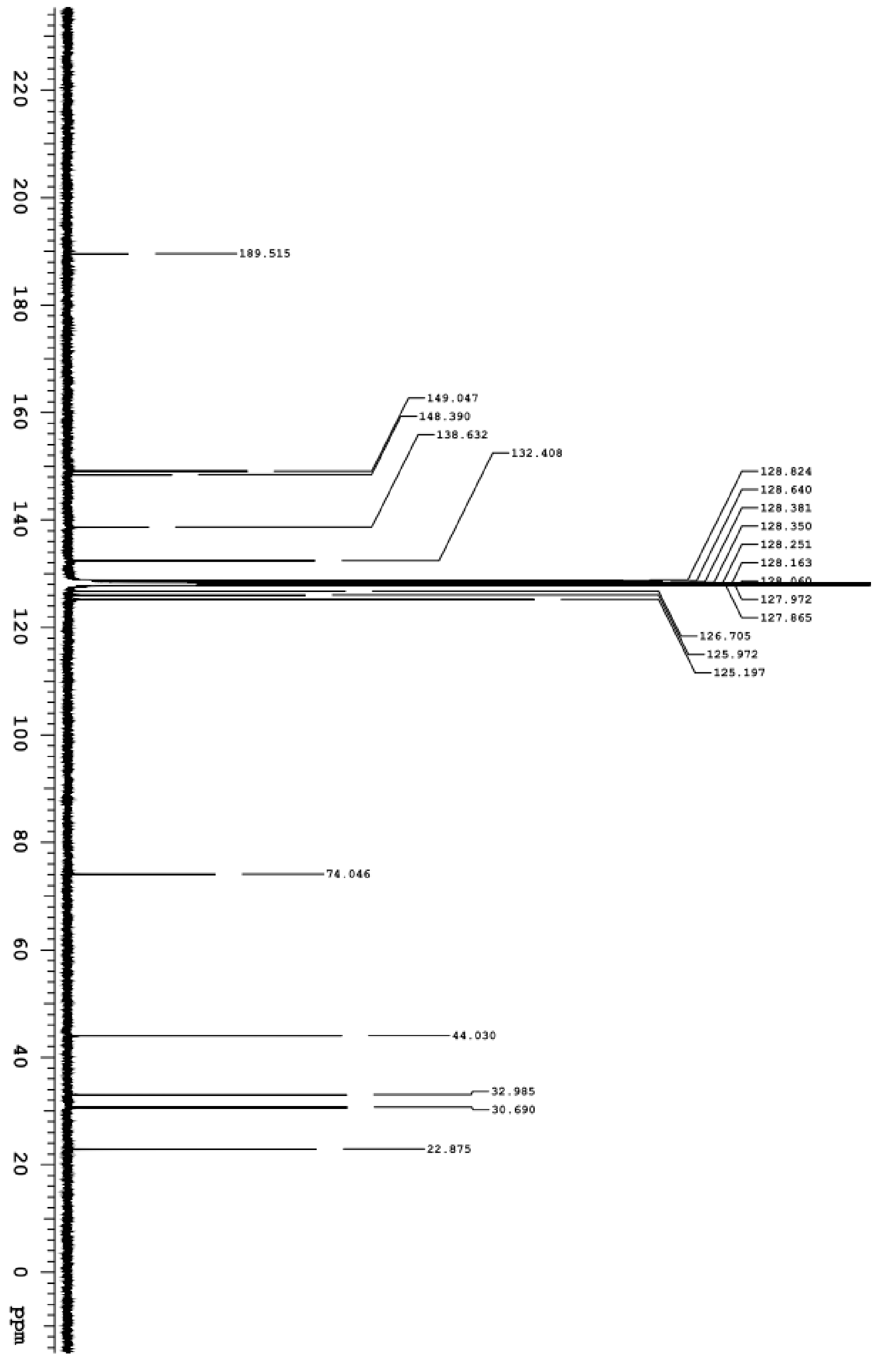

Supplementary Figure 87. ^{13}C NMR spectrum of (*E*)-7-hydroxy-1,7-diphenyloct-2-en-1-one (**37**)

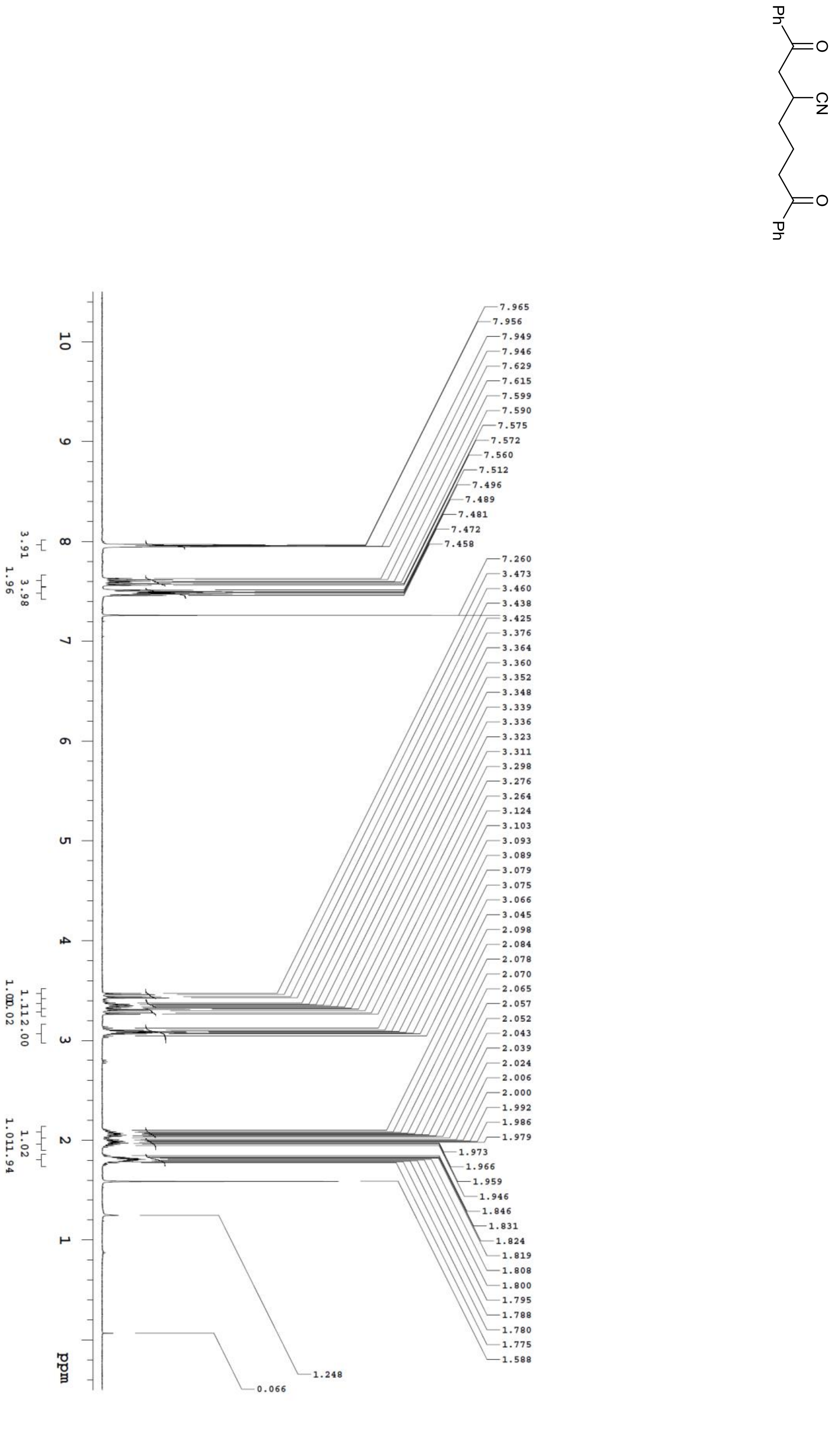

Supplementary Figure 88. ^{1}H NMR spectrum of 6-oxo-2-(2-oxo-2-phenylethyl)-6-phenylhexanenitrile (**38**).

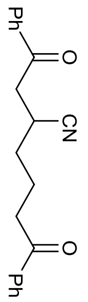

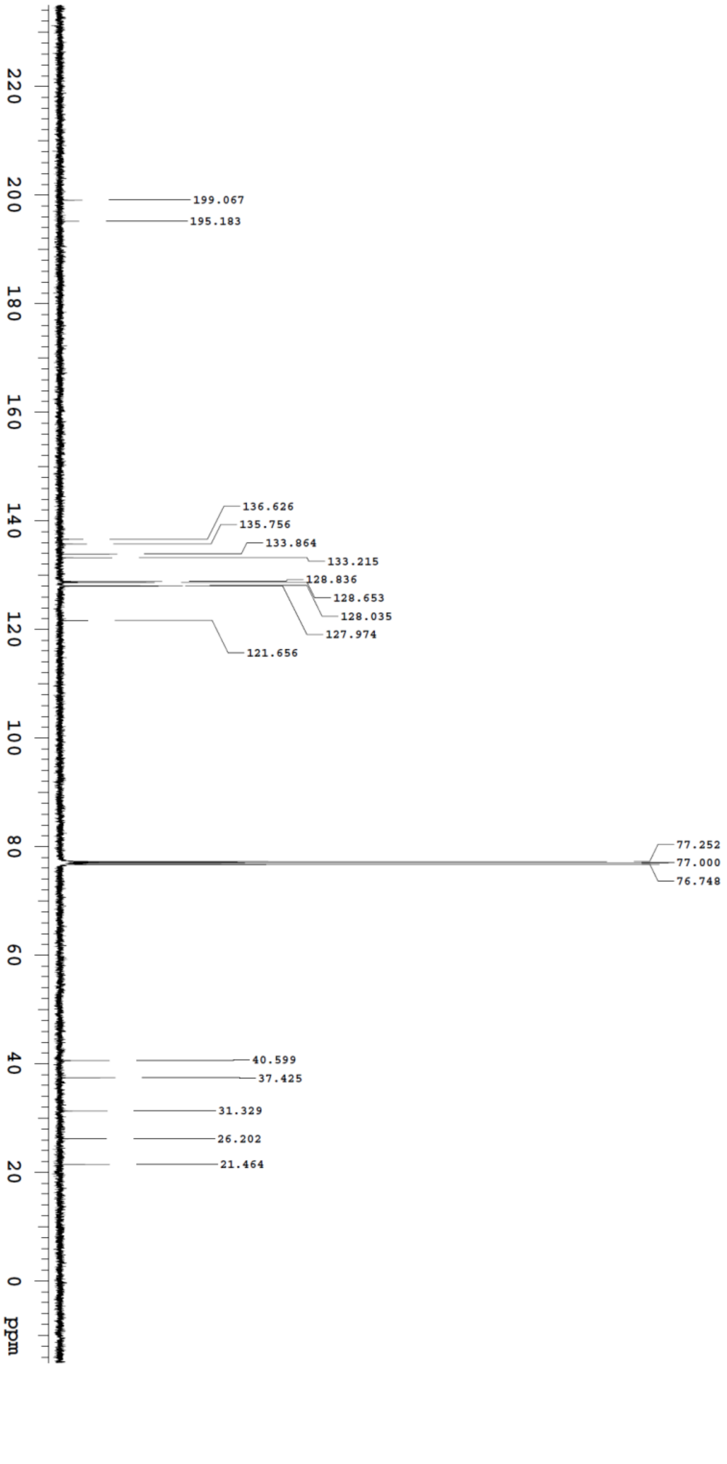

Supplementary Figure 89. ^{13}C NMR spectrum of 6-oxo-2-(2-oxo-2-phenylethyl)-6-phenylhexanenitrile (**38**).

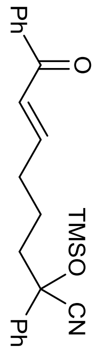

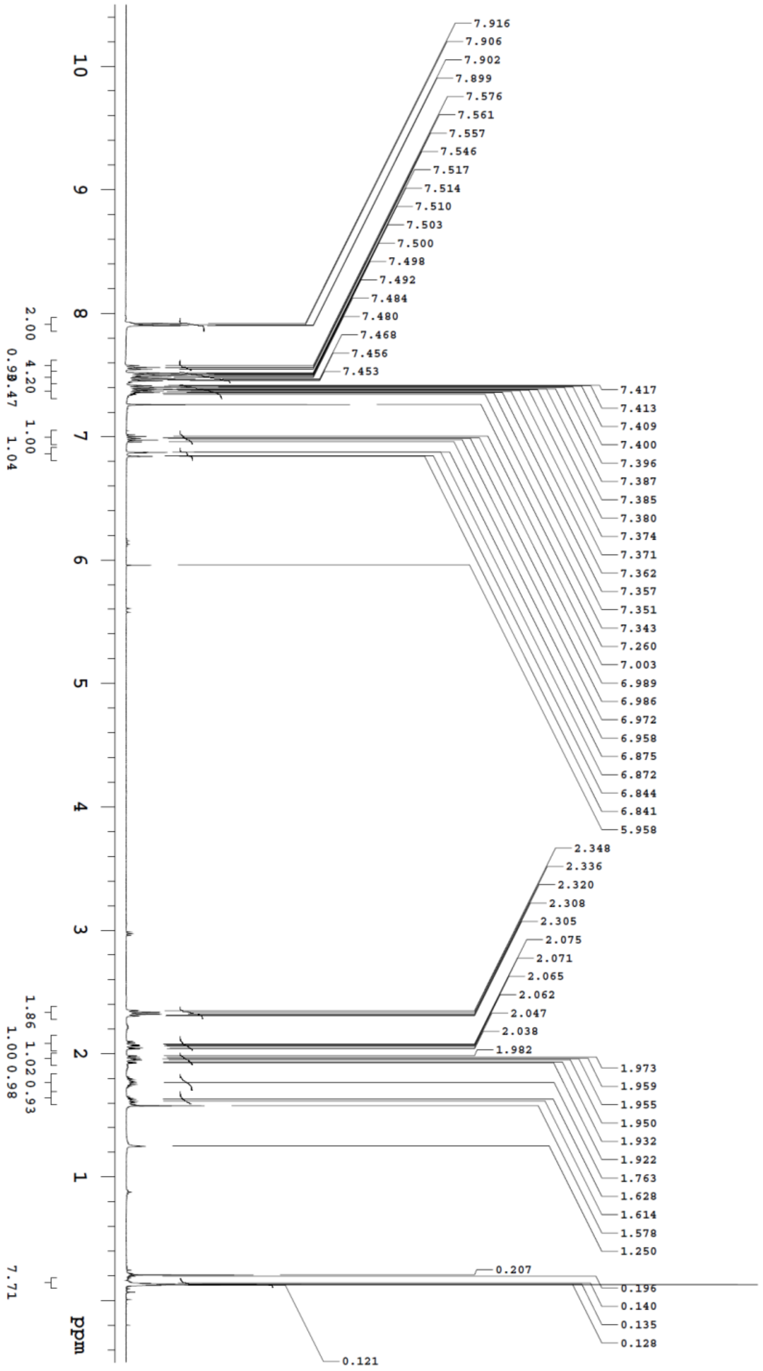

Supplementary Figure 90. ^{1}H NMR spectrum of (*E*)-8-oxo-2,8-diphenyl-2-((trimethylsilyl)oxy)oct-6-enenitrile (**39**).

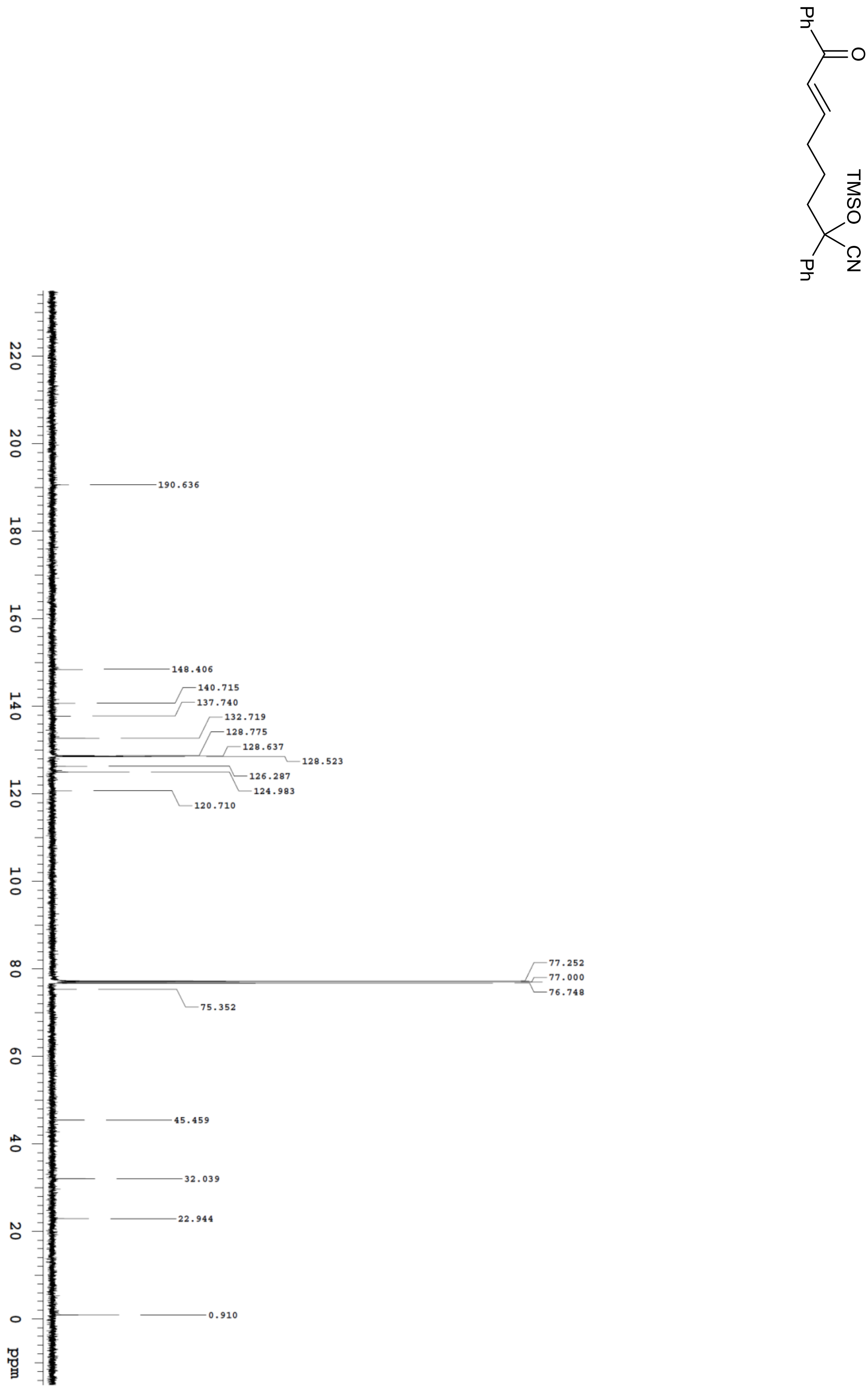

Supplementary Figure 91. ^{13}C NMR spectrum of (*E*)-8-oxo-2,8-diphenyl-2-((trimethylsilyl)oxy)oct-6-enenitrile (**39**).

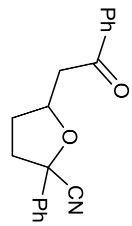

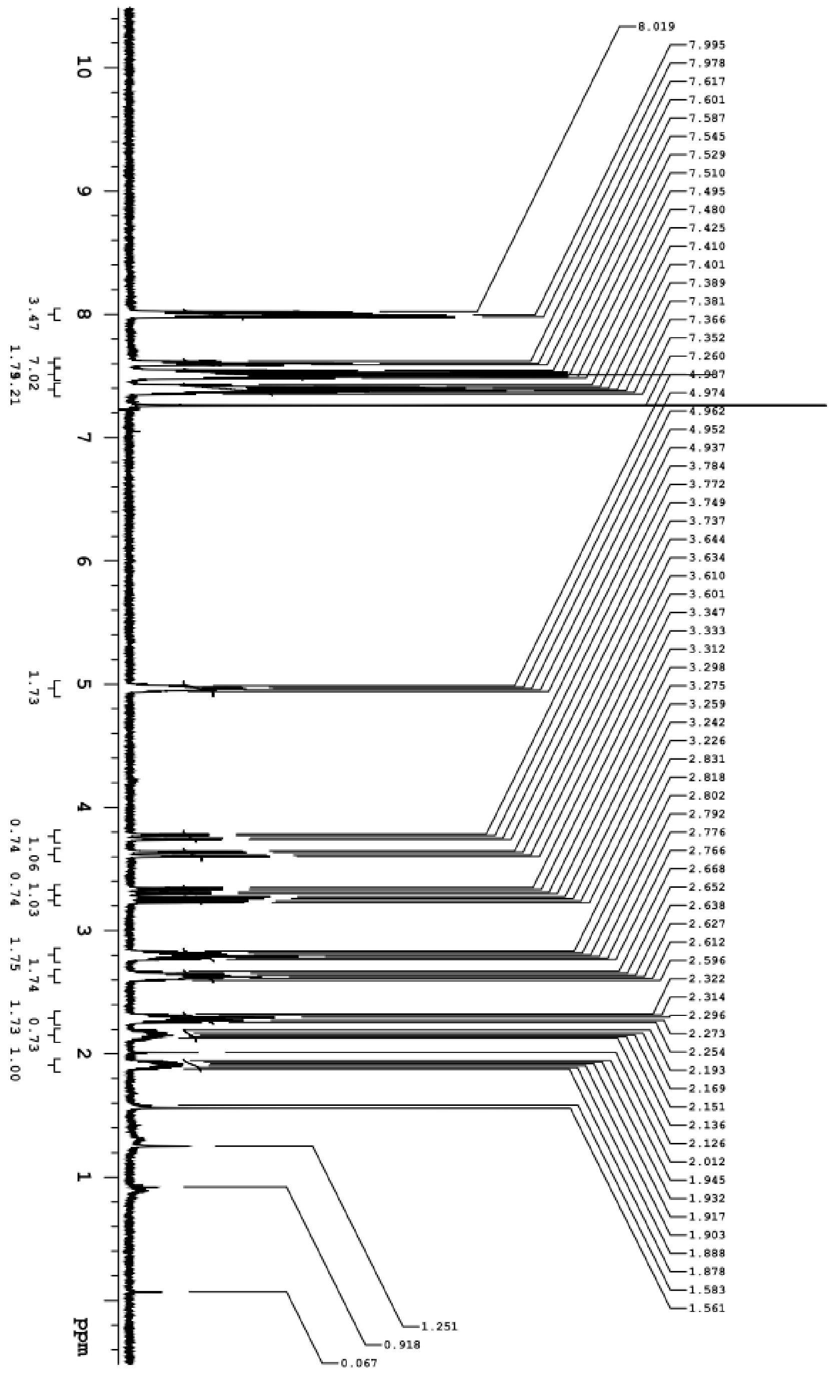

Supplementary Figure 92. ^{1}H NMR spectrum of 5-(2-oxo-2-phenylethyl)-2-phenyltetrahydrofuran-2-carbonitrile (**40**).

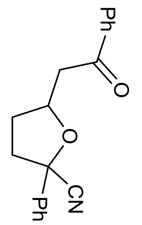

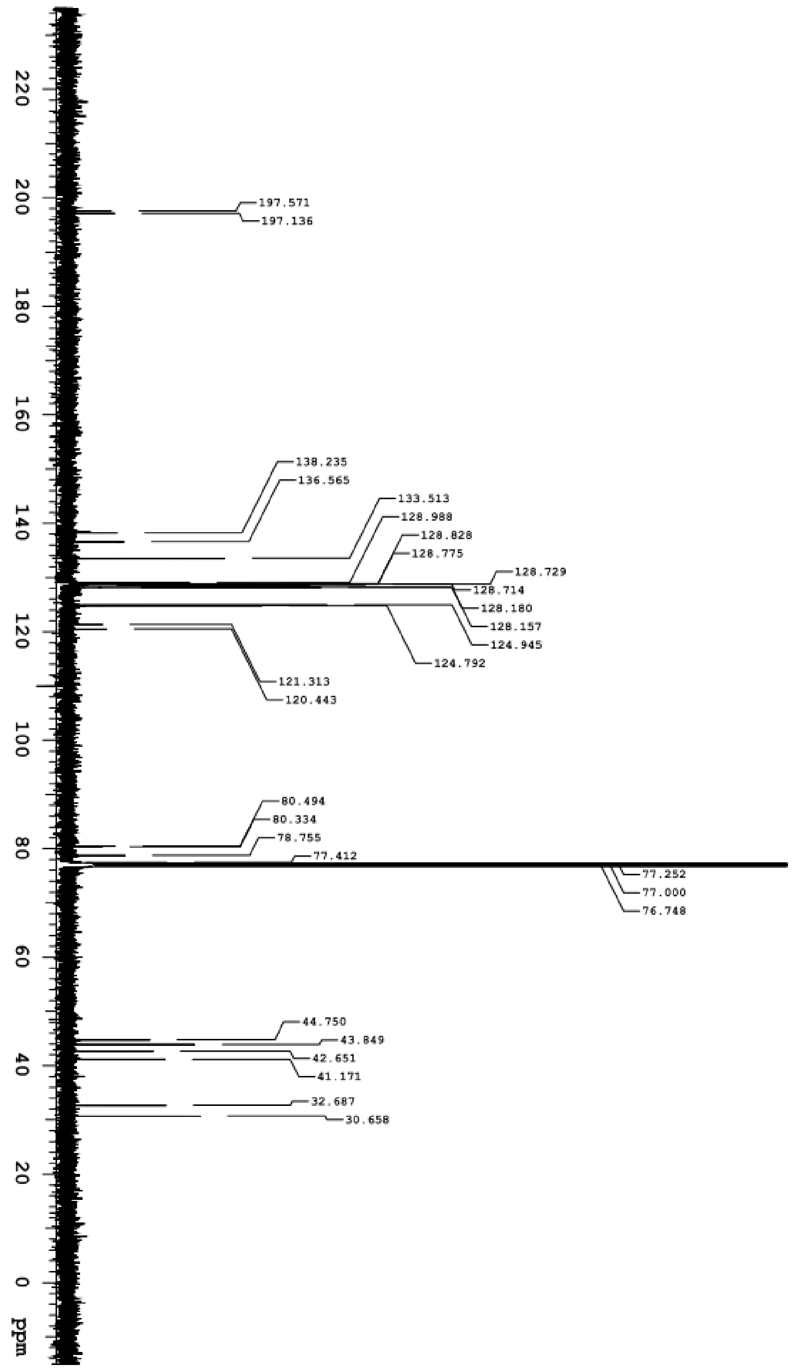

Supplementary Figure 93. ^{13}C NMR spectrum of 5-(2-oxo-2-phenylethyl)-2-phenyltetrahydrofuran-2-carbonitrile (**40**).

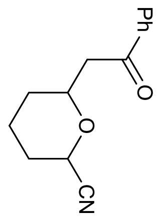

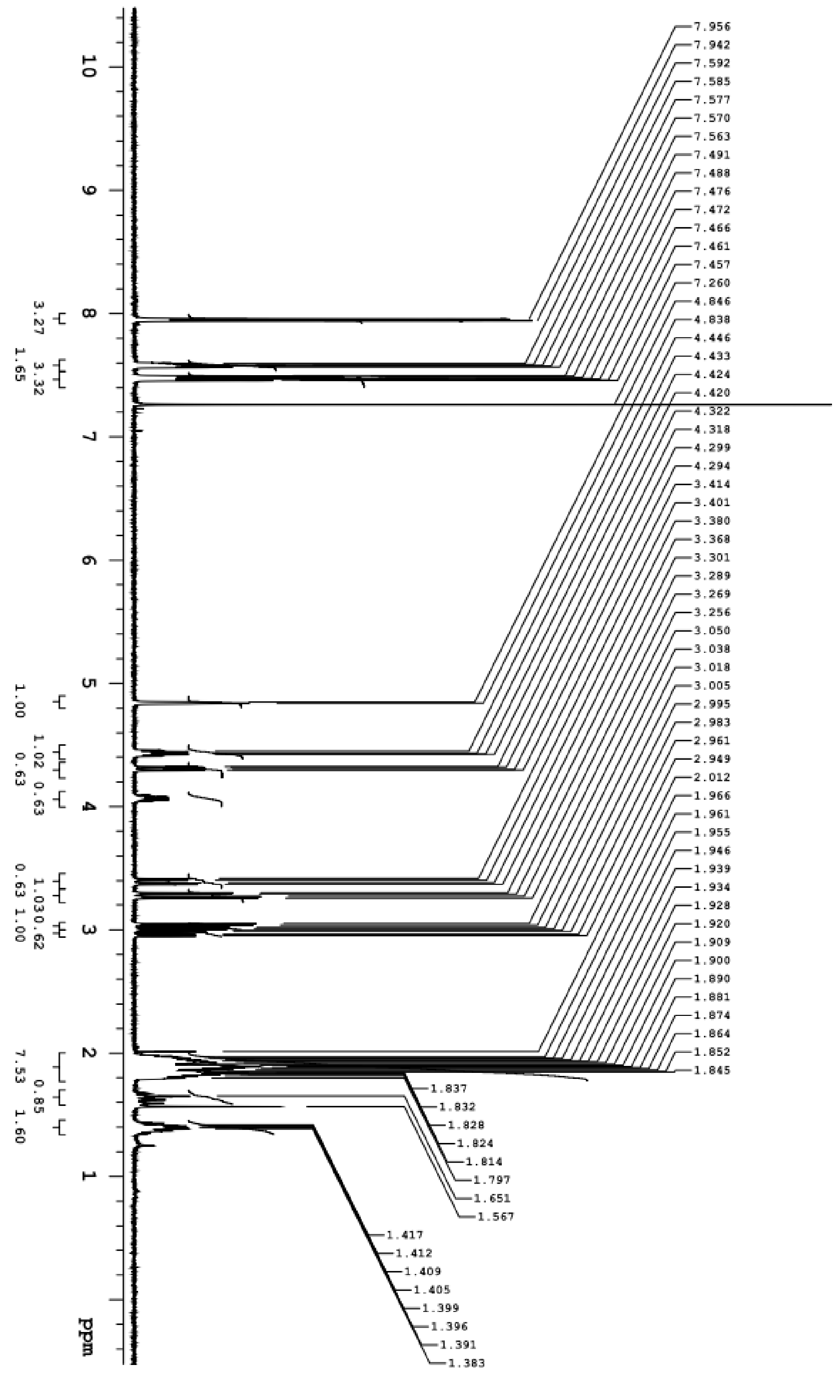

Supplementary Figure 94. ^{1}H NMR spectrum of 6-(2-oxo-2-phenylethyl)tetrahydro-2*H*-pyran-2-carbonitrile (**41**).

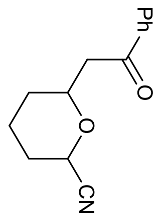

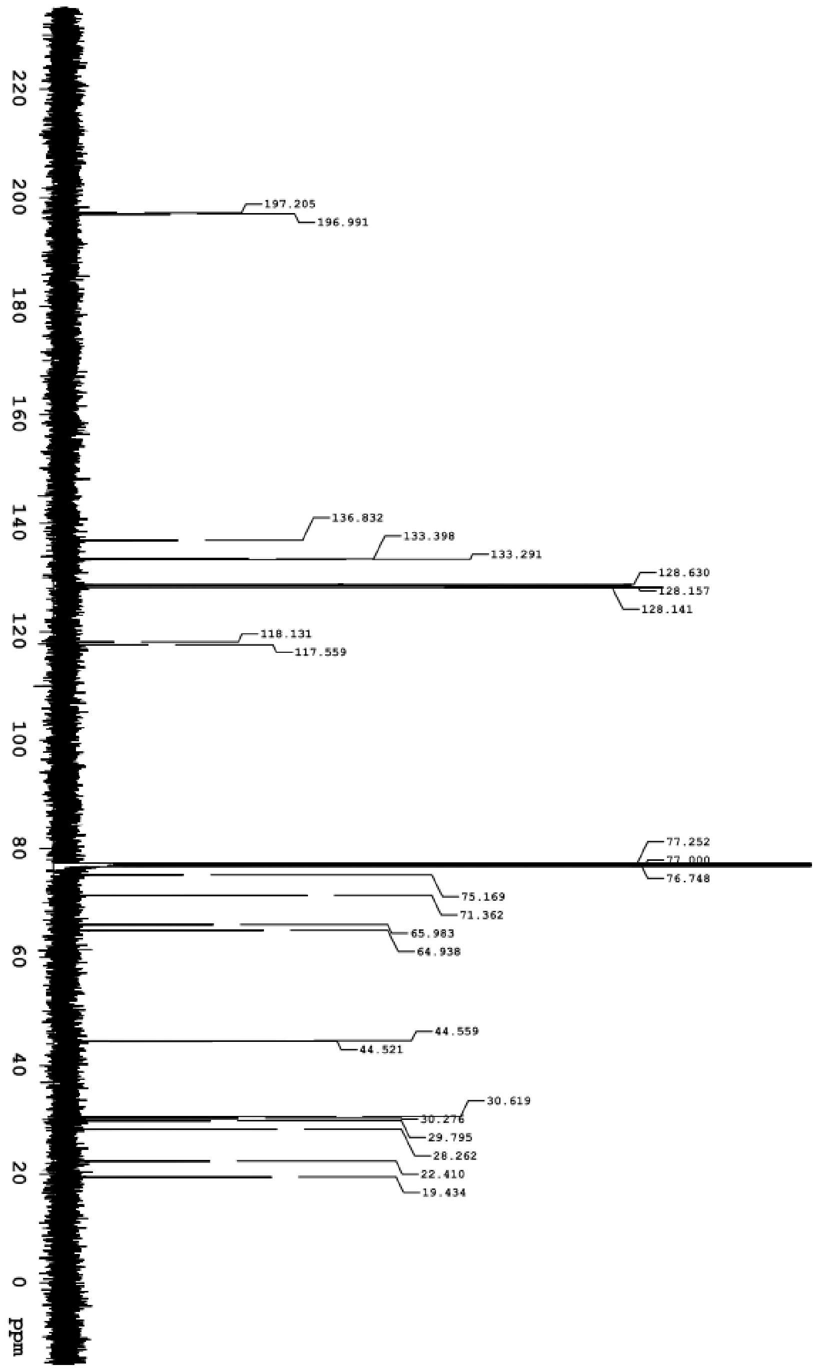

Supplementary Figure 95. ^{13}C NMR spectrum of 6-(2-oxo-2-phenylethyl)tetrahydro-2*H*-pyran-2-carbonitrile (**41**).

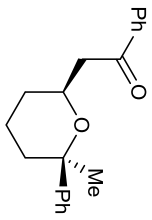

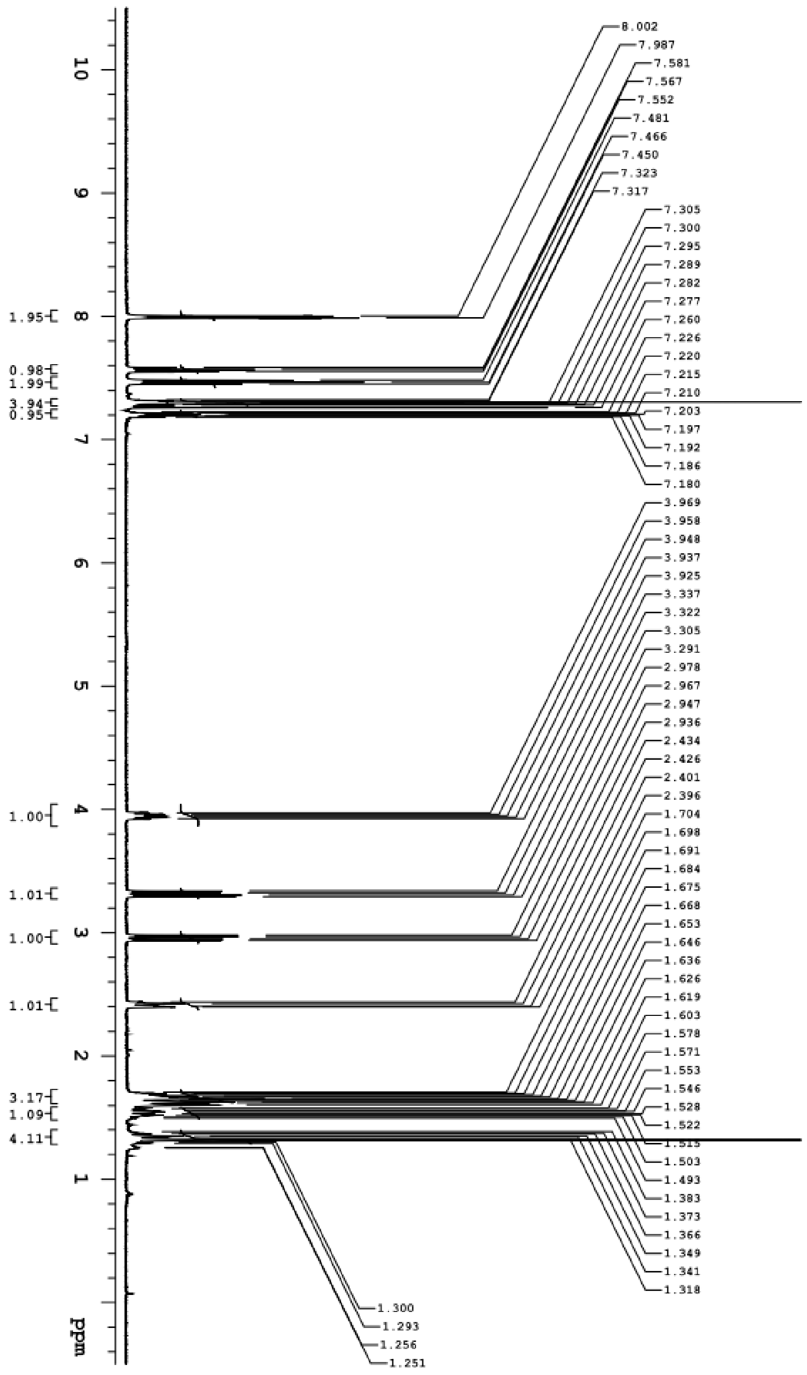

Supplementary Figure 96. ^{1}H NMR spectrum of 2-((2*S*,6*R*)-6-methyl-6-phenyltetrahydro-2*H*-pyran-2-yl)-1-phenylethan-1-one (**42**).

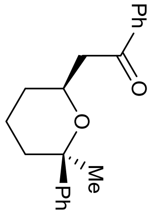

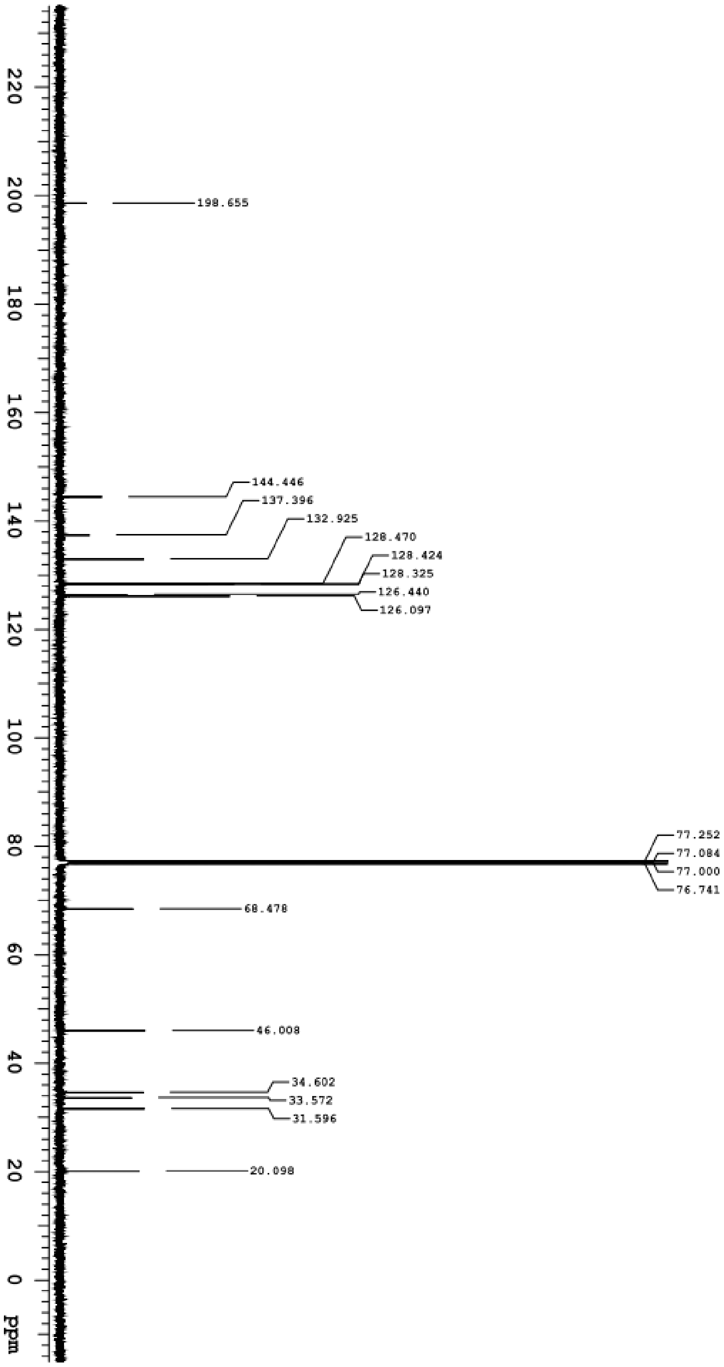

Supplementary Figure 97. ^{13}C NMR spectrum of 2-((2*S*,6*R*)-6-methyl-6-phenyltetrahydro-2*H*-pyran-2-yl)-1-phenylethan-1-one (**42**).

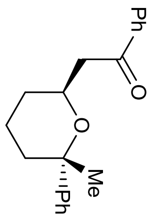

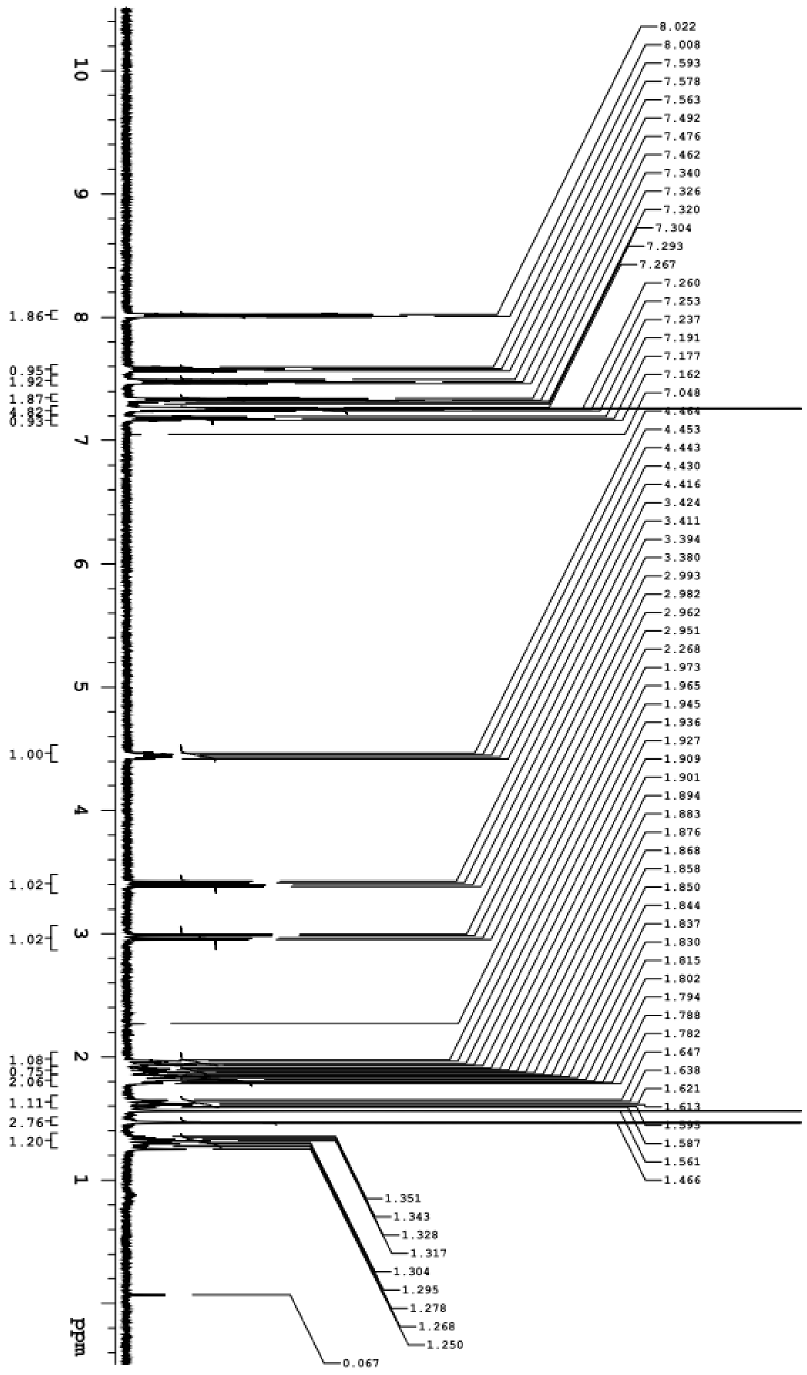

Supplementary Figure 98. ^{1}H NMR spectrum of 2-((2*S*,6*S*)-6-methyl-6-phenyltetrahydro-2*H*-pyran-2-yl)-1-phenylethan-1-one (**42'**).

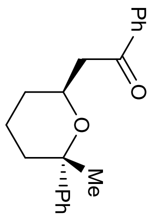

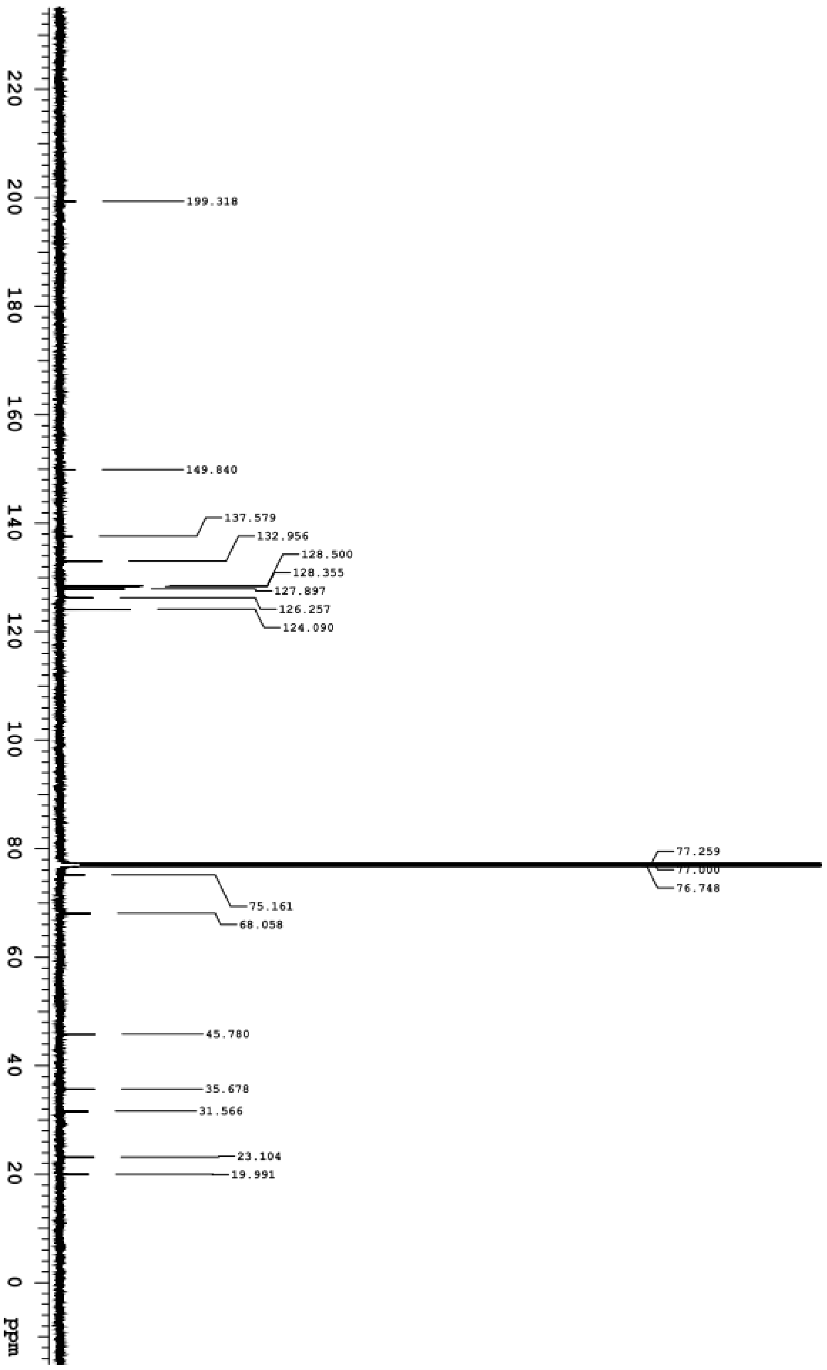

Supplementary Figure 99. ^{13}C NMR spectrum of 2-((2*S*,6*S*)-6-methyl-6-phenyltetrahydro-2*H*-pyran-2-yl)-1-phenylethan-1-one (**42'**).

HPLC Chromatogram Profiles

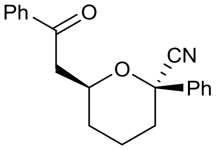

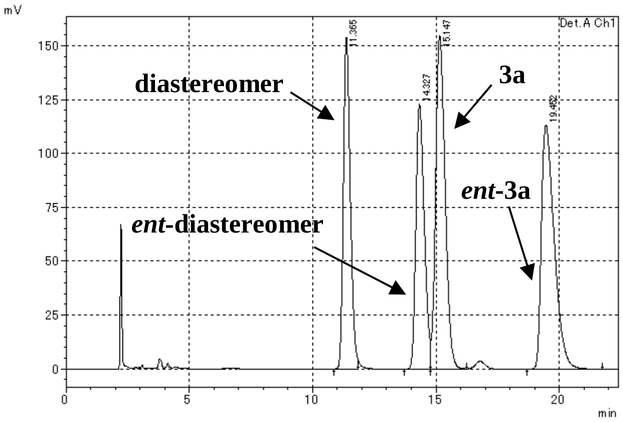

peak	retention time (min)	area %
1	11.365	21.302
2	14.327	20.573
3	15.147	29.107
4	19.452	29.018

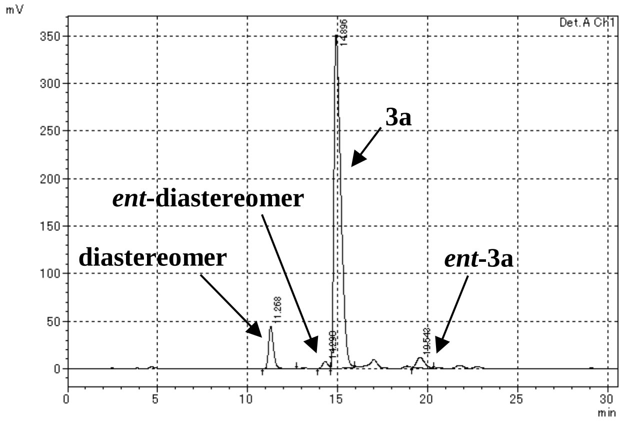

peak	retention time (min)	area %
1	11.268	7.723
2	14.290	1.385
3	14.896	87.853
4	19.543	3.039

Supplementary Figure 100. HPLC chromatogram profiles of (2*R*,6*S*)-6-(2-oxo-2-phenylethyl)-2-phenyltetrahydro-2*H*-pyran-2-carbonitrile (**3a**).

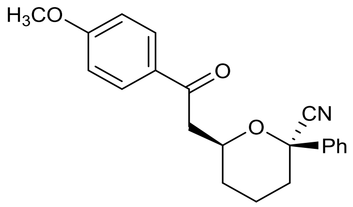

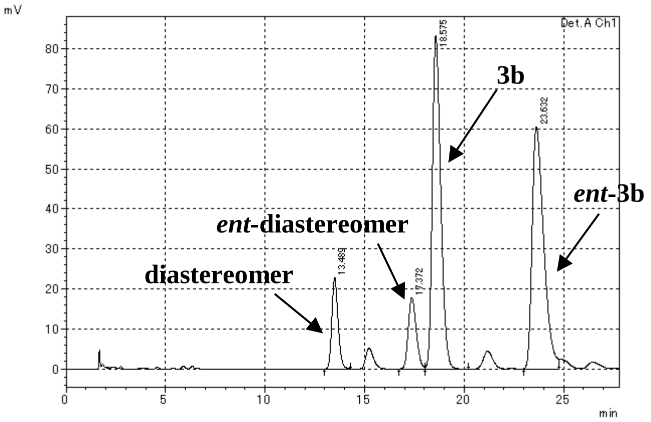

peak	retention time (min)	area %
1	13.489	8.443
2	17.372	8.396
3	18.575	41.721
4	23.632	41.440

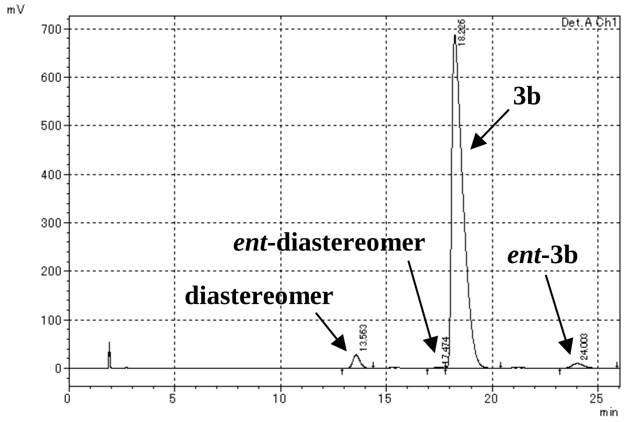

peak	retention time (min)	area %
1	13.563	2.413
2	17.474	0.162
3	18.226	95.842
4	24.003	1.583

Supplementary Figure 101. HPLC chromatogram profiles of 6-(2-(4-methoxyphenyl)-2-oxoethyl)-2-phenyltetrahydro-2*H*-pyran-2-carbonitrile (**3b**).

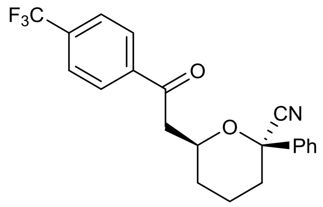

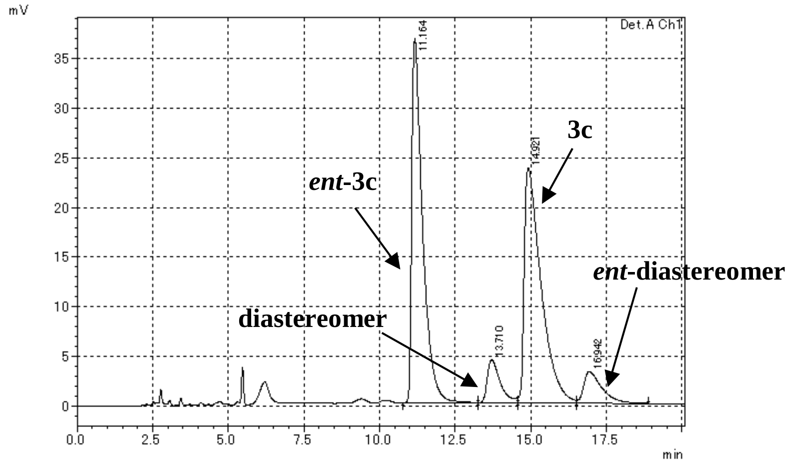

peak	retention time (min)	area %
1	11.164	43.677
2	13.710	6.276
3	14.921	43.662
4	16.942	6.384

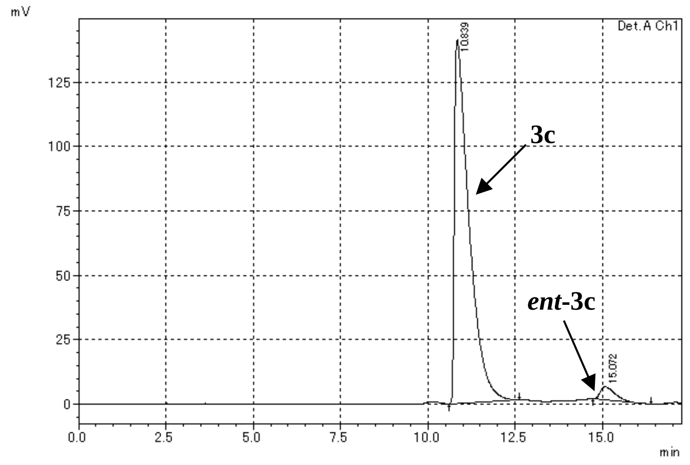

peak	retention time (min)	area %
1	10.839	96.604
2	15.072	3.396

Supplementary Figure 102. HPLC chromatogram profiles of 6-(2-oxo-2-(4-(trifluoromethyl)phenyl)ethyl)-2-phenyltetrahydro-2*H*-pyran-2-carbonitrile (**3c**).

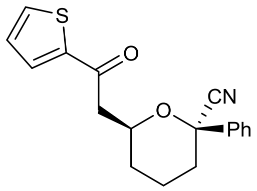

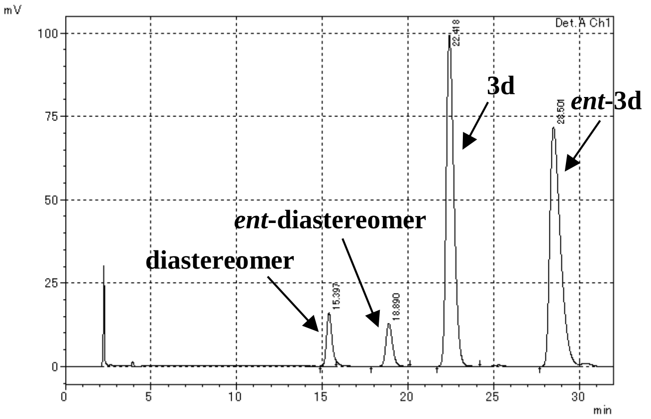

peak	retention time (min)	area %
1	15.397	5.048
2	18.890	4.917
3	22.418	44.916
4	28.501	45.119

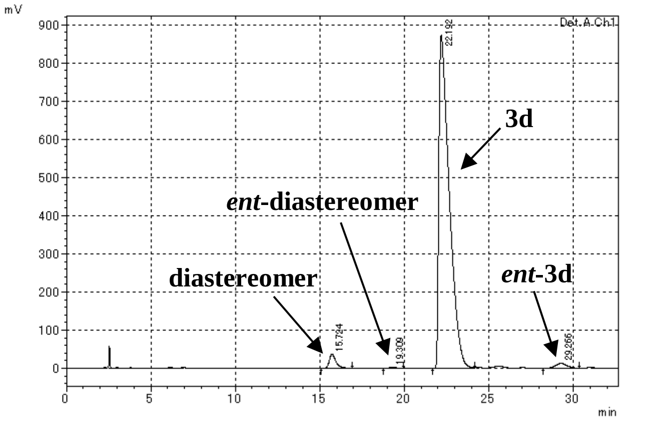

peak	retention time (min)	area %
1	15.724	2.601
2	19.309	0.161
3	22.192	95.597
4	29.266	1.641

Supplementary Figure 103. HPLC chromatogram profiles of 6-(2-oxo-2-(thiophen-2-yl)ethyl)-2-phenyltetrahydro-2*H*-pyran-2-carbonitrile (**3d**).

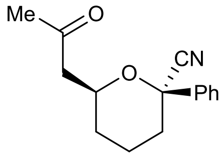

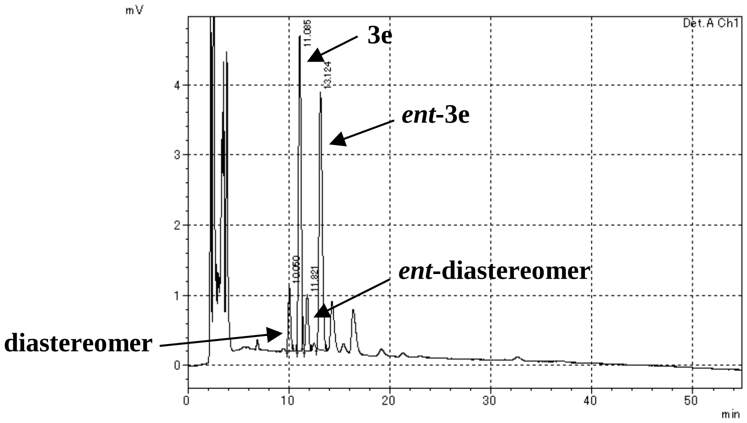

peak	retention time (min)	area %
1	10.050	7.890
2	11.085	42.293
3	11.821	7.692
4	13.124	42.125

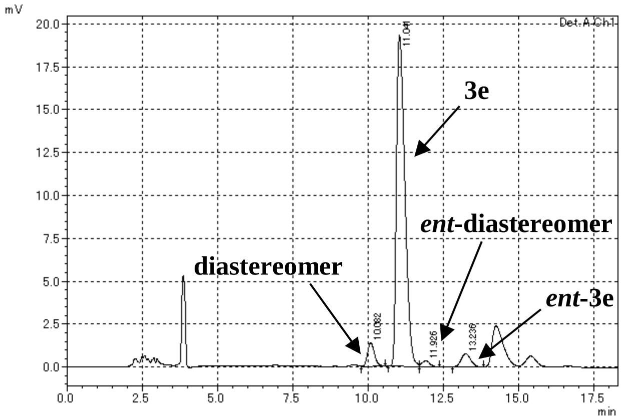

peak	retention time (min)	area %
1	10.082	5.164
2	11.041	89.318
3	11.926	1.459
4	13.236	4.059

Supplementary Figure 104. HPLC chromatogram profiles of 6-(2-oxopropyl)-2-phenyltetrahydro-2*H*-pyran-2-carbonitrile (**3e**).

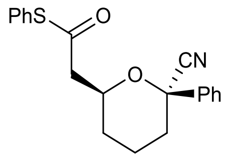

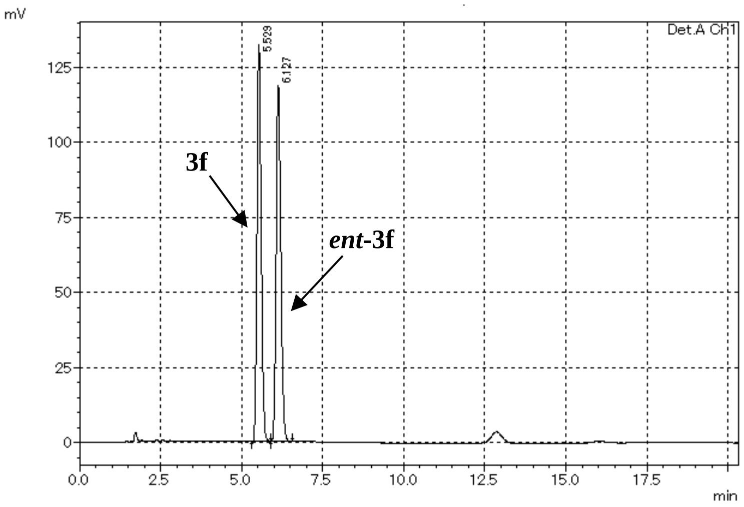

peak	retention time (min)	area %
1	5.529	50.132
2	6.127	49.868

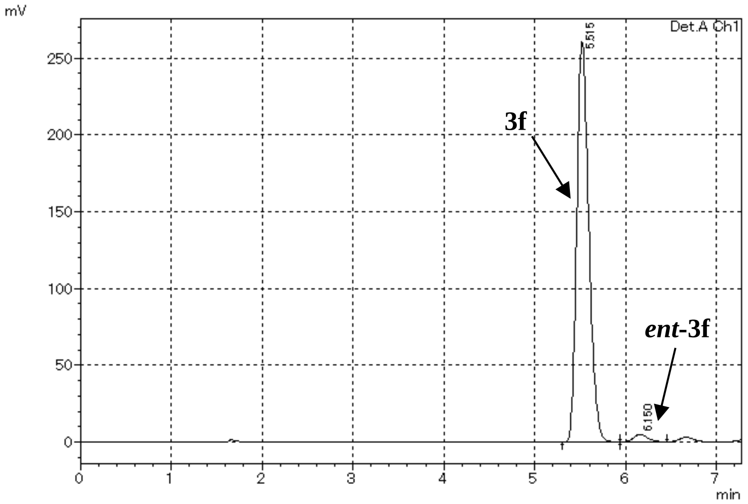

peak	retention time (min)	area %
1	5.515	97.886
2	6.150	2.114

Supplementary Figure 105. HPLC chromatogram profiles of *S*-phenyl 2-(6-cyano-6-phenyltetrahydro-2*H*-pyran-2-yl)ethanethioate (**3f**).

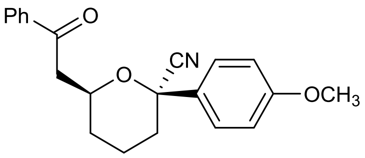

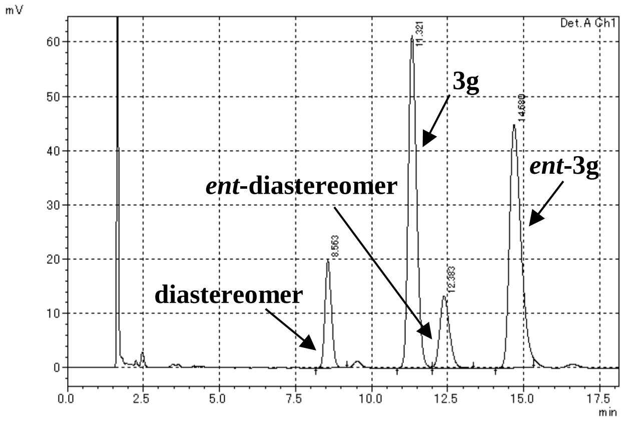

peak	retention time (min)	area %
1	8.563	9.912
2	11.321	39.954
3	12.383	9.995
4	14.680	40.139

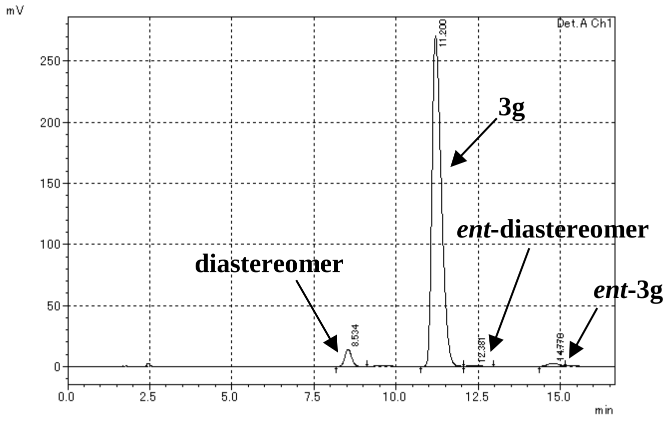

peak	retention time (min)	area %
1	8.534	3.593
2	11.200	94.778
3	12.381	0.408
4	14.770	1.221

Supplementary Figure 106. HPLC chromatogram profiles of 2-(4-methoxyphenyl)-6-(2-oxo-2-phenylethyl)tetrahydro-2*H*-pyran-2-carbonitrile (**3g**).

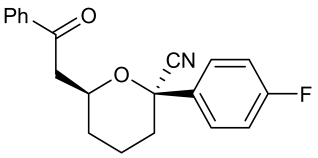

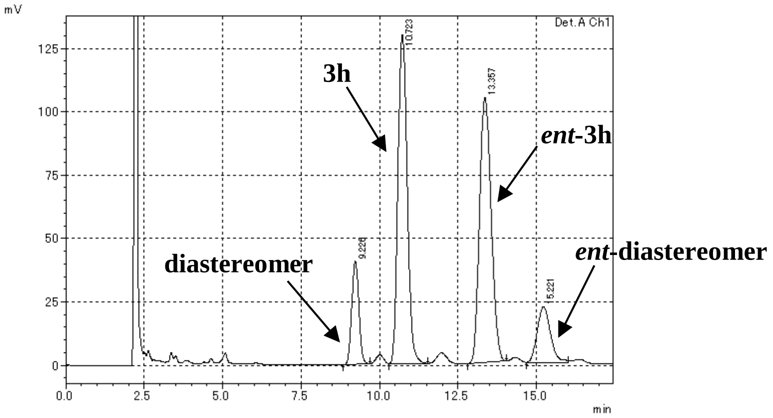

peak	retention time (min)	area %
1	9.226	9.714
2	10.723	40.115
3	13.357	40.531
4	15.221	9.640

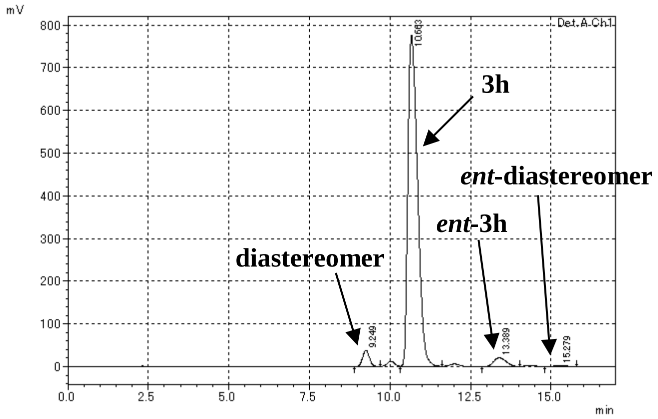

peak	retention time (min)	area %
1	9.249	3.224
2	10.663	93.427
3	13.389	3.065
4	15.279	0.283

Supplementary Figure 107. HPLC chromatogram profiles of 2-(4-fluorophenyl)-6-(2-oxo-2-phenylethyl)tetrahydro-2*H*-pyran-2-carbonitrile (**3h**).

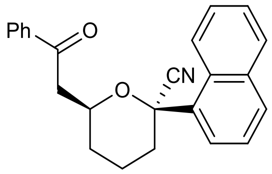

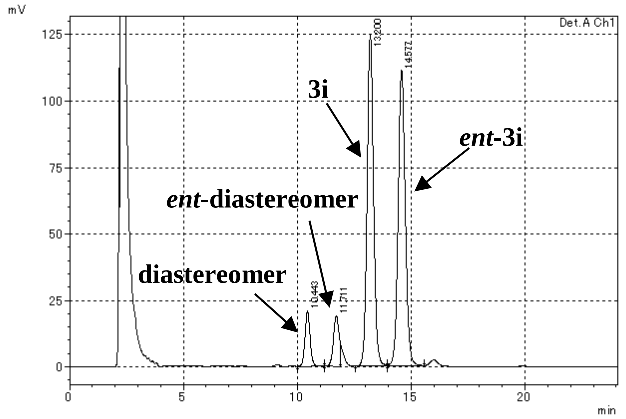

peak	retention time (min)	area %
1	10.443	5.875
2	11.711	5.923
3	13.200	44.242
4	14.577	43.960

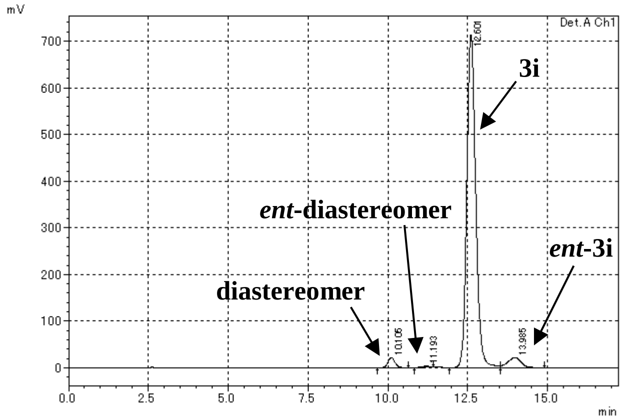

peak	retention time (min)	area %
1	10.105	2.255
2	11.193	0.266
3	12.601	93.270
4	13.985	4.210

Supplementary Figure 108. HPLC chromatogram profiles of 2-(naphthalen-1-yl)-6-(2-oxo-2-phenylethyl)tetrahydro-2*H*-pyran-2-carbonitrile (**3i**).

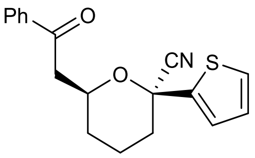

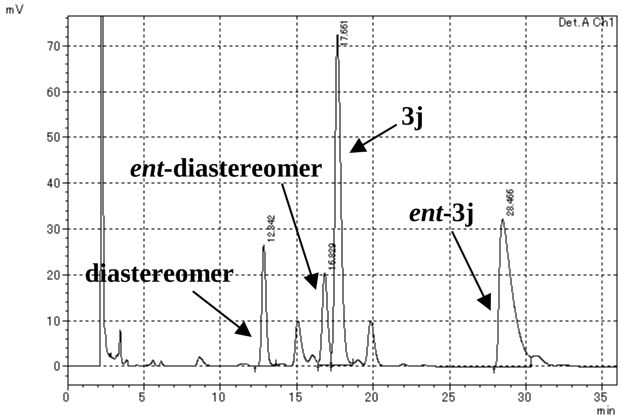

peak	retention time (min)	area %
1	12.842	10.448
2	16.829	10.165
3	17.661	39.988
4	28.466	39.399

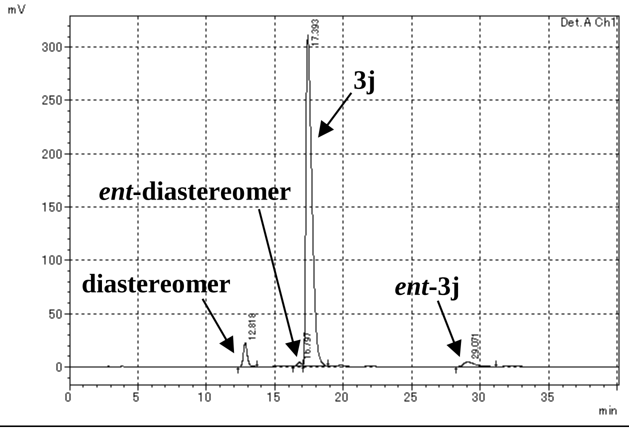

peak	retention time (min)	area %
1	12.818	4.278
2	16.797	0.812
3	17.393	92.565
4	29.071	2.345

Supplementary Figure 109. HPLC chromatogram profiles of 6-(2-oxo-2-phenylethyl)-2-(thiophen-2-yl)tetrahydro-2*H*-pyran-2-carbonitrile (**3j**).

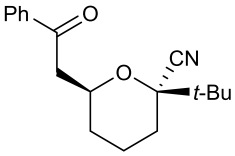

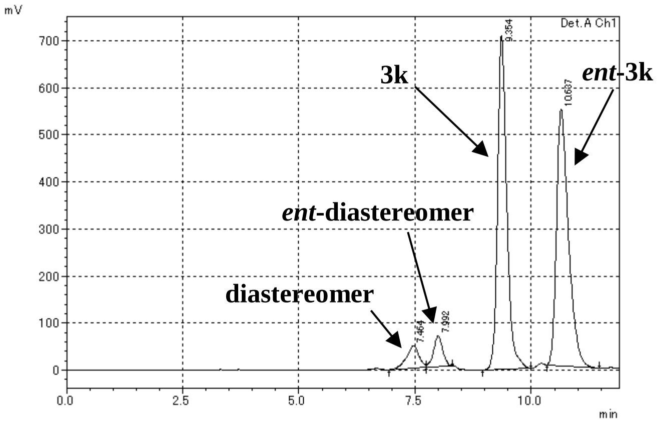

peak	retention time (min)	area %
1	7.464	4.202
2	7.992	4.182
3	9.354	45.740
4	10.637	45.876

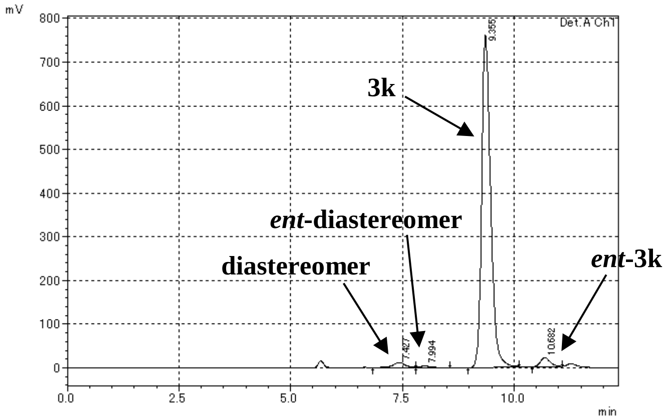

peak	retention time (min)	area %
1	7.427	2.183
2	7.994	0.666
3	9.355	94.322
4	10.682	2.829

Supplementary Figure 110. HPLC chromatogram profiles of 2-(*tert*-butyl)-6-(2-oxo-2-phenylethyl)tetrahydro-2*H*-pyran-2-carbonitrile (**3k**).

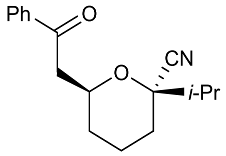

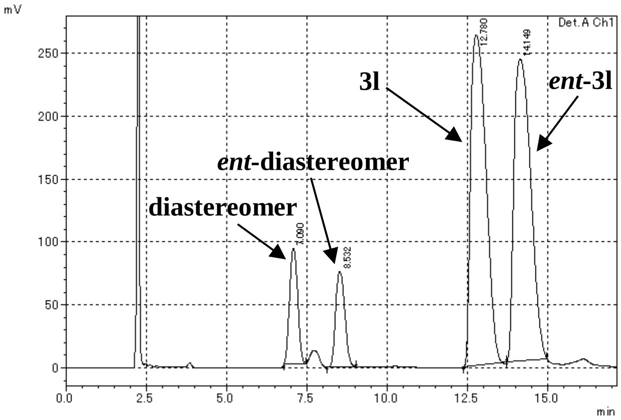

peak	retention time (min)	area %
1	7.090	7.876
2	8.532	7.282
3	12.780	42.342
4	14.149	42.500

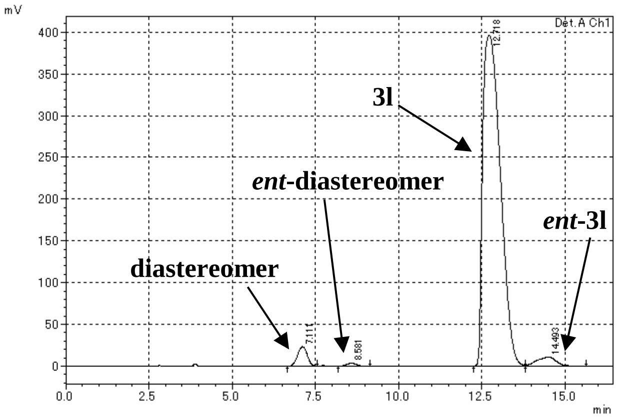

peak	retention time (min)	area %
1	7.111	2.983
2	8.581	0.399
3	12.718	93.982
4	14.493	2.636

Supplementary Figure 111. HPLC chromatogram profiles of 2-isopropyl-6-(2-oxo-2-phenylethyl)tetrahydro-2*H*-pyran-2-carbonitrile (**3l**).

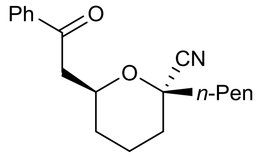

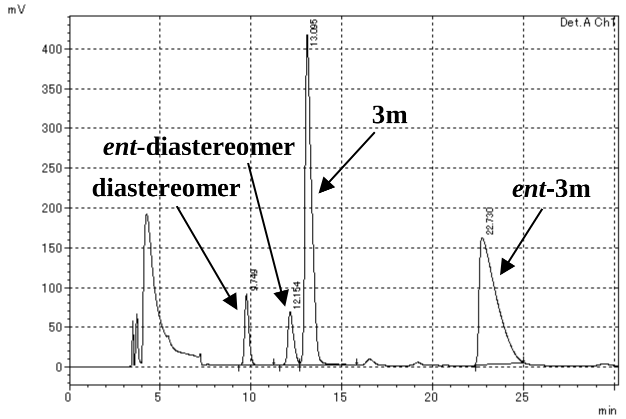

peak	retention time (min)	area %
1	9.749	6.152
2	12.154	6.142
3	13.095	43.702
4	22.730	44.004

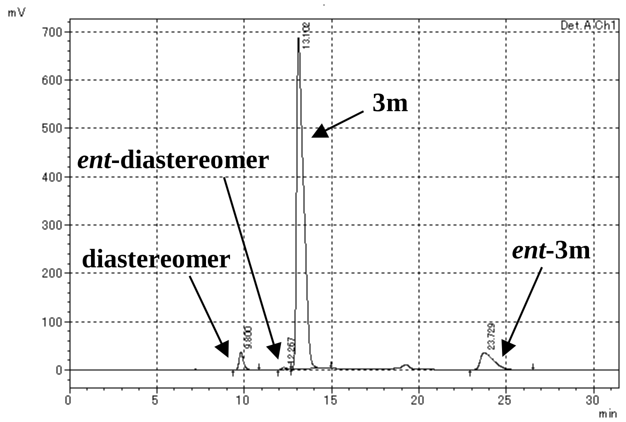

peak	retention time (min)	area %
1	9.800	2.889
2	12.267	0.411
3	13.102	88.066
4	23.729	8.635

Supplementary Figure 112. HPLC chromatogram profiles of 6-(2-oxo-2-phenylethyl)-2-pentyltetrahydro-2*H*-pyran-2-carbonitrile (**3m**).

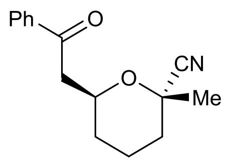

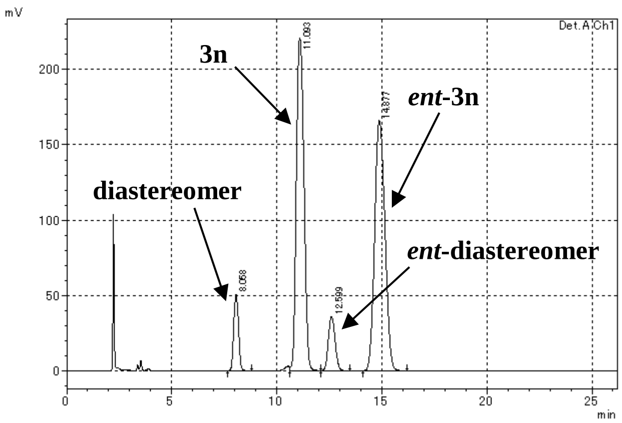

peak	retention time (min)	area %
1	8.058	6.270
2	11.093	43.385
3	12.599	6.272
4	14.877	44.073

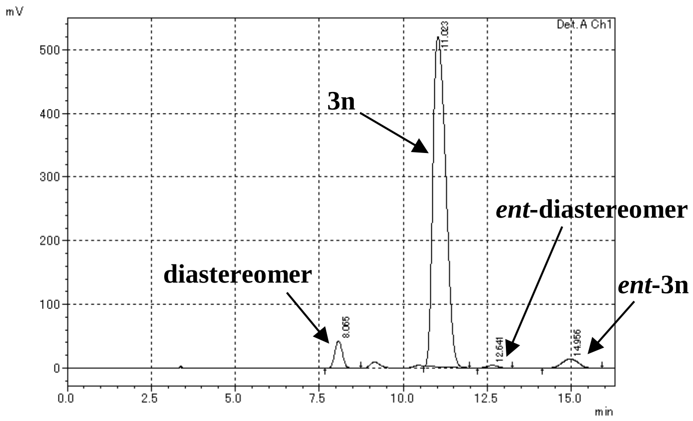

peak	retention time (min)	area %
1	8.065	4.465
2	11.023	91.675
3	12.641	0.629
4	14.956	3.232

Supplementary Figure 113. HPLC chromatogram profiles of 2-methyl-6-(2-oxo-2-phenylethyl)tetrahydro-2*H*-pyran-2-carbonitrile (**3n**).

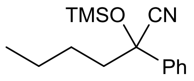

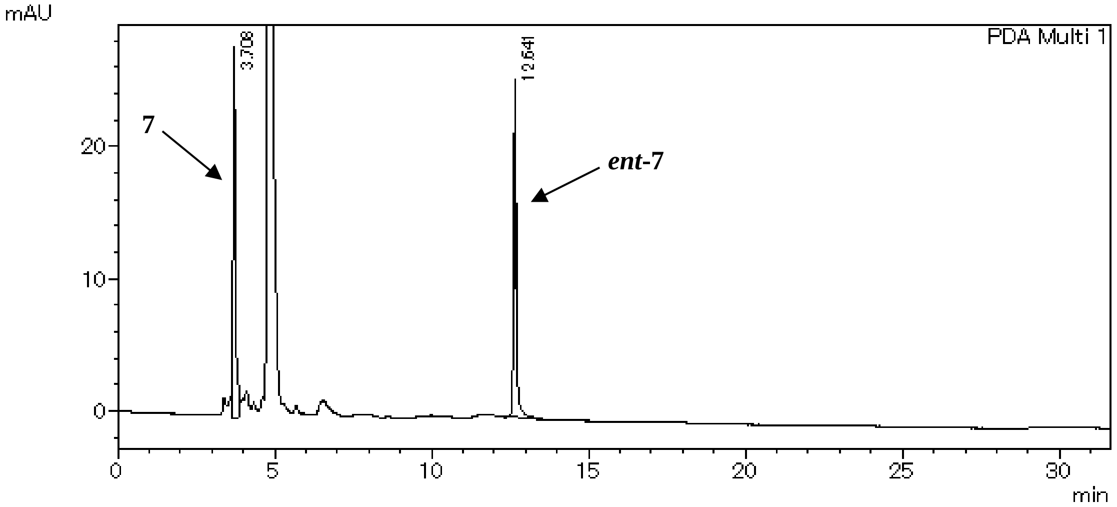

peak	retention time (min)	area %
1	3.708	50.655
2	12.641	49.345

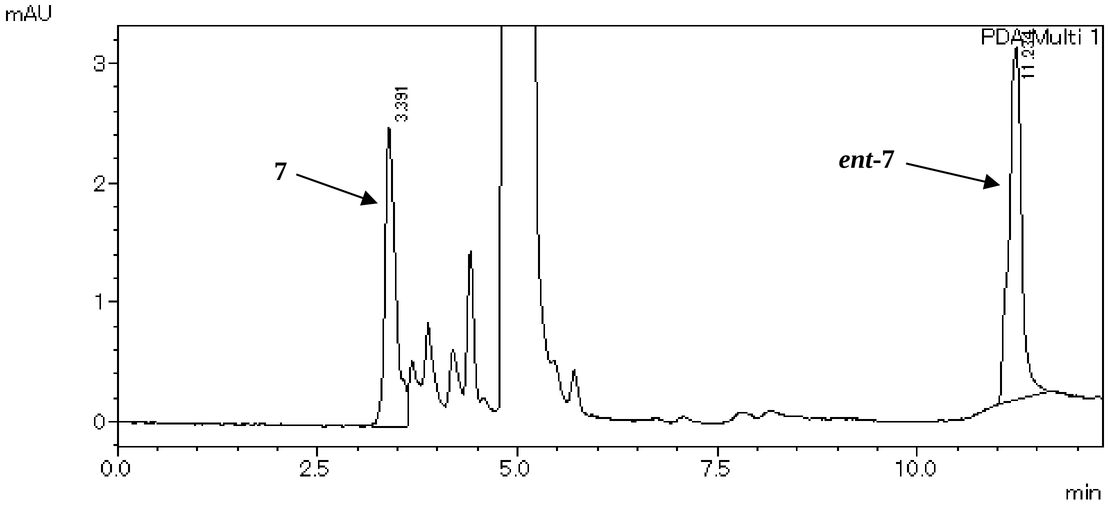

peak	retention time (min)	area %
1	3.391	42.175
2	11.234	57.825

Supplementary Figure 114. HPLC chromatogram profiles of 2-phenyl-2-((trimethylsilyl)oxy)hexanenitrile (**7**).

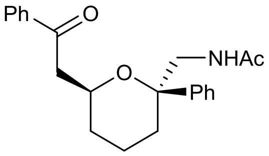

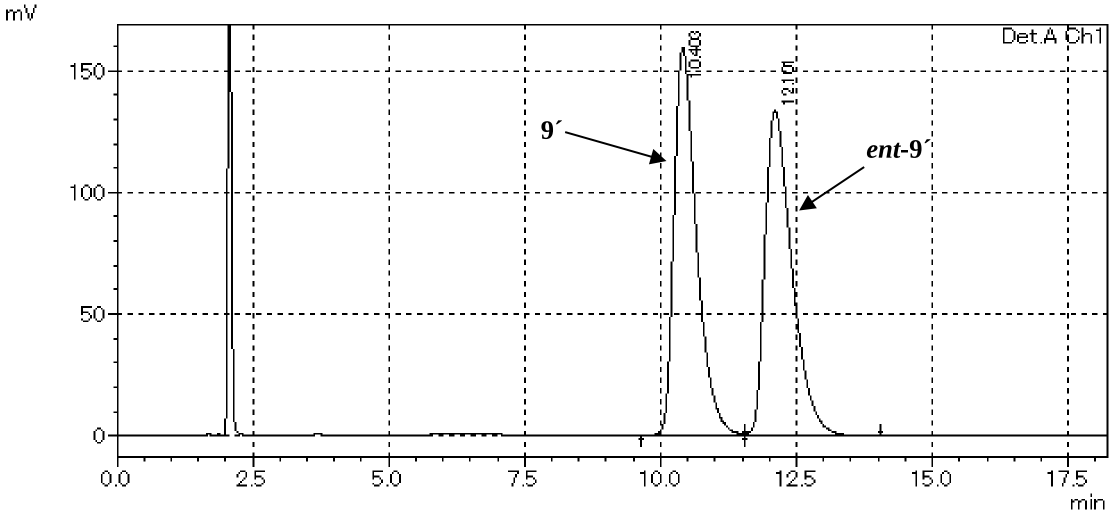

peak	retention time (min)	area %
1	10.403	49.954
2	12.101	50.046

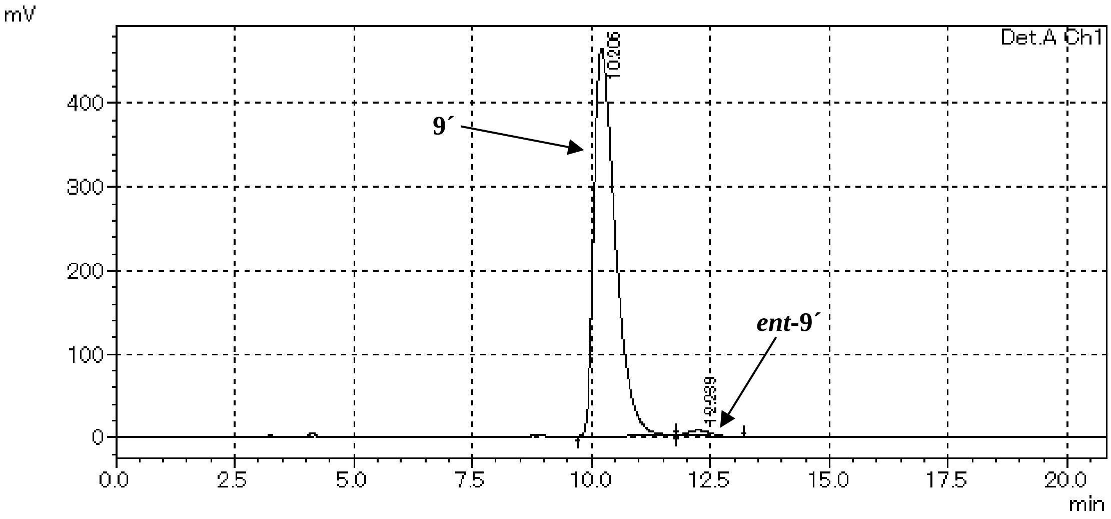

peak	retention time (min)	area %
1	10.206	98.698
2	12.239	1.302

Supplementary Figure 115. HPLC chromatogram profiles of *N*-((6-(2-oxo-2-phenylethyl)-2-phenyltetrahydro-2*H*-pyran-2-yl)methyl)acetamide (**9´**).

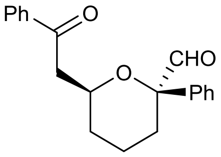

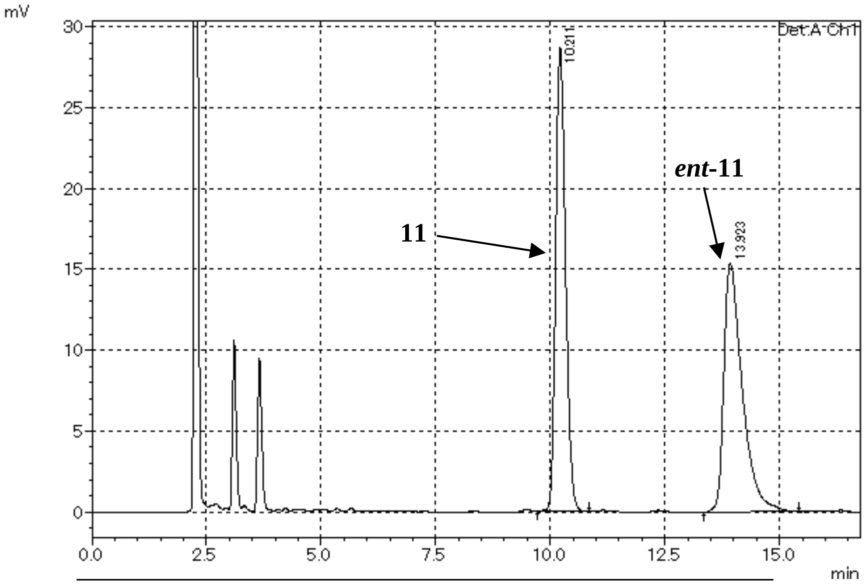

peak	retention time (min)	area %
1	10.211	49.863
2	13.923	50.137

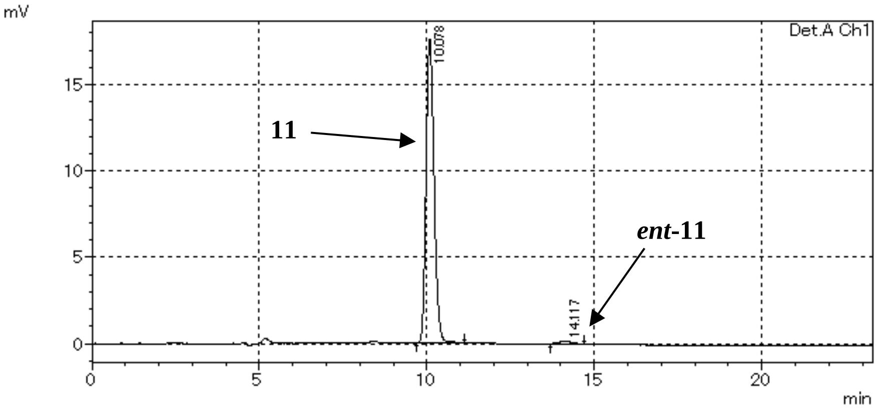

peak	retention time (min)	area %
1	10.078	98.523
2	14.117	1.477

Supplementary Figure 116. HPLC chromatogram profiles of 6-(2-oxo-2-phenylethyl)-2-phenyltetrahydro-2*H*-pyran-2-carbaldehyde (**11**).

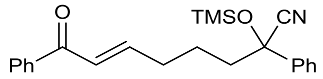

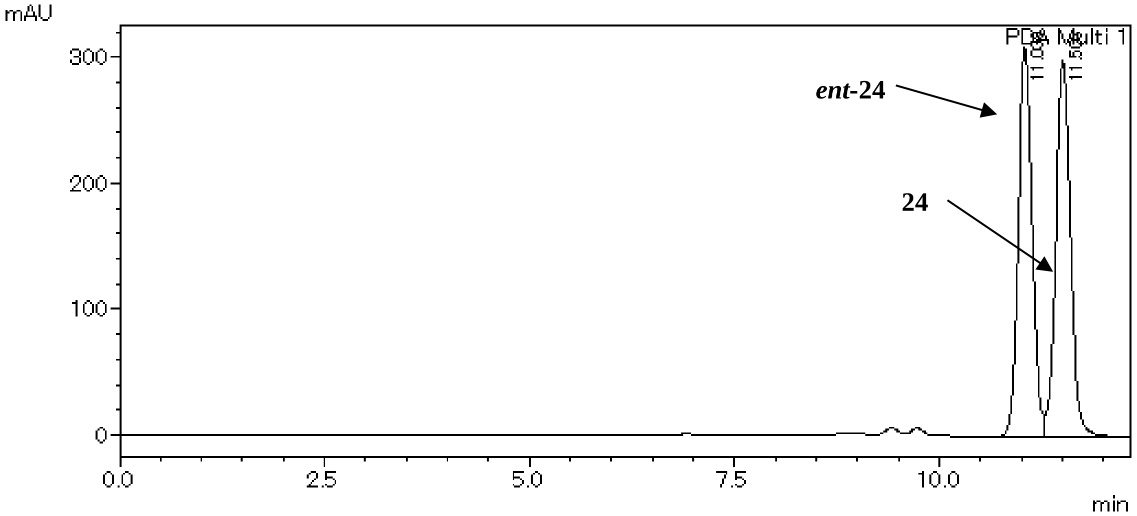

peak	retention time (min)	area %
1	11.039	49.547
2	11.503	50.453

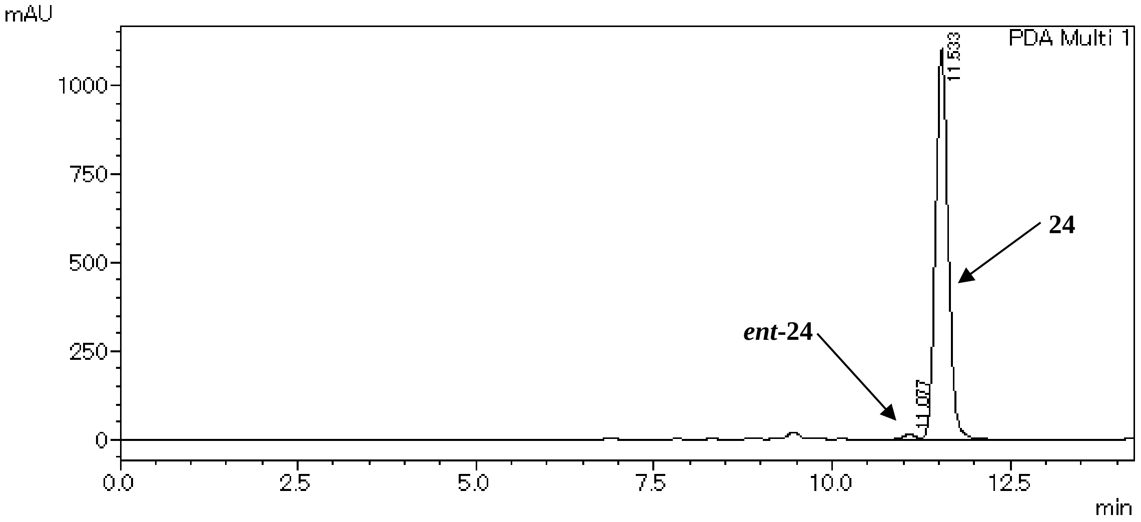

peak	retention time (min)	area %
1	11.077	1.098
2	11.533	98.902

Supplementary Figure 117. HPLC chromatogram profiles of (*E*)-8-oxo-2,8-diphenyl-2-((trimethylsilyl)oxy)oct-6-enenitrile (**39**).

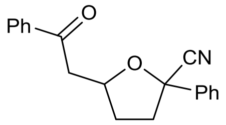

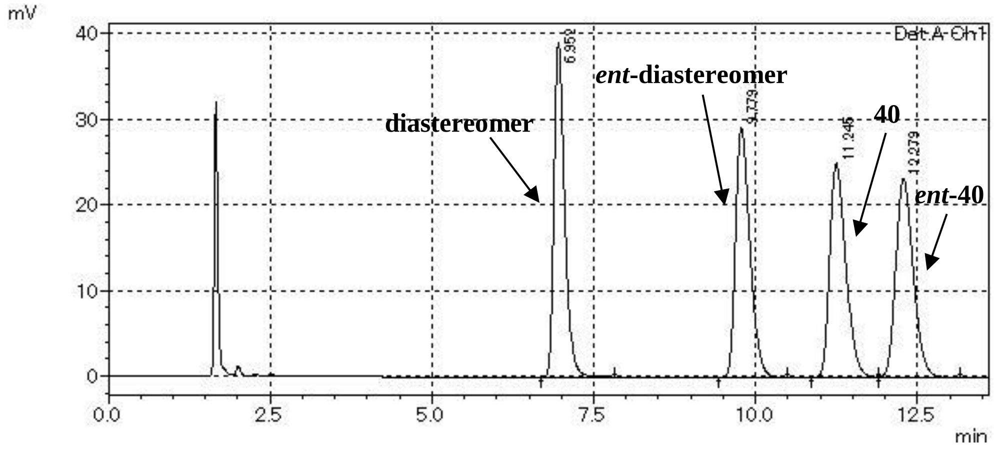

peak	retention time (min)	area %
1	6.952	25.490
2	9.779	25.602
3	11.245	24.448
4	12.279	24.460

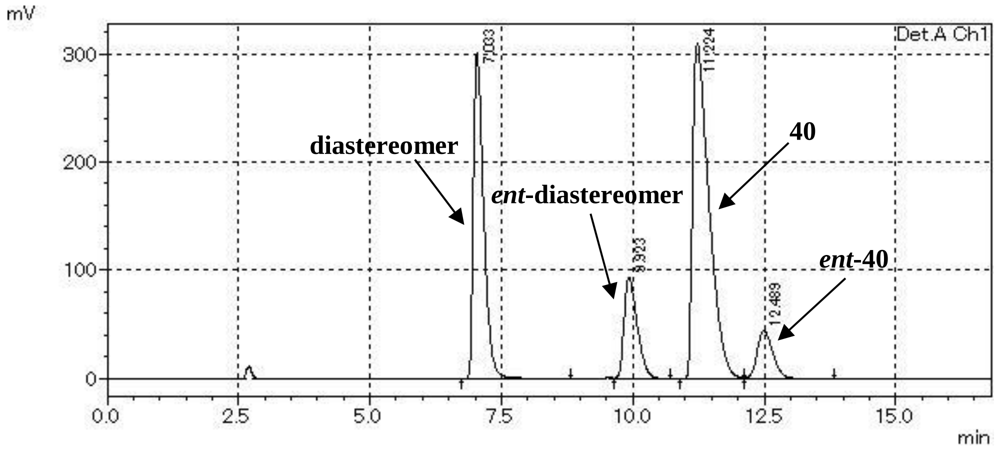

peak	retention time (min)	area %
1	7.033	30.682
2	9.923	12.075
3	11.224	50.357
4	12.489	6.886

Supplementary Figure 118. HPLC chromatogram profiles of 5-(2-oxo-2-phenylethyl)-2-phenyltetrahydrofuran-2-carbonitrile (**40**).

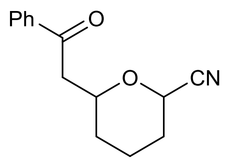

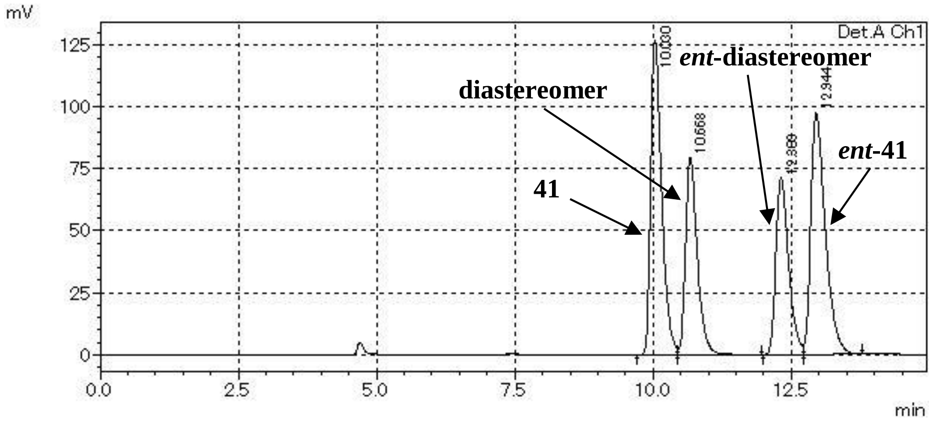

peak	retention time (min)	area %
1	10.030	30.004
2	10.668	20.099
3	12.309	19.519
4	12.944	30.378

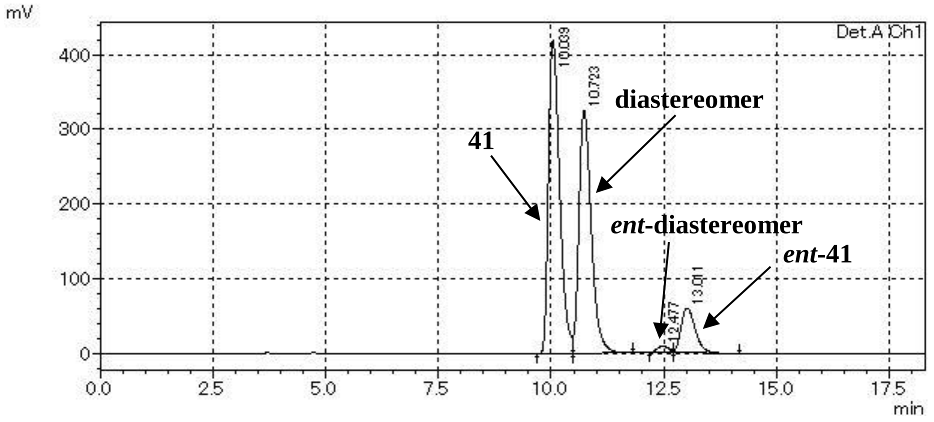

peak	retention time (min)	area %
1	10.039	48.685
2	10.723	41.536
3	12.477	1.115
4	13.011	8.665

Supplementary Figure 119. HPLC chromatogram profiles of 6-(2-oxo-2-phenylethyl)tetrahydro-2*H*-pyran-2-carbonitrile (**41**).

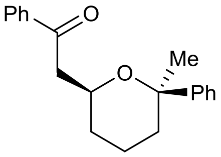

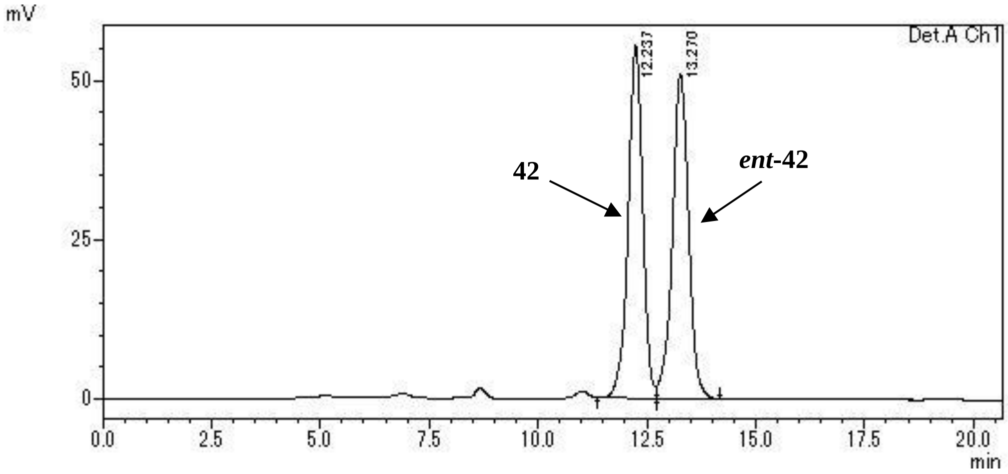

peak	retention time (min)	area %
1	12.237	49.448
2	13.270	50.552

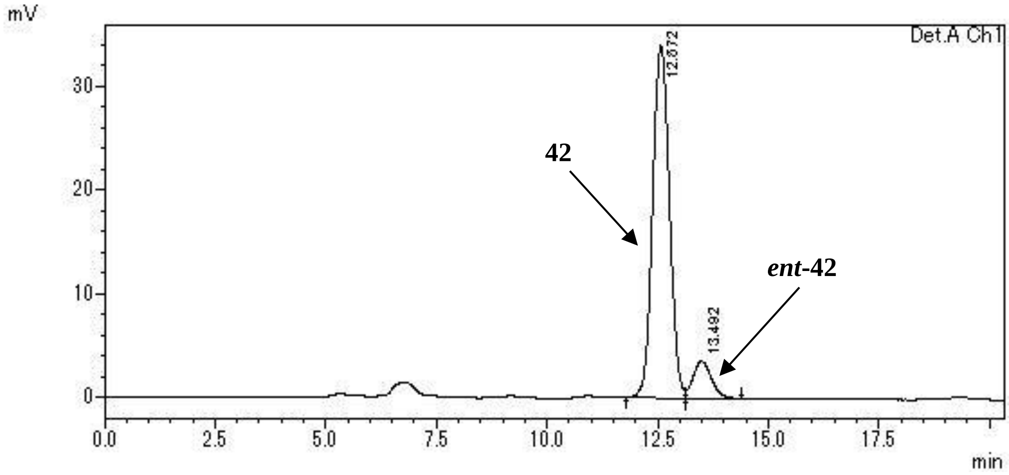

peak	retention time (min)	area %
1	12.572	89.540
2	13.492	10.460

Supplementary Figure 120. HPLC chromatogram profiles of 2-((2*S*,6*R*)-6-methyl-6-phenyltetrahydro-2*H*-pyran-2-yl)-1-phenylethan-1-one (**42**).

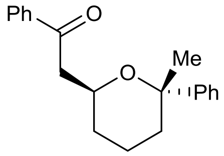

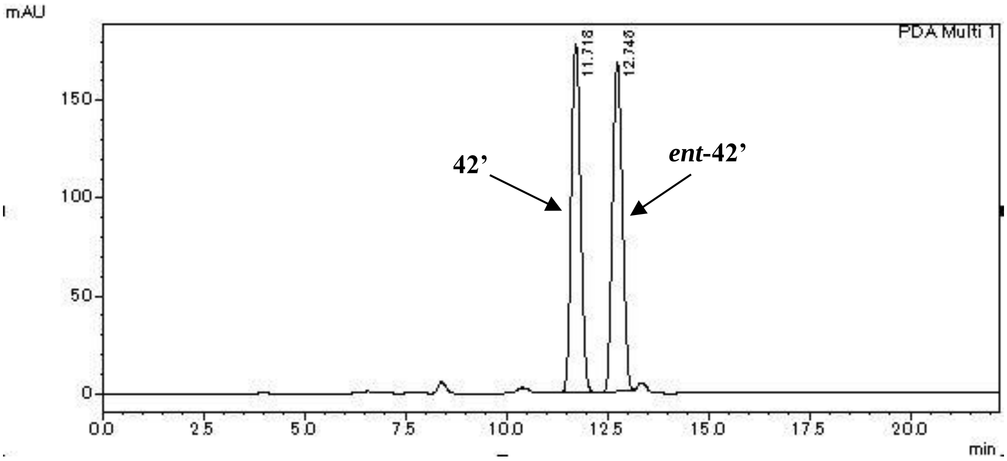

peak	retention time (min)	area %
1	11.728	50.267
2	12.745	49.733

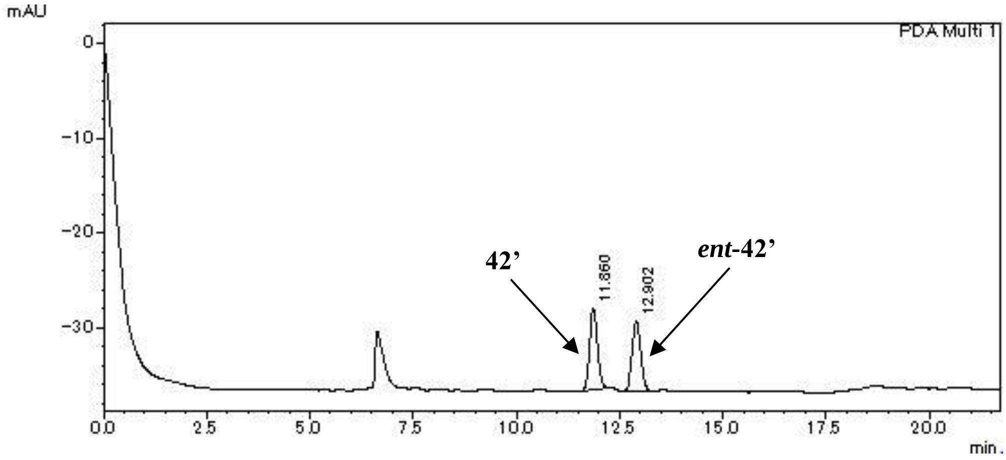

peak	retention time (min)	area %
1	11.860	52.393
2	12.902	47.607

Supplementary Figure 121. HPLC chromatogram profiles of 2-((2*S*,6*S*)-6-methyl-6-phenyltetrahydro-2*H*-pyran-2-yl)-1-phenylethan-1-one (**42’**).

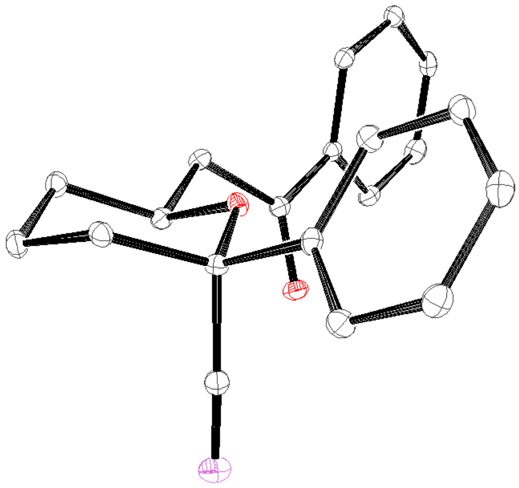

Supplementary Figure 122. ORTEP drawing of **3a**.

Supplementary Note 1

A. Crystal Data

Empirical Formula	$C_{20}H_{19}NO_2$
Formula Weight	305.38
Crystal Color, Habit	Colorless, Needle
Crystal Dimensions	0.560 × 0.090 × 0.090 mm
Crystal System	Orthorhombic
Lattice Type	Primitive
Lattice Parameters	a = 10.7965(3) Å
	b = 11.3690(3) Å
	c = 12.8070(4) Å
	V = 1572.00(7) $Å^3$
Space Group	$P2_12_12_1$ (#19)
Z value	4
D_{calc}	1.290 g/cm^3
F_{000}	648.00
μ(CuKα)	6.604 cm^{-1}

B. Intensity Measurements

Diffractometer	R-AXIS RAPID
Radiation	CuKα (λ = 1.54187 Å)
	Multi-layer mirror monochromated
Voltage, Current	40kV, 30mA
Temperature	–180.0 °C
Detector Aperture	460.0 × 256.0 mm
Data Images	90 exposures
ω Oscillation Range (χ = 54.0, φ = 0.0)	80.0–260.0°
Exposure Rate	4.0 sec./°
ω Oscillation Range (χ = 54.0, φ = 90.0)	80.0–260.0°
Exposure Rate	4.0 sec./°
ω Oscillation Range (χ = 54.0, φ = 180.0)	80.0–260.0°
Exposure Rate	4.0 sec./°
ω Oscillation Range (χ = 54.0, φ = 270.0)	80.0–260.0°
Exposure Rate	4.0 sec./°
ω Oscillation Range (χ = 0.0, φ = 0.0)	80.0–260.0°
Exposure Rate	4.0 sec./°
Detector Position	127.40 mm
Pixel Size	0.100 mm
$2\theta_{max}$	136.4°
No. of Reflections Measured	Total: 17920
	Unique: 2873 (R_{int} = 0.0447)
	Parsons quotients (Flack x parameter): 1192
Corrections	Lorentz-polarization
	Absorption
	(trans. factors: 0.699–0.942)

C. Structure Solution and Refinement

Structure Solution	Direct Methods (SHELXT)
Refinement	Full-matrix least-squares on F^2
Function Minimized	Σ w $(F_0^2 – F_c^2)^2$

Least Squares Weights	$w = 1/[\sigma^2(F_0^2) + (0.0549 \cdot P)^2$ $+ 0.2889 \cdot P]$ where $P = (Max(F_0^2,0) + 2F_c^2)/3$
$2\theta_{max}$ cutoff	136.4°
Anomalous Dispersion	All non-hydrogen atoms
No. Observations (All reflections)	2873
No. Variables	208
Reflection/Parameter Ratio	13.81
Residuals: R1 ($I>2.00\sigma(I)$)	0.0362
Residuals: R (All reflections)	0.0364
Residuals: wR2 (All reflections)	0.0956
Goodness of Fit Indicator	1.218
Flack parameter (Parsons' quotients = 1192)	–0.04(4)
Max Shift/Error in Final Cycle	0.000
Maximum peak in Final Diff. Map	0.22 $e^-/Å^3$
Minimum peak in Final Diff. Map	–0.34 $e^-/Å^3$

Supplementary References

1. Vakulya, B., Varga, S., Csámpai, A. & Soós, T. Highly enantioselective conjugate addition of nitromethane to chalcones using bifunctional cinchona organocatalysts. *Org. Lett.* **7,** 1967–1969 (2005).
2. Gharpure, S. J., Prasad, J. V. K. & Bera, K. Tandem nucleophilic addition/oxa-Michael reaction for the synthesis of *cis*-2,6-disubstituted tetrahydropyrans. *Eur. J. Org. Chem.* 3570–3574 (2014).
3. Hamashima, Y., Kanai, M. & Shibasaki, M. Catalytic enantioselective cyanosilylation of ketones. *J. Am. Chem. Soc.* **122,** 7412–7413 (2000).
4. Fuerst, D. E. & Jacobsen, E. N. Thiourea-catalyzed enantioselective cyanosilylation of ketones. *J. Am. Chem. Soc.* **127,** 8964–8965 (2005).
